# Enantioselective and Diastereoselective Synthesis of Azaspiro[n.2]alkanes by Rhodium-Catalyzed Cyclopropanations

**DOI:** 10.1021/acscatal.5c04199

**Published:** 2025-08-19

**Authors:** Joshua K. Sailer, Duc Ly, Andrew Wang, Djamaladdin G. Musaev, Huw M. L. Davies

**Affiliations:** † Department of Chemistry, 1371 Emory University, 1515 Dickey Drive, Atlanta, Georgia 30322, United States; ∥ Cherry L. Emerson Center for Scientific Computation, 1371 Emory University, Atlanta, Georgia 30322, United States

**Keywords:** spiro compounds, asymmetric catalysis, cyclopropanation, catalyst-substrate recognition, dirhodium, carbene

## Abstract

Spirocyclopropanes have become a prevalent structural motif in drug discovery campaigns. However, methods to generate these spirocyclopropanes stereoselectively are scarce, highlighting the need for efficient synthetic methods. In this report, we describe the synthesis of various azaspiro­[n.2]­alkanes by means of a dirhodium tetracarboxylate catalyzed cyclopropanation of exocyclic olefins using donor/acceptor carbenes. The optimum chiral dirhodium tetracarboxylate catalyst, Rh_2_(*p*-PhTPCP)_4_, results in highly enantioselective cyclopropanation of symmetrical azacyclomethylidenes and highly enantioselective and diastereoselective cyclopropanation of nonsymmetrical azacyclomethylidenes and can achieve up to 83,000 turnovers. Computational studies reveal that the stereoselectivity is controlled by the way the substrate can fit into the chiral pocket generated by the ligands.

## Graphical abstract

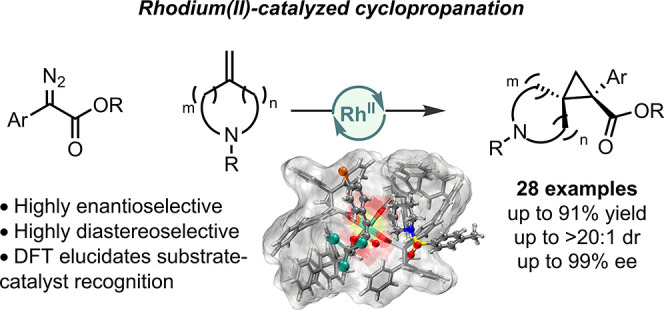

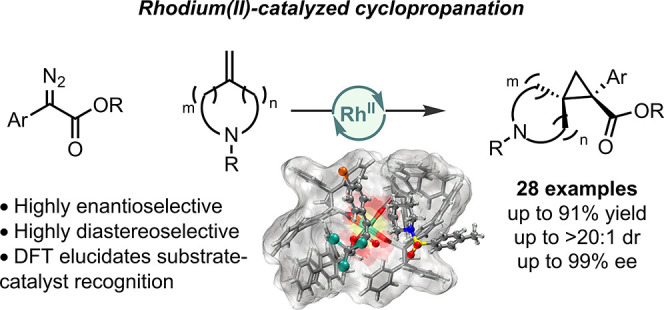

## Introduction

The cyclopropyl ring has become a common unit in many pharmaceutical drugs because it has a rigid structure, placing the substituents in a defined position. The cyclopropane motif in many of these drugs are monosubstituted or 1,1-disubstitued as this avoids challenges associated with the synthesis of chiral cyclopropanes.^1,2^ With greater emphasis by the pharmaceutical industry in recent years to move away from “flat-land” and embrace complex three-dimensional structures, more highly functionalized chiral cyclopropane scaffolds are becoming prevalent in drug candidates.^3–5^ One particular class of compounds that has generated considerable recent interest has been azaspiro­[n.2]­alkanes.^4,6–12^ Representative examples are compounds **1**–**3** (Figure 1A). Compound **1**, an HDAC inhibitor, showed good potency and high selectivity, particularly after installing the azaspirocyclopropane motif.^13^ Similarly, compounds **2** and **3** showed promising activity as a human epidermal growth factor receptor-2 (HER-2) inhibitor^14^ and a histamine-3 (H_3_R) antagonist,^15^ respectively. However, the pure enantiomers of **1** and **3** were prepared by chiral resolution,^13,16^ highlighting the need for enantioselective methods to be developed for this general class of compounds.

**Figure 1:**
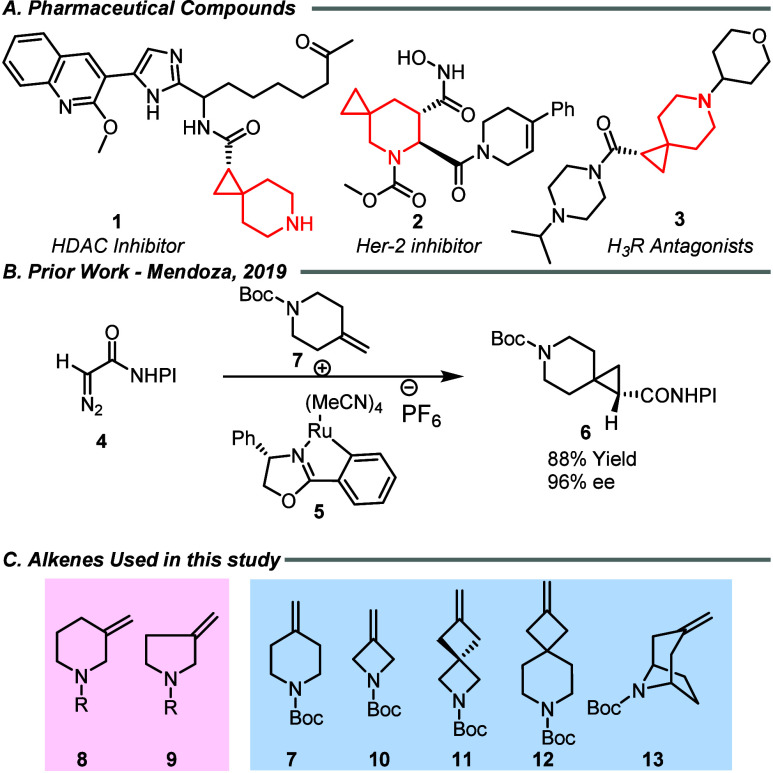
Background and current work.

A number of methods have been reported for the synthesis of azaspiro­[n.2]­alkanes, such as the Corey-Chiakovsky cyclopropanation,^16^ phosphorus-mediated ring closure,^17^ and metal-catalyzed carbene addition,^18,19^ but few enantioselective methods are available.^20^ One such method was reported by Mendoza in 2019 in which the selective cyclopropanation of exocyclic olefins was obtained using a ruthenium Pheox catalyst (Figure 1B).^21^ Another ruthenium catalyst has been applied to the enantioselective synthesis of a pharmaceutically relevant carbospirocyclopropane, but the reaction required a 5% catalyst loading.^22^ The highly selective cyclopropanation with a dirhodium paddle-wheel catalyst to generate a spiro compound was also reported.^23,24^ However, those studies mainly focused only on symmetrical 1,1-disubstituted alkenes due to diastereoselective challenges of the unsymmetrical one. With the current state of the field, the development of highly diastereoselective and enantioselective methods to readily access highly functionalized azaspiro­[n.2]­alkanes would greatly enhance the viability of such compounds as potential drug candidates.

Our group has a long-standing interest in rhodium-catalyzed cyclopropanation with donor/acceptor carbenes,^25^ and so, we decided to challenge our system to determine if it could be used to achieve stereoselective synthesis of a variety of novel chiral azaspiro­[n.2]­alkanes. The study explored two classes of compounds: nonsymmetrical azacyclomethylidienes (**8**, **9**) and symmetrical azacyclomethylidenes (**7**, **10**–**13**) (Figure 1C). The diastereoselectivity in the first set would be expected to be quite challenging for small transition metal catalysts, because the differentiation between the two substituents on the alkene occurs distal to the alkene. It is likely that secondary interactions away from the reactive site would be required for subtle diastereoselectivity, and this type of feature is more commonly exhibited by enzymes.^26–28^ We have shown, however, that our bowl-shaped catalysts exhibit subtle selectivity due to interactions between the approaching substrate and the wall of the catalyst,^29,30^ and thus, there was a reasonable chance our catalysts could achieve good levels of stereoselectivity.

Dirhodium-catalyzed cyclopropanation of monosubstituted alkenes with donor/acceptor carbenes is a well-established process and has even been applied in the scale-up synthesis of drug candidates.^31–33^ The cyclopropanation of 1,1-diarylethylenes has also been shown to be highly enantioselective,^29^ but the extension to other 1,1-disubstituted alkenes has been limited. One of the hallmarks of the cyclopropanation of monosubstituted alkenes is its highly diastereoselective nature.^25,34^ Furthermore, a series of chiral dirhodium catalysts have been designed so that the reaction can also be achieved with high asymmetric induction.^35^ Some of the most notable chiral catalysts are Rh_2_(DOSP)_4_, which gives high asymmetric induction when the acceptor group is a methyl ester,^36^ Rh_2_(PTAD)_4_, which is effective with many types of acceptor groups,^37^ Rh_2_(TCPTAD)_4_, which is a more rigid version of Rh_2_(PTAD)_4_,^38^ Rh_2_(*p*-PhTPCP)_4_, which is sterically demanding and capable of operating with low catalyst loading (<0.001 mol %),^32^ and the bowl-shaped complex, Rh_2_(TPPTTL)_4_, which is capable of inducing unusual selectivity due to interactions between the substrate and the wall of the bowl.^30,39^ With our broad experience with donor/acceptor carbenes^40^ and a series of the most promising chiral catalysts identified, we were well placed to take on the challenge of developing enantioselective approaches to azaspiro­[n.2]­alkanes.

## Results and Discussion

The study began by applying some of our more successful catalysts in a test reaction using the nonsymmetrical *N*-Boc-3-methylene piperidine (**8a**) as the substrate with 2,2,2-trichloroethyl 2-(4-bromophenyl)-2-diazoacetate (**14a**) as the carbene precursor (Table 1). The trichloroethyl ester is used because many of our recent catalysts give higher levels of asymmetric induction compared to the corresponding methyl ester.^35^ Even though **8a** has an allylic methylene site adjacent to the *N*-Boc group that is activated for C–H functionalization, all of the catalysts were successful at cyclopropanation of **8a** because the 1,1-disubstituted alkene is sterically accessible. This is a nice illustration of the influence of steric factors on the cyclopropanation with donor/acceptor carbenes. Rh_2_(*S*-DOSP)_4_ gave low enantioselectivity and diastereoselectivity on the formation of **15a** (entry 1). The three *C*_4_-symmetric phthalimido derived catalysts gave higher levels of enantioselectivity (70–86% ee) compared to the reactions with **7**, but the diastereoselectivity was still very poor (1.2:1 to 2.4:1 d.r.) (entries 2–4). The only catalyst that gave promising results was Rh_2_(*S*-*p*PhTPCP)_4_, which generated **15a** in 11:1 d.r. with 99% ee.

**Table 1:**
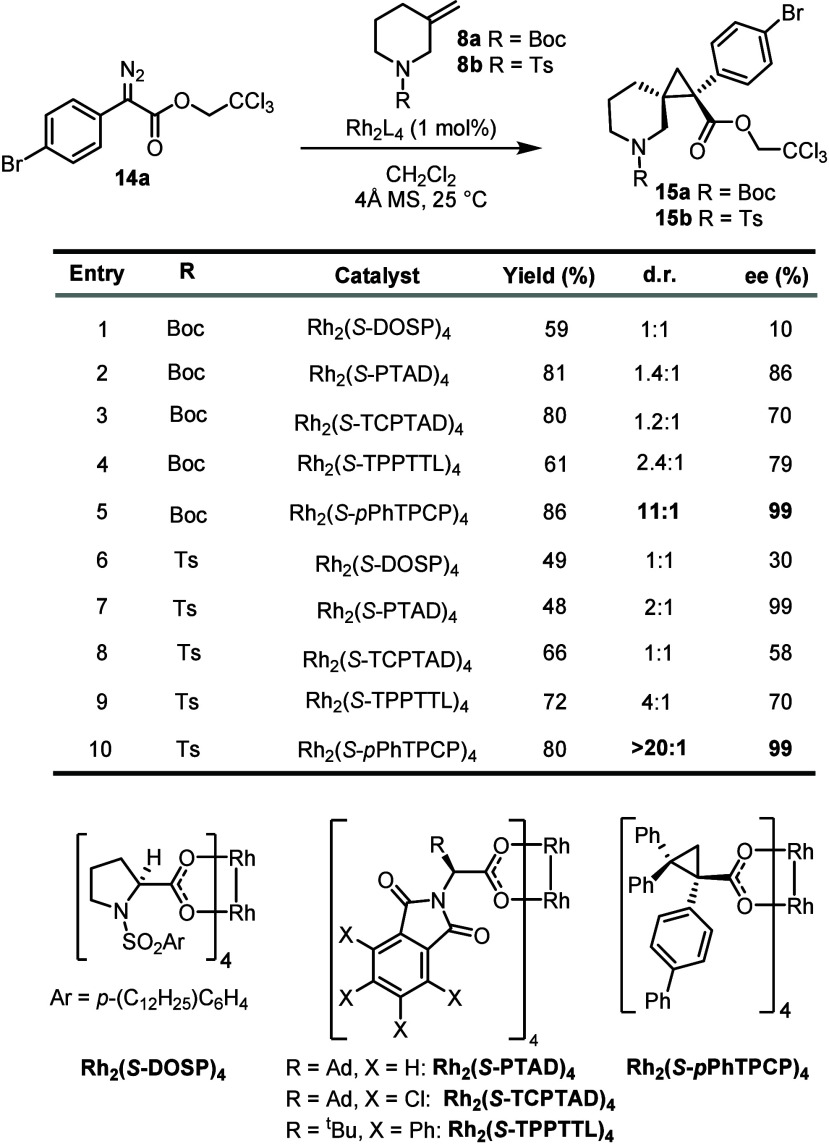
Catalyst Optimization Studies^a^

a Reaction conditions: **14a** (0.2 mmol), **8** (1.5 equiv), CH_2_Cl_2_, 4 Å molecular sieve. Yields are isolated yields. Enantiomeric excess (ee) was determined by HPLC and SFC analysis.

We assumed that switching the protecting group from the Boc group to the Ts group would enable greater interactions with the catalyst wall because the steric environment around the N-center would increase as the trigonal geometry of carbamate changed to the trigonal pyramidal geometry of sulfonamide. The reactions were repeated using the *N*-tosyl derivative **8b** (entries 6–10). A significant improvement was observed with Rh_2_(*S*-*p*PhTPCP)_4_, which generated **15b** in 80% yield, >20:1 d.r., and 99% ee (entry 10). The relative stereochemistry of the major diastereomer of **15** is as drawn and is readily assigned by means of the distinctive shielding of the methylene group cis to the aryl substituent.^25^ On the basis of these optimization studies, it was clear that Rh_2_(*S*-*p*PhTPCP)_4_ was by far the most superior catalyst for high levels of diastereoselectivity and enantioselectivity.

One of the potential advantages of using small transition metal complexes as catalysts over enzymes is that often the transition metal catalysts have a broader substrate scope.^41^ Even though the combination of donor/acceptor carbenes and chiral dirhodium catalysts is uniquely suited for highly enantioselective intermolecular reactions, typically a range of functionality can be accommodated on the donor and acceptor groups.^40^ In order to determine whether a similar trend is observed here, the Rh_2_(*S*-*p*PhTPCP)_4_-catalyzed cyclopropanation of **8b** was examined with a variety of donor/acceptor carbene precursors, and the results are summarized in Table 2. The reaction is particularly effective with *para*-substituted aryldiazoacetates. Both electron-withdrawing and electron-donating groups are accommodated as illustrated in the formation of **17**–**22** with good diastereocontrol (18 → 20:1 d.r.) and enantiocontrol (98–99% ee). A particularly interesting product is triflate **21** because it is well suited for further diversification. Even an unsubstituted phenyl derivative performed well, generating **22** in 91% yield, 20:1 d.r., and 98% ee. The reaction can also be extended to *meta*-substituted aryldiazoacetates, albeit with a slight decrease in diastereoselectivity, although it retained high enantioselectivity. This effect is seen with the *meta*-methyl and *meta*-bromo derivatives **23** and **24** formed in 17:1 and 13:1 d.r., respectively, but still with high enantioselectivity (96–98% ee). The reaction with a 3,5-dibromo derivative was not as diastereoselective, forming **25** with a 6:1 d.r. The reaction can be extended to heteroaryldiazoacetates, forming **26**–**29** with good diastereocontrol and enantiocontrol. The reaction to form the pyridyl derivative **29** is particularly impressive, proceeding in >20:1 d.r and 98% ee. The reaction with the aryldiazoacetate **14a** was also conducted with the *p*-nosyl derivative of **8b**, and the resulting product **30** was formed in 79% yield, >20:1 d.r., and 98% ee. This reaction was run at a 1.0 mmol scale, and the product readily crystallized. X-ray crystallographic analysis of the product was used to determine the absolute configuration and confirm the assigned relative configuration. The absolute stereochemistry of the other spirocyclopropanes is tentatively assigned by analogy. The vinyl group is another common donor group used in donor/acceptor carbenes.^35^ Product **31** was formed with good diastereocontrol (15:1 d.r.), but in this case, there was a considerable drop in the enantioselectivity to 78% ee. The cyclopropanation was also applied to the 5-membered *N*-tosyl-3-methylenepyrrolidine **9b**. The reaction was very effective, generating spirocyclopropane **32** in 85% yield. The enantioselectivity remained very high (98% ee), but the diastereoselectivity (7:1 d.r.) was lower than what had been seen with the six-membered ring homologue **8b**. Then, we turned to 3-methylenetetrahydropyran **33**, an even more challenging substrate as high diastereoselectivity with **33** requires distinguishing between two sterically similar (CH_2_ and O) groups. The oxaspiro product **34** is also of significant interest. Initially, the reaction performed moderately to give **34** in 54% yield with a 5:1 d.r. However, upon addition of 1,1,1,3,3,3-hexafluoro-2-propanol (HFIP) as an additive,^42^ the reaction performed much better with 69% yield, 10:1 d.r., and 98% ee. We hypothesized that the hydrogen-bonding additive HFIP could interact with the oxygen center of **33**, increasing the steric environment around this center and improving the diastereoselectivity.

**Table 2:**
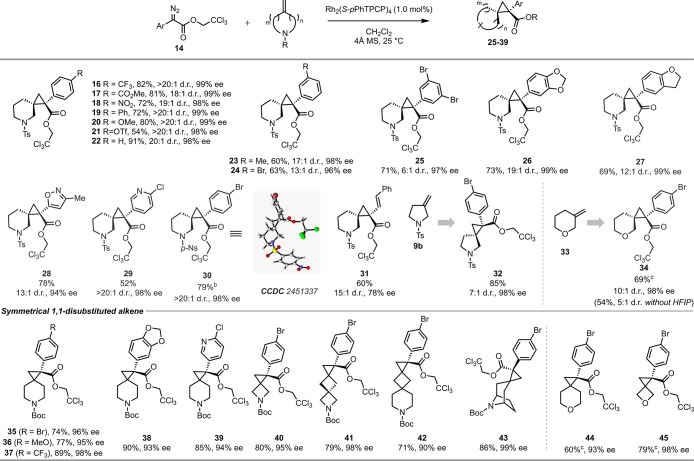
Substrate Scope for Highly Selective Cyclopropanation^a^

a Reaction conditions: **14** (0.2 mmol), alkene (1.5 equiv), CH_2_Cl_2_, 4 Å molecular sieve. Yields are isolated yields. Enantiomeric excess (ee) was determined by HPLC and SFC analysis.

b The reaction was conducted at 1.0 mmol scale.

c 1,1,1,3,3,3-Hexafluoro-2-propanol (HFIP) (5.0 equiv) was added.

We also examined our optimized conditions with symmetrical exomethylene azacycles because of the interest in this class of compounds.^7,11,13–15^ The reaction with 4-methylenpiperidine **7** generated cyclopropane **35** in 74% yield and 96% ee. Rh_2_(*S*-*p*-PhTPCP)_4_-catalyzed cyclopropanation is typically tolerant to a range of aryl functionality on the aryldiazoacetates, and this was confirmed to be the case here. The reaction of **7** with aryldiazoacetates **14b**–**e** all proceeded well to form the spirocyclopropanes **36**–**39** in 77–90% yield with 93–98% ee. Smaller rings such as 3-methylenazetidine **10** were also compatible and generated cyclopropane **40** in 80% yield with 95% ee. The reaction was similarly effective with azaspiro[3.3]­heptane **11** and azaspiro[3.5]­nonane **12**, furnishing the cyclopropanes **41** and **42** in good yield and high asymmetric induction (98 and 90% ee, respectively). The reaction also worked well with the tropane derivative **13**, resulting in the formation of **43** in 86% yield and 99% ee, as a signal diastereomer. The reaction can also be applied to 4-methylenetetrahydropyran and 3-methyleneoxetane to provide the oxaspiro cyclopropane products **44** and **45** in good yield (60% and 79%) and enantioselectivity (93% and 98%). HFIP as an additive was again found to be beneficial, presumably because it would hydrogen bond to the oxygen in the starting material and, thus, avoid potential interference.^42^

The cyclopropanations described so far were conducted with 1 mol % of catalyst loading in order to have definitive values for the stereoselectivity by avoiding possible variability in selectivity when using very low catalyst loadings. However, Rh_2_(*S*-*p*PhTPCP)_4_ has been shown to be effective at very low catalyst loadings in cyclopropanation studies with monosubstituted alkenes.^32^ In order to evaluate whether high turnover numbers were equally feasible here, two representative reactions were conducted at low catalyst loadings (Scheme 1). The Rh_2_(*S*-*p*PhTPCP)_4_-catalyzed reaction between **7** and **14a** was conducted at 0.001% catalyst loading and generated **33** in 83% yield (83,000 TON) and 90% ee, whereas the reaction with **8b** could be achieved with 0.01 mol % to generate **15b** in 82% yield (8,200 TON), 14:1 d.r., and 95% ee.

**Scheme 1:**
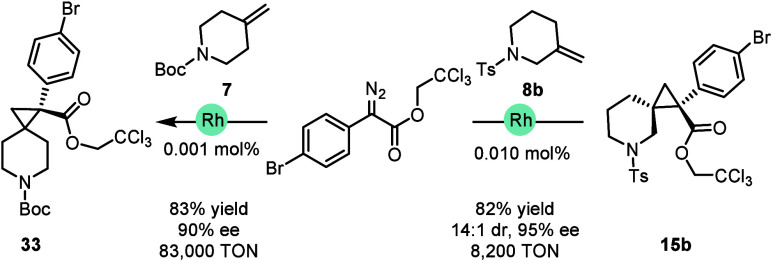
Low Catalyst Loading Cyclopropanation^a^

a Reaction conditions: alkene (1.5 equiv), Rh_2_(*S-p*PhTPCP)_4_, 4 Å molecular sieve at 39 °C.

## Computational Study

One of the intriguing features of the current studies is the observation that only Rh_2_(*S*-*p*PhTPCP)_4_ achieved highly diastereoselective cyclopropanation during the formation of **15b**. This is quite different from the cyclopropanation of monosubstituted alkenes with donor/acceptor carbene in which high diastereoselectivity is routine with dirhodium tetracarboxylates catalysts.^40^ Therefore, we decided to conduct a computational study to rationalize this finding.

The density functional theory (DFT) calculations were conducted using the [(B3LYP-D3BJ) + CPCM­(CH_2_Cl_2_)]/[6-31G­(d,p) + Lanl2dz (for Rh)] level of theory. As previously reported, Rh_2_(*S*-*p*PhTPCP)_4_ has a *C*_2_-symmetric structure, in which all four ligands are pointed up with two tilted ligands to create a (α,α′,α,α′) configuration^43,44^ (Figure 2A). The carbene preferably binds the top face of the catalyst because of steric reasons to generate two diastereomeric metal-carbene intermediates **II** and **III** (Figure 2B).^43^ An interconversion between **II** and **III** does not occur under the reaction time scale because the rotational barrier of the ester group is higher than the barrier for cyclopropanation.^43^ As the metal-carbene diastereomer **II** is more stable than **III**, the further analysis conducted here is focused on the reaction of intermediate **II** with *N*-tosyl-3-methylenepiperidine (**8b**).

**Figure 2:**
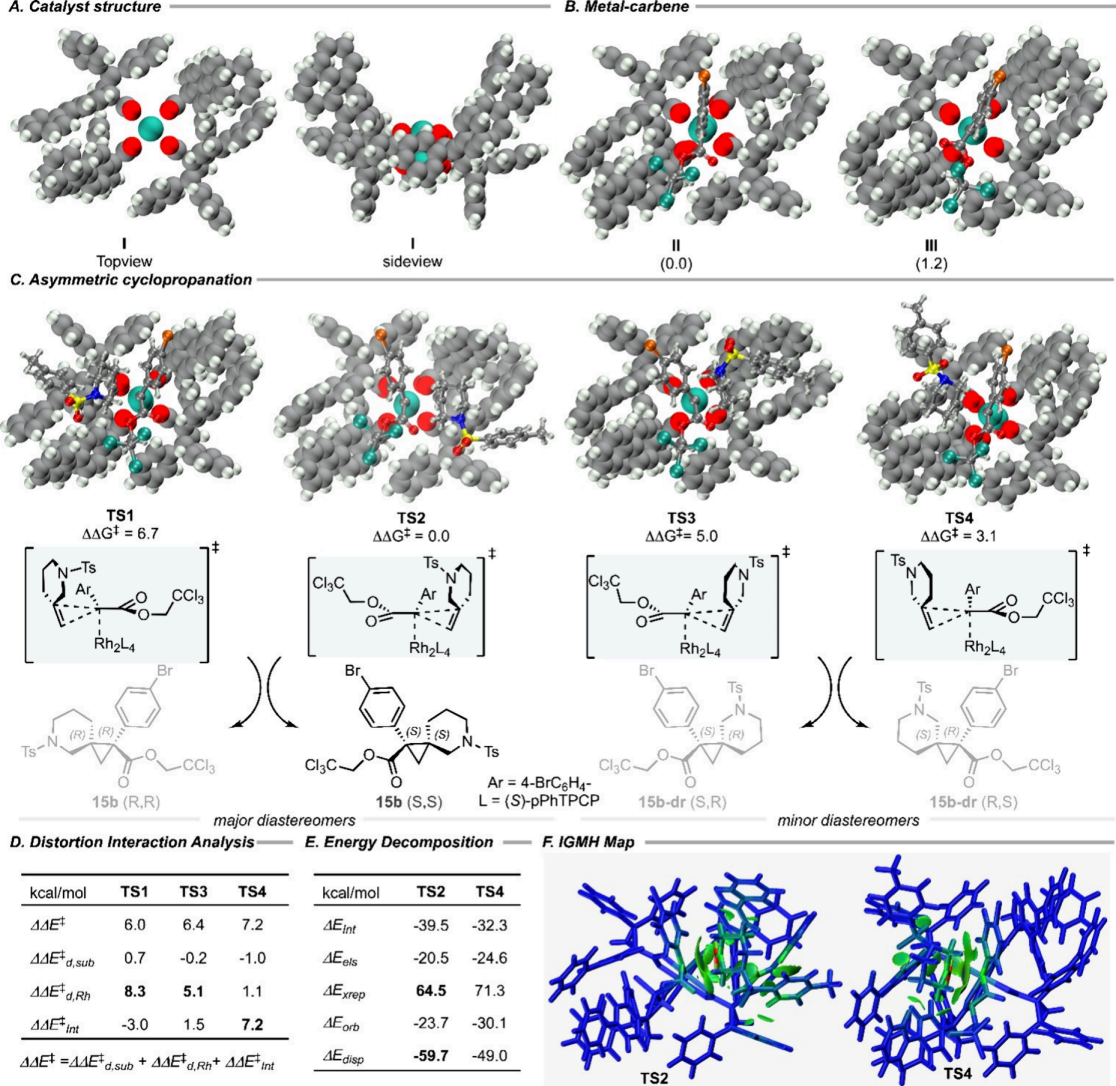
Computational studies. (A) X-ray structure of Rh_2_(*S*-*p*PhTPCP)_4_. (B) DFT-optimized rhodium-carbene intermediate structures. (C) The transition states of cyclopropanation lead to four possible stereoisomers. (D) Distortion-interaction (activation-strain) analysis. (E) Energy decomposition analysis via the sobEDA method provided by the Multiwfn program. (F) Visualization of the noncovalent interaction (NCI) by an independent gradient model based on Hirshfeld partition (IGMH), isovalue = 0.007. The structures were prepared using the VMD program.^51^

First, the enantioselectivity of the reaction was studied by analyzing cyclopropanation transition states **TS1** and **TS2**, resulting in the formation of the major diastereomer (Figure 2C). Transition state **TS2** leading to (*S*,*S*)-**15b** is more energetically favored than **TS1** leading to (*R*,*R*)-**15b** by 6.7 kcal/mol, which is in good agreement with the experimental result of 99% ee. A distortion-interaction analysis^45,46^ was conducted on **TS1** and **TS2**. This analysis decomposed the activation energy into the $\Delta E_{d}^{‡}$ distortion energy of substrates and metal-carbene fragments (metal-carbene $\Delta E_{d,Rh}^{‡}$ and substrate $\Delta E_{d,Sub}^{‡}$ ) and the interaction energy $\Delta E_{int}^{‡}$ between them. As we are interested in the differences between **TS1** and **TS2**, we report all energies relative to those for **TS2** (Figure 2D).^45,46^ The calculations reveal that distortion, particularly metal-carbene distortion, is the most contributing factor in the obtained differences between **TS1** and **TS2**. The identified significant distortion could be attributed to an increasing steric repulsion between the *N*-tosyl group of **8b** and the carbene’s aryl group with the catalyst wall in **TS1** (see the RDG map^47^ in Figure S3 for more details).

The cyclopropanation transition states **TS3** and **TS4** resulting in minor diastereomers (*R*,*S*)-**15b**-**dr** and (*S*,*R*)-**15b**-**dr**, respectively, were also located. These transition states are higher in energy than **TS2** by 5.0 and 3.1 kcal/mol. The distortion-interaction model reveals the distortion of the rhodium-carbene fragment as the most dominant factor for the preference of **TS2** over **TS3**, but the interaction between the rhodium-carbene and substrate fragments is the major factor stabilizing **TS2** over **TS4**. The high distortion in **TS3** is again attributed to the increasing steric repulsion between the *N*-tosyl group of the substrate **8b** with the catalyst wall (see Figure S3 for more details). To gain a deeper understanding of the $\Delta E_{int}^{‡}$ interaction components in **TS2** and **TS4**, the energy decomposition based on dispersion-corrected density functional theory (DFT) (sobEDA)^48,49^ was used.

The sobEDA analysis decomposed $\Delta E_{int}^{‡}$ into electrostatic interaction (Δ*E*_*els*_), Pauli-exchange repulsion (Δ*E*_*xrep*_), orbital interaction (Δ*E*_*orb*_), and dispersion energy (Δ*E*_*dsip*_). The results are shown in Figure 2E. As seen in this figure, Δ*E*_*xrep*_ is significantly higher in **TS4** than **TS2**, which fully reflects the increasing steric repulsion of the carbene’s aryl with the catalyst wall in **TS4** (see Figure S3). The dispersion interaction (i.e., Δ*E*_*disp*_) stabilizes **TS2** compared to **TS4**. The noncovalent interactions contributing to the dispersion energy in **TS2** and **TS4** were also visualized by an independent gradient model based on Hirshfeld partition (IGMH)^50^ (see Figure 2F). The green surface represents the noncovalent interaction between the substrate and catalyst pocket, while the brighter the color of the atoms, the larger is their contribution to the noncovalent interaction. The IGMH map vividly shows there is much more dispersive interaction in **TS2** than in **TS4**. These computational results cleanly demonstrate that the distal control of Rh_2_(*S*-*p*PhTPCP)_4_ in cyclopropanation with **8b** was achieved by catalyst wall–substrate interaction via a combination of steric repulsion and favorable dispersive interactions as the substrate enters the catalyst pocket.

## Conclusion

In conclusion, these studies show that Rh_2_(*S*-*p*PhTPCP)_4_ is an exceptional chiral catalyst for the enantioselective cyclopropanation of **7**–**13** with donor/acceptor carbenes.^51,52^ Not only does the catalyst routinely give high asymmetric induction with a range of substrates, but also it is uniquely suited for high diastereocontrol with substrates that generate two stereogenic centers. Computational studies reveal that the cause of the diastereoselectivity is due to secondary interactions occurring between the catalyst wall and the approaching substrate. The influence of secondary interactions on site selectivity was previously seen in other C_4_ symmetric bowl-shaped catalysts,^39,52^ such as Rh_2_(TPPTTL)_4_, and the recognition it can also apply to Rh_2_(*S*-*p*PhTPCP)_4_ offers interesting opportunities for further application in catalyst-controlled reactions.^53,54^

## Supplementary Material





## Notes

Complete experimental procedures and compound characterization (PDF)
CIF file of compound **30** (CIF)

## Notes

Deposition Number 2451337 contains the supplementary crystallographic data for this paper. These data can be obtained free of charge via the joint Cambridge Crystallographic Data Centre (CCDC) and Fachinformationszentrum Karlsruhe Access Structures service.
