# Supplementary material for: Enantioselective and Diastereoselective Synthesis of Azaspiro[n.2]alkanes by Rhodium-Catalyzed Cyclopropanations
Source: ACS Catal. 2025 Aug 19;15(17):15253–60. doi: 10.1021/acscatal.5c04199 (PMC12418304; doi:10.1021/acscatal.5c04199)
Supplement: Supplementary file 1 [file cs5c04199_si_001.pdf]

Enantioselective and Diastereoselective Synthesis of Azaspiro[n.2]alkanes by Rhodium-Catalyzed Cyclopropanations

Joshua K. Sailer[1,‡], Duc Ly[1,‡], Andrew Wang[1], Djamaladdin G. Musaev[1,2], Huw M. L. Davies[1,*]

[1]Department of Chemistry, Emory University, 1515 Dickey Drive, Atlanta, Georgia 30322, United States

[2]Cherry L. Emerson Center for Scientific Computation, Emory University, Atlanta, Georgia 30322, United States

Corresponding Author: hmdavie@emory.edu

‡These authors contributed equally

Supporting Information

Complete experimental procedures, materials, computational details, and compound characterizations

A. Table of Contents

1. *General Considerations* S2
2. *Known Compounds* S2
3. *Substrate Synthesis* S2
4. *Product Characterization* S5
5. *Low catalyst loading experiments* S27
6. *NMR for Diastereomer Determination* S30
7. *HPLC and SFC Chiral Traces* S39
8. *NMR of Novel Compounds* S72
9. *X-Ray Crystallographic Data* S110
10. *DFT calculations* S121
11. *References* S149

1. General Considerations

All experiments were carried out in flame-dried glassware under argon atmosphere unless otherwise stated. Flash column chromatography was performed on silica gel. Unless otherwise noted, all other reagents were obtained from commercial sources (Sigma Aldrich, Fisher, TCI Chemicals, AK Scientific, Combi Blocks, Oakwood Chemicals, Ambeed) and used as received without purification. ^{1}H, ^{13}C, and ^{19}F NMR spectra were recorded at either 400 MHz (^{13}C at 100 MHz) on Bruker 400 spectrometer or 600 MHz (^{13}C at 151 MHz) on INOVA 600 or Bruker 600 spectrometer. NMR spectra were run in solutions of deuterated chloroform ($CDCl_3$) with residual chloroform taken as an internal standard (7.26 ppm for ^{1}H, and 77.16 ppm for ^{13}C), and were reported in parts per million (ppm). The abbreviations for multiplicity are as follows: s = singlet, d = doublet, t = triplet, q = quartet, p = pentet, m = multiplet, dd = doublet of doublet, etc. Coupling constants (J values) are obtained from the spectra. Thin layer chromatography was performed on aluminum-back silica gel plates with UV light and cerium aluminum molybdate (CAM) stain to visualize. Mass spectra were taken on a Thermo Finnigan LTQ-FTMS spectrometer with APCI or ESI. Enantiomeric excess (% ee) data were obtained on an Agilent 1100 HPLC eluting the purified products using a mixed solution of HPLC-grade 2-propanol (i-PrOH) and n-hexane or a Waters SFC eluting with supercritical CO2 and a 1:1 mixture of HPLC grade methanol:isopropanol with 0.2% formic acid.

2. Known Compounds

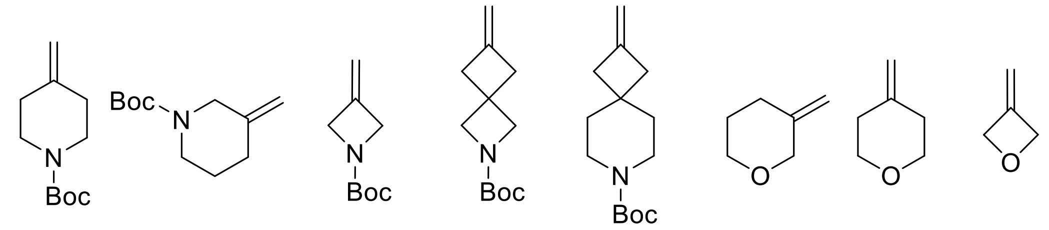

7 **8a** **10** **11** **12** **33** **S2** **S3**

Compound **7** and **10**,[1] **11**,[2] and **12**,[3] **8a**[4], **33**[5], **S2**[5], and **S3**[5] were synthesized according to known methods and spectra matched literature procedure.

3. Substrate Synthesis

3-methylene-1-tosylpiperidine (8b)

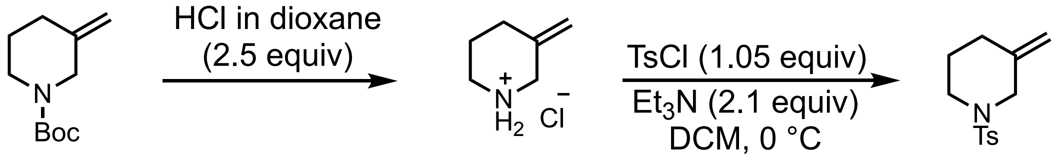

1-Boc-3-methylenepiperidine (5.0 g, 25.3 mmol) and diethyl ether (10 mL) were added into a single-necked flask. Then, hydrogen chloride in dioxane solution (15.8 ml, 63.4 mmol, 4.0 M, 2.5 equiv) was added dropwise, and the reaction was stirred at room temperature for 0.5 h. At this time, the solution was suction filtered, and the filter cake was rinsed with 20 mL of diethyl ether to obtain 3-methylenepiperidine hydrochloride as a white solid, which was directly used in the next reaction without further purification.

To a mixture of 3-methylenepiperidine hydrochloride (802 mg, 6.0 mmol) and Et_3N (1.76 mL, 12.6 mmol, 2.1 equiv) in DCM (20 mL) was added tosyl chloride (1.20 g, 6.3 mmol, 1.05 equiv) at 0 °C. This solution was stirred for 4 h at room temperature. Then, HCl (5 ml, 1 M) and H_2O (10 ml) were added, and the organic layer was separated, washed with brine (10 ml), dried over Na_2SO_4, filtered and concentrated under reduced pressure. The residue was purified by flash column chromatography (SiO_2, 0-30% Et_2O in hexane) to afford 3-methylene-1-tosylpiperidine as a white solid (1.30 g, 86% yield)

^{1}H NMR (400 MHz, $CDCl_3$) δ 7.66 (d, J = 8.0 Hz, 2H), 7.32 (d, J = 8.0 Hz, 2H), 4.90 (s, 1H), 4.82 (s, 1H), 3.50 (s, 2H), 3.08 – 3.04 (m, 2H), 2.43 (s, 2H), 2.10 (t, J = 6.3 Hz, 2H), 1.75 – 1.63 (m, 2H).

^{13}C NMR (101 MHz, $CDCl_3$) δ 143.5, 140.6, 133.2, 129.6, 127.9, 111.8, 52.5, 46.4, 32.0, 25.7, 21.6.

HRMS (+p APCI) calcd for $C_{13}H_{18}O_2N^{32}S$ (M+H) 252.1053, found 252.1054

3-methylene-1-((4-nitrophenyl)sulfonyl)piperidine (S1)

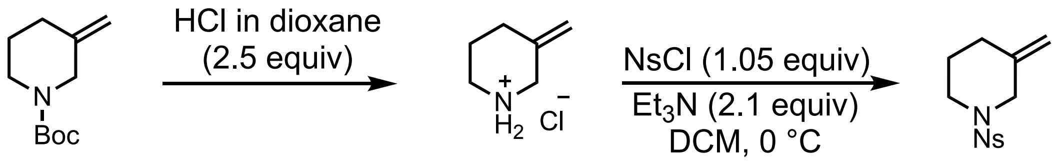

First step analogous to above.

To a mixture of 3-methylenepiperidine hydrochloride (267 mg, 2.0 mmol) and Et_3N (0.73 mL, 5.2 mmol, 2.6 equiv) in DCM (6.7 mL) was added nosyl chloride (465 mg, 2.1 mmol, 1.05 equiv) at 0 °C. This solution was stirred for 4 h at room temperature. Then, HCl (5 ml, 1 M) and H_2O (10 ml) were added, and the organic layer was separated, washed with brine (10

ml), dried over Na_2SO_4, filtered and concentrated under reduced pressure. The residue was purified by flash column chromatography (SiO_2, 0-13% ethyl acetate in hexane) to afford the title compound as an off-white solid (462 mg, 92% yield)

^{1}H NMR (400 MHz, $CDCl_3$) δ 8.37 (d, J = 8.9 Hz, 2H), 7.97 (d, J = 8.8 Hz, 2H), 4.91 (d, J = 1.7 Hz, 1H), 4.84 (d, J = 1.5 Hz, 1H), 3.62 (s, 2H), 3.25 – 3.11 (m, 2H), 2.14 (t, J = 6.3 Hz, 2H), 1.77 – 1.62 (m, 2H).

^{13}C NMR (101 MHz, $CDCl_3$) δ 150.1, 143.0, 139.8, 128.9, 124.3, 112.4, 52.3, 46.3, 31.8, 25.7.

HRMS (+p APCI) calcd for $C_{12}H_{15}O_4N_2{}^{32}S$ (M+H) 283.0747, found 283.0745

2-methylene-1-tosylpyrrolidine (9b)

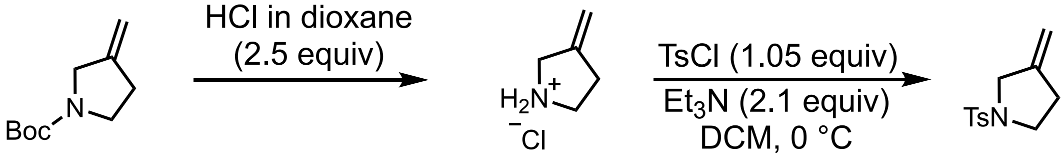

Tert-butyl 3-methylenepyrrolidine-1-carboxylate (2.5 g, 13.6 mmol) and diethyl ether (7.0 mL) were added into a single-necked. Then, hydrogen chloride in dioxane solution (8.53 ml, 34.1 mmol, 4.0 M, 2.5 equiv) was added dropwise, and the solution was stirred at room temperature. After 2.0 hour, the reaction mixture was cooled to 0 °C with an ice bath. Then, 20 ml of DCM and Et_3N (5.89 mL, 42.3 mmol, 2.1 equiv) were added. Finally, tosyl chloride (2.73 g, 14.3 mmol, 1.05 equiv) was added and the mixture was stirred for 4 h at room temperature. HCl (5 ml, 1 M) and H_2O (10 ml) were added and the organic layer was separated, washed with brine (10 ml), dried over Na_2SO_4, filtered and concentrated under reduced pressure. The residue was purified by flash column chromatography (SiO_2, 0-30% Et_2O in hexane) to afford 3-methylene-1-tosylpyrrolidine as a white solid (2.50 g, 77% yield)

^{1}H NMR (400 MHz, $CDCl_3$) δ 7.71 (d, J = 8.3 Hz, 2H), 7.33 (d, J = 8.0 Hz, 2H), 4.91 (dt, J = 7.4, 2.2 Hz, 2H), 3.79 – 3.74 (m, 2H), 3.28 (t, J = 7.1 Hz, 2H), 2.51 – 2.44 (m, 2H), 2.43 (s, 3H).

^{13}C NMR (101 MHz, $CDCl_3$) δ 144.1, 143.7, 132.7, 129.7, 127.9, 107.4, 51.9, 48.1, 31.8, 21.6.

HRMS (+p APCI) calcd for $C_{12}H_{16}O_2N^{32}S$ (M+H) 238.0896, found 238.0898

4. Product Characterization

General Procedure 1: To a flame dried vial equipped with a stir bar and 4Å MS (1000 w%) was added catalyst (1.0 mol%). The reaction was then purged and backfilled three times with nitrogen and capped with an argon balloon. Then, the substrate and 2 mL of DCM was added to the vial and it was set to stir (200 RPM) at 25 °C. At this time, the aryldiazoacetate compound (1.0 equiv) was dissolved in 2 mL of DCM and added to the reaction vial over a period of 1 h via syringe pump. The reaction was left either for an additional 2 h or overnight. At this time, the reaction was stopped, concentrated to dryness, and taken for crude NMR analysis. Following this, the reaction was purified via column chromatography to afford the desired product. Note. The reaction can be conducted routinely at 0.5 mol% with the same level of selectivity

5-(tert-butyl) 1-(2,2,2-trichloroethyl) (1S,3S)-1-(4-bromophenyl)-5-azaspiro[2.5]octane-1,5-dicarboxylate (15a)

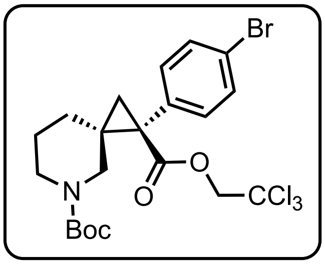

General procedure 1 was used for the cyclopropanation of *tert*-butyl 3-methylenepiperidine-1-carboxylate (29.6 mg, 0.15 mmol, 1.5 equiv) with 2,2,2-trichloroethyl 2-(4-bromophenyl)-2-diazoacetate (37.2 mg, 0.10 mmol, 1.0 equiv) using Rh_2(*S*-*p*PhTPCP)$_4$ (1.7 mg, 1.0 mol%) as catalyst. Purification by column chromatography (0-40% diethyl ether/hexanes) afforded the product as an amorphous white solid 36.7 mg, 86% yield).

$[\alpha]^{20}_D$: +62.4° (c = 4.54 g/100 ml, $CHCl_3$)

^{1}H NMR (400 MHz, $CDCl_3$) δ 7.43 (d, J = 8.5 Hz, 2H), 7.28 (s, 2H), 4.79 (bs, 1H), 4.66 – 4.44 (m, 1H), 3.88 (s, 1H), 3.49 (s, 2H), 2.94 (s, 1H), 1.95 (d, J = 5.0 Hz, 1H), 1.65 – 1.55 (m, 1H), 1.48 (s, 9H), 1.21 (d, J = 5.0 Hz, 1H), 1.09 – 0.75 (m, 1H).

^{13}C NMR (101 MHz, $CDCl_3$) δ 169.4, 169.1, 154.8, 134.6, 133.1, 131.8, 131.1, 128.3, 121.6, 94.8, 79.7, 74.7, 49.8, 48.2, 43.9, 38.9, 33.3, 28.5, 24.6.

HRMS (+p APCI) calcd for $C_{21}H_{25}O_4N^{79}Br^{35}Cl_3$ (M+) 539.0027, found 539.0028.

Chiral SFC: The enantiopurity was determined to be 99% ee by SFC analysis (OJ3, 2.5 mL/min, 3% (50% methanol in isopropanol with 0.2% Formic Acid) in CO2, 1.0 mg/ml), λ=230 nm, RT: Major: 2.51 min., Minor: 4.19 min.)

2,2,2-trichloroethyl (1S,3S)-1-(4-bromophenyl)-5-tosyl-5-azaspiro[2.5]octane-1-carboxylate (15b)

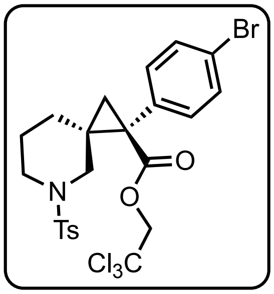

General procedure 1 was used for the cyclopropanation of 3-methylene-1-tosylpiperidine (50.3 mg, 0.20 mmol, 2 equiv) with 2,2,2-trichloroethyl 2-(4-bromophenyl)-2-diazoacetate (37.2 mg, 0.10 mmol, 1.0 equiv) using Rh_2(*S*-pPhTPCP)$_4$ (1.7 mg, 1.0 mol%) as catalyst. Purification by column chromatography (0-25% diethyl ether/hexanes) afforded the product as a white solid (48 mg, 80%).

$[\alpha]^{20}_D$: +31.4° (c = 0.560 g/100 ml, $CHCl_3$)

^{1}H NMR (400 MHz, $CDCl_3$) δ 7.62 (d, J = 8.3 Hz, 2H), 7.44 (d, J = 8.5 Hz, 2H), 7.31 (dd, J = 8.1, 5.4 Hz, 4H), 4.90 (d, J = 12.0 Hz, 1H), 4.65 (d, J = 12.0 Hz, 1H), 3.13 (s, 2H), 3.11 – 2.98 (m, 2H), 2.44 (s, 3zH), 1.95 (d, J = 5.4 Hz, 1H), 1.75 (ddd, J = 14.4, 6.9, 3.3 Hz, 1H), 1.60 (tt, J = 9.4, 5.1 Hz, 1H), 1.30 (d, J = 5.4 Hz, 1H), 1.11 (ddd, J = 12.6, 7.9, 4.2 Hz, 1H), 1.05 – 0.94 (m, 1H).

^{13}C NMR (101 MHz, $CDCl_3$) δ 169.1, 143.6, 134.1, 133.3, 133.0, 131.1, 129.7, 127.7, 121.8, 94.8, 74.9, 50.1, 46.6, 39.1, 32.4, 30.6, 23.8, 23.4, 21.6.

HRMS (+p APCI) calcd for (M+) $C_{23}H_{24}O_4N^{79}Br^{35}Cl_3{}^{32}S$ 593.9670, found 593.9674.

Chiral HPLC: The enantiopurity was determined to be 98% ee by HPLC analysis (AD-H, 1 mL/min, 10% IPA/Hexane, λ=230 nm, RT: Major: 29.1 min., Minor: 24.4 min.)

2,2,2-trichloroethyl (1*S*,3*S*)-5-tosyl-1-(4-(trifluoromethyl)phenyl)-5-azaspiro[2.5]octane-1-carboxylate (16)

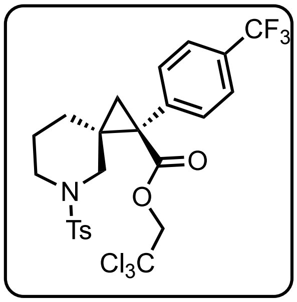

General procedure 1 was used for the cyclopropanation of 3-methylene-1-tosylpiperidine (75.4 mg, 0.30 mmol, 1.5 equiv) with 2,2,2-trichloroethyl 2-diazo-2-(4-(trifluoromethyl)phenyl)acetate (73.2 mg, 0.20 mmol, 1.0 equiv) using Rh_2(*S*-pPhTPCP)$_4$ (3.5 mg, 1.0 mol%) as catalyst. Purification by column chromatography (0-40%% diethyl ether/hexanes) afforded the product as a white solid (93 mg, 83%).

$[\alpha]^{20}_D$: +35.6° (c = 2.57 g/100 ml, $CHCl_3$)

^{1}H NMR (400 MHz, $CDCl_3$) δ 7.60 (d, J = 8.2 Hz, 2H), 7.32 (d, J = 8.1 Hz, 2H), 6.50 (s, 1H), 4.95 (d, J = 12.0 Hz, 1H), 4.87 (d, J = 12.1 Hz, 1H), 3.53 – 3.39 (m, 2H), 2.78 (d, J = 12.5 Hz, 1H), 2.60 – 2.51 (m, 1H), 2.43 (s, 3H), 2.29 (s, 3H), 2.02 (d, J = 5.8 Hz, 1H), 1.86 (d, J = 5.8 Hz, 1H), 1.78 – 1.64 (m, 1H), 1.57 (dt, J = 13.7, 4.2 Hz, 1H), 1.43 – 1.34 (m, 1H), 1.13 (dt, J = 14.4, 4.6 Hz, 1H).

^{13}**C NMR (101 MHz, CDCl$_3$)** δ 168.8, 143.6, 139.1, 133.3, 131.8, 130.1, 129.7, 127.7, 124.9 (q, J = 3.8 Hz), 94.7, 74.9, 50.0, 46.6, 39.3, 32.6, 30.7, 29.7, 23.8, 23.6, 21.6.

^{19}**F NMR (376 MHz, CDCl$_3$)** δ -62.5.

HRMS (+p APCI) calcd for (M+H) $C_{24}H_{24}O_4N^{35}Cl_3F_3{}^{32}S$ 584.0438, found 584.0429

Chiral HPLC: The enantiopurity was determined to be 99.5:0.5 er by HPLC analysis (AD-H, 1 mL/min, 10% IPA/Hexane, λ=230 nm, RT: Major: 19.5 min., Minor: 25.7 min.)

2,2,2-trichloroethyl (1*S*,3*S*)-1-(4-(methoxycarbonyl)phenyl)-5-tosyl-5-azaspiro[2.5]octane-1-carboxylate (17)

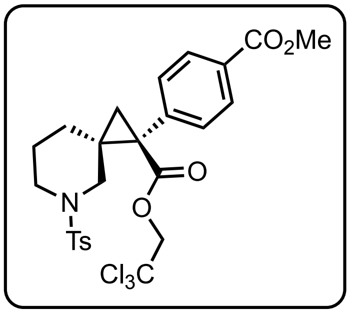

General procedure 1 was used for the cyclopropanation of 3-methylene-1-tosylpiperidine (75.4 mg, 0.30 mmol, 1.5 equiv) with methyl 4-(1-diazo-2-oxo-2-(2,2,2-trichloroethoxy)ethyl)benzoate (70.3 mg, 0.20 mmol, 1.0 equiv) using Rh_2(*S*-pPhTPCP)$_4$ (3.5 mg, 1.0 mol%) as catalyst. Purification by column chromatography (0-30% diethyl ether/hexanes) afforded the product as a white solid (91 mg, 81%).

[α]$^{20}_{D}$: +51.2° (c = 1.25 g/100 ml, CHCl$_3$)

^{1}**H NMR (400 MHz, CDCl$_3$)** δ 8.00 (d, J = 8.2 Hz, 2H), 7.64 (d, J = 8.3 Hz, 2H), 7.53 (d, J = 8.1 Hz, 2H), 7.34 (d, J = 8.0 Hz, 2H), 4.91 (d, J = 11.9 Hz, 1H), 4.68 (d, J = 12.0 Hz, 1H), 3.93 (s, 3H), 3.19 (s, 2H), 3.11 (td, J = 7.3, 3.4 Hz, 1H), 3.08 – 2.99 (m, 1H), 2.46 (s, 3H), 2.01 (d, J = 5.4 Hz, 1H), 1.84 – 1.73 (m, 1H), 1.60 (dtt, J = 15.7, 7.7, 4.2 Hz, 1H), 1.40 (d, J = 5.5 Hz, 1H), 1.12 (ddd, J = 12.5, 7.8, 4.2 Hz, 1H), 0.98 (ddd, J = 13.3, 7.9, 4.2 Hz, 1H).

^{13}**C NMR (101 MHz, CDCl$_3$)** δ 168.9, 166.8, 143.6, 140.2, 133.3, 131.4, 129.7, 129.5, 129.2, 127.7, 94.7, 74.9, 52.2, 50.0, 46.6, 39.5, 32.7, 30.7, 23.8, 23.6, 21.6.

HRMS (+p APCI) calcd for (M+H) $C_{25}H_{27}O_6N^{35}Cl_3{}^{32}S$ 574.0619, found 574.0609.

Chiral HPLC: The enantiopurity was determined to be 99:1 er by HPLC analysis (AD-H, 1 mL/min, 10% IPA/Hexane, λ=230 nm, RT: Major: 41.4 min., Minor: 35.7 min.)

2,2,2-trichloroethyl (1*S*,3*S*)-1-(4-nitrophenyl)-5-tosyl-5-azaspiro[2.5]octane-1-carboxylate (18)

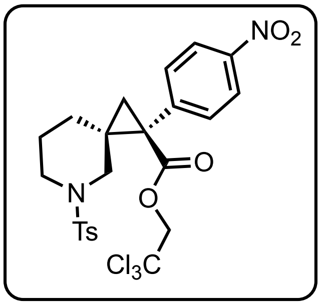

General procedure 1 was used for the cyclopropanation of 3-methylene-1-tosylpiperidine (75.4 mg, 0.30 mmol, 1.5 equiv) with 2,2,2-trichloroethyl 2-diazo-2-(4-nitrophenyl)acetate (67.7 mg, 0.20 mmol, 1.0 equiv) using Rh_2(*S*-*p*-PhTPCP)$_4$ (3.5 mg, 1.0 mol%) as catalyst. Purification by column chromatography (0-40% diethyl ether/hexanes) afforded the product as a white solid (81 mg, 72% yield).

$[\alpha]^{20}_D$: +15.3° (c = 3.27 g/100 ml, $CHCl_3$)

^{1}H NMR (400 MHz, $CDCl_3$) δ 8.18 (d, J = 8.8 Hz, 2H), 7.63 (dd, J = 8.6, 2.1 Hz, 4H), 7.33 (d, J = 8.1 Hz, 2H), 4.89 (d, J = 12.0 Hz, 1H), 4.69 (d, J = 12.0 Hz, 1H), 3.24 (d, J = 12.4 Hz, 1H), 3.15 (d, J = 12.3 Hz, 1H), 3.12 – 3.00 (m, 2H), 2.44 (s, 3H), 2.07 (d, J = 5.5 Hz, 1H), 1.75 (ddt, J = 12.2, 8.0, 4.9 Hz, 1H), 1.66 – 1.58 (m, 1H), 1.41 (d, J = 5.6 Hz, 1H), 1.05 (dt, J = 7.8, 4.9 Hz, 2H).

^{13}C NMR (101 MHz, $CDCl_3$) δ 168.3, 147.3, 143.7, 142.5, 133.3, 132.4, 129.7, 127.6, 123.1, 94.6, 75.0, 49.7, 46.5, 39.3, 33.2, 30.8, 23.8, 23.7, 21.6.

HRMS (+p APCI) calcd for $C_{23}H_{24}O_6N_2{}^{35}Cl_3{}^{32}S$ (M+H) 561.0415, found 561.0407.

Chiral HPLC: The enantiopurity was determined to be 99% ee by HPLC analysis (AD-H, 1 mL/min, 10% IPA/Hexane, λ=230 nm, RT: Major: 26.8 min., Minor: 37.5 min.)

2,2,2-trichloroethyl (1S,3S)-1-([1,1'-biphenyl]-4-yl)-5-tosyl-5-azaspiro[2.5]octane-1-carboxylate (19)

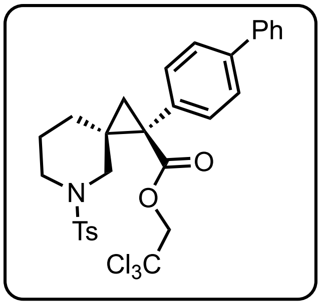

General procedure 1 was used for the cyclopropanation of 3-methylene-1-tosylpiperidine (75.4 mg, 0.30 mmol, 1.5 equiv) with 2,2,2-trichloroethyl 2-([1,1'-biphenyl]-4-yl)-2-diazoacetate (73.9 mg, 0.20 mmol, 1.0 equiv) using Rh_2(*S*-*p*-PhTPCP)$_4$ (3.5 mg, 1.0 mol%) as catalyst. Purification by column chromatography (0-40% diethyl ether/hexanes) afforded the product as a white solid (102.8 mg, 87% yield).

$[\alpha]^{20}_D$: +58.3° (c = 1.6 g/100 ml, $CHCl_3$)

^{1}H NMR (400 MHz, CDCl$_3$) δ 7.64 (d, *J* = 8.3 Hz, 2H), 7.59 (d, *J* = 7.0 Hz, 2H), 7.53 (s, 2H), 7.49 (d, *J* = 8.4 Hz, 2H), 7.46 – 7.40 (m, 2H), 7.38 – 7.30 (m, 3H), 4.93 (d, *J* = 12.0 Hz, 1H), 4.66 (d, *J* = 11.9 Hz, 1H), 3.26 – 3.11 (m, 3H), 2.98 (ddd, *J* = 11.6, 8.2, 3.6 Hz, 1H), 2.45 (s, 3H), 1.97 (d, *J* = 5.4 Hz, 1H), 1.89 – 1.74 (m, 1H), 1.69 – 1.58 (m, 1H), 1.38 (d, *J* = 5.4 Hz, 1H), 1.22 (ddd, *J* = 13.0, 8.6, 4.4 Hz, 1H), 1.00 (ddd, *J* = 13.8, 7.5, 4.1 Hz, 1H).

^{13}C NMR (101 MHz, CDCl$_3$) δ 169.7, 143.6, 140.6, 140.5, 134.1, 133.5, 131.8, 129.8, 128.9, 127.8, 127.6, 127.2, 126.7, 95.0, 74.9, 50.5, 46.8, 39.5, 32.3, 30.8, 24.0, 23.5, 21.7.

HRMS (+p APCI) calcd for $C_{29}H_{29}O_4N^{35}Cl_3{}^{32}S$ (M+H) 592.0877, found 592.0871.

Chiral HPLC: The enantiopurity was determined to be 99% ee by HPLC analysis (AD-H, 1 mL/min, 10% IPA/Hexane, λ=230 nm, RT: Major: 26.8 min., Minor: 37.5 min.)

2,2,2-trichloroethyl (1S,3S)-1-(4-methoxyphenyl)-5-tosyl-5-azaspiro[2.5]octane-1-carboxylate (20)

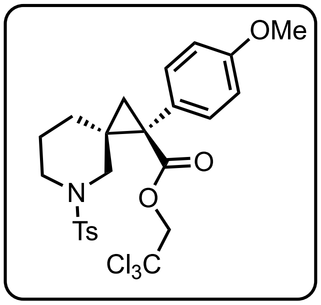

General procedure 1 was used for the cyclopropanation of 3-methylene-1-tosylpiperidine (75.4 mg, 0.30 mmol, 1.5 equiv) with 2,2,2-trichloroethyl 2-diazo-2-(4-methoxyphenyl)acetate (64.7 mg, 0.20 mmol, 1.0 equiv) using Rh$_2$(*S*-*p*-PhTPCP)$_4$ (3.5 mg, 1.0 mol%) as catalyst. Purification by column chromatography (0-40% diethyl ether/hexanes) afforded the product as a white solid (97.8 mg, 89% yield).

$[\alpha]^{20}_D$: +55.9^o (c = 1.42 g/100 ml, CHCl$_3$)

^{1}H NMR (400 MHz, CDCl$_3$) δ 7.62 (d, *J* = 8.3 Hz, 2H), 7.38 – 7.28 (m, 4H), 6.83 (d, *J* = 8.8 Hz, 2H), 4.89 (d, *J* = 12.0 Hz, 1H), 4.64 (d, *J* = 12.0 Hz, 1H), 3.79 (s, 3H), 3.19 – 3.06 (m, 3H), 3.01 – 2.91 (m, 1H), 2.44 (s, 3H), 1.89 (d, *J* = 5.3 Hz, 1H), 1.81 – 1.69 (m, 1H), 1.64 – 1.52 (m, 1H), 1.28 (d, *J* = 5.3 Hz, 1H), 1.16 (ddd, *J* = 13.0, 8.3, 4.1 Hz, 1H), 0.96 (ddd, *J* = 13.7, 7.6, 4.2 Hz, 1H).

^{13}C NMR (101 MHz, CDCl$_3$) δ 169.9, 159.1, 143.6, 133.5, 132.4, 129.8, 127.8, 127.1, 113.4, 95.0, 74.9, 55.4, 50.5, 46.8, 39.0, 32.1, 30.7, 24.0, 23.5, 21.7.

HRMS (+p APCI) calcd for $C_{24}H_{27}O_5N^{35}Cl_3{}^{32}S$ (M+H$^+$) 546.0670, found 546.0665.

Chiral SFC: The enantiopurity was determined to be 99% ee by SFC analysis (OJ3, 2.5 mL/min, 10% (50% methanol in isopropanol with 0.2% Formic Acid) in CO2, 1.0 mg/ml), λ=230 nm, RT: Major: 4.08 min., Minor: 5.21 min.)

2,2,2-trichloroethyl (1*S*,3*S*)-5-tosyl-1-(4-(((trifluoromethyl)sulfonyl)oxy)phenyl)-5-azaspiro[2.5]octane-1-carboxylate (21)

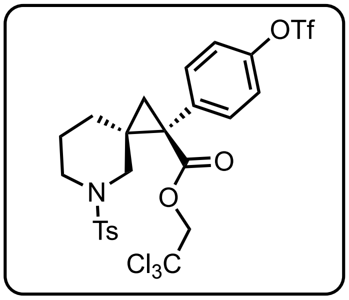

General procedure 1 was used for the cyclopropanation of 3-methylene-1-tosylpiperidine (75.4 mg, 0.30 mmol, 1.5 equiv) with 2,2,2-trichloroethyl 2-diazo-2-(4-(((trifluoromethyl)sulfonyl)oxy)phenyl)acetate (88.3 mg, 0.20 mmol, 1.0 equiv) using Rh_2(*S*-pPhTPCP)$_4$ (3.5 mg, 1.0 mol%) as catalyst. Purification by column chromatography (0-35% diethyl ether/hexanes) afforded the product as a clear oil (71.8 mg, 54%).

$[\alpha]^{20}_D$: +42.1° (c = 1.0 g/100 ml, $CHCl_3$)

^{1}H NMR (600 MHz, $CDCl_3$) δ 7.63 (d, *J* = 8.3 Hz, 2H), 7.52 (d, *J* = 8.8 Hz, 2H), 7.34 – 7.30 (m, 2H), 7.23 (d, *J* = 8.8 Hz, 2H), 4.83 (d, *J* = 11.9 Hz, 1H), 4.69 (d, *J* = 12.0 Hz, 1H), 3.21 – 3.14 (m, 2H), 3.13 – 3.02 (m, 2H), 2.44 (s, 3H), 1.99 (d, *J* = 5.4 Hz, 1H), 1.80 – 1.72 (m, 1H), 1.67 – 1.58 (m, 1H), 1.33 (d, *J* = 5.4 Hz, 1H), 1.13 (ddd, *J* = 13.9, 7.9, 4.2 Hz, 1H), 1.02 (ddd, *J* = 13.8, 8.0, 4.2 Hz, 1H).

^{13}C NMR (151 MHz, $CDCl_3$) δ 168.9, 149.0, 143.7, 135.9, 133.6, 133.3, 129.8, 127.8, 121.0, 118.9 (q, *J* = 320.9 Hz), 94.8, 75.1, 50.0, 46.6, 39.1, 32.8, 30.8, 23.9, 23.8, 21.7.

^{19}F NMR (565 MHz, $CDCl_3$) δ -72.79.

HRMS (+p APCI) calcd for $C_{24}H_{24}O_7N^{35}Cl_3F_3{}^{32}S_2$ (M+H) 664.0006, found 664.0018.

Chiral HPLC: The enantiopurity was determined to be 98% ee by HPLC analysis (AD-H, 1 mL/min, 10% IPA/Hexane, λ=230 nm, RT: Major: 38.2 min., Minor: 33.3 min.)

2,2,2-trichloroethyl (1*S*,3*S*)-1-phenyl-5-tosyl-5-azaspiro[2.5]octane-1-carboxylate (22)

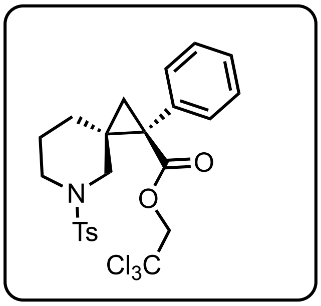

General procedure 1 was used for the cyclopropanation of 3-methylene-1-tosylpiperidine (75.4 mg, 0.30 mmol, 1.5 equiv) with 2,2,2-trichloroethyl 2-diazo-2-phenylacetate (58.7 mg, 0.20 mmol, 1.0 equiv) using Rh_2(*S*-*p*-PhTPCP)$_4$ (3.5 mg, 1.0 mol%) as catalyst. Purification by column chromatography (0-40% diethyl ether/hexanes) afforded the product as a white solid (97.8 mg, 89% yield).

$[\alpha]^{20}_D$: +47.5° (c = 2.73 g/100 ml, $CHCl_3$)

^{1}H NMR (400 MHz, $CDCl_3$) δ 7.63 (d, J = 8.2 Hz, 2H), 7.44 – 7.39 (m, 2H), 7.35 – 7.27 (m, 5H), 4.89 (d, J = 12.0 Hz, 1H), 4.64 (d, J = 12.0 Hz, 1H), 3.25 – 3.06 (m, 3H), 2.94 (ddd, J =

11.6, 8.1, 3.5 Hz, 1H), 2.44 (s, 3H), 1.93 (d, J = 5.3 Hz, 1H), 1.77 (dtt, J = 14.8, 7.5, 3.9 Hz, 1H), 1.67 – 1.52 (m, 1H), 1.33 (d, J = 5.3 Hz, 1H), 1.15 (dd, J = 8.7, 4.4 Hz, 1H), 0.93 (ddd, J = 13.8, 7.4, 4.1 Hz, 1H).

^{13}C NMR (101 MHz, CDCl$_3$) δ 169.6, 143.5, 135.0, 133.4, 131.3, 129.7, 128.0, 127.7, 127.6, 94.9, 74.8, 65.9, 50.4, 46.6, 39.6, 32.1, 30.7, 23.9, 23.4, 21.6, 15.3.

HRMS (+p APCI) calcd for $C_{23}H_{25}O_4N^{35}Cl_3{}^{32}S$ (M+H) 516.0564, found 516.0555.

Chiral HPLC: The enantiopurity was determined to be 99% ee by HPLC analysis (AD-H, 1 mL/min, 10% IPA/Hexane, λ=230 nm, RT: Major: 27.4 min., Minor: 20.1 min.)

2,2,2-trichloroethyl (1S,3S)-1-(m-tolyl)-5-tosyl-5-azaspiro[2.5]octane-1-carboxylate (23)

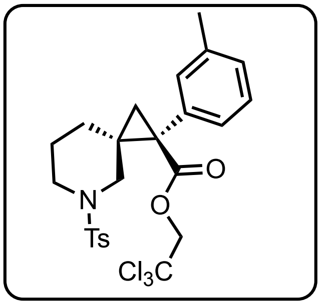

General procedure 1 was used for the cyclopropanation of 3-methylene-1-tosylpiperidine (75.4 mg, 0.30 mmol, 1.5 equiv) with 2,2,2-trichloroethyl 2-diazo-2-(m-tolyl)acetate (61.5 mg, 0.20 mmol, 1.0 equiv) using Rh_2(*S*-*p*-PhTPCP)$_4$ (3.5 mg, 1.0 mol%) as catalyst. Purification by column chromatography (0-20% diethyl ether/hexanes) afforded the product as a white solid (95.6 mg, 90% yield).

[α]$^{20}_{D}$: +40.5° (c = 0.890 g/100 ml, CHCl$_3$)

^{1}H NMR (400 MHz, CDCl$_3$) δ 7.62 (d, *J* = 8.0 Hz, 2H), 7.32 (d, *J* = 8.0 Hz, 2H), 7.26 (s, 1H), 7.19 (d, *J* = 1.4 Hz, 2H), 7.12 – 7.04 (m, 1H), 4.91 (d, *J* = 11.9 Hz, 1H), 4.62 (d, *J* = 11.9 Hz, 1H), 3.25 – 3.02 (m, 3H), 3.00 – 2.85 (m, 1H), 2.44 (s, 3H), 2.32 (s, 3H), 1.91 (d, *J* = 5.3 Hz, 1H), 1.76 (ddt, *J* = 14.4, 7.6, 3.7 Hz, 1H), 1.60 (ddt, *J* = 13.4, 8.8, 4.4 Hz, 1H), 1.33 (d, *J* = 5.3 Hz, 1H), 1.17 (ddd, *J* = 13.3, 8.6, 4.1 Hz, 1H), 0.92 (ddd, *J* = 13.9, 7.4, 4.2 Hz, 1H).

^{13}C NMR (101 MHz, CDCl$_3$) δ 169.7, 143.6, 137.6, 134.9, 133.4, 132.3, 129.8, 128.5, 128.3, 127.9, 127.8, 95.0, 74.8, 50.5, 46.8, 39.7, 32.0, 30.8, 24.0, 23.4, 21.7, 21.5.

HRMS (+p APCI) calcd for $C_{24}H_{27}O_4N^{35}Cl_3{}^{32}S$ (M+H) 530.0721, found 530.0707.

Chiral HPLC: The enantiopurity was determined to be 98% ee by HPLC analysis (AD-H, 1 mL/min, 10% IPA/Hexane, λ=230 nm, RT: Major: 28.98 min., Minor: 13.86 min.)

2,2,2-trichloroethyl (1S,3S)-1-(3-bromophenyl)-5-tosyl-5-azaspiro[2.5]octane-1-carboxylate (24)

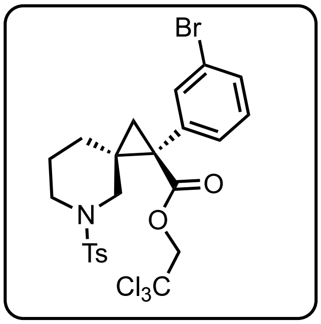

General procedure 1 was used for the cyclopropanation of 3-methylene-1-tosylpiperidine (75.4 mg, 0.30 mmol, 1.5 equiv) with 2,2,2-trichloroethyl 2-(3-bromophenyl)-2-diazoacetate (74.5 mg, 0.20 mmol, 1.0 equiv) using Rh_2(*S*-*p*-PhTPCP)$_4$ (3.5 mg, 1.0 mol%) as catalyst. Purification by column chromatography (0-30% diethyl ether/hexanes) afforded the product as a white solid (108.9 mg, 91% yield).

$[\alpha]^{20}_D$: +41.2° (c = 0.450 g/100 ml, $CHCl_3$)

^{1}H NMR (400 MHz, $CDCl_3$) δ 7.66 – 7.56 (m, 3H), 7.41 (d, *J* = 7.9 Hz, 1H), 7.35 (d, *J* = 8.0 Hz, 1H), 7.32 (d, *J* = 8.0 Hz, 2H), 7.18 (t, *J* = 7.9 Hz, 1H), 4.89 (d, *J* = 11.9 Hz, 1H), 4.65 (d, *J* = 11.9 Hz, 1H), 3.22 – 3.06 (m, 3H), 2.99 (ddd, *J* = 11.4, 7.9, 3.6 Hz, 1H), 2.44 (s, 3H), 1.95 (d, *J* = 5.4 Hz, 1H), 1.83 – 1.70 (m, 1H), 1.66 – 1.56 (m, 1H), 1.33 (d, *J* = 5.5 Hz, 1H), 1.16 (ddd, *J* = 12.9, 8.2, 4.2 Hz, 1H), 0.98 (ddd, *J* = 13.8, 7.7, 4.2 Hz, 1H).

^{13}C NMR (101 MHz, $CDCl_3$) δ 169.0, 143.7, 137.4, 134.6, 133.5, 131.0, 130.0, 129.8, 129.6, 127.8, 122.0, 94.9, 75.0, 50.2, 46.7, 39.4, 32.6, 30.8, 23.9, 23.6, 21.7.

HRMS (+p APCI) calcd for $C_{23}H_{24}O_4N^{79}Br^{35}Cl_3^{32}S$ (M+H$^+$) 593.9670, found 593.9661.

Chiral HPLC: The enantiopurity was determined to be 96% ee by HPLC analysis (AD-H, 1 mL/min, 10% IPA/Hexane, λ=230 nm, RT: Major: 31.7 min., Minor: 19.8 min.)

2,2,2-trichloroethyl (1S,3S)-1-(3,5-dibromophenyl)-5-tosyl-5-azaspiro[2.5]octane-1-carboxylate (25)

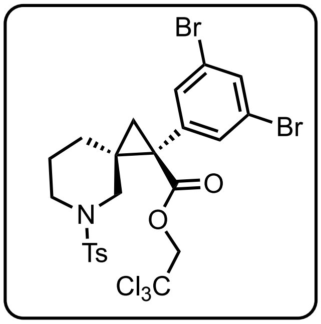

General procedure 1 was used for the cyclopropanation of 3-methylene-1-tosylpiperidine (75.4 mg, 0.30 mmol, 1.5 equiv) with 2,2,2-trichloroethyl 2-diazo-2-(3,5-dibromophenyl)acetate (90.3 mg, 0.20 mmol, 1.0 equiv) using Rh_2(*S*-*p*-PhTPCP)$_4$ (3.5 mg, 1.0 mol%) as catalyst. Purification by column chromatography (0-20% diethyl ether/hexanes) afforded the product as a white solid (94.4 mg, 73% yield, 7:1 dr).

$[\alpha]^{20}_D$: +49.1° (c = 0.750 g/100 ml, $CHCl_3$)

^{1}H NMR (400 MHz, $CDCl_3$) δ 7.66 – 7.57 (m, 3H), 7.52 (d, *J* = 1.8 Hz, 2H), 7.32 (d, *J* = 8.1 Hz, 2H), 4.90 (d, *J* = 11.9 Hz, 1H), 4.66 (d, *J* = 11.9 Hz, 1H), 3.20 – 3.06 (m, 3H), 3.06 – 2.94 (m, 1H), 2.44 (s, 3H), 1.96 (d, *J* = 5.6 Hz, 1H), 1.79 – 1.70 (m, 1H), 1.69 – 1.59 (m, 1H), 1.33

(d, *J* = 5.6 Hz, 1H), 1.16 (ddd, *J* = 12.6, 7.9, 4.3 Hz, 1H), 1.02 (ddd, *J* = 13.4, 7.7, 4.2 Hz, 1H). *For clarity, only the major diastereomer is reported.*

^{13}C NMR (101 MHz, $CDCl_3$) δ 168.4, 143.8, 139.0, 133.6, 133.4, 133.3, 129.8, 127.8, 122.5, 94.8, 75.1, 49.9, 46.7, 39.1, 33.0, 30.9, 23.9, 23.6, 21.7. *For clarity, only the major diastereomer is reported.*

HRMS (+p APCI) calcd for $C_{23}H_{23}O_4N^{79}Br_2{}^{35}Cl_3{}^{32}S$ (M+H) 671.8775, found 671.8776.

Chiral SFC: The enantiopurity was determined to be 97% ee by SFC analysis (SSWhelk, 2.5 mL/min, 10% (50% methanol in isopropanol with 0.2% Formic Acid) in CO2, 1.0 mg/ml), λ=230 nm, RT: Major: 9.98 min., Minor: 10.82 min.)

2,2,2-trichloroethyl (1S,3S)-1-(benzo[d][1,3]dioxol-5-yl)-5-tosyl-5-azaspiro[2.5]octane-1-carboxylate (26)

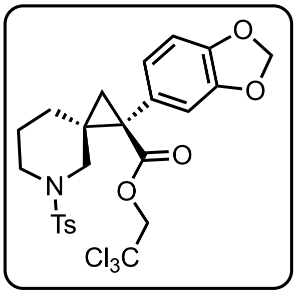

General procedure 1 was used for the cyclopropanation of 3-methylene-1-tosylpiperidine (75.4 mg, 0.30 mmol, 1.5 equiv) with 2,2,2-trichloroethyl 2-(benzo[d][1,3]dioxol-5-yl)-2-diazoacetate (67.5 mg, 0.20 mmol, 1.0 equiv) using Rh_2(*S*-pPhTPCP)$_4$ (3.5 mg, 1.0 mol%) as catalyst. Purification by column chromatography (0-30% diethyl ether/hexanes) afforded the product as white solid (82.1 mg, 73%).

$[\alpha]^{20}_D$: +45.2^o (c = 0.740 g/100 ml, $CHCl_3$)

^{1}H NMR (400 MHz, $CDCl_3$) δ 7.61 (d, J = 8.3 Hz,2H), 7.31 (d, J = 8.0 Hz, 2H), 6.93 (d, J = 1.8 Hz, 1H), 6.84 (dd, J = 8.1, 1.8 Hz, 1H), 6.73 (d, J = 8.0 Hz, 1H), 5.94 (s, 2H), 4.89 (d, J = 11.9 Hz, 1H), 4.65 (d, J = 11.9 Hz, 1H), 3.16 (dd, J = 13.5, 7.2 Hz, 2H), 3.06 (d, J = 12.1 Hz, 1H), 2.92 (ddd, J = 11.7, 8.1, 3.4 Hz, 1H), 2.44 (s, 3H), 1.89 (d, J = 5.3 Hz, 1H), 1.77 (ddq, J = 14.5, 7.4, 3.6 Hz, 1H), 1.68 – 1.54 (m, 2H), 1.26 (d, J = 5.4 Hz, 1H), 1.21 (tt, J = 8.5, 4.2 Hz, 1H), 1.03 – 0.94 (m, 1H).

^{13}C NMR (101 MHz, $CDCl_3$) δ 169.6, 147.2, 147.1, 143.5, 133.3, 129.7, 128.6, 127.7, 124.5, 111.9, 107.7, 101.2, 94.9, 74.8, 50.4, 46.6, 39.4, 32.1, 30.6, 23.9, 23.7, 21.6.

HRMS (+p APCI) calcd for $C_{24}H_{25}O_6N^{35}Cl_3{}^{32}S$ (M+) 560.0463, found 560.0456.

Chiral HPLC: The enantiopurity was determined to be 99.5:0.5 er by HPLC analysis (AD-H, 1 mL/min, 10% IPA/Hexane, λ=230 nm, RT: Major: 49.6 min., Minor: 28.4 min.)

2,2,2-trichloroethyl (1S,3S)-1-(2,3-dihydrobenzofuran-5-yl)-5-tosyl-5 azaspiro[2.5]octane-1-carboxylate (27)

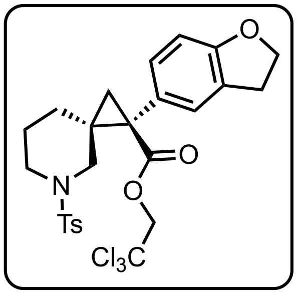

General procedure 1 was used for the cyclopropanation of 3-methylene-1-tosylpiperidine (75.4 mg, 0.30 mmol, 1.5 equiv) with 2 2,2,2-trichloroethyl 2-diazo-2-(2,3-dihydrobenzofuran-6-yl)acetate (67.1 mg, 0.20 mmol, 1.0 equiv) using Rh_2(*S*-pPhTPCP)$_4$ (3.5 mg, 1.0 mol%) as catalyst. Purification by column chromatography (0-30% diethyl ether/hexanes) afforded the product as a white solid (77 mg, 69%).

$[\alpha]^{20}_D$: +53.3° (c = 0.60 g/100 ml, $CHCl_3$)

^{1}H NMR (400 MHz, $CDCl_3$) δ 7.61 (d, J = 8.3 Hz, 2H), 7.31 (d, J = 8.1 Hz, 2H), 7.28 (d, J = 1.9 Hz, 1H), 7.11 (dd, J = 8.4, 2.0 Hz, 1H), 6.69 (d, J = 8.3 Hz, 1H), 4.90 (d, J = 12.0 Hz, 1H), 4.63 (d, J = 12.0 Hz, 1H), 4.56 (t, J = 8.7 Hz, 2H), 3.25 – 3.03 (m, 5H), 2.95 (ddd, J = 11.5, 8.2, 3.5 Hz, 1H), 2.44 (s, 3H), 1.88 (d, J = 5.3 Hz, 1H), 1.76 (ddq, J = 14.5, 7.4, 3.6 Hz, 1H), 1.67 – 1.53 (m, 1H), 1.26 (d, J = 5.3 Hz, 1H), 1.24 – 1.13 (m, 1H), 0.98 (ddd, J = 14.0, 7.6, 4.0 Hz, 1H).

^{13}C NMR (101 MHz, $CDCl_3$) δ 169.9, 159.6, 143.5, 133.3, 130.9, 129.7, 128.0, 127.7, 126.8, 126.7, 108.6, 95.0, 74.8, 71.4, 50.5, 46.7, 39.2, 31.9, 30.6, 29.6, 23.9, 23.4, 21.6.

HRMS (+p APCI) calcd for $C_{25}H_{27}O_5N^{35}Cl_3{}^{32}S$ (M+) 558.0670, found 558.0659.

Chiral HPLC: The enantiopurity was determined to be 99.5:0.5 er by HPLC analysis (AD-H, 1 mL/min, 10% IPA/Hexane, λ=230 nm, RT: Major: 50.3 min., Minor: 21.3 min.)

2,2,2-trichloroethyl (1*R*,3*S*)-1-(3-methylisoxazol-5-yl)-5-tosyl-5-azaspiro[2.5]octane-1-carboxylate (28)

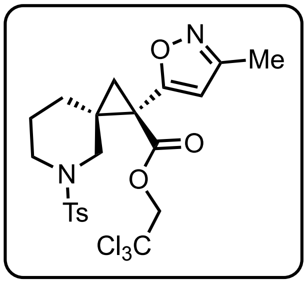

General procedure 1 was used for the cyclopropanation of 3-methylene-1-tosylpiperidine (75.4 mg, 0.30 mmol, 1.5 equiv) with 2,2,2-trichloroethyl 2-diazo-2-(3-methylisoxazol-5-yl)acetate (60 mg, 0.20 mmol, 1.0 equiv) using Rh_2(*S*-pPhTPCP)$_4$ (3.5 mg, 1.0 mol%) as catalyst. Purification by column chromatography (0-35% diethyl ether/hexanes) afforded the product as a white solid (81.4 mg, 78%).

$[\alpha]^{20}_D$: +51.3° (c = 0.99 g/100 ml, $CHCl_3$)

^{1}H NMR (400 MHz, $CDCl_3$) δ 7.60 (d, J = 8.2 Hz, 2H), 7.32 (d, J = 8.1 Hz, 2H), 6.50 (s, 1H), 4.95 (d, J = 12.0 Hz, 1H), 4.87 (d, J = 12.1 Hz, 1H), 3.53 – 3.39 (m, 2H), 2.78 (d, J = 12.5

Hz, 1H), 2.60 – 2.51 (m, 1H), 2.43 (s, 3H), 2.29 (s, 3H), 2.02 (d, J = 5.8 Hz, 1H), 1.86 (d, J = 5.8 Hz, 1H), 1.78 – 1.64 (m, 1H), 1.57 (dt, J = 13.7, 4.2 Hz, 1H), 1.43 – 1.34 (m, 1H), 1.13 (dt, J = 14.4, 4.6 Hz, 1H).

^{13}C NMR (101 MHz, $CDCl_3$) δ 166.7, 165.9, 160.5, 143.7, 133.1, 129.8, 127.6, 106.9, 94.6, 75.4, 49.4, 46.6, 36.0, 32.0, 29.3, 23.6, 23.1, 21.6, 11.6.

HRMS (+p APCI) calcd for $C_{21}H_{24}O_5N_2{}^{35}Cl_3{}^{32}S$ (M+) 521.0466, 521.0460 found.

Chiral HPLC: The enantiopurity was determined to be 97:3 er by HPLC analysis (AD-H, 1 mL/min, 10% IPA/Hexane, λ=230 nm, RT: Major: 39.7 min., Minor: 22.1 min.)

2,2,2-trichloroethyl (1*R*,3*S*)-1-(6-chloropyridin-3-yl)-5-tosyl-5-azaspiro[2.5]octane-1-carboxylate (29)

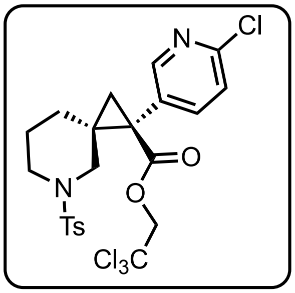

General procedure 1 was used for the cyclopropanation of 3-methylene-1-tosylpiperidine (75.4 mg, 0.30 mmol, 1.5 equiv) with 2,2,2-trichloroethyl 2-(6-chloropyridin-3-yl)-2-diazoacetate (65.8 mg, 0.20 mmol, 1.0 equiv) using Rh_2(*S*-pPhTPCP)$_4$ (3.5 mg, 1.0 mol%) as catalyst. Purification by column chromatography (0-35% diethyl ether/hexanes) afforded the product as a white solid (57.8 mg, 50%).

$[\alpha]^{20}_D$: +57.4° (c = 0.55 g/100 ml, $CHCl_3$)

^{1}H NMR (400 MHz, $CDCl_3$) δ 8.39 (d, J = 2.5 Hz, 1H), 7.82 (dd, J = 8.3, 2.6 Hz, 1H), 7.62 (d, J = 8.2 Hz, 2H), 7.31 (dd, J = 8.3, 6.4 Hz, 3H), 4.89 (d, J = 11.9 Hz, 1H), 4.70 (d, J = 11.9 Hz, 1H), 3.23 (d, J = 12.4 Hz, 1H), 3.17 – 3.07 (m, 2H), 3.01 (td, J = 8.0, 4.1 Hz, 1H), 2.04 (d, J = 5.6 Hz, 1H), 1.72 (tt, J = 6.7, 3.6 Hz, 1H), 1.66 – 1.55 (m, 2H), 1.37 (d, J = 5.6 Hz, 1H), 1.08 (t, J = 6.1 Hz, 2H).

^{13}C NMR (101 MHz, $CDCl_3$) δ 168.3, 151.8, 150.8, 143.7, 142.0, 133.3, 130.1, 129.8, 127.6, 123.6, 94.6, 75.0, 49.6, 46.5, 36.5, 32.8, 30.6, 23.7, 23.2, 21.6.

HRMS (+p APCI) calcd for $C_{22}H_{23}O_4N_2{}^{35}Cl_4{}^{32}S$ (M+H) 551.0127, found 551.0120.

Chiral HPLC: The enantiopurity was determined to be 98% ee by HPLC analysis (AD-H, 1 mL/min, 10% IPA/Hexane, λ=230 nm, RT: Major: 38.2 min., Minor: 33.3 min.)

2,2,2-trichloroethyl (1*S*,3*S*)-1-(4-bromophenyl)-5-((4-nitrophenyl)sulfonyl)-5-azaspiro[2.5]octane-1-carboxylate--ethyne (30)

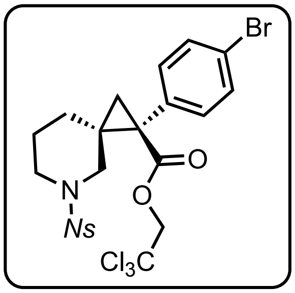

General procedure 1 with some slight modifcations used for the cyclopropanation of 3-methylene-1-((4-nitrophenyl)sulfonyl)piperidine (339 mg, 1.20 mmol, 1.2 equiv) with 2,2,2-trichloroethyl 2-(4-bromophenyl)-2-diazoacetate (372 mg, 1.0 mmol, 1.0 equiv) using Rh_2(*S*-pPhTPCP)$_4$ (17.6 mg, 1.0 mol%) as catalyst. Purification by column chromatography (0-35% diethyl ether/hexanes) afforded the product as a white solid (461 mg, 78%).

$[\alpha]^{20}_D$: +0.66° (c = 1.57 g/100 ml, $CHCl_3$)

^{1}H NMR (400 MHz, $CDCl_3$) δ 8.05 – 7.96 (m, 1H), 7.76 – 7.66 (m, 2H), 7.66 – 7.59 (m, 1H), 7.45 (d, J = 8.6 Hz, 2H), 7.30 (d, J = 8.5 Hz, 2H), 4.79 (d, J = 11.9 Hz, 1H), 4.66 (d, J = 11.9 Hz, 1H), 3.55 – 3.45 (m, 2H), 3.48 – 3.39 (m, 1H), 3.26 (ddd, J = 12.3, 8.1, 3.6 Hz, 1H), 1.95 (d, J = 5.4 Hz, 1H), 1.76 (dtd, J = 14.3, 7.3, 3.7 Hz, 1H), 1.63 (dtt, J = 12.7, 8.4, 4.0 Hz, 1H), 1.32 (d, J = 5.5 Hz, 1H), 1.30 – 1.24 (m, 1H), 1.07 (ddd, J = 13.1, 7.4, 4.1 Hz, 1H).

^{13}C NMR (101 MHz, $CDCl_3$) δ 169.1, 148.2, 134.1, 133.6, 133.0, 132.0, 131.6, 131.2, 131.1, 124.1, 121.9, 94.7, 74.9, 49.4, 46.4, 39.2, 32.6, 31.0, 24.3, 23.8.

HRMS (+p APCI) calcd for $C_{22}H_{21}O_6N_2{}^{79}Br^{35}Cl_3{}^{32}S$ (M+H) 624.9364, found 624.9362.

Chiral SFC: The enantiopurity was determined to be 99% ee by chiral SFC analysis (CEL-1, 2.5 mL/min, 10% methanol in isopropanol with 0.2% Formic Acid in CO2, 1.0 mg/ml, λ=230 nm, RT: Major: 6.13 min., Minor: 6.72 min.)

2,2,2-trichloroethyl (1*S*,3*S*)-1-((*E*)-styryl)-5-tosyl-5-azaspiro[2.5]octane-1-carboxylate (31)

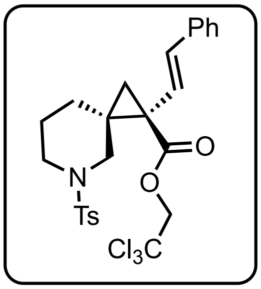

General procedure 1 was used for the cyclopropanation of 3-methylene-1-tosylpiperidine (75.4 mg, 0.30 mmol, 1.5 equiv) with 2,2,2-trichloroethyl (E)-2-diazo-4-phenylbut-3-enoate (63.9 mg, 0.20 mmol, 1.0 equiv) using Rh_2(*S*-pPhTPCP)$_4$ (3.5 mg, 1.0 mol%) as catalyst. Purification by column chromatography (0-40% diethyl ether/hexanes) afforded the product as a white solid (65.3 mg, 60% yield).

$[\alpha]^{20}_D$: -145.3° (c = 0.54 g/100 ml, $CHCl_3$)

^{1}H NMR (400 MHz, $CDCl_3$) δ 7.52 (d, J = 8.3 Hz, 2H), 7.34 – 7.27 (m, 2H), 7.23 (d, J = 7.8 Hz, 3H), 7.20 – 7.15 (m, 2H), 6.71 (d, J = 16.0 Hz, 1H), 6.35 (d, J = 15.9 Hz, 1H), 4.91 (d, J = 12.0 Hz, 1H), 4.74 (d, J = 12.0 Hz, 1H), 3.49 – 3.38 (m, 1H), 3.31 (d, J = 12.3 Hz, 1H), 2.66 (d, J = 12.3 Hz, 1H), 2.51 (ddd, J = 11.3, 8.4, 4.8 Hz, 1H), 2.35 (s, 3H), 1.68 (d, J = 5.6 Hz, 1H), 1.64 – 1.55 (m, 2H), 1.43 – 1.33 (m, 2H), 1.22 (d, J = 5.7 Hz, 1H).

^{13}C NMR (101 MHz, CDCl$_3$) δ 169.6, 143.5, 136.5, 133.5, 133.3, 129.7, 128.7, 127.9, 127.6, 126.4, 123.6, 95.1, 74.9, 49.9, 46.7, 36.4, 33.6, 28.7, 24.6, 21.5, 21.1.

HRMS (+p APCI) calcd for $C_{25}H_{27}O_4N^{35}Cl_3{}^{32}S$ (M+H) 542.0721, 542.0717 found.

Chiral HPLC: The enantiopurity was determined to be 78% ee by HPLC analysis (R,R-Whelk, 1 mL/min, 10% IPA/Hexane, λ=230 nm, RT: Major: 30.3 min., Minor: 25.8 min.)

2,2,2-trichloroethyl (1S,3S)-1-(4-bromophenyl)-5-tosyl-5-azaspiro[2.4]heptane-1-carboxylate (32)

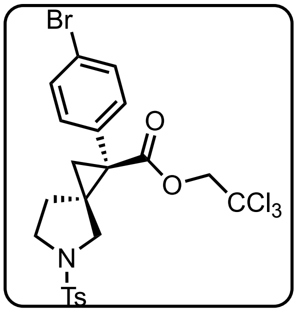

General procedure 1 was used for the cyclopropanation of 3-methylene-1-tosylpyrrolidine (71.2 mg, 0.30 mmol, 1.5 equiv) with 2,2,2-trichloroethyl 2-(4-bromophenyl)-2-diazoacetate (74.5 mg, 0.20 mmol, 1.0 equiv) using Rh_2(*S*-*p*-PhTPCP)$_4$ (3.5 mg, 1.0 mol%) as catalyst. Purification by column chromatography (0-40% diethyl ether/hexanes) afforded the product as a white solid (98.4 mg, 85% yield, 7:1 dr) – a mixture of 7:1 of two diastereomers which are inseparable by flash chromatography.

$[\alpha]^{20}_D$: +35.6^o (c = 2.42 g/100 ml, CHCl$_3$)

^{1}H NMR (400 MHz, CDCl$_3$) δ 7.72 (d, *J* = 8.3 Hz, 2H), 7.43 (d, *J* = 8.4 Hz, 2H), 7.35 (d, *J* = 7.9 Hz, 2H), 7.07 (d, *J* = 8.5 Hz, 2H), 4.82 (d, *J* = 11.9 Hz, 1H), 4.51 (d, *J* = 11.9 Hz, 1H), 3.53 (d, *J* = 11.2 Hz, 1H), 3.45 (d, *J* = 11.2 Hz, 1H), 3.42 – 3.35 (m, 1H), 3.31 – 3.20 (m, 1H), 2.46 (s, 3H), 1.85 (d, *J* = 5.2 Hz, 1H), 1.70 (dt, *J* = 13.1, 8.0 Hz, 1H), 1.43 (d, *J* = 5.2 Hz, 1H), 1.14 (ddd, *J* = 13.1, 7.0, 4.4 Hz, 1H). *For clarity, only the major diastereomer is reported.*

^{13}C NMR (101 MHz, CDCl$_3$) δ 169.8, 143.8, 134.2, 133.3, 132.4, 131.5, 129.8, 127.8, 122.0, 94.5, 74.6, 52.3, 47.5, 37.0, 36.6, 32.7, 26.5, 21.6. *For clarity, only the major diastereomer is reported.*

HRMS (+p APCI) calcd for $C_{22}H_{22}O_4N^{79}Br^{35}Cl_3{}^{32}S$ (M+H) 579.9513, found 579.9511.

Chiral SFC: The enantiopurity was determined to be 98% ee by SFC analysis (OJ3, 2.5 mL/min, 10% (50% methanol in isopropanol with 0.2% Formic Acid) in CO2, 1.0 mg/ml), λ=230 nm, RT: Major: 3.06 min., Minor: 3.62 min.)

2,2,2-trichloroethyl (1S,3S)-1-(4-bromophenyl)-5-oxaspiro[2.5]octane-1-carboxylate (34)

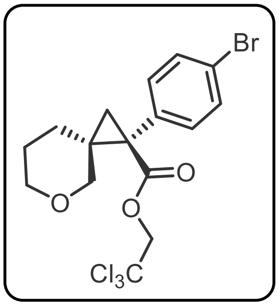

General procedure 1 was used with slight modification for the cyclopropanation 3-methylenetetrahydro-2H-pyran (29.4 mg, 0.30 mmol, 1.5 equiv) with 2,2,2-trichloroethyl 2-(4-bromophenyl)-2-diazoacetate (74.5 mg, 0.20 mmol, 1.0 equiv) using Rh_2(*S*-*p*-PhTPCP)$_4$ (3.5 mg, 1.0 mol%) as catalyst. An additional amount of Hexafluoroisopropanol (HFIP, 105 µL, 168 mg, 1.0 mmol, 5 equiv) was introduced into the catalyst solution to help improve the diastereoselectivity and yield. Purification by column chromatography (0-20% diethyl ether/hexanes) afforded the product as a white solid (60.6 mg, 69% yield, 10:1 dr) – two diastereomers are separable by flash chromatography. *Note*. The reaction gave 54% yield, 5:1 dr if no HFIP was used. The major diastereomer's structure was confirmed by NOSEY experiment. (see below)

$[\alpha]^{20}_D$: +33.0° (c = 1.56 g/100 ml, $CHCl_3$)

^{1}H NMR (600 MHz, $CDCl_3$) δ 7.45 (d, *J* = 8.2 Hz, 2H), 7.28 (s, 1H), 4.80 (d, *J* = 11.9 Hz, 1H), 4.56 (d, *J* = 11.9 Hz, 1H), 3.85 – 3.74 (m, 3H), 3.60 (ddd, *J* = 11.7, 8.4, 3.5 Hz, 1H), 1.96 (s, 1H), 1.63 (ddq, *J* = 17.4, 8.8, 4.7 Hz, 2H), 1.45 (ddd, *J* = 13.8, 9.1, 4.7 Hz, 1H), 1.31 (d, *J* = 5.1 Hz, 1H), 0.98 (dt, *J* = 13.5, 5.1 Hz, 1H).

^{13}C NMR (101 MHz, $CDCl_3$) δ 169.6, 134.7, 133.2, 131.2, 121.8, 94.8, 74.7, 70.9, 68.3, 38.3, 33.4, 31.0, 25.6, 24.2.

HRMS (+p APCI) calcd for $C_{16}H_{16}O_3{}^{79}Br^{35}Cl_3$ (M+H) 439.9343, found 439.9342.

Chiral HPLC: The enantiopurity was determined to be 99.2:0.8 by HPLC analysis (AD-H, 1 mL/min, 2% IPA/Hexane, λ=230 nm, RT: Major: 11.05 min., Minor: 8.61 min.)

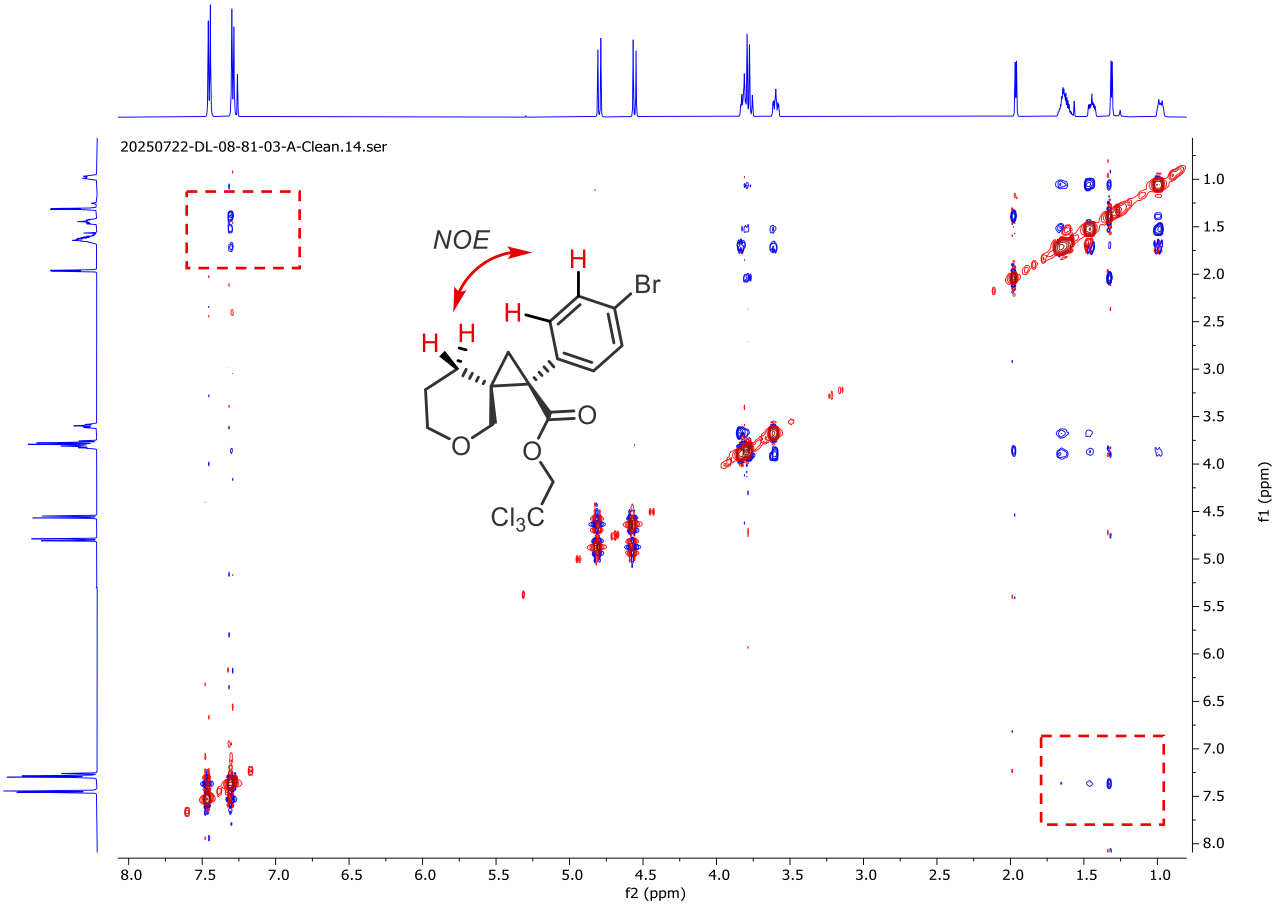

6-(*tert*-butyl) 1-(2,2,2-trichloroethyl) (*R*)-1-(4-bromophenyl)-6-azaspiro[2.5]octane-1,6-dicarboxylate (35)

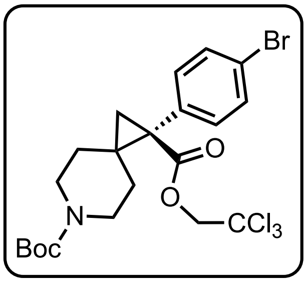

General procedure 1 was used for the cyclopropanation of *tert*-butyl 4-methylenepiperidine-1-carboxylate (40 µL, 0.20 mmol, 2.0 equiv) with 2,2,2-trichloroethyl 2-(4-bromophenyl)-2-diazoacetate (37.2 mg, 0.10 mmol, 1.0 equiv) using Rh_2(*S*-*p*-PhTPCP)$_4$ (1.7 mg, 1.0 mol%) as catalyst. Purification by column chromatography (0-40% diethyl ether/hexanes) afforded the product as a white solid (41.9 mg, 77%).

$[\alpha]^{20}_D$: +27.2° (c = 2.12 g/100 ml, $CHCl_3$)

^{1}H NMR (400 MHz, $CDCl_3$) δ 7.44 (d, J = 8.5 Hz, 1H), 7.27 (d, J = 8.0 Hz, 2H), 4.84 (d, J = 12.0 Hz, 1H), 4.50 (d, J = 11.9 Hz, 1H), 3.98 (s, 1H), 3.86 (s, 1H), 3.03 (t, J = 12.0 Hz, 1H), 2.86 (t, J = 11.9 Hz, 1H), 1.89 – 1.77 (m, 1H), 1.81 – 1.74 (m, 1H), 1.65 (d, J = 13.6 Hz, 1H), 1.45 (s, 9H), 1.29 (d, J = 5.1 Hz, 1H), 0.64 (d, J = 13.5 Hz, 1H).

^{13}C NMR (101 MHz, $CDCl_3$) δ 169.4 154.7, 134.7, 133.2, 131.2, 121.7, 94.7, 79.7, 74.6, 38.9, 33.2, 30.2, 28.5, 23.9, 14.2.

HRMS (+p APCI) calcd for $C_{21}H_{25}O_4N^{79}Br^{35}Cl_3$ (M+) 539.0027, found 539.0029.

Chiral SFC: The enantiopurity was determined to be 98:2 er by SFC analysis (SS-Whelk, 10% MeOH/IPA 0.2% Formic Acid, 2.5 mL/min, λ=230 nm, RT: Major: 2.88 min., Minor: 2.49 min.)

6-(*tert*-butyl) 1-(2,2,2-trichloroethyl) (*R*)-1-(4-methoxyphenyl)-6-azaspiro[2.5]octane-1,6-dicarboxylate (36)

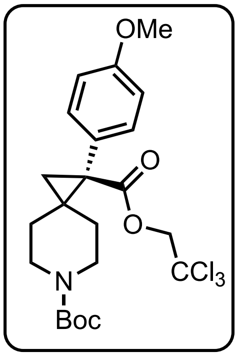

General procedure 1 was used for the cyclopropanation of tert-butyl 4-methylenepiperidine-1-carboxylate (59.2 mg, 0.30 mmol, 1.5 equiv) with 2,2,2-trichloroethyl 2,2,2-trichloroethyl 2-diazo-2-(4-methoxyphenyl)acetate (64.7 mg, 0.20 mmol, 1.0 equiv) using Rh_2(*S*-*p*-PhTPCP)$_4$ (3.5 mg, 1.0 mol%) as catalyst. Purification by column chromatography (0-20% diethyl ether/hexanes) afforded the product as a white amorphous solid (76.4 mg, 77% yield).

$[\alpha]^{20}_{D}$: +43.7° (c = 1.66 *g*/100 ml, $CHCl_3$)

^{1}H NMR (600 MHz, $CDCl_3$) δ 7.31 (d, *J* = 8.6 Hz, 2H), 6.83 (d, *J* = 8.8 Hz, 2H), 4.84 (d, *J* = 12.0 Hz, 1H), 4.50 (d, *J* = 11.9 Hz, 1H), 4.13 – 3.80 (m, 2H), 3.79 (s, 3H), 3.06 (t, *J* = 11.8 Hz, 1H), 2.95 – 2.82 (m, 1H), 1.85 – 1.71 (m, 2H), 1.65 – 1.59 (m, 1H), 1.57 – 1.49 (m, 1H), 1.45 (s, 9H), 1.27 (d, *J* = 4.9 Hz, 1H), 0.70 (d, *J* = 13.6 Hz, 1H).

^{13}C NMR (151 MHz, $CDCl_3$) δ 170.3, 159.0, 154.9, 132.6, 127.8, 113.5, 95.0, 79.7, 74.6, 55.4, 43.4, 38.9, 33.4, 32.9, 30.6, 29.8, 28.6, 23.8.

HRMS (+p APCI) calcd for $C_{22}H_{28}O_5N^{35}Cl_3$ (M+) 491.1028, found 491.1036.

Chiral HPLC: The enantiopurity was determined to be 97.5:2.5 er by HPLC analysis (AD-H, 1 mL/min, 5% IPA/Hexane, λ=230 nm, RT: Major: 8.10 min., Minor: 6.95 min.)

6-(*tert*-butyl) 1-(2,2,2-trichloroethyl) (*R*)-1-(4-(trifluoromethyl)phenyl)-6-azaspiro[2.5]octane-1,6-dicarboxylate (37)

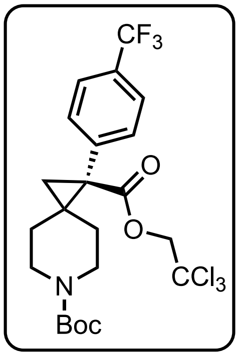

General procedure 1 was used for the cyclopropanation of tert-butyl 4-methylenepiperidine-1-carboxylate (59.2 mg, 0.30 mmol, 1.5 equiv) with 2,2,2-trichloroethyl 2-diazo-2-(4-(trifluoromethyl)phenyl)acetate (72.3 mg, 0.20 mmol, 1.0 equiv) using Rh_2(*S*-*p*-PhTPCP)$_4$ (3.5 mg, 1.0 mol%) as catalyst. Purification by column chromatography (0-20% diethyl ether/hexanes) afforded the product as a white amorphous solid (96.7 mg, 89% yield).

$[\alpha]^{20}_{D}$: +42.0° (c = 1.28 g/100 ml, $CHCl_3$)

^{1}H NMR (400 MHz, CDCl$_3$) δ 7.57 (d, *J* = 8.1 Hz, 2H), 7.52 (d, *J* = 8.0 Hz, 2H), 4.84 (d, *J* = 11.9 Hz, 1H), 4.52 (d, *J* = 12.0 Hz, 1H), 4.18 – 3.71 (m, 2H), 3.03 (t, *J* = 12.2 Hz, 1H), 2.86 (t, *J* = 12.3 Hz, 1H), 1.94 – 1.87 (m, 1H), 1.82 (td, *J* = 12.3, 4.1 Hz, 1H), 1.69 (dd, *J* = 11.2, 6.6 Hz, 1H), 1.55 (q, *J* = 10.6 Hz, 1H), 1.45 (s, 9H), 1.36 (d, *J* = 5.1 Hz, 1H), 0.60 (d, *J* = 13.5 Hz, 1H).

^{13}C NMR (101 MHz, CDCl$_3$) δ 169.3, 154.8, 139.9, 132.0, 129.9 (q, *J* = 32.5 Hz), 126.9, 125.1 (q, *J* = 3.7 Hz), 124.2 (q, *J* = 272.1 Hz), 94.8, 79.8, 74.7, 43.3, 39.3, 33.7, 33.5, 30.3, 28.6, 24.2.

^{19}F NMR (565 MHz, CDCl$_3$) δ -72.79.

HRMS (+p APCI) calcd for $C_{22}H_{25}O_4N^{35}Cl_3F_3$ (M+) 529.0796, found 529.0810.

Chiral HPLC: The enantiopurity was determined to be 99.2:0.8 er by HPLC analysis (AD-H, 1 mL/min, 5% IPA/Hexane, λ=230 nm, RT: Major: 5.26 min., Minor: 6.83 min.)

6-(*tert*-butyl) 1-(2,2,2-trichloroethyl) (*R*)-1-(benzo[*d*][1,3]dioxol-5-yl)-6-azaspiro[2.5]octane-1,6-dicarboxylate (38)

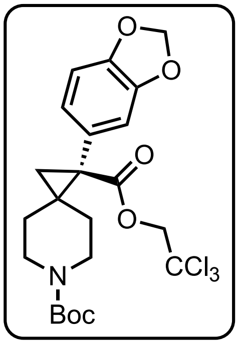

General procedure 1 was used for the cyclopropanation of tert-butyl 4-methylenepiperidine-1-carboxylate (59.2 mg, 0.30 mmol, 1.5 equiv) with 2,2,2-trichloroethyl 2-(benzo[d][1,3]dioxol-5-yl)-2-diazoacetate (67.5 mg, 0.20 mmol, 1.0 equiv) using Rh_2(*S*-*p*-PhTPCP)$_4$ (3.5 mg, 1.0 mol%) as catalyst. Purification by column chromatography (0-20% diethyl ether/hexanes) afforded the product as a white amorphous solid (91.0 mg, 90% yield).

$[\alpha]^{20}_D$: +39.1° (c = 0.280 g/100 ml, CHCl$_3$)

^{1}H NMR (400 MHz, CDCl$_3$) δ 6.91 (s, 1H), 6.83 (dd, *J* = 8.1, 1.8 Hz, 1H), 6.73 (d, *J* = 8.0 Hz, 1H), 5.95 (s, 2H), 4.83 (d, *J* = 12.0 Hz, 1H), 4.53 (d, *J* = 11.9 Hz, 1H), 4.09 – 3.74 (m, 2H), 3.03 (t, *J* = 11.8 Hz, 1H), 2.88 (t, *J* = 12.0 Hz, 1H), 1.87 – 1.73 (m, 2H), 1.65 – 1.59 (m, 1H), 1.58 – 1.53 (m, 1H), 1.45 (s, 9H), 1.25 (d, *J* = 4.8 Hz, 1H), 0.73 (d, *J* = 13.5 Hz, 1H).

^{13}C NMR (101 MHz, CDCl$_3$) δ 170.1, 154.9, 147.4, 147.1, 129.4, 124.7, 112.1, 107.8, 101.2, 94.9, 79.7, 74.7, 43.3, 39.3, 33.4, 33.0, 30.6, 28.6, 24.1.

HRMS (+p APCI) calcd for $C_{22}H_{26}O_6N^{35}Cl_3$ (M+) 505.0820, found 505.0829.

Chiral HPLC: The enantiopurity was determined to be 96.4:3.6 er by HPLC analysis (AD-H, 1 mL/min, 5% IPA/Hexane, λ=230 nm, RT: Major: 10.75 min., Minor: 7.78 min.)

6-(*tert*-butyl) 1-(2,2,2-trichloroethyl) (*R*)-1-(6-chloropyridin-3-yl)-6-azaspiro[2.5]octane-1,6-dicarboxylate (39)

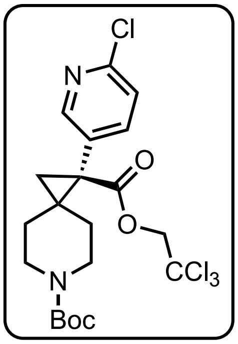

General procedure 1 was used for the cyclopropanation of tert-butyl 4-methylenepiperidine-1-carboxylate (59.2 mg, 0.30 mmol, 1.5 equiv) with 2,2,2-trichloroethyl 2-(6-chloropyridin-3-yl)-2-diazoacetate (65.8 mg, 0.20 mmol, 1.0 equiv) using Rh_2(*S*-*p*-PhTPCP)$_4$ (3.5 mg, 1.0 mol%) as catalyst. Purification by column chromatography (0-30% diethyl ether/hexanes) afforded the product as a clear oil (84.5 mg, 85% yield).

$[\alpha]^{20}_D$: +47.2° (c = 1.58 g/100 ml, $CHCl_3$)

^{1}H NMR (400 MHz, $CDCl_3$) δ 8.37 (d, *J* = 2.6 Hz, 1H), 7.70 (dd, *J* = 8.2, 2.5 Hz, 1H), 7.29 (d, *J* = 8.2 Hz, 1H), 4.83 (d, *J* = 11.9 Hz, 1H), 4.53 (d, *J* = 11.9 Hz, 1H), 4.13 – 3.82 (m, 2H), 3.01 (t, *J* = 11.9 Hz, 1H), 2.86 (t, *J* = 12.1 Hz, 1H), 1.93 (d, *J* = 5.2 Hz, 1H), 1.80 (td, *J* = 12.3, 4.1 Hz, 1H), 1.73 – 1.66 (m, 1H), 1.61 – 1.49 (m, 1H), 1.44 (s, 9H), 1.35 (d, *J* = 5.2 Hz, 1H), 0.62 (d, *J* = 13.3 Hz, 1H).

^{13}C NMR (101 MHz, $CDCl_3$) δ 168.7, 154.7, 152.1, 150.8, 142.0, 130.9, 123.7, 94.6, 79.9, 74.8, 43.1, 36.3, 33.7, 33.6, 30.0, 28.5, 24.1.

HRMS (+p APCI) calcd for $C_{20}H_{24}O_4N_2{}^{35}Cl_4$ (M+) 496.0485, found 496.0494.

Chiral HPLC: The enantiopurity was determined to be 96.9:3.1 er by HPLC analysis (AD-H, 1 mL/min, 5% IPA/Hexane, λ=230 nm, RT: Major: 12.09 min., Minor: 9.87 min.)

5-(*tert*-butyl) 1-(2,2,2-trichloroethyl) (*S*)-1-(4-bromophenyl)-5-azaspiro[2.3]hexane-1,5-dicarboxylate (40)

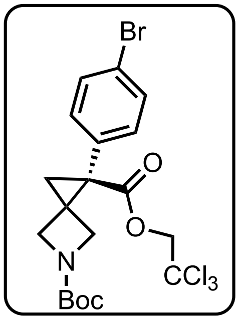

General procedure 1 was used for the cyclopropanation of *tert*-butyl 3-methyleneazetidine-1-carboxylate (34 µL, 0.20 mmol, 2 equiv) with 2,2,2-trichloroethyl 2-(4-bromophenyl)-2-diazoacetate (37.1 mg, 0.10 mmol, 1.0 equiv) using Rh_2(*S*-*p*PhTPCP)$_4$ (1.7 mg, 1.0 mol%) as catalyst. Purification by column chromatography (0-40% diethyl ether/hexanes) afforded the product as a white solid (39.5 mg, 77%).

$[\alpha]^{20}_D$: +23.7° (c = 1.60 g/100 ml, $CHCl_3$)

^{1}H NMR (400 MHz, $CDCl_3$) δ 7.50 (d, J = 8.4 Hz, 2H), 7.24 – 7.18 (m, 2H), 4.89 (d, J = 11.9 Hz, 1H), 4.50 (d, J = 11.9 Hz, 1H), 4.23 – 4.13 (m, 2H), 3.73 (d, J = 9.2 Hz, 1H), 3.62 (d, J = 9.2 Hz, 1H), 2.05 (d, J = 5.6 Hz, 1H), 1.67 (d, J = 5.6 Hz, 1H), 1.43 (s, 9H).

^{13}C NMR (101 MHz, CDCl$_3$) δ 169.8, 156.0, 133.0, 132.4, 131.7, 128.3, 122.2, 94.5, 80.0, 74.5, 35.1, 31.0, 28.4, 24.3.

HRMS (+p APCI) calcd for $C_{19}H_{21}O_4N^{79}Br^{35}Cl_3$ (M+) 510.9714, found 510.9725.

Chiral SFC: The enantiopurity was determined to be 97:3 er by SFC analysis (SS-Whelk, 10% MeOH/IPA 0.2% Formic Acid, 2.5 mL/min, λ=230 nm, RT: Major: 3.53 min., Minor: 2.29 min.)

7-(*tert*-butyl) 1-(2,2,2-trichloroethyl) (*R*)-1-(4-bromophenyl)-7-azadispiro[2.1.3^5.1^3]nonane-1,7-dicarboxylate (41)

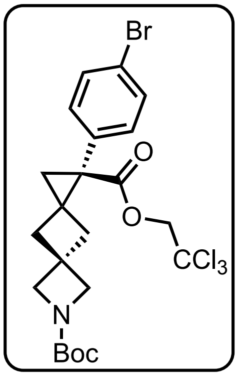

General procedure 1 was used for the cyclopropanation of *tert*-butyl 6-methylene-2-azaspiro[3.3]heptane-2-carboxylate (52.3 mg, 0.25 mmol, 2 equiv) with 2,2,2-trichloroethyl 2-(4-bromophenyl)-2-diazoacetate (37.2 mg, 0.10 mmol, 1.0 equiv) using Rh_2(*S*-*p*-PhTPCP)$_4$ (3.5 mg, 1.0 mol%) as catalyst. Purification by column chromatography (0-40% diethyl ether/hexanes) afforded the product as a white solid (44 mg, 79%).

[α]$^{20}_D$: +24.5° (c = 1.82 g/100 ml, CHCl$_3$)

^{1}H NMR (400 MHz, CDCl$_3$) δ 7.47 (d, J = 8.4 Hz, 2H), 7.15 (d, J = 8.5 Hz, 2H), 4.88 (d, J = 12.0 Hz, 1H), 4.49 (d, J = 12.0 Hz, 1H), 4.03 (d, J = 8.7 Hz, 1H), 3.98 – 3.87 (m, 3H), 2.65 – 2.43 (m, 2H), 2.18 (d, J = 12.8 Hz, 1H), 1.96 (d, J = 5.1 Hz, 1H), 1.81 (d, J = 13.3 Hz, 1H), 1.53 (d, J = 5.1 Hz, 1H), 1.42 (s, 9H).

^{13}C NMR (101 MHz, CDCl$_3$) δ 170.1, 156.2, 134.3, 132.5, 131.5, 121.6, 94.8, 79.5, 74.3, 40.6, 39.3, 35.8, 32.4, 32.1, 28.4, 26.4.

HRMS (+p APCI) calcd for $C_{22}H_{25}O_4N^{79}Br^{35}Cl_3$ (M+) 551.0027, found. 551.0032.

Chiral HPLC: The enantiopurity was determined to be 99:1 er by HPLC analysis (AD-H, 1 mL/min, 2% IPA/Hexane, λ=230 nm, RT: Major: 29.7 min., Minor: 26.4 min.)

8-(*tert*-butyl) 1-(2,2,2-trichloroethyl) (*R*)-1-(4-bromophenyl)-8-azadispiro[2.1.5^5.1^3]undecane-1,8-dicarboxylate (42)

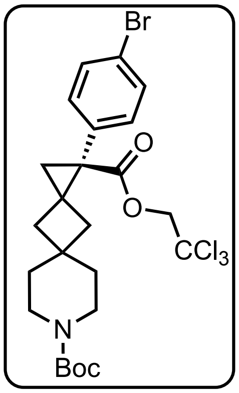

General procedure 1 was used for the cyclopropanation of *tert*-butyl 2-methylene-7-azaspiro[3.5]nonane-7-carboxylate (71.2 mg, 0.30 mmol, 1.5 equiv) with 2,2,2-trichloroethyl 2-(4-bromophenyl)-2-diazoacetate (74.5 mg, 0.20 mmol, 1.0 equiv) using Rh_2(*S*-*p*-PhTPCP)$_4$ (3.5 mg, 1.0 mol%) as catalyst. Purification by column chromatography (0-40% diethyl ether/hexanes) afforded the product as a clear oil (82.9 mg, 71% yield).

$[\alpha]^{20}_D$: +18.3° (c = 1.81 g/100 ml, $CHCl_3$)

^{1}H NMR (400 MHz, CDCl$_3$) δ 7.47 (d, *J* = 8.4 Hz, 2H), 7.19 (d, *J* = 8.5 Hz, 2H), 4.93 (d, *J* = 11.9 Hz, 1H), 4.47 (d, *J* = 12.0 Hz, 1H), 3.43 – 3.16 (m, 4H), 2.25 – 2.08 (m, 2H), 1.98 (d, *J* = 4.9 Hz, 1H), 1.77 (d, *J* = 12.3 Hz, 1H), 1.68 – 1.52 (m, 5H), 1.44 (s, 9H).

^{13}C NMR (101 MHz, CDCl$_3$) δ 170.4, 155.0, 134.8, 132.8, 131.5, 121.6, 95.0, 79.5, 74.4, 39.8, 38.6, 35.9, 33.1, 32.5, 28.6, 27.7.

HRMS (+p APCI) calcd for $C_{24}H_{29}O_4N^{79}Br^{35}Cl_3$ (M+) 579.0340, found 579.0356.

Chiral SFC: The enantiopurity was determined to be 90% ee by SFC analysis (SSWhelk, 2.5 mL/min, 10% (50% methanol in isopropanol with 0.2% Formic Acid) in CO2, 1.0 mg/ml), λ=230 nm, RT: Major: 4.48 min., Minor: 3.52 min.)

8-(tert-butyl) 2'-(2,2,2-trichloroethyl) (1R,2'R,3s,5S)-2'-(4-bromophenyl)-8-azaspiro[bicyclo[3.2.1]octane-3,1'-cyclopropane]-2',8-dicarboxylate (43)

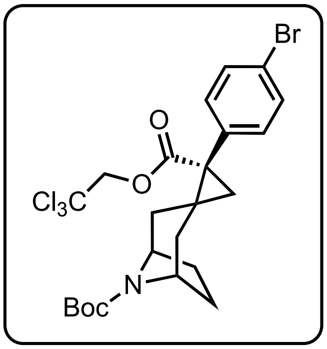

General procedure 1 was used for the cyclopropanation of tert-butyl (1R,5S)-3-methylene-8-azabicyclo[3.2.1]octane-8-carboxylate (67.0 mg, 0.30 mmol, 1.5 equiv) with 2,2,2-trichloroethyl 2-(4-bromophenyl)-2-diazoacetate (74.5 mg, 0.20 mmol, 1.0 equiv) using Rh_2(*S*-pPhTPCP)$_4$ (3.5 mg, 1.0 mol%) as catalyst. Purification by column chromatography (0-20% diethyl ether/hexanes) afforded the product as a single diastereomer white amorphous solid (97.7 mg, 86% yield). To determine the diastereoselectivity, the clean sample was treated with TFA in DCM, to obtain a TFA-salt. The NMR showed that the sample was a single diastereomer.

$[\alpha]^{20}_D$: +65.6° (c = 0.23 g/100 ml, $CHCl_3$)

^{1}H NMR (400 MHz, CDCl$_3$) δ 7.43 (bd, J = 7.9 Hz, 2H), 7.22 (bd, J = 8.1 Hz, 2H), 4.80 (m), 4.62 – 4.01 (m, 3H), 2.17 – 1.84 (m, 6H), 1.76 (d, J = 10.8 Hz, 1H), 1.52 (d, J = 5.1 Hz, 2H), 1.47 (s, 9H), 0.31 (d, J = 13.6 Hz, 1H). A mixture of rotamers was observed.

^{13}C NMR (101 MHz, CDCl$_3$) δ 169.3, 153.5, 134.6, 133.2, 131.2, 121.8, 94.8, 79.6, 74.7, 54.2, 39.4, 36.7, 34.8, 29.1, 28.7, 28.1.

HRMS (+p APCI) calcd for $C_{23}H_{28}O_4N^{79}Br^{35}Cl_3$ (M+H) 566.0262, found 566.0272.

Chiral HPLC: The enantiopurity was determined to be 99.0:1.0 er by HPLC analysis (AD-H, 1 mL/min, 5% IPA/Hexane, λ=230 nm, RT: Major: 8.20 min., Minor: 6.46 min.)

2,2,2-trichloroethyl (R)-1-(4-bromophenyl)-6-oxaspiro[2.5]octane-1-carboxylate (44)

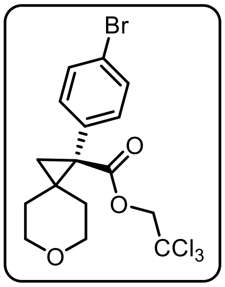

General procedure 1 was used with slight modification for the cyclopropanation of 4-methylenetetrahydro-2H-pyran (29.4 mg, 0.30 mmol, 1.5 equiv) with 2,2,2-trichloroethyl 2-(4-bromophenyl)-2-diazoacetate (74.5 mg, 0.20 mmol, 1.0 equiv) using Rh_2(*S*-*p*-PhTPCP)$_4$ (3.5 mg, 1.0 mol%) as catalyst. An additional amount of Hexafluoroisopropanol (HFIP, 105 µL, 168 mg, 1.0 mmol, 5 equiv) was introduced into the catalyst solution to help improve the yield. Purification by column chromatography (0-20% diethyl ether/hexanes) afforded the product as a clear oil (53.2 mg, 60% yield).

$[\alpha]^{20}_D$: +35.5° (c = 4.03 g/100 ml, CHCl$_3$)

^{1}H NMR (400 MHz, CDCl$_3$) δ 7.45 (d, *J* = 8.5 Hz, 2H), 7.28 (d, *J* = 8.4 Hz, 2H), 4.85 (d, *J* = 11.9 Hz, 1H), 4.50 (d, *J* = 11.9 Hz, 1H), 3.98 (dt, *J* = 11.4, 3.8 Hz, 1H), 3.85 (dt, *J* = 11.3, 3.9 Hz, 1H), 3.59 (td, *J* = 11.1, 2.7 Hz, 1H), 3.44 (td, *J* = 11.0, 2.7 Hz, 1H), 1.94 (ddd, *J* = 14.3, 10.9, 4.2 Hz, 1H), 1.86 (dd, *J* = 5.0, 1.3 Hz, 1H), 1.76 – 1.61 (m, 2H), 1.32 (dd, *J* = 5.0, 1.1 Hz, 1H), 0.57 (dd, *J* = 13.7, 2.8 Hz, 1H).

^{13}C NMR (101 MHz, CDCl$_3$) δ 169.5, 134.8, 133.3, 131.3, 121.8, 94.8, 74.7, 67.6, 67.1, 38.8, 34.5, 32.7, 31.3, 24.6.

HRMS (+p APCI) calcd for $C_{16}H_{16}O_3{}^{79}Br^{35}Cl_3$ (M+) 439.9343, found 439.9342.

Chiral HPLC: The enantiopurity was determined to be 96.3:3.7 er by HPLC analysis (AD-H, 1 mL/min, 1% IPA/Hexane, λ=230 nm, RT: Major: 10.2 min., Minor: 11.1 min.)

2,2,2-trichloroethyl (S)-1-(4-bromophenyl)-5-oxaspiro[2.3]hexane-1-carboxylate (45)

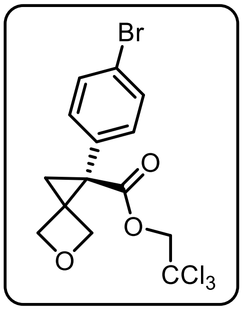

General procedure 1 was used with slight modification for the cyclopropanation of 3-methyleneoxetane (21.0 mg, 0.30 mmol, 1.5 equiv) with 2,2,2-trichloroethyl 2-(4-bromophenyl)-2-diazoacetate (74.5 mg, 0.20 mmol, 1.0 equiv) using Rh_2(*S*-*p*-PhTPCP)$_4$ (3.5 mg, 1.0 mol%) as catalyst. An additional amount of Hexafluoroisopropanol (HFIP, 105 μL, 168 mg, 1.0 mmol, 5 equiv) was introduced into the catalyst solution to help improve the yield. Purification by column chromatography (0-20% diethyl ether/hexanes) afforded the product as a clear oil (70.0 mg, 79% yield).

$[\alpha]^{20}_D$: -8.52° (c = 1.49 g/100 ml, $CHCl_3$)

^{1}H NMR (600 MHz, $CDCl_3$) δ 7.51 (d, *J* = 8.4 Hz, 2H), 7.27 (d, *J* = 8.2 Hz, 2H), 4.97 (d, *J* = 7.1 Hz, 1H), 4.93 (d, *J* = 7.1 Hz, 1H), 4.89 (d, *J* = 11.9 Hz, 1H), 4.53 (d, *J* = 12.0 Hz, 1H), 4.50 (d, *J* = 6.7 Hz, 1H), 4.41 (d, *J* = 6.7 Hz, 1H), 2.06 (d, *J* = 5.6 Hz, 1H), 1.69 (d, *J* = 5.6 Hz, 1H).

^{13}C NMR (151 MHz, $CDCl_3$) δ 169.8, 132.9, 132.5, 131.8, 128.4, 122.3, 94.7, 75.3, 74.5, 35.3, 35.2, 23.9.

HRMS (+p APCI) calcd for $C_{14}H_{13}O_3{}^{79}Br^{35}Cl_3$ (M+) 412.9108, found 412.9108.

Chiral HPLC: The enantiopurity was determined to be 98.9:1.1 er by HPLC analysis (AD-H, 1 mL/min, 1% IPA/Hexane, λ=230 nm, RT: Major: 22.8 min., Minor: 34.5 min.)

5. Low catalyst loading experiments

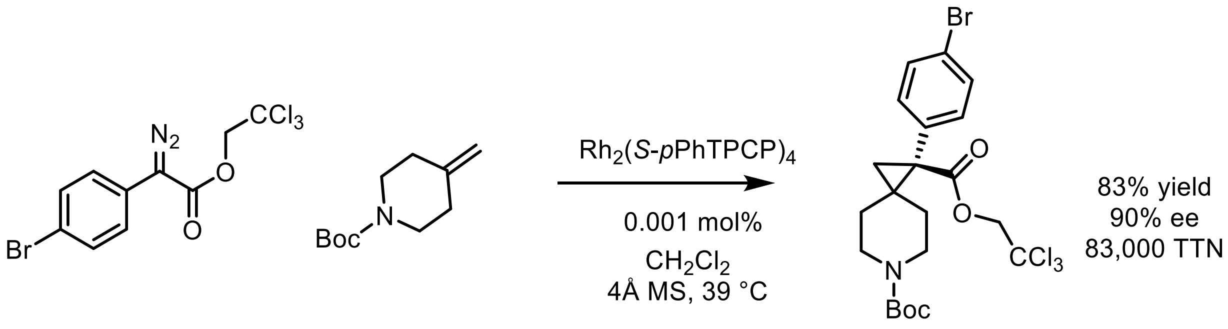

General procedure 1 was used for the cyclopropanation of tert-butyl 4-methylenepiperidine-1-carboxylate (59.2 mg, 0.30 mmol, 1.5 equiv) with 2,2,2-trichloroethyl 2-(4-bromophenyl)-2-diazoacetate (74.5 mg, 0.20 mmol, 1.0 equiv) using Rh_2(*S*-pPhTPCP)$_4$ (7.0 μL, 0.50 mg/ml stock solution in DCM, 0.001 mol%) as catalyst at 39 ℃. Purification by column chromatography (0-20% diethyl ether/hexanes) afforded the product as a white solid (89.5 mg, 83% yield, 90% ee). *Note:* the enantioselectivity slightly decreased compared to 1.0 mol% and 0.5 mol% conditions.

Chiral HPLC: The enantiopurity was determined to be 90% ee by HPLC analysis (AD-H, 1 mL/min, 10% IPA/Hexane, λ=230 nm, RT: Major: 5.62 min., Minor: 4.78 min.)

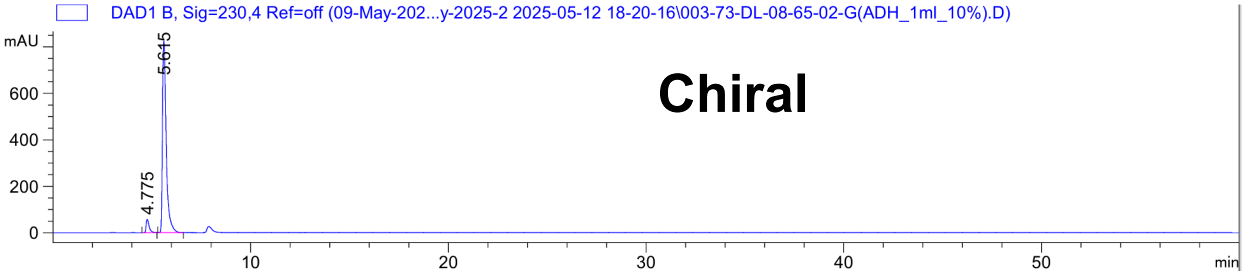

Signal 2: DAD1 B, Sig=230,4 Ref=off

Peak #	RetTime [min]	Type	Width [min]	Area [mAU*s]	Height [mAU]	Area %
1	4.775	BB	0.1633	605.05927	57.23265	5.2508
2	5.615	BB	0.2027	1.09181e4	823.63660	94.7492
Totals :				1.15231e4	880.86925	

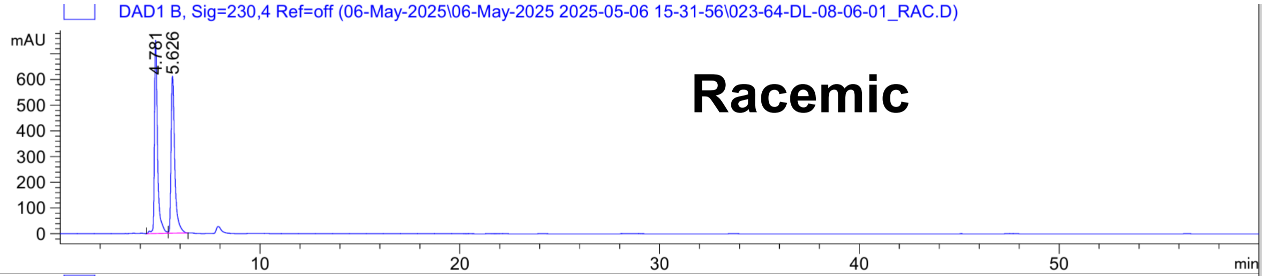

Signal 2: DAD1 B, Sig=230,4 Ref=off

Peak #	RetTime [min]	Type	Width [min]	Area [mAU*s]	Height [mAU]	Area %
1	4.781	VV R	0.1619	8081.54346	751.61932	50.5974
2	5.626	VB	0.1962	7890.69336	611.02423	49.4026

Totals : 1.59722e4 1362.64355

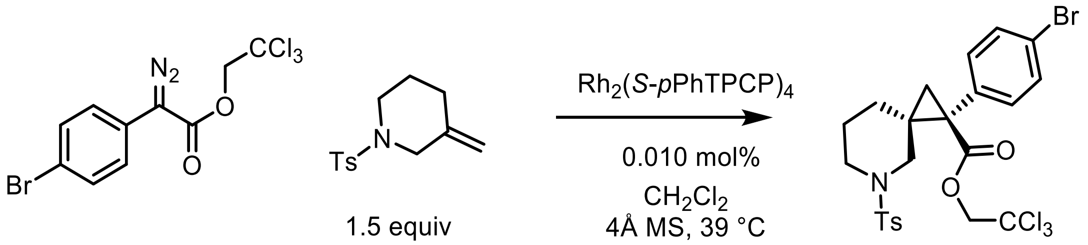

General procedure 1 was used for the cyclopropanation of 3-methylene-1-tosylpiperidine (75.4 mg, 0.30 mmol, 1.5 equiv) with 2,2,2-trichloroethyl 2-(4-bromophenyl)-2-diazoacetate (74.5 mg, 0.20 mmol, 1.0 equiv) using Rh_2(*S*-pPhTPCP)$_4$ (70.0 µL, 0.50 mg/ml stock solution in DCM, 0.01 mol%) as catalyst at 39℃. Purification by column chromatography (0-30% diethyl ether/hexanes) afforded the product as a white solid (97.9 mg, 82% yield, 14:1 dr, 95% ee). *Note:* the diastereoselectivity slightly decreased compared to 1.0 mol% and 0.5 mol% conditions.

Chiral HPLC: The enantiopurity was determined to be 95% ee by HPLC analysis (AD-H, 1 mL/min, 10% IPA/Hexane, λ=230 nm, RT: Major: 27.67 min., Minor: 24.11 min.)

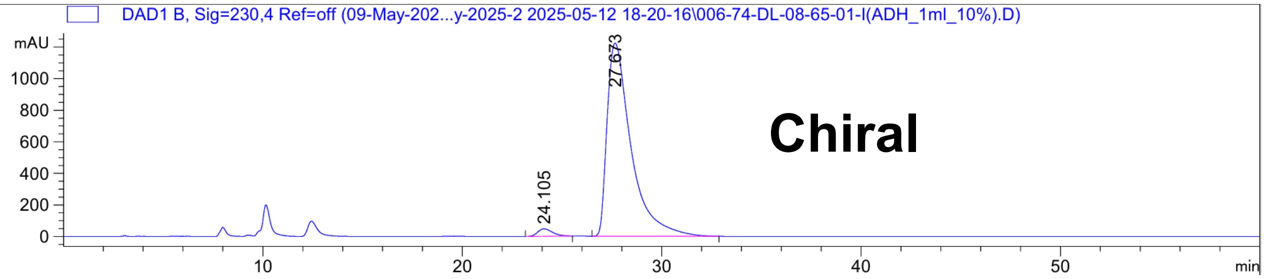

Signal 2: DAD1 B, Sig=230,4 Ref=off

Peak #	RetTime [min]	Type	Width [min]	Area [mAU*s]	Height [mAU]	Area %
1	24.105	BB	0.6013	2429.47461	47.33222	2.3147
2	27.673	BB	0.9801	1.02531e5	1223.54468	97.6853

Totals : 1.04960e5 1270.87690

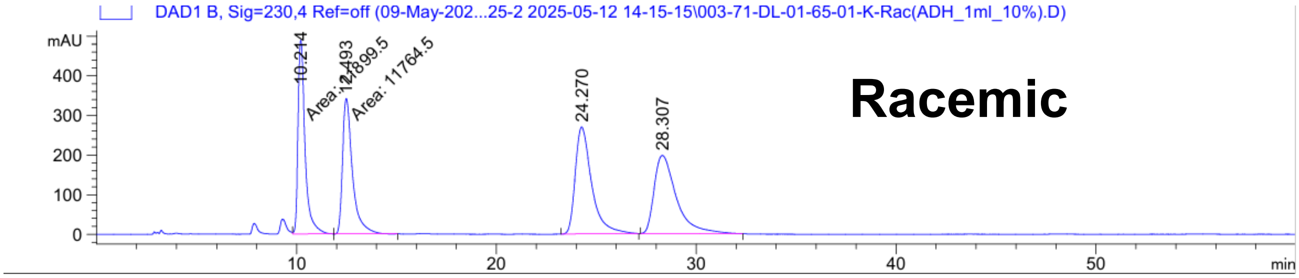

Signal 2: DAD1 B, Sig=230,4 Ref=off

Peak #	RetTime [min]	Type	Width [min]	Area [mAU*s]	Height [mAU]	Area %
1	10.214	FM	0.4062	1.18995e4	488.26547	21.9253
2	12.493	FM	0.5742	1.17645e4	341.48871	21.6767
3	24.270	BB	0.6728	1.53941e4	269.94220	28.3643
4	28.307	BB	0.9012	1.52147e4	197.28365	28.0338

Totals : 5.42728e4 1296.98003

6. NMR for Diastereomer Determination

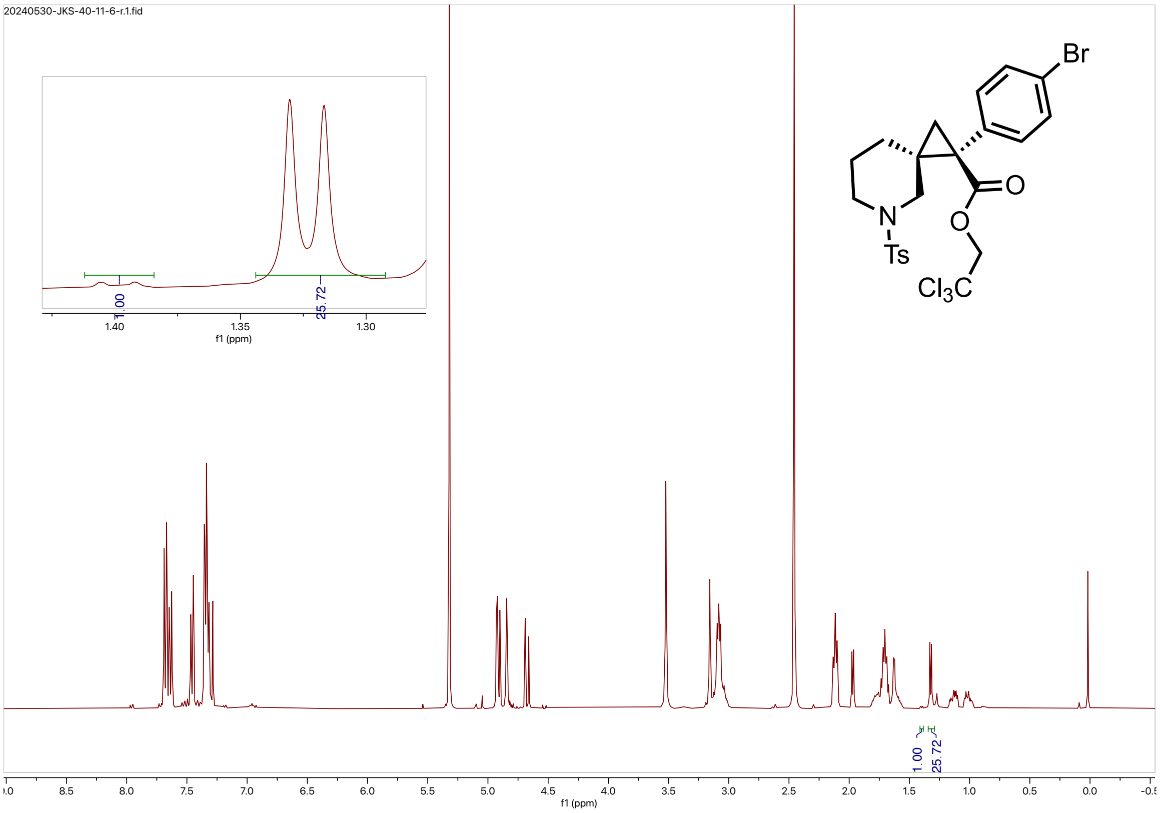

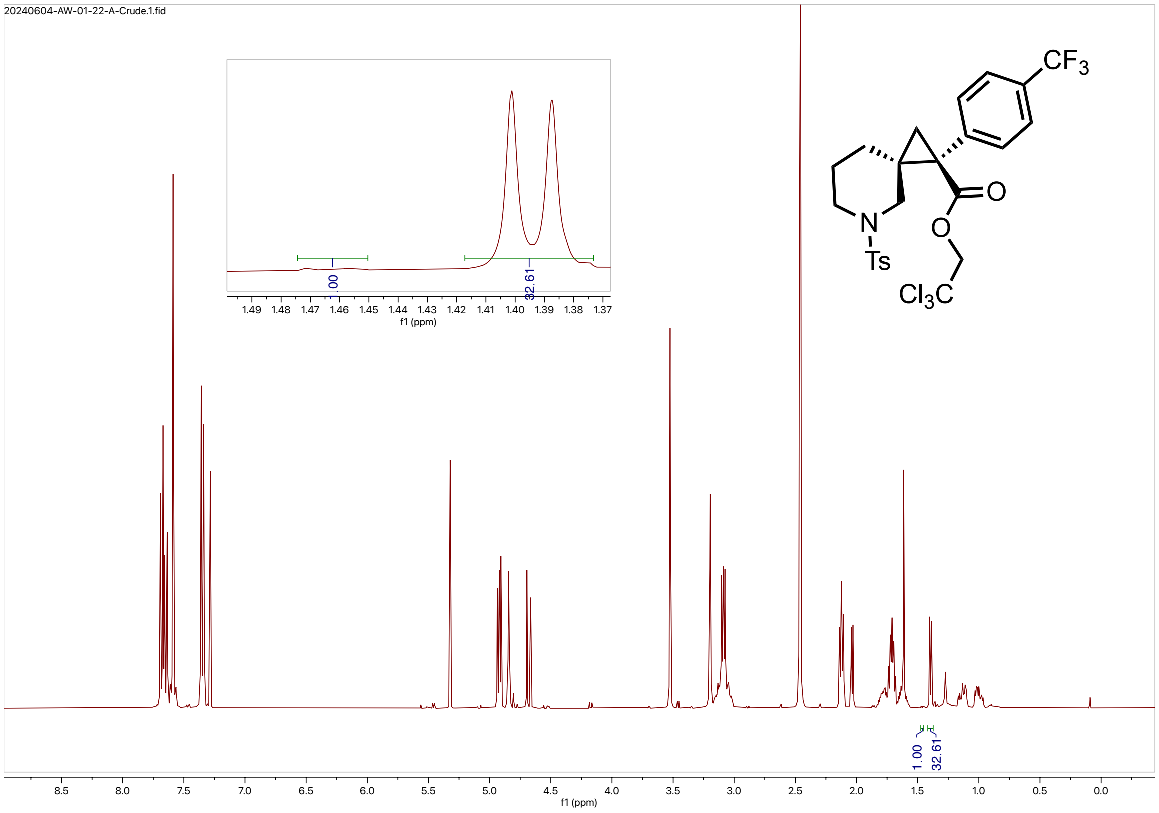

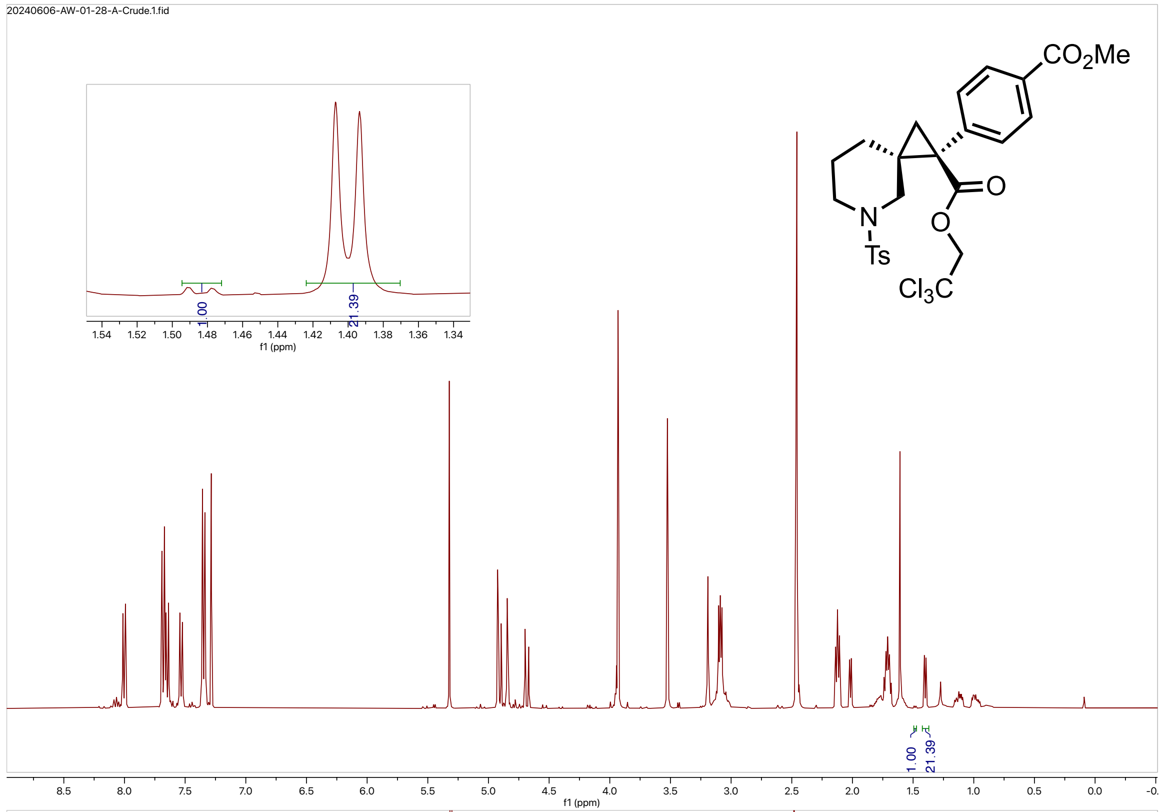
20240606-AW-01-28-A-Crude.1.fid
CO_2Me
N
Ts
O
O
Cl_3C
1.00
21.39
f1 (ppm)

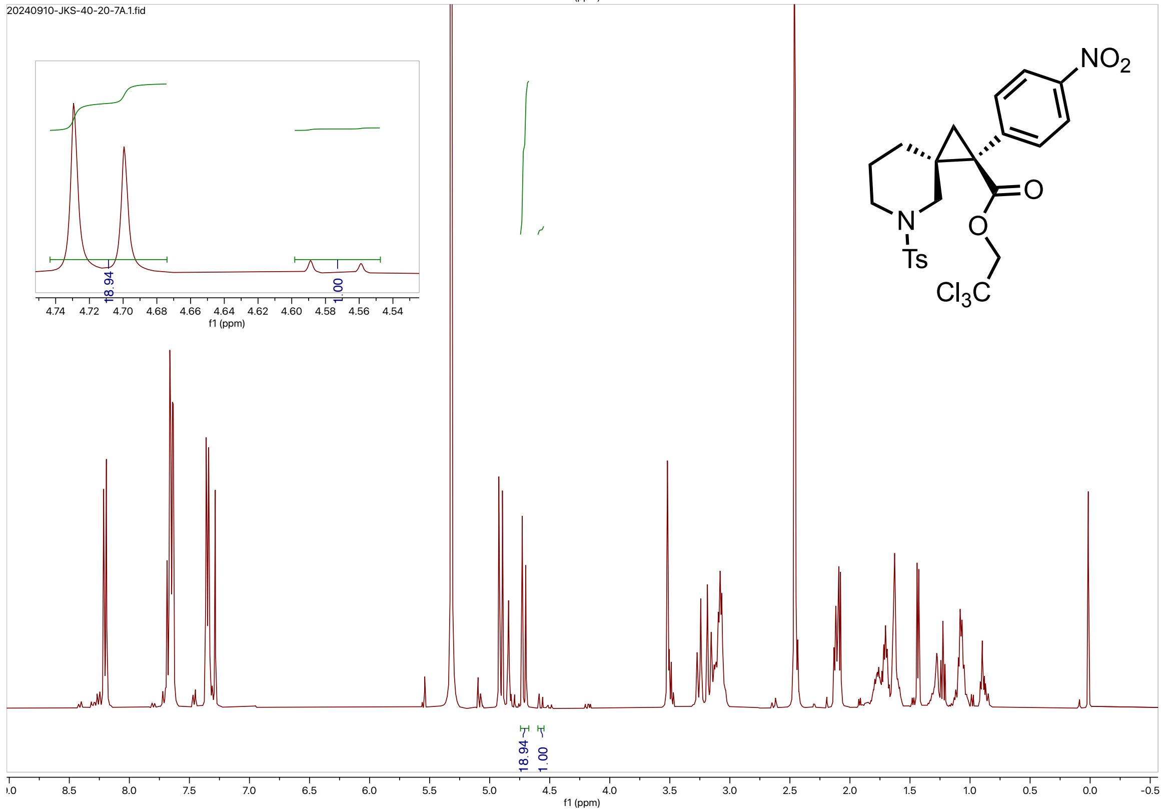
20240910-JKS-40-20-7A.1.fid
NO_2
N
Ts
O
O
Cl_3C
18.94
1.00
f1 (ppm)

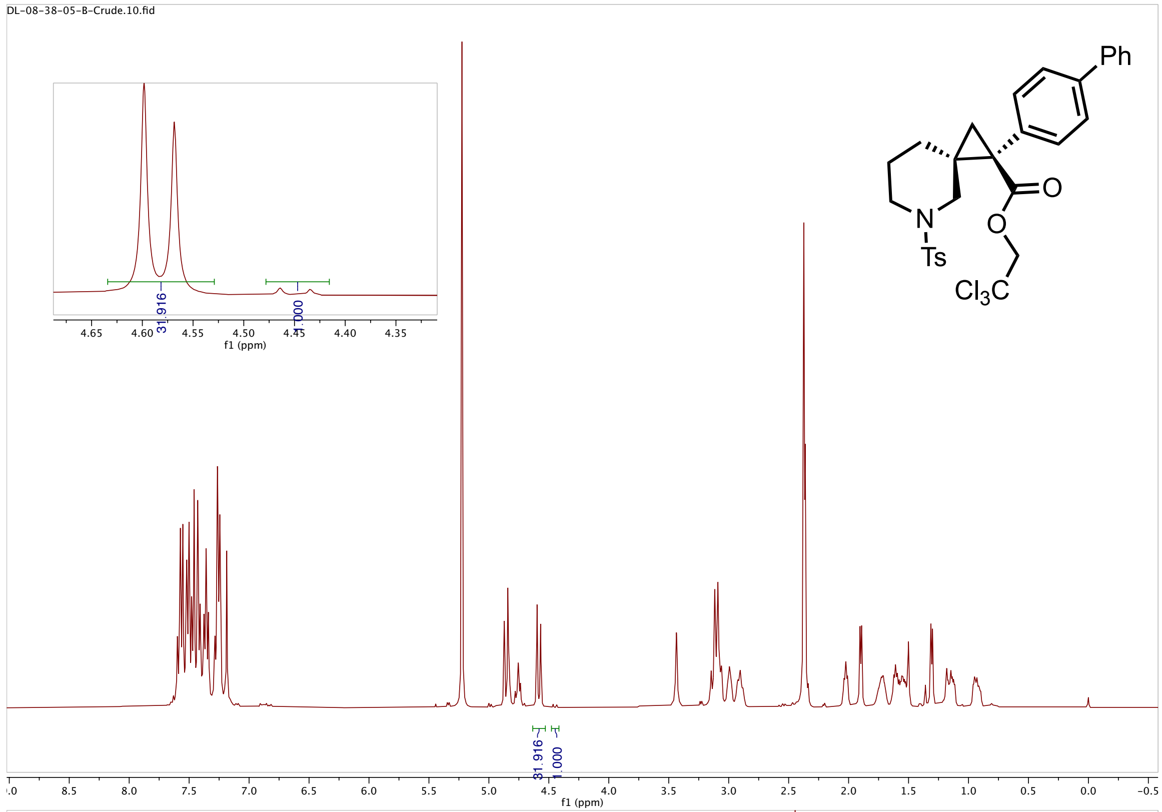
DL-08-38-05-B-Crude.10.fid
Ph
N
Ts
O
O
Cl3C
31.916
1.000
f1 (ppm)

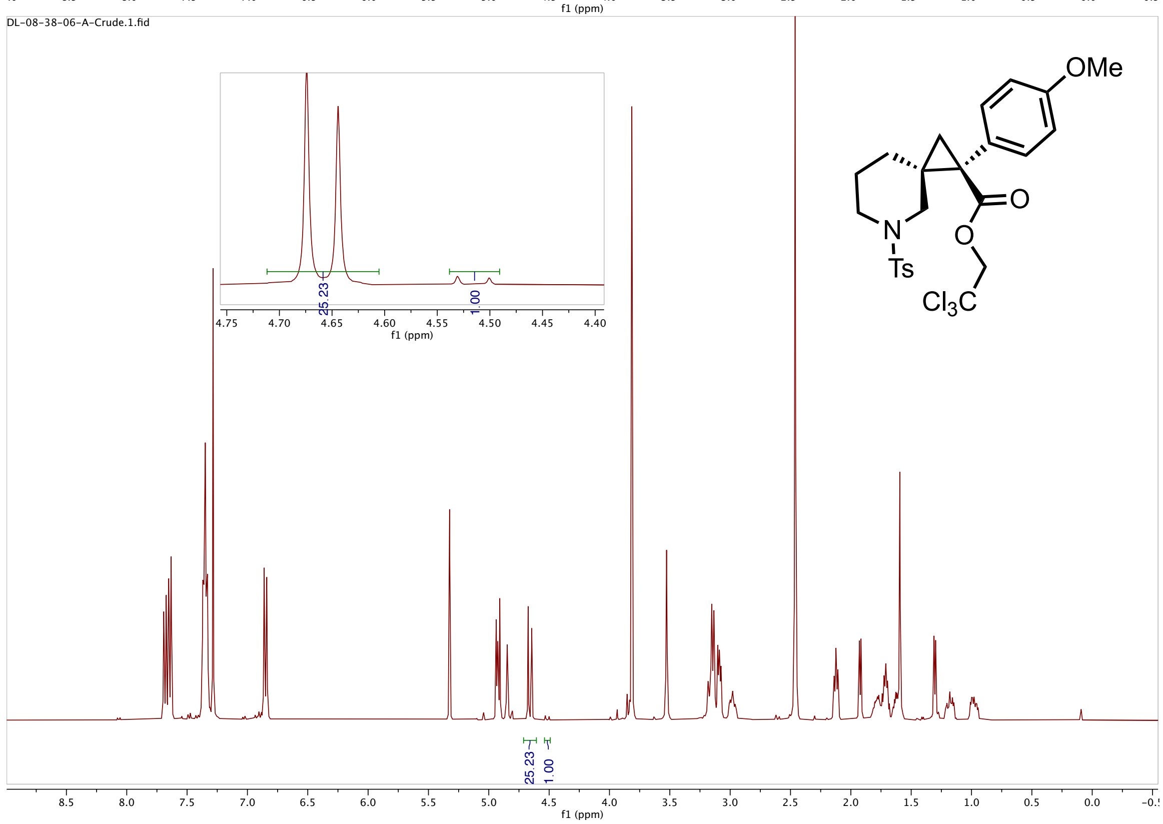
DL-08-38-06-A-Crude.1.fid
OMe
N
Ts
O
O
Cl3C
25.23
1.00
f1 (ppm)

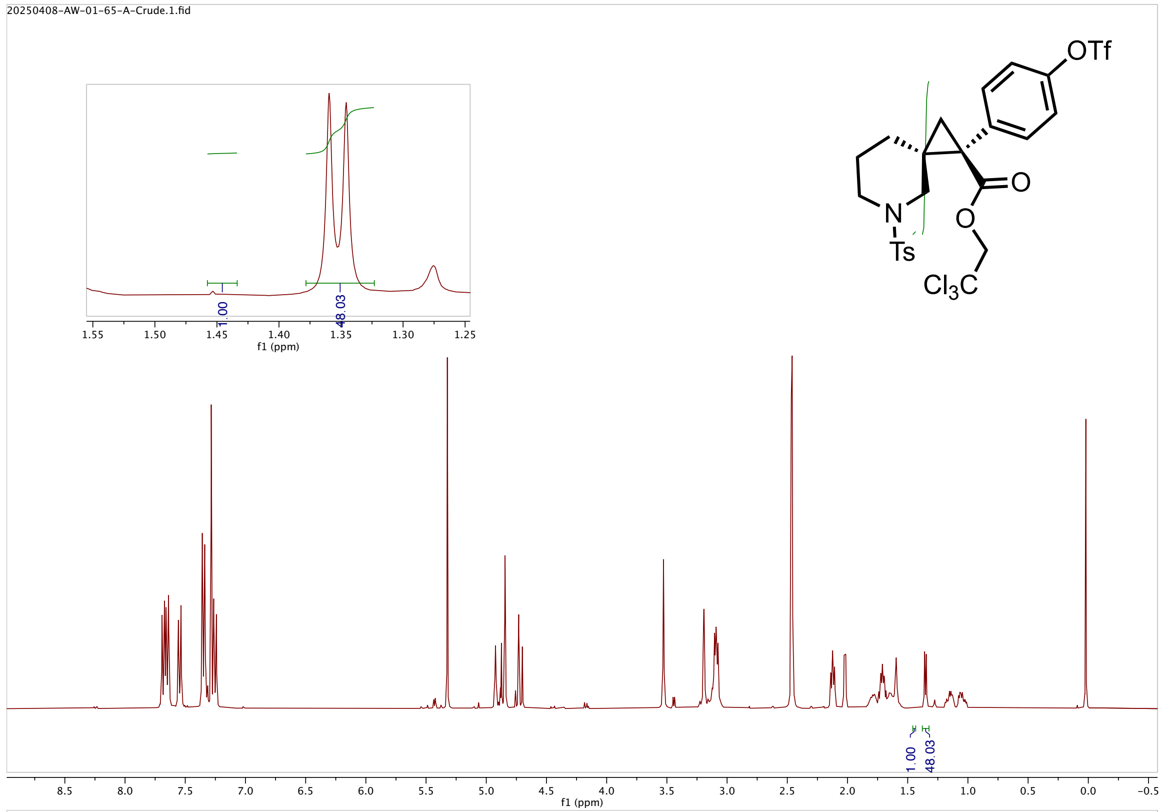
20250408-AW-01-65-A-Crude.1.fid
OTf
O
O
N
Ts
Cl3C
1.00
48.03
f1 (ppm)

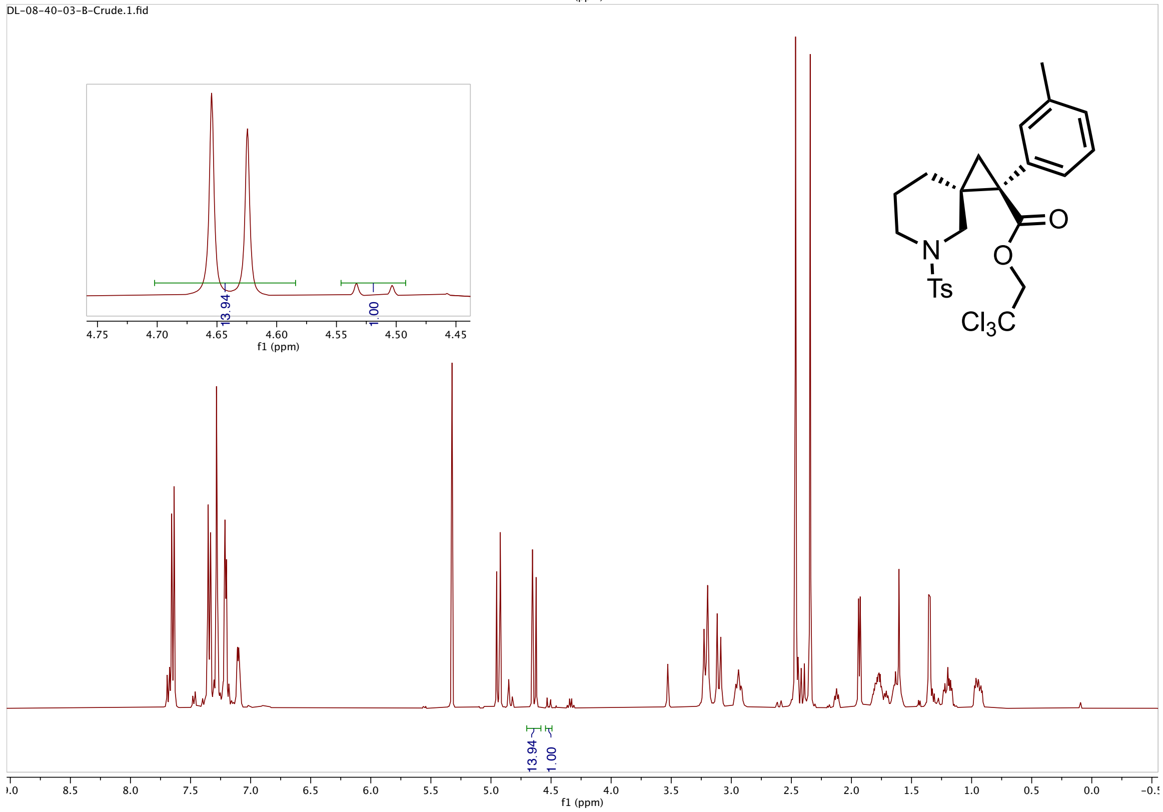
DL-08-40-03-B-Crude.1.fid
O
O
N
Ts
Cl3C
13.94
1.00
f1 (ppm)

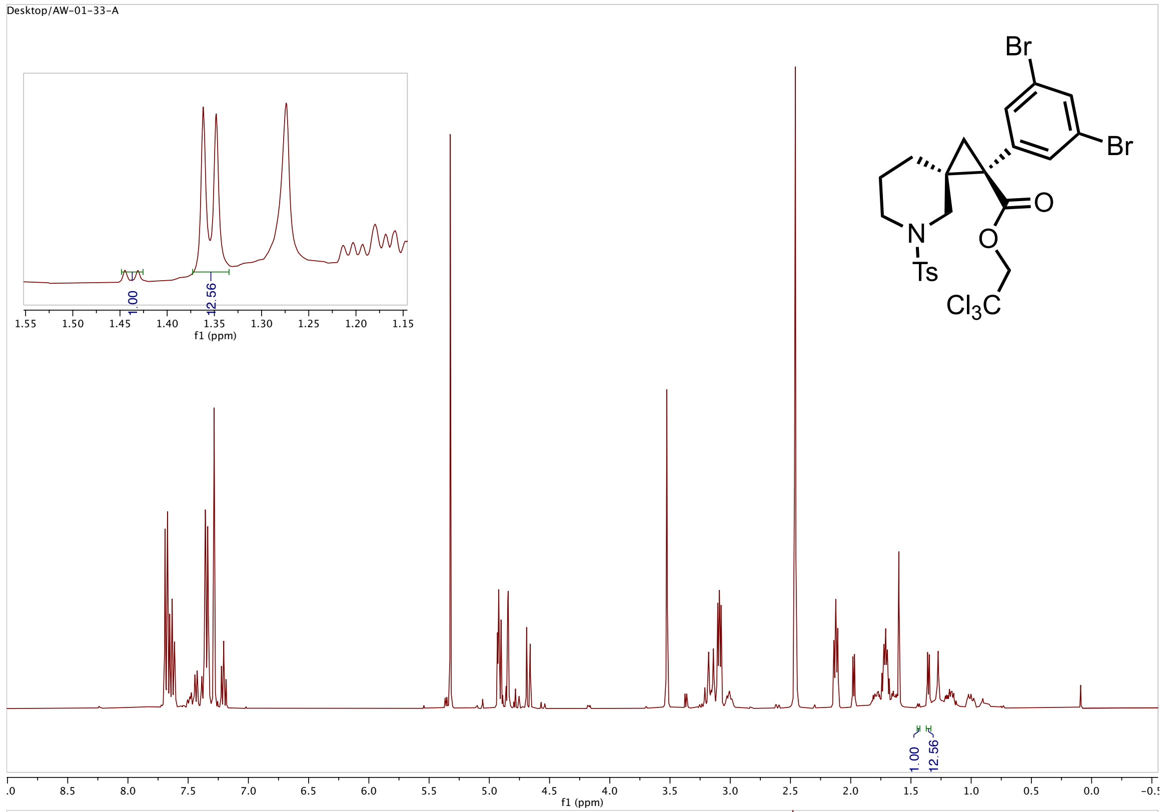
Desktop/AW-01-33-A
Br
Br
O
O
N
Ts
Cl3C
1.00
12.56
f1 (ppm)

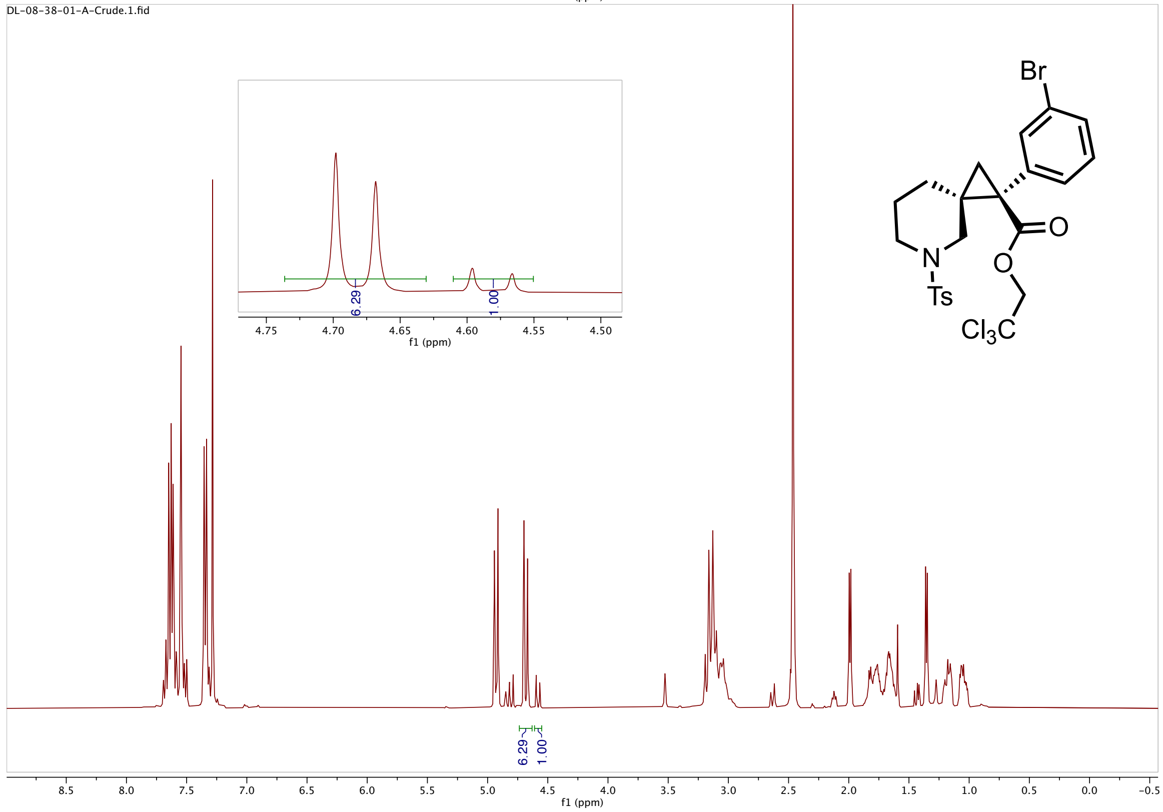
DL-08-38-01-A-Crude.1.fid
Br
O
O
N
Ts
Cl3C
6.29
1.00
f1 (ppm)

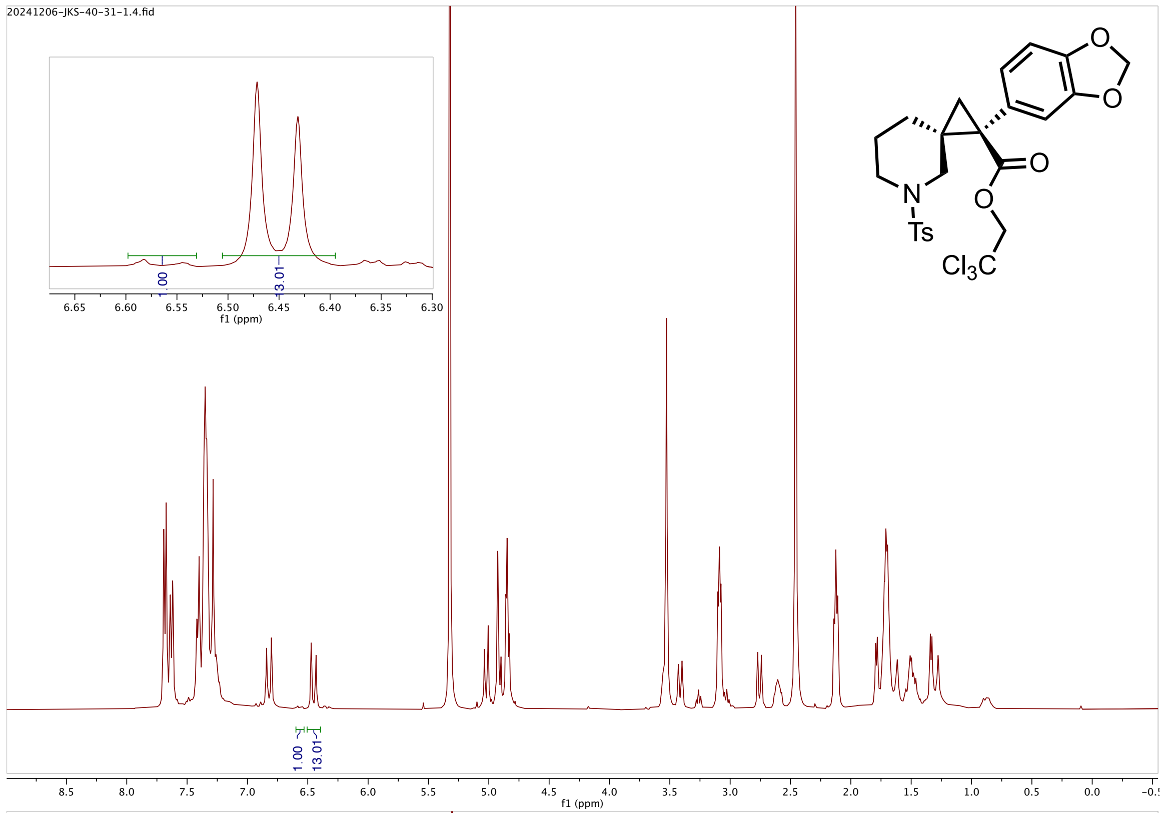
20241206-JKS-40-31-1.4.fid
f1 (ppm)
1.00
13.01
N
Ts
O
O
O
O
Cl3C

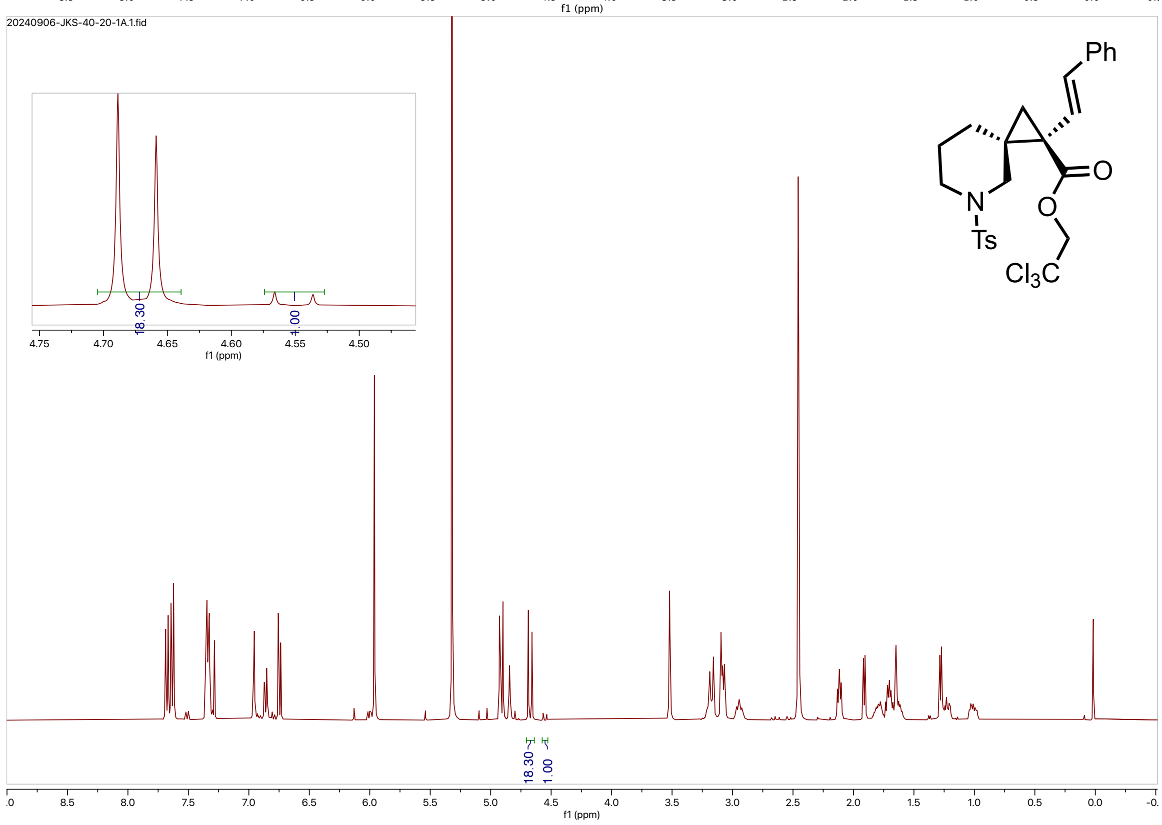
20240906-JKS-40-20-1A.1.fid
f1 (ppm)
18.30
1.00
Ph
N
Ts
O
O
Cl3C

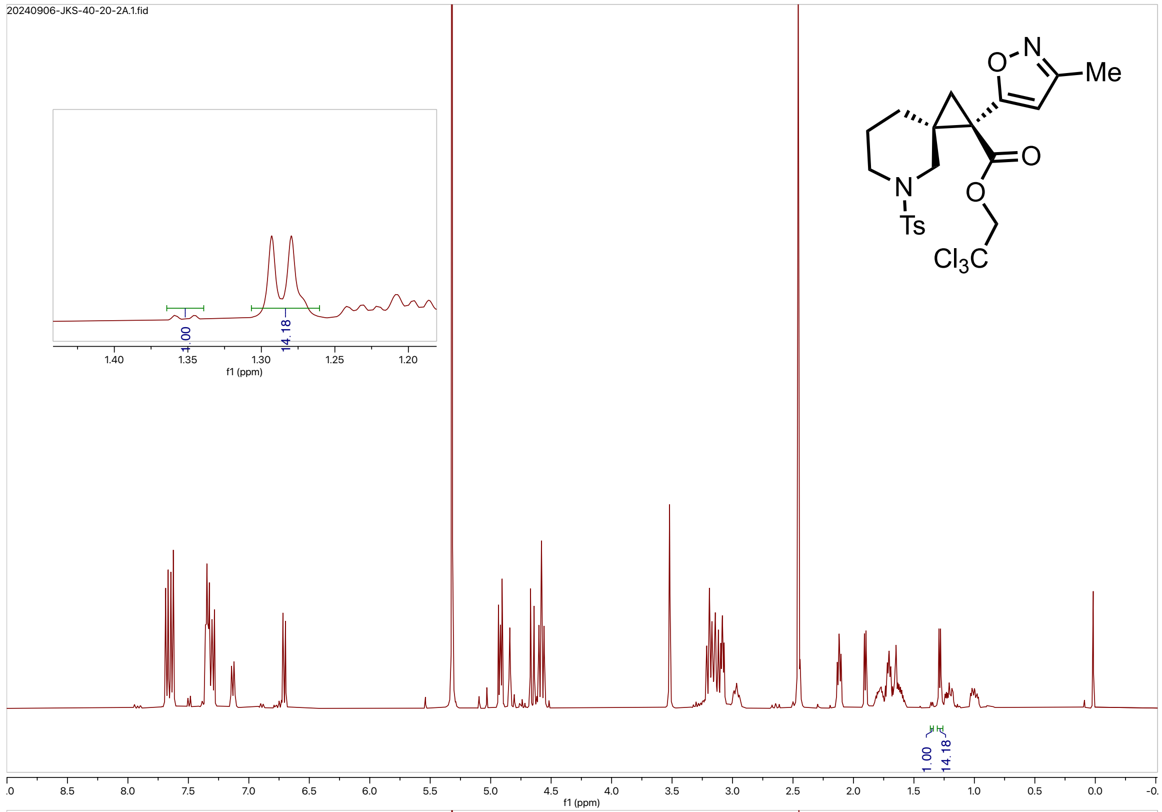
20240906-JKS-40-20-2A.1.fid
O
N
Me
O
N
Ts
O
Cl3C
1.00
14.18
f1 (ppm)

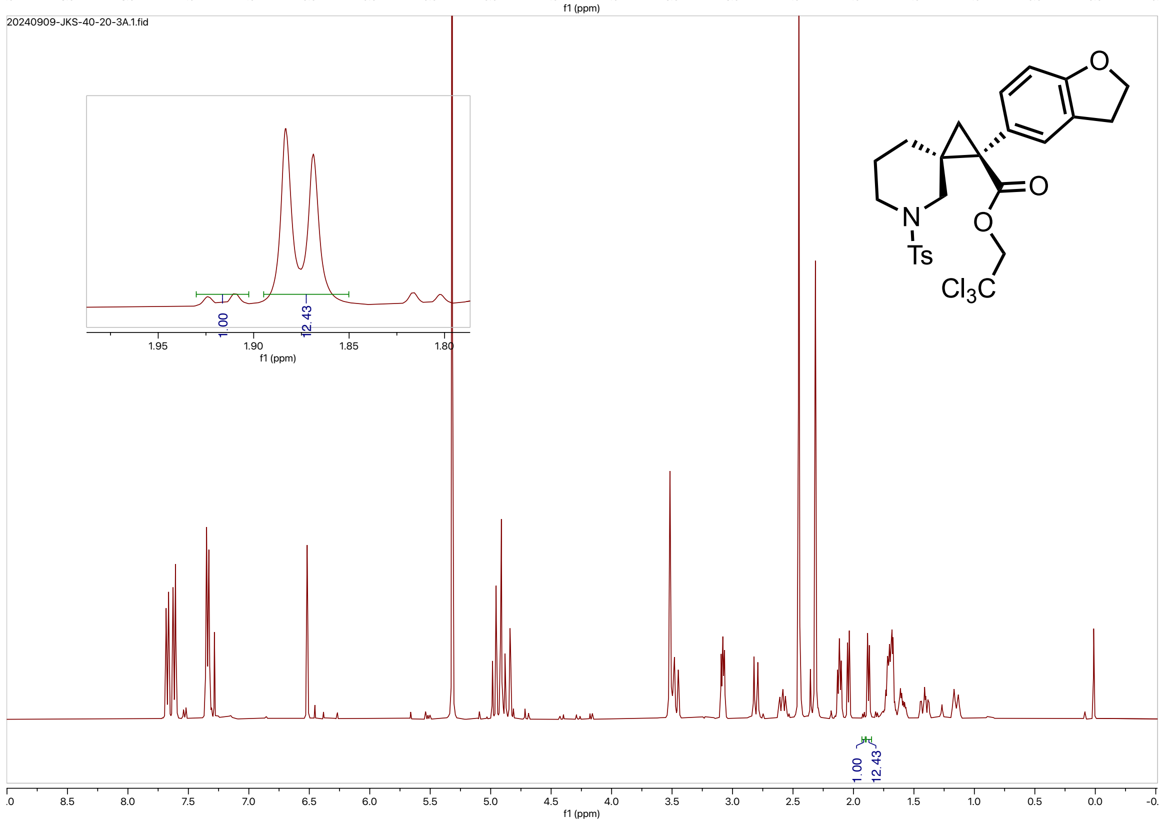
20240909-JKS-40-20-3A.1.fid
O
O
N
Ts
O
Cl3C
1.00
12.43
f1 (ppm)

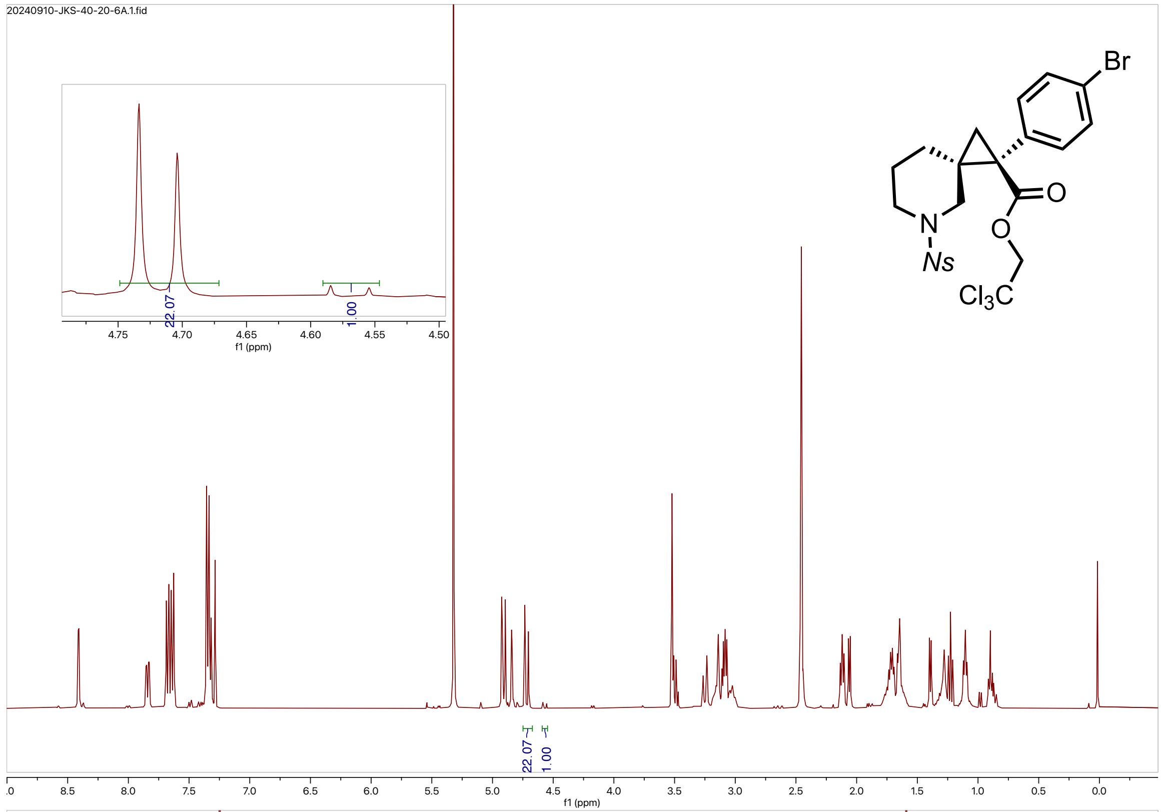
20240910-JKS-40-20-6A.1.fid
Br
N
Ns
O
O
Cl3C
22.07
1.00
f1 (ppm)

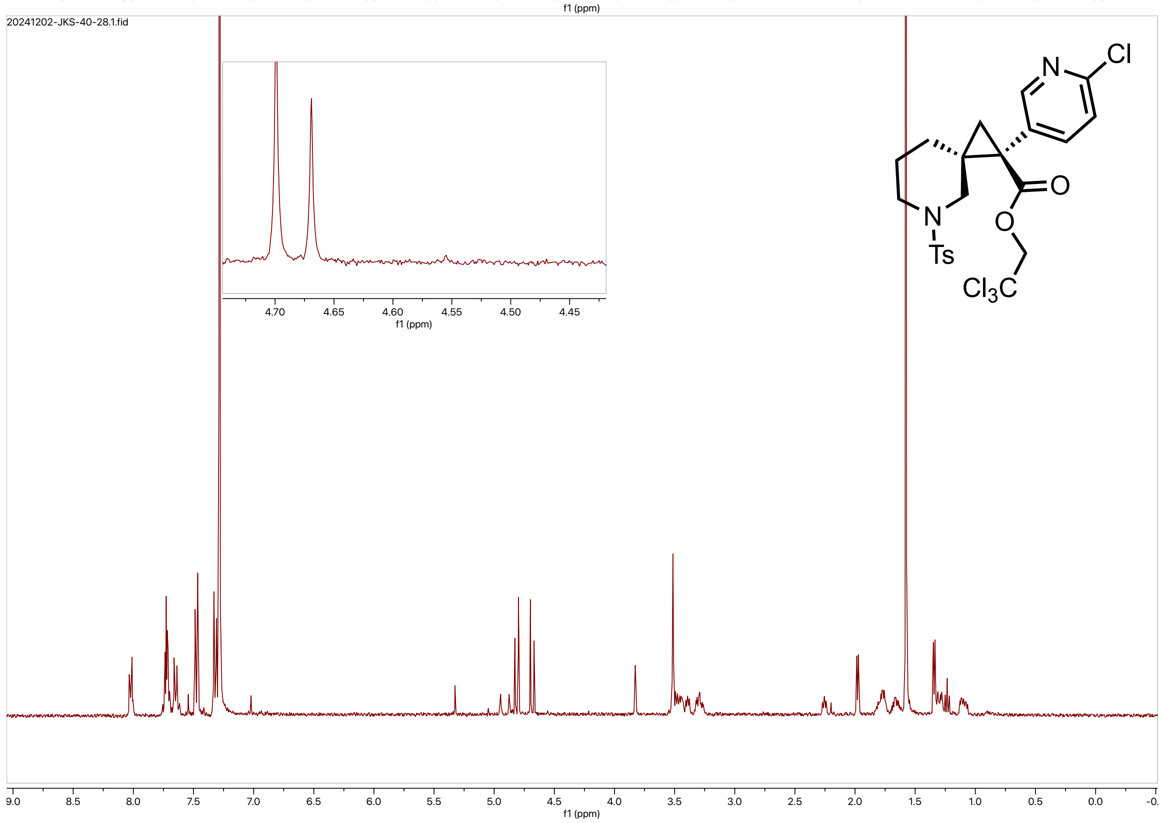
20241202-JKS-40-28.1.fid
N
Cl
N
Ts
O
O
Cl3C
f1 (ppm)

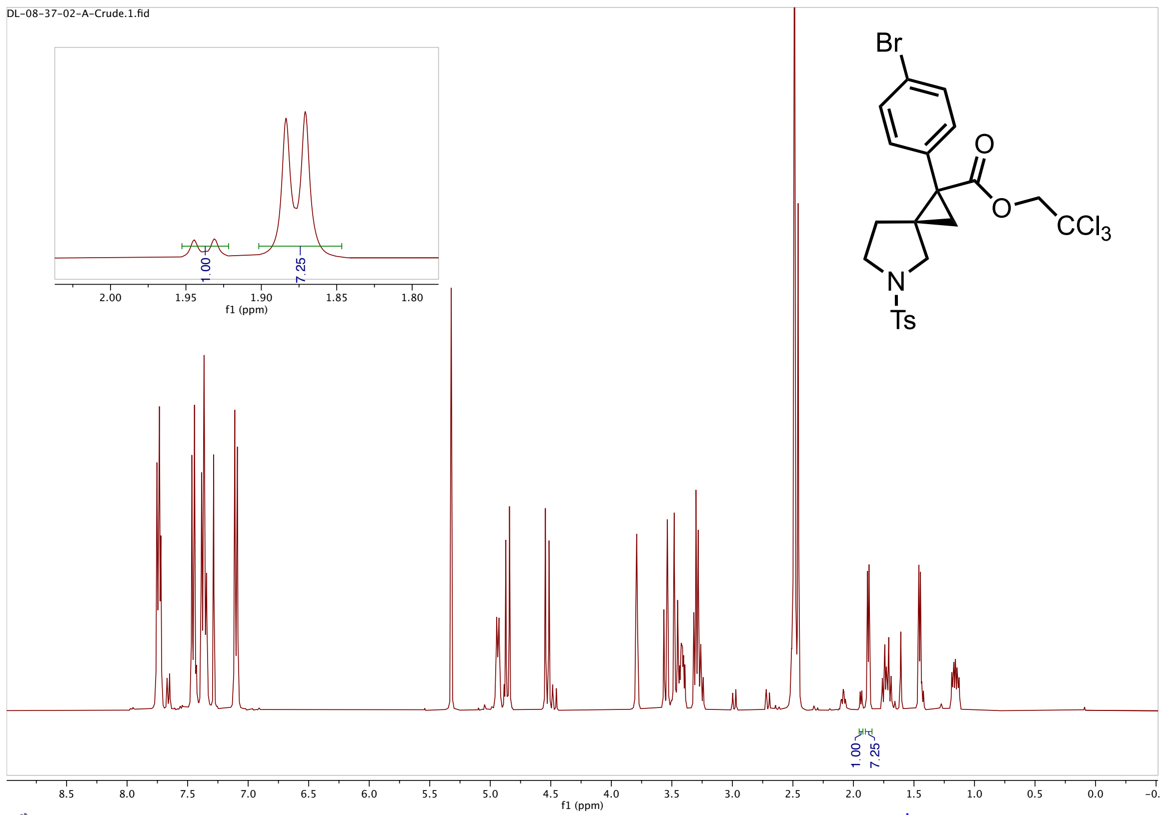

DL-08-37-02-A-Crude.1.fid
Br
O
O
CCl3
N
Ts
1.00
7.25
f1 (ppm)

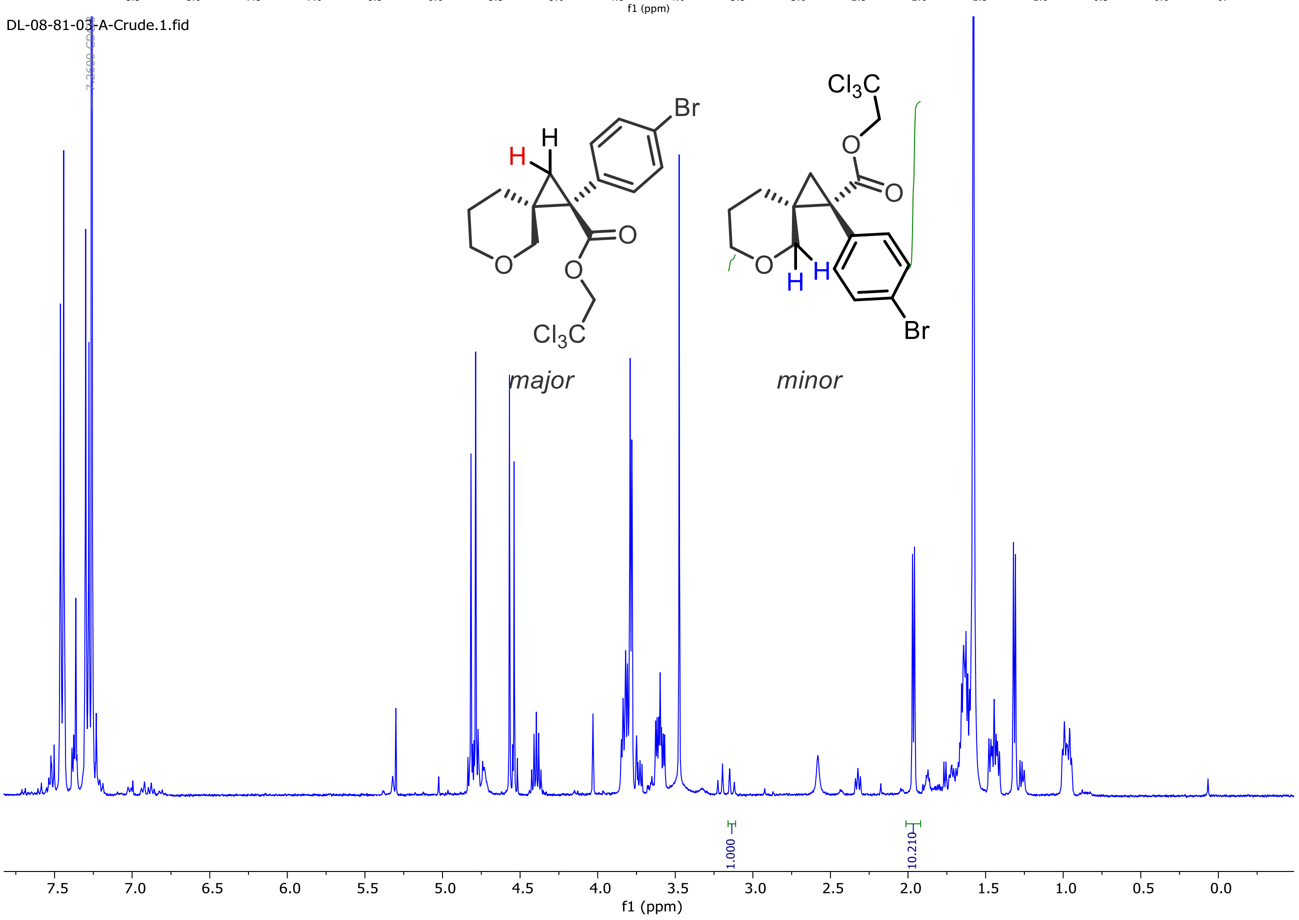

DL-08-81-03-A-Crude.1.fid
Br
H
H
O
O
O
Cl3C
major
Cl3C
O
O
O
H
H
Br
minor
1.000
10.210
f1 (ppm)

7. HPLC and SFC Chiral Traces

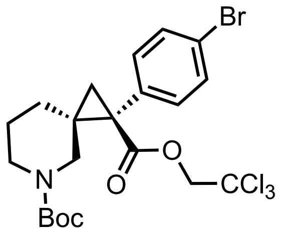

Compound 15a

OJ3_3%MeOH_IPA_0_2% Formic Acid_2.5mL/min_5min

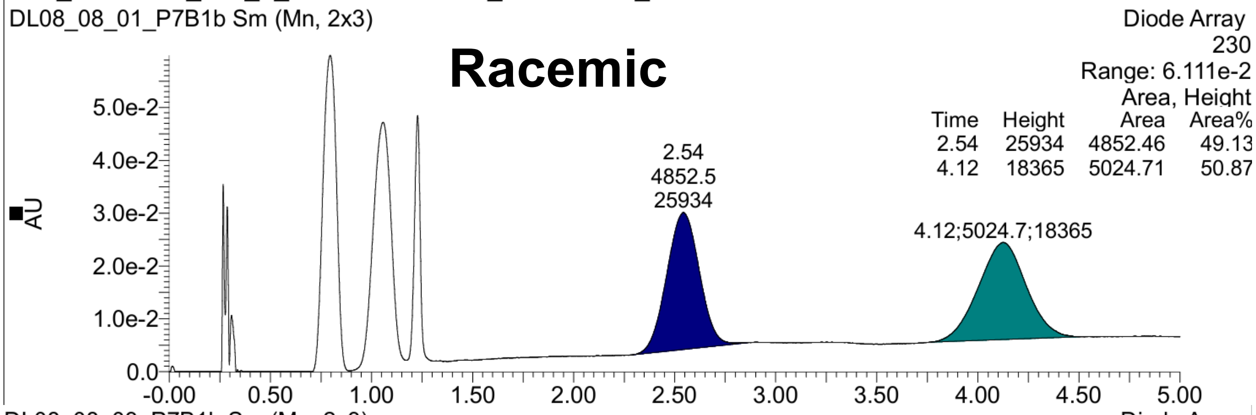

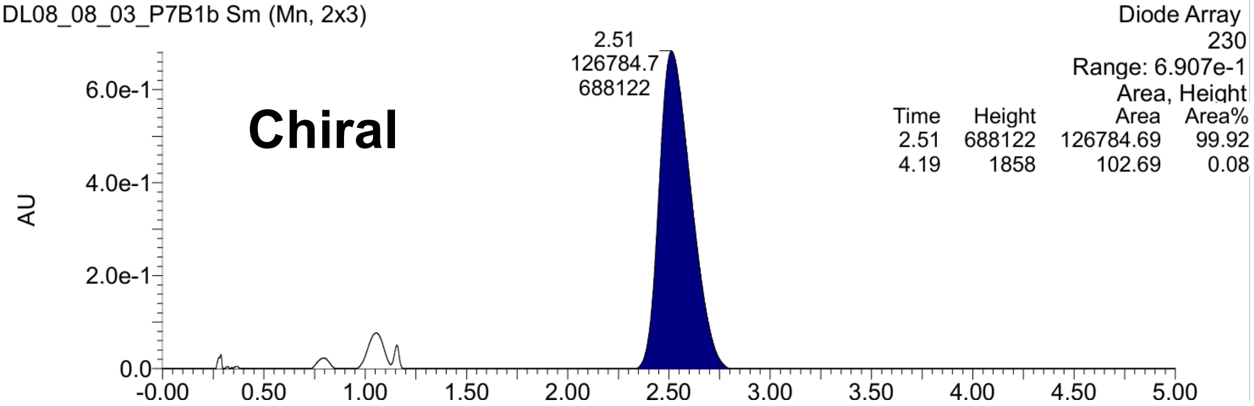

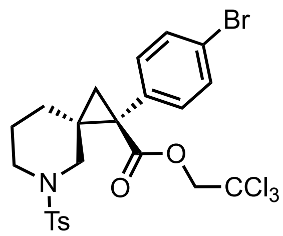

Compound 15b

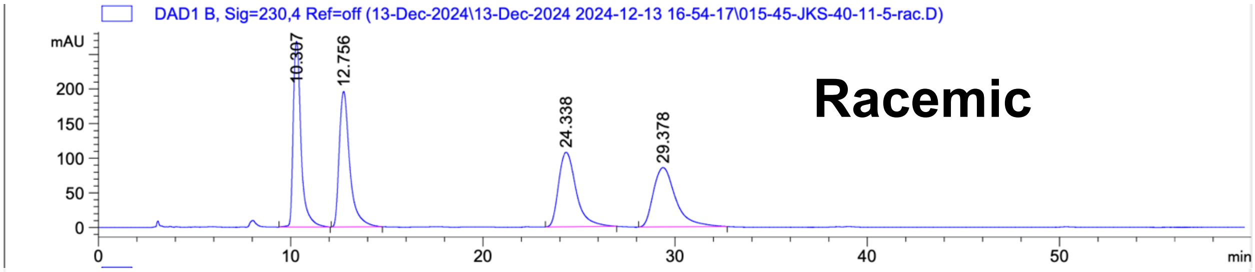

Signal 2: DAD1 B, Sig=230,4 Ref=off

Peak #	RetTime [min]	Type	Width [min]	Area [mAU*s]	Height [mAU]	Area %
1	10.307	BB	0.4240	7579.24902	268.33194	26.2699
2	12.756	BB	0.5437	7440.13916	195.83569	25.7878
3	24.338	BB	0.7523	6905.04004	107.58592	23.9331
4	29.378	BB	0.9456	6927.00830	85.73902	24.0092
Totals :				2.88514e4	657.49258	

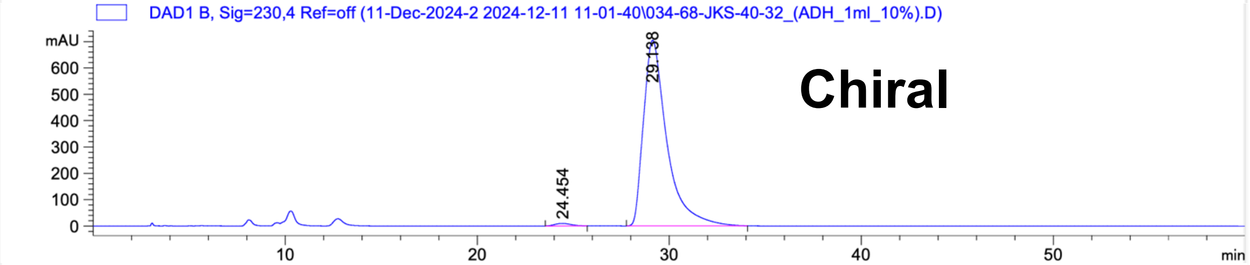

Signal 2: DAD1 B, Sig=230,4 Ref=off

Peak #	RetTime [min]	Type	Width [min]	Area [mAU*s]	Height [mAU]	Area %
1	24.454	BB	0.6733	540.69104	9.41764	0.8991
2	29.138	BB	0.9910	5.95989e4	704.82300	99.1009
Totals :				6.01396e4	714.24064	

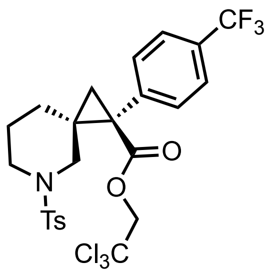

Compound 16

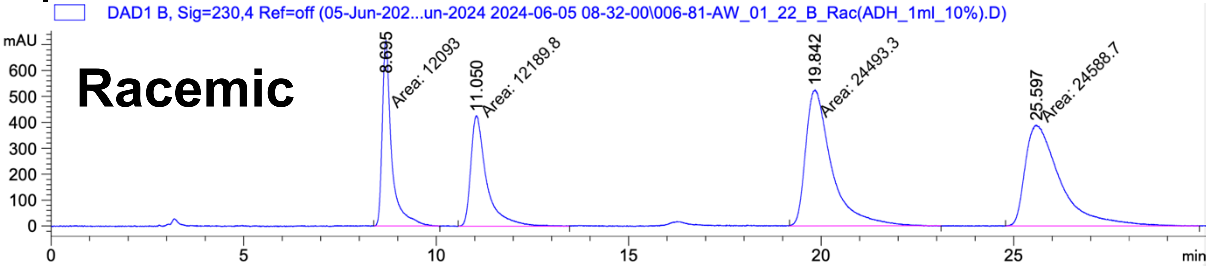

Signal 2: DAD1 B, Sig=230,4 Ref=off

Peak #	RetTime [min]	Type	Width [min]	Area [mAU*s]	Height [mAU]	Area %
1	8.695	MM	0.2811	1.20930e4	717.11755	16.4834
2	11.050	MM	0.4760	1.21898e4	426.84717	16.6153
3	19.842	MM	0.7774	2.44933e4	525.12231	33.3856
4	25.597	MM	1.0536	2.45887e4	388.97781	33.5157

Totals : 7.33649e4 2058.06485

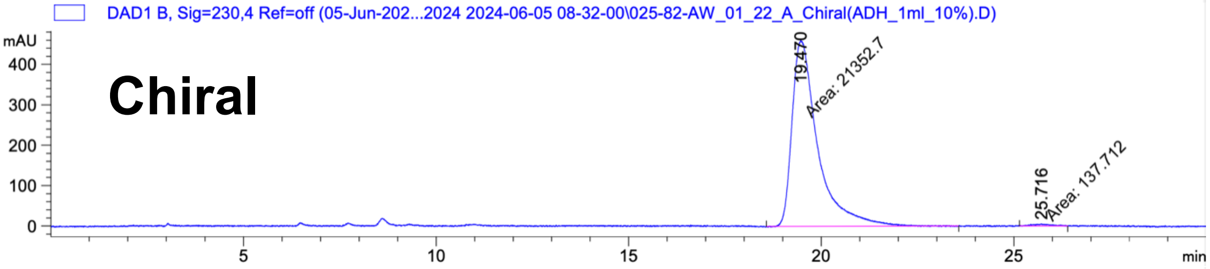

Signal 2: DAD1 B, Sig=230,4 Ref=off

Peak #	RetTime [min]	Type	Width [min]	Area [mAU*s]	Height [mAU]	Area %
1	19.470	MM	0.7735	2.13527e4	460.09781	99.3592
2	25.716	MM	0.4654	137.71191	4.93118	0.6408

Totals : 2.14904e4 465.02899

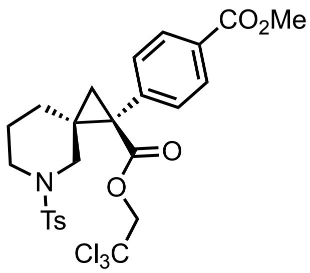

Compound 17

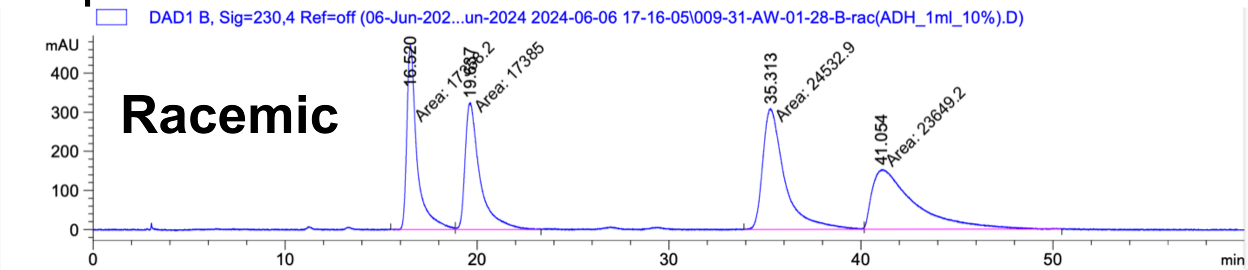

Signal 2: DAD1 B, Sig=230,4 Ref=off

Peak #	RetTime [min]	Type	Width [min]	Area [mAU*s]	Height [mAU]	Area %
1	16.520	MF	0.6129	1.73582e4	472.04211	20.9324
2	19.637	FM	0.8936	1.73850e4	324.23386	20.9646
3	35.313	MF	1.3217	2.45329e4	309.36719	29.5844
4	41.054	FM	2.5788	2.36492e4	152.84401	28.5187
Totals :				8.29253e4	1258.48717	

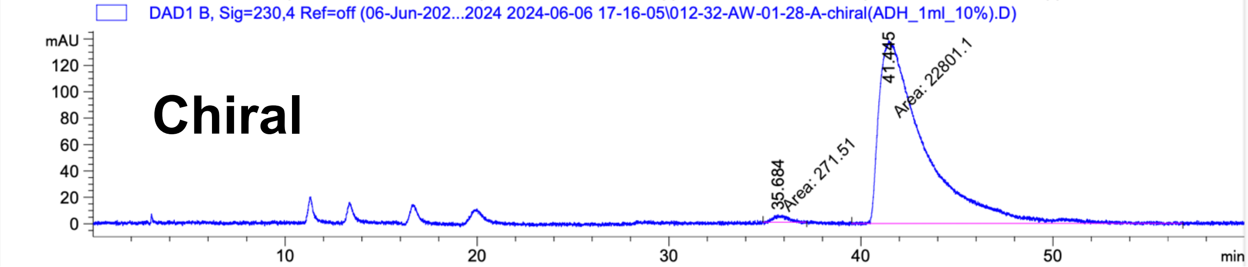

Signal 2: DAD1 B, Sig=230,4 Ref=off

Peak #	RetTime [min]	Type	Width [min]	Area [mAU*s]	Height [mAU]	Area %
1	35.684	MM	0.7741	271.50958	5.84591	1.1768
2	41.445	MM	2.7371	2.28011e4	138.84218	98.8232
Totals :				2.30726e4	144.68809	

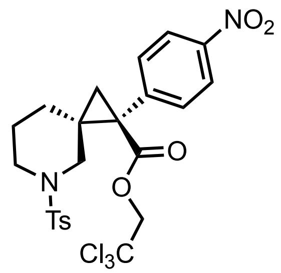
NO2
O
N
Ts
O
Cl3C

Compound 18

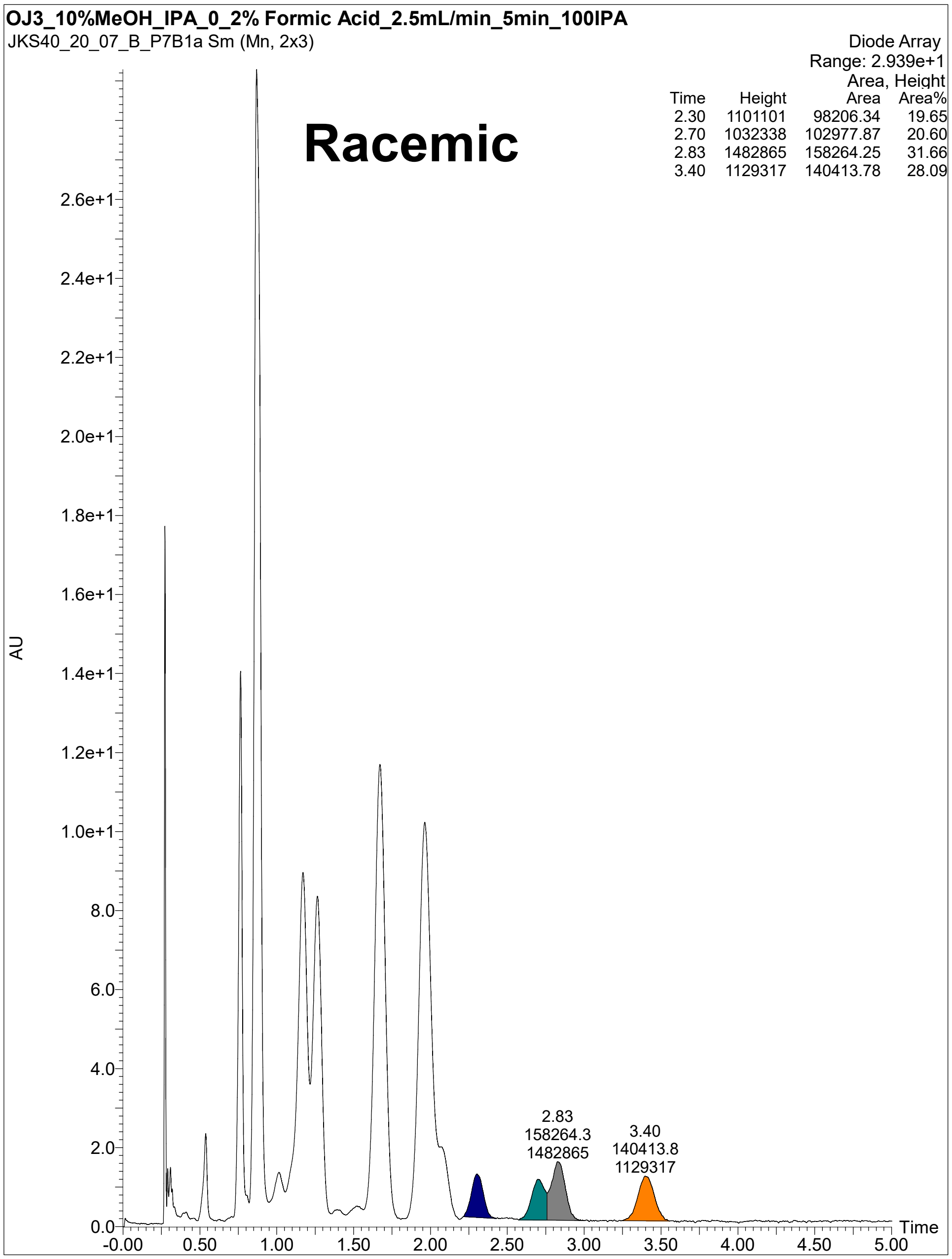
OJ3_10%MeOH_IPA_0_2% Formic Acid_2.5mL/min_5min_100IPA
JKS40_20_07_B_P7B1a Sm (Mn, 2x3)
Diode Array
Range: 2.939e+1
Area, Height
Time Height Area Area%
2.30 1101101 98206.34 19.65
2.70 1032338 102977.87 20.60
2.83 1482865 158264.25 31.66
3.40 1129317 140413.78 28.09
Racemic
AU
2.83
158264.3
1482865
3.40
140413.8
1129317
Time

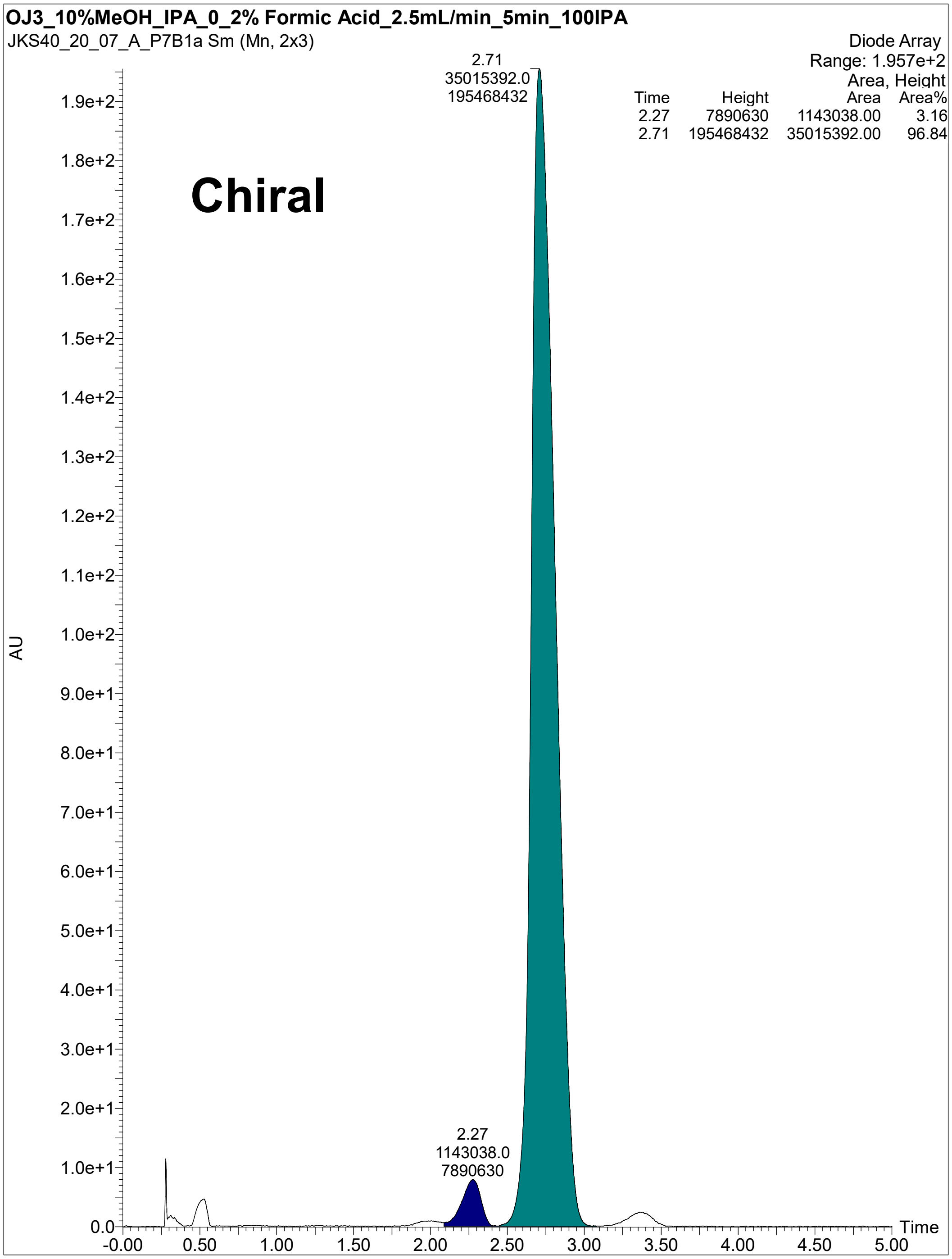
OJ3_10%MeOH_IPA_0_2% Formic Acid_2.5mL/min_5min_100IPA
JKS40_20_07_A_P7B1a Sm (Mn, 2x3)
Diode Array
Range: 1.957e+2
Area, Height
Time Height Area Area%
2.27 7890630 1143038.00 3.16
2.71 195468432 35015392.00 96.84
Chiral
2.71
35015392.0
195468432
2.27
1143038.0
7890630
AU
Time

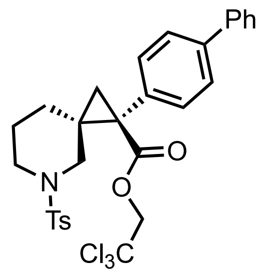

Compound 19

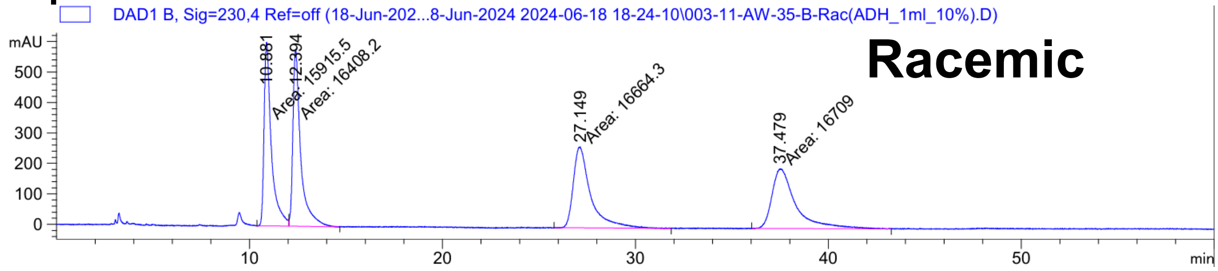

Signal 2: DAD1 B, Sig=230,4 Ref=off

Peak #	RetTime [min]	Type	Width [min]	Area [mAU*s]	Height [mAU]	Area %
1	10.881	MF	0.4399	1.59155e4	603.01996	24.2256
2	12.394	FM	0.4706	1.64082e4	581.11865	24.9756
3	27.149	MM	1.0453	1.66643e4	265.68961	25.3654
4	37.479	MM	1.4157	1.67090e4	196.70488	25.4334

Totals : 6.56970e4 1646.53310

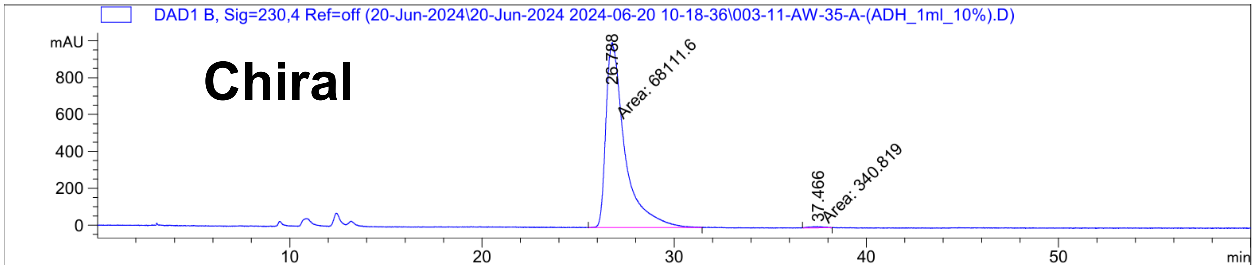

Signal 2: DAD1 B, Sig=230,4 Ref=off

Peak #	RetTime [min]	Type	Width [min]	Area [mAU*s]	Height [mAU]	Area %
1	26.788	MM	1.1299	6.81116e4	1004.72119	99.5021
2	37.466	MM	0.7474	340.81906	7.60009	0.4979

Totals : 6.84524e4 1012.32129

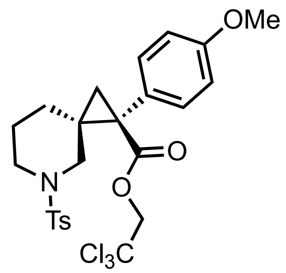
OMe
O
N
O
Ts
Cl3C

Compound 20

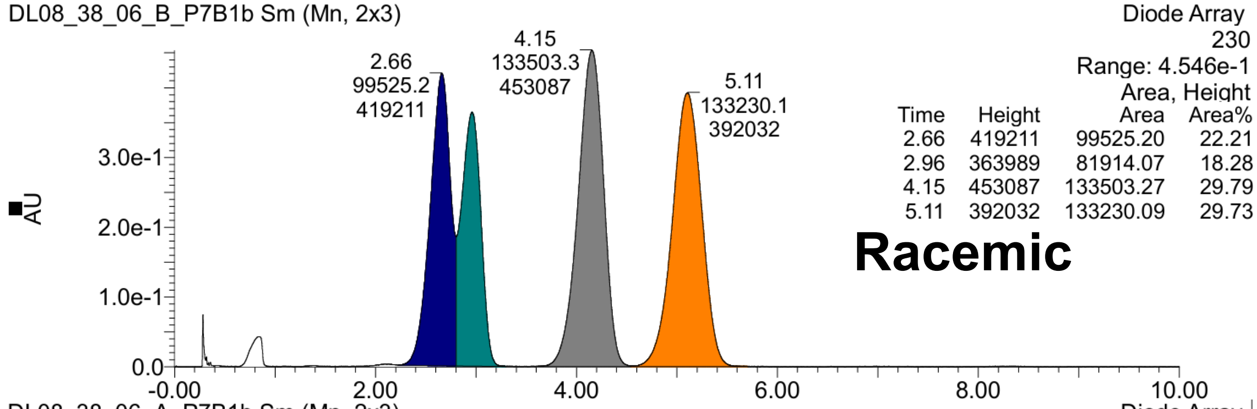
DL08_38_06_B_P7B1b Sm (Mn, 2x3)
Diode Array
230
Range: 4.546e-1
Area, Height
Time Height Area Area%
2.66 419211 99525.20 22.21
2.96 363989 81914.07 18.28
4.15 453087 133503.27 29.79
5.11 392032 133230.09 29.73
2.66
99525.2
419211
4.15
133503.3
453087
5.11
133230.1
392032
Racemic
AU
3.0e-1
2.0e-1
1.0e-1
0.0
-0.00
2.00
4.00
6.00
8.00
10.00

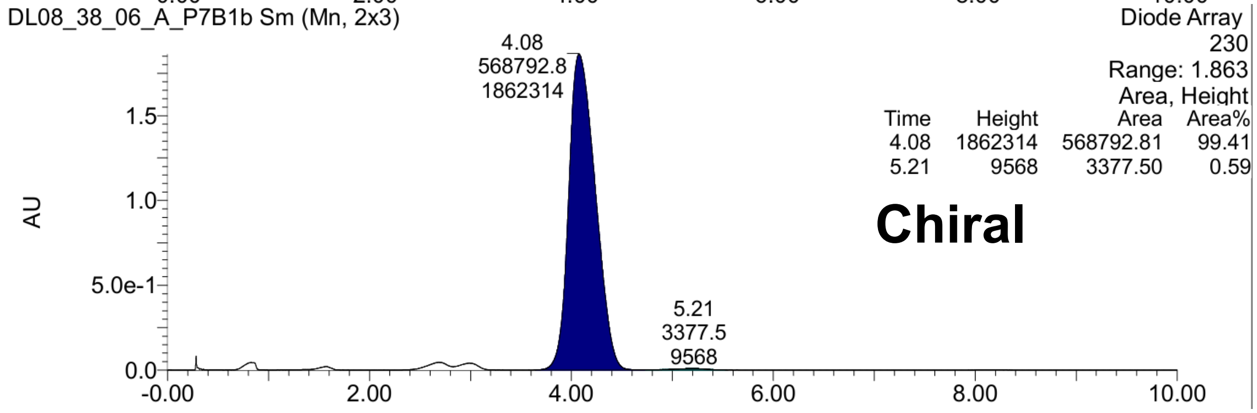
DL08_38_06_A_P7B1b Sm (Mn, 2x3)
Diode Array
230
Range: 1.863
Area, Height
Time Height Area Area%
4.08 1862314 568792.81 99.41
5.21 9568 3377.50 0.59
4.08
568792.8
1862314
5.21
3377.5
9568
Chiral
AU
1.5
1.0
5.0e-1
0.0
-0.00
2.00
4.00
6.00
8.00
10.00

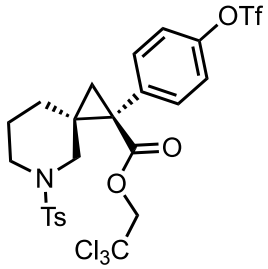

Compound 21

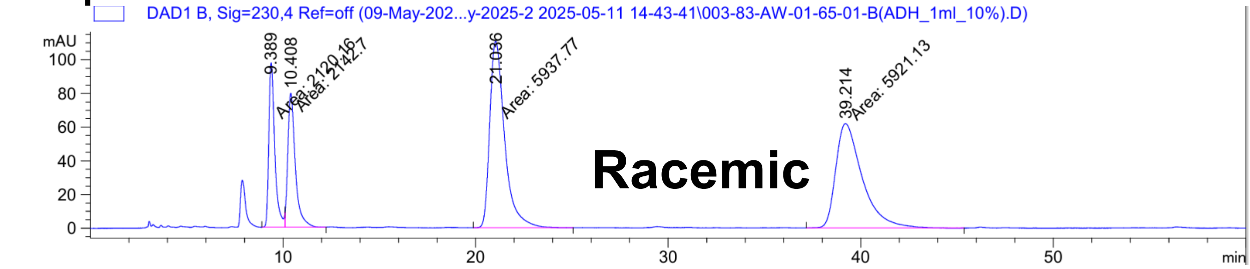

Signal 2: DAD1 B, Sig=230,4 Ref=off

Peak #	RetTime [min]	Type	Width [min]	Area [mAU*s]	Height [mAU]	Area %
1	9.389	MF	0.3625	2120.15527	97.49049	13.1509
2	10.408	FM	0.4495	2142.69580	79.44405	13.2907
3	21.036	MM	0.8945	5937.77002	110.63117	36.8308
4	39.214	MM	1.5935	5921.12891	61.93156	36.7276

Totals : 1.61218e4 349.49727

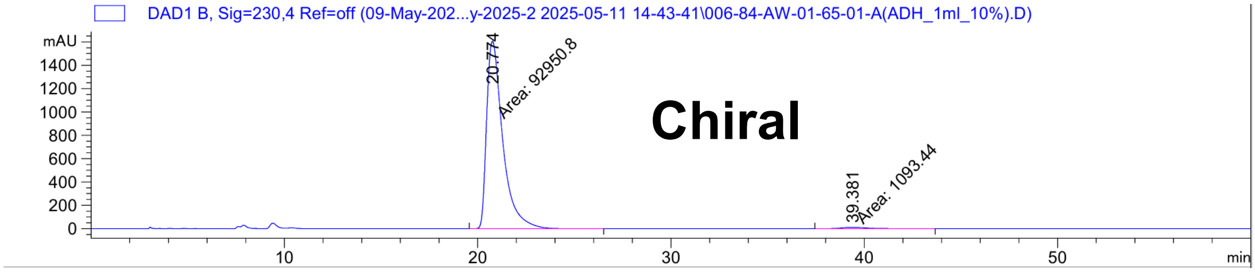

Signal 2: DAD1 B, Sig=230,4 Ref=off

Peak #	RetTime [min]	Type	Width [min]	Area [mAU*s]	Height [mAU]	Area %
1	20.774	MM	0.9645	9.29508e4	1606.14368	98.8373
2	39.381	MM	1.5343	1093.43835	11.87756	1.1627

Totals : 9.40443e4 1618.02123

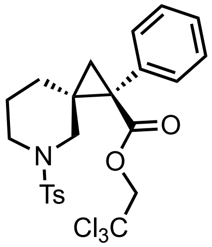

Compound 22

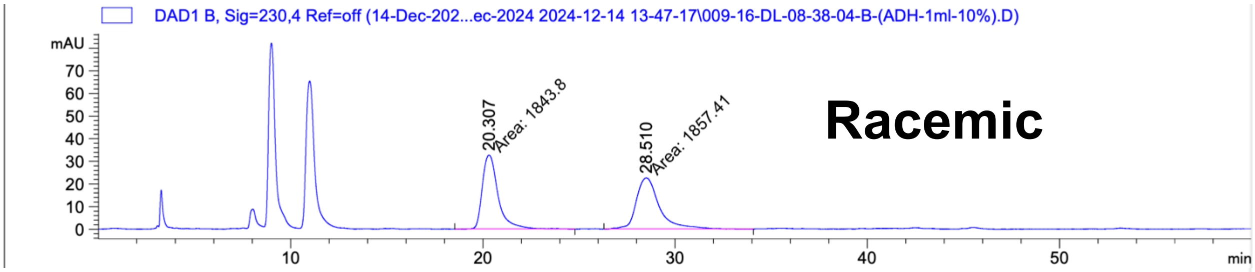

Signal 2: DAD1 B, Sig=230,4 Ref=off

Peak #	RetTime [min]	Type	Width [min]	Area [mAU*s]	Height [mAU]	Area %
1	20.307	MM	0.9422	1843.80420	32.61585	49.8162
2	28.510	MM	1.3755	1857.40833	22.50531	50.1838
Totals :				3701.21252	55.12116	

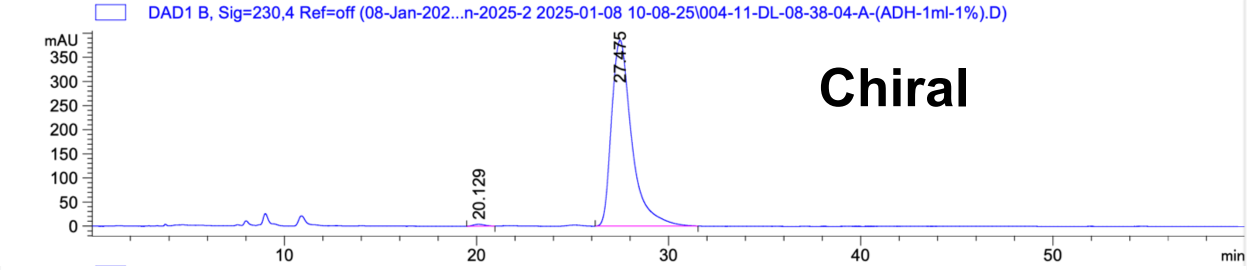

Signal 2: DAD1 B, Sig=230,4 Ref=off

Peak #	RetTime [min]	Type	Width [min]	Area [mAU*s]	Height [mAU]	Area %
1	20.129	BB	0.4672	152.31375	3.82389	0.5353
2	27.475	BB	0.8624	2.83015e4	385.79425	99.4647
Totals :				2.84538e4	389.61814	

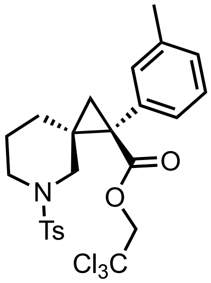

Compound 23

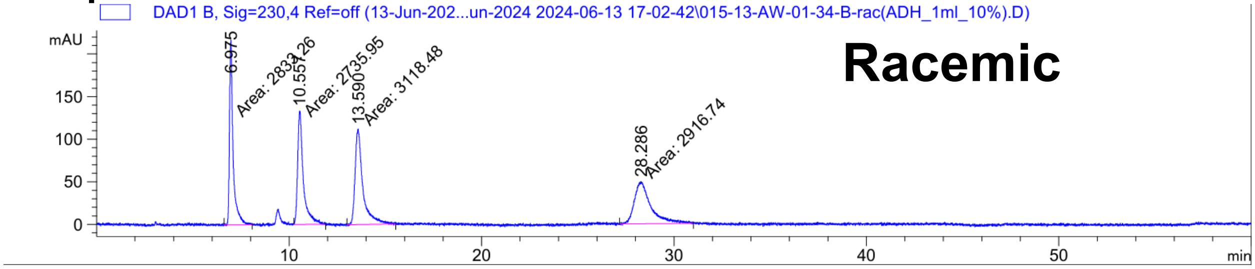


```
Signal 2: DAD1 B, Sig=230,4 Ref=off

Peak RetTime Type  Width     Area       Height      Area
  #   [min]        [min]   [mAU*s]      [mAU]        %
----|-------|----|-------|----------|----------|--------|
   1   6.975 MM    0.2174 2833.26294  217.18852  24.4153
   2  10.557 MM    0.3422 2735.95483  133.24054  23.5768
   3  13.590 MM    0.4632 3118.48145  112.21455  26.8732
   4  28.286 MM    0.9771 2916.74219   49.75204  25.1347

Totals :                  1.16044e4   512.39565
```

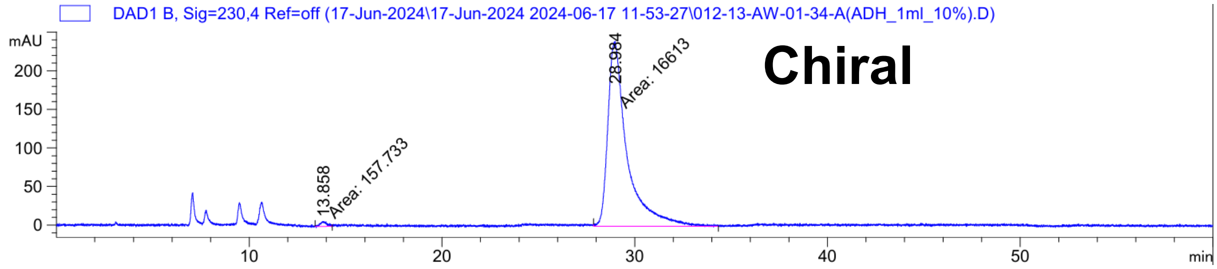


```
Signal 2: DAD1 B, Sig=230,4 Ref=off

Peak RetTime Type  Width     Area       Height      Area
  #   [min]        [min]   [mAU*s]      [mAU]        %
----|-------|----|-------|----------|----------|--------|
   1  13.858 MM    0.3966  157.73332    6.62812   0.9405
   2  28.984 MM    1.1533 1.66130e4   240.07753  99.0595

Totals :                  1.67708e4   246.70565
```

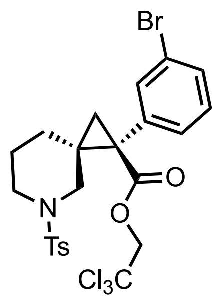

Compound 24

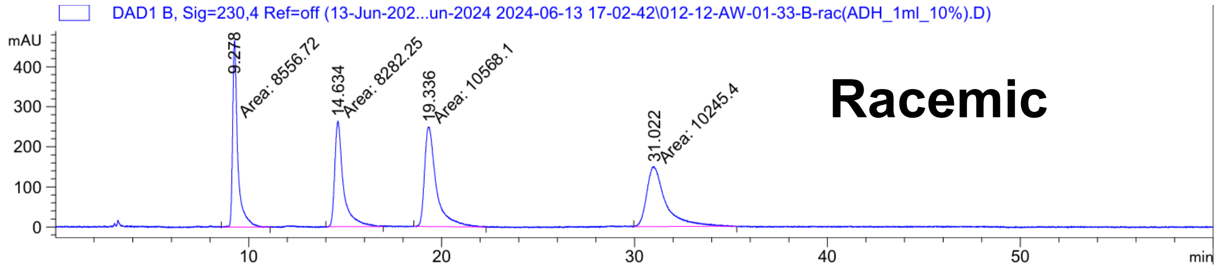

Signal 2: DAD1 B, Sig=230,4 Ref=off

Peak #	RetTime [min]	Type	Width [min]	Area [mAU*s]	Height [mAU]	Area %
1	9.278	MM	0.3065	8556.71680	465.34409	22.7255
2	14.634	MM	0.5231	8282.24707	263.89575	21.9966
3	19.336	MM	0.7058	1.05681e4	249.54501	28.0674
4	31.022	MM	1.1378	1.02454e4	150.07767	27.2105

Totals : 3.76525e4 1128.86252

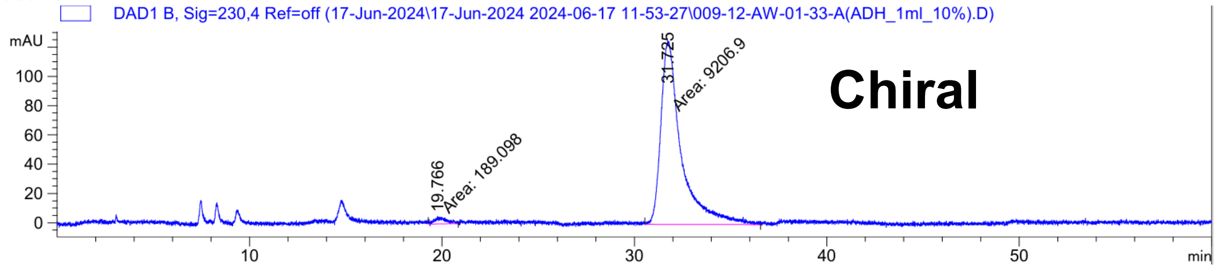

Signal 2: DAD1 B, Sig=230,4 Ref=off

Peak #	RetTime [min]	Type	Width [min]	Area [mAU*s]	Height [mAU]	Area %
1	19.766	MM	0.6149	189.09784	5.12543	2.0125
2	31.725	MM	1.2239	9206.90234	125.37157	97.9875

Totals : 9396.00018 130.49701

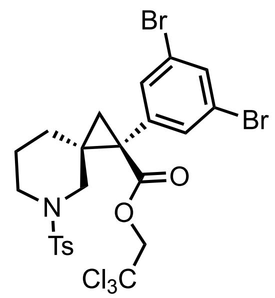

Compound 25

DL08_38_01_B_P8B1f Sm (Mn, 2x3)

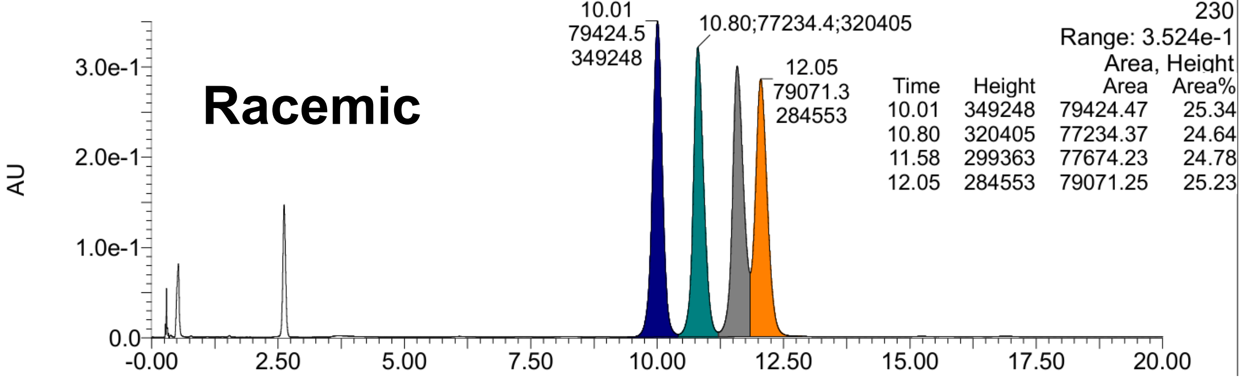

Time	Height	Area	Area%
10.01	349248	79424.47	25.34
10.80	320405	77234.37	24.64
11.58	299363	77674.23	24.78
12.05	284553	79071.25	25.23

DL08_38_01_A_P8B1ab Sm (Mn, 2x3)

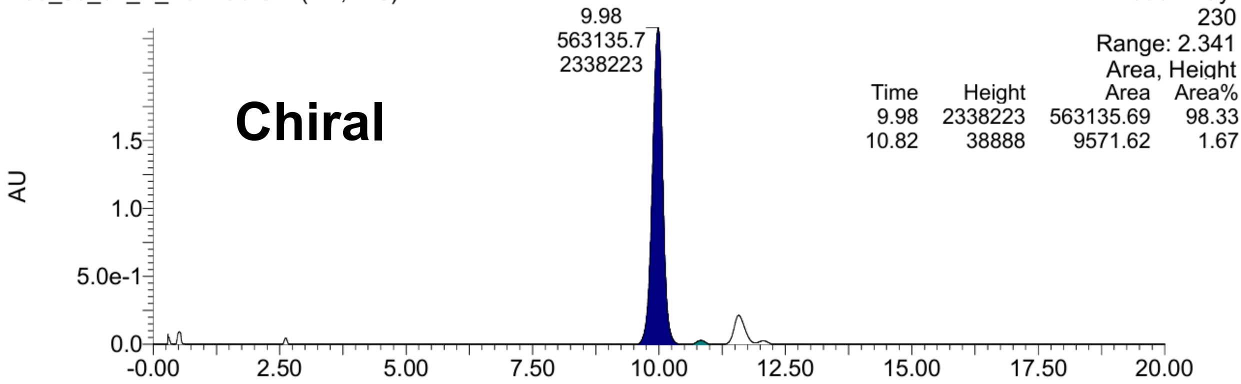

Time	Height	Area	Area%
9.98	2338223	563135.69	98.33
10.82	38888	9571.62	1.67

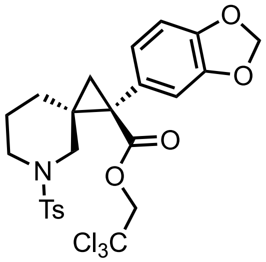

Compound 26

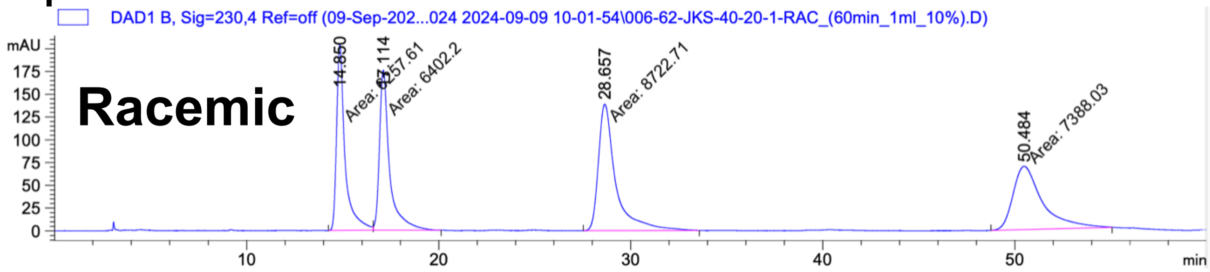

Signal 2: DAD1 B, Sig=230,4 Ref=off

Peak #	RetTime [min]	Type	Width [min]	Area [mAU*s]	Height [mAU]	Area %
1	14.850	MF	0.5124	6257.60889	203.52505	21.7500
2	17.114	FM	0.6071	6402.19727	175.76636	22.2526
3	28.657	MM	1.0460	8722.71484	138.98969	30.3182
4	50.484	MM	1.7739	7388.03027	69.41290	25.6791

Totals : 2.87706e4 587.69400

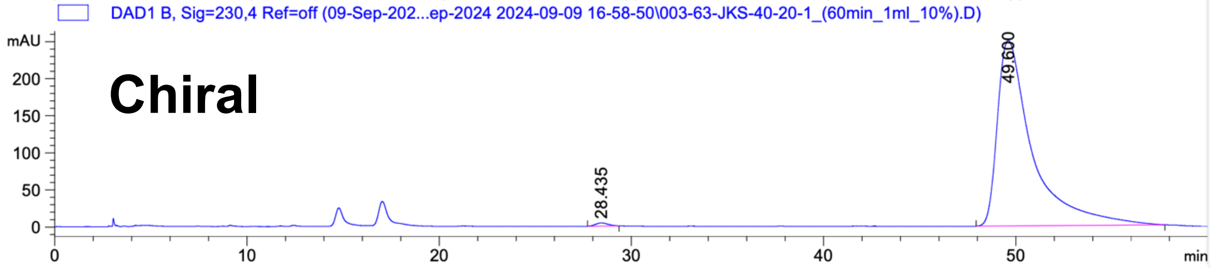

Signal 2: DAD1 B, Sig=230,4 Ref=off

Peak #	RetTime [min]	Type	Width [min]	Area [mAU*s]	Height [mAU]	Area %
1	28.435	BB	0.5279	186.83641	4.15263	0.5858
2	49.600	BB	1.4802	3.17083e4	250.16870	99.4142

Totals : 3.18951e4 254.32133

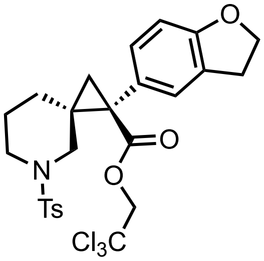

Compound 27

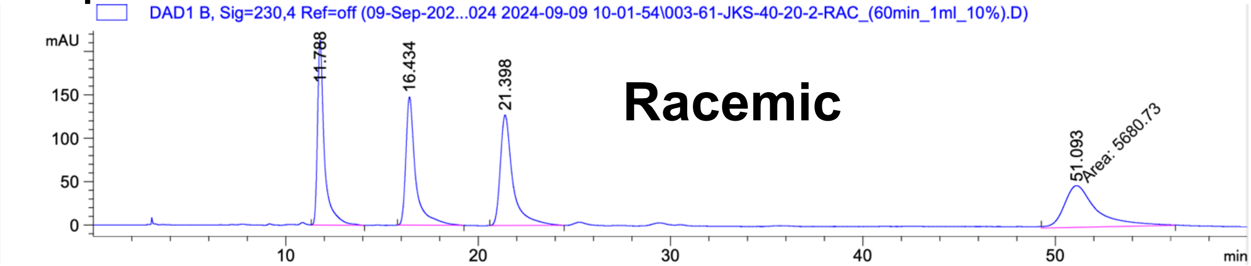

Signal 2: DAD1 B, Sig=230,4 Ref=off

Peak #	RetTime [min]	Type	Width [min]	Area [mAU*s]	Height [mAU]	Area %
1	11.788	BB	0.3573	5312.70117	212.81085	24.1626
2	16.434	BB	0.4837	5223.51416	147.66246	23.7569
3	21.398	BB	0.6140	5770.38525	127.26087	26.2441
4	51.093	MM	1.9687	5680.73047	48.09103	25.8364
Totals :				2.19873e4	535.82522	

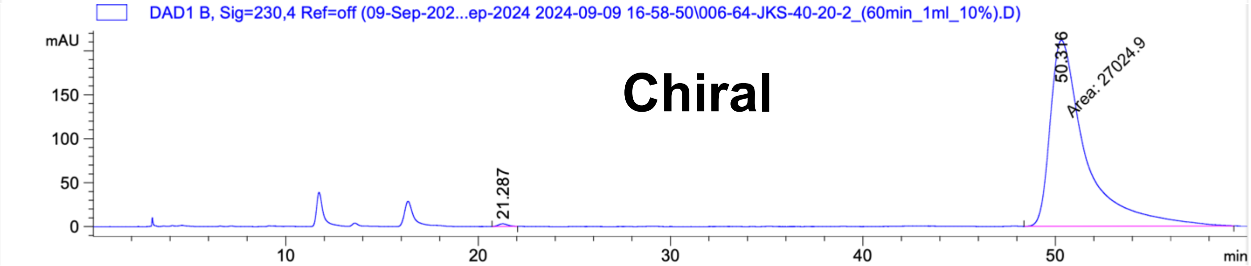

Signal 2: DAD1 B, Sig=230,4 Ref=off

Peak #	RetTime [min]	Type	Width [min]	Area [mAU*s]	Height [mAU]	Area %
1	21.287	BB	0.3950	100.09361	2.96406	0.3690
2	50.316	MM	2.1288	2.70249e4	211.58566	99.6310
Totals :				2.71250e4	214.54972	

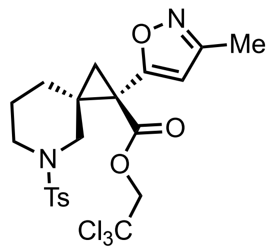

Compound 28

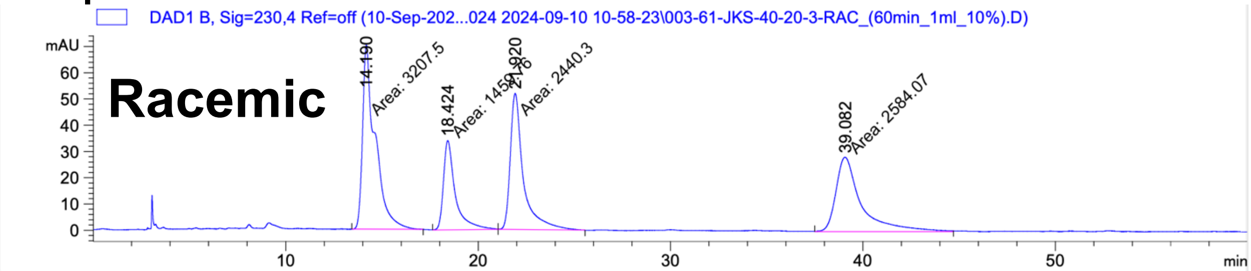


```
Signal 2: DAD1 B, Sig=230,4 Ref=off
```

Peak #	RetTime [min]	Type	Width [min]	Area [mAU*s]	Height [mAU]	Area %
1	14.190	MM	0.7616	3207.50220	70.19487	33.0956
2	18.424	MM	0.7152	1459.75647	34.01768	15.0620
3	21.920	MM	0.7842	2440.30127	51.86623	25.1795
4	39.082	MM	1.5211	2584.06812	28.31438	26.6629
Totals :				9691.62805	184.39316	

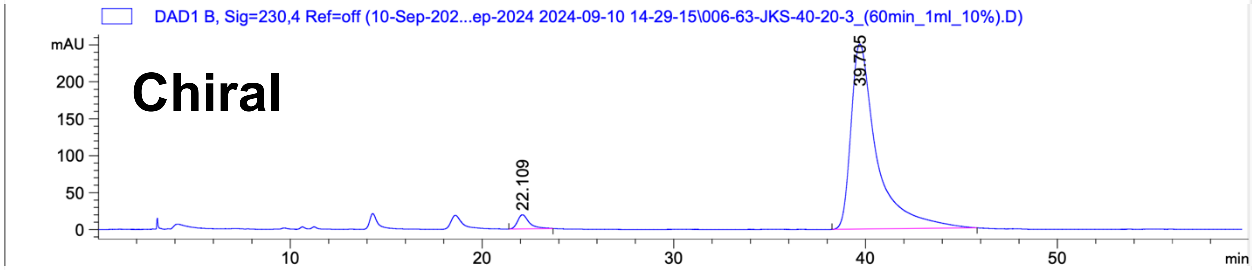


```
Signal 2: DAD1 B, Sig=230,4 Ref=off
```

Peak #	RetTime [min]	Type	Width [min]	Area [mAU*s]	Height [mAU]	Area %
1	22.109	BB	0.4918	801.14905	19.12359	3.2992
2	39.705	BB	1.0970	2.34818e4	250.76302	96.7008
Totals :				2.42829e4	269.88661	

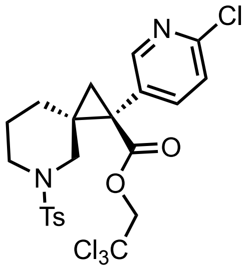

Compound 29

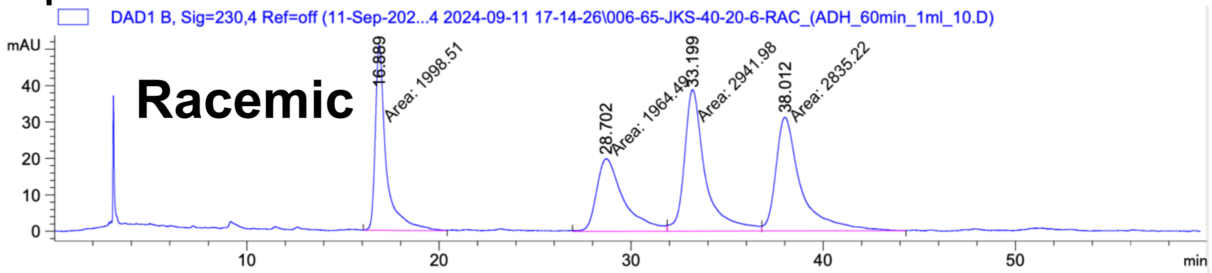

Signal 2: DAD1 B, Sig=230,4 Ref=off

Peak #	RetTime [min]	Type	Width [min]	Area [mAU*s]	Height [mAU]	Area %
1	16.889	MM	0.6540	1998.50598	50.92659	20.5181
2	28.702	MF	1.6441	1964.49316	19.91454	20.1689
3	33.199	MF	1.2619	2941.98315	38.85551	30.2045
4	38.012	FM	1.5153	2835.21680	31.18527	29.1084

Totals : 9740.19910 140.88191

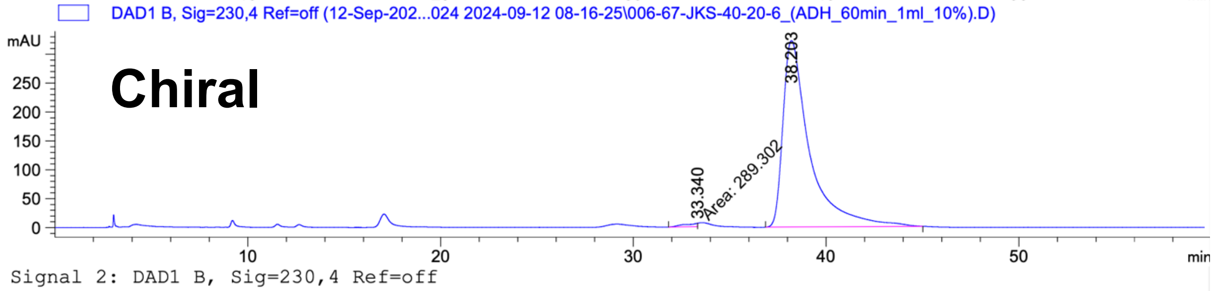

Signal 2: DAD1 B, Sig=230,4 Ref=off

Peak #	RetTime [min]	Type	Width [min]	Area [mAU*s]	Height [mAU]	Area %
1	33.340	MM	0.8001	289.30200	6.02654	0.9205
2	38.203	BB	1.1296	3.11380e4	322.58481	99.0795

Totals : 3.14273e4 328.61135

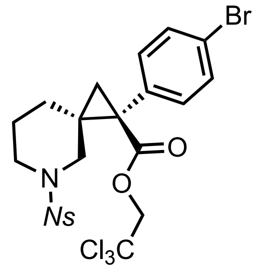
Br
O
N
Ns
O
Cl3C

Compound 30

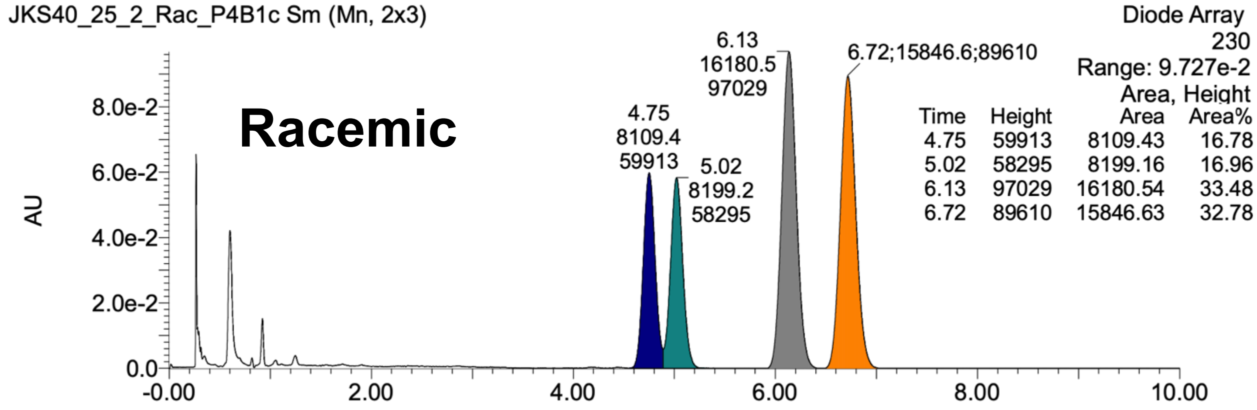
JKS40_25_2_Rac_P4B1c Sm (Mn, 2x3)
Diode Array
230
Range: 9.727e-2
Area, Height
Racemic
AU
Time Height Area Area%
4.75 59913 8109.43 16.78
5.02 58295 8199.16 16.96
6.13 97029 16180.54 33.48
6.72 89610 15846.63 32.78
4.75 8109.4 59913
5.02 8199.2 58295
6.13 16180.5 97029
6.72;15846.6;89610

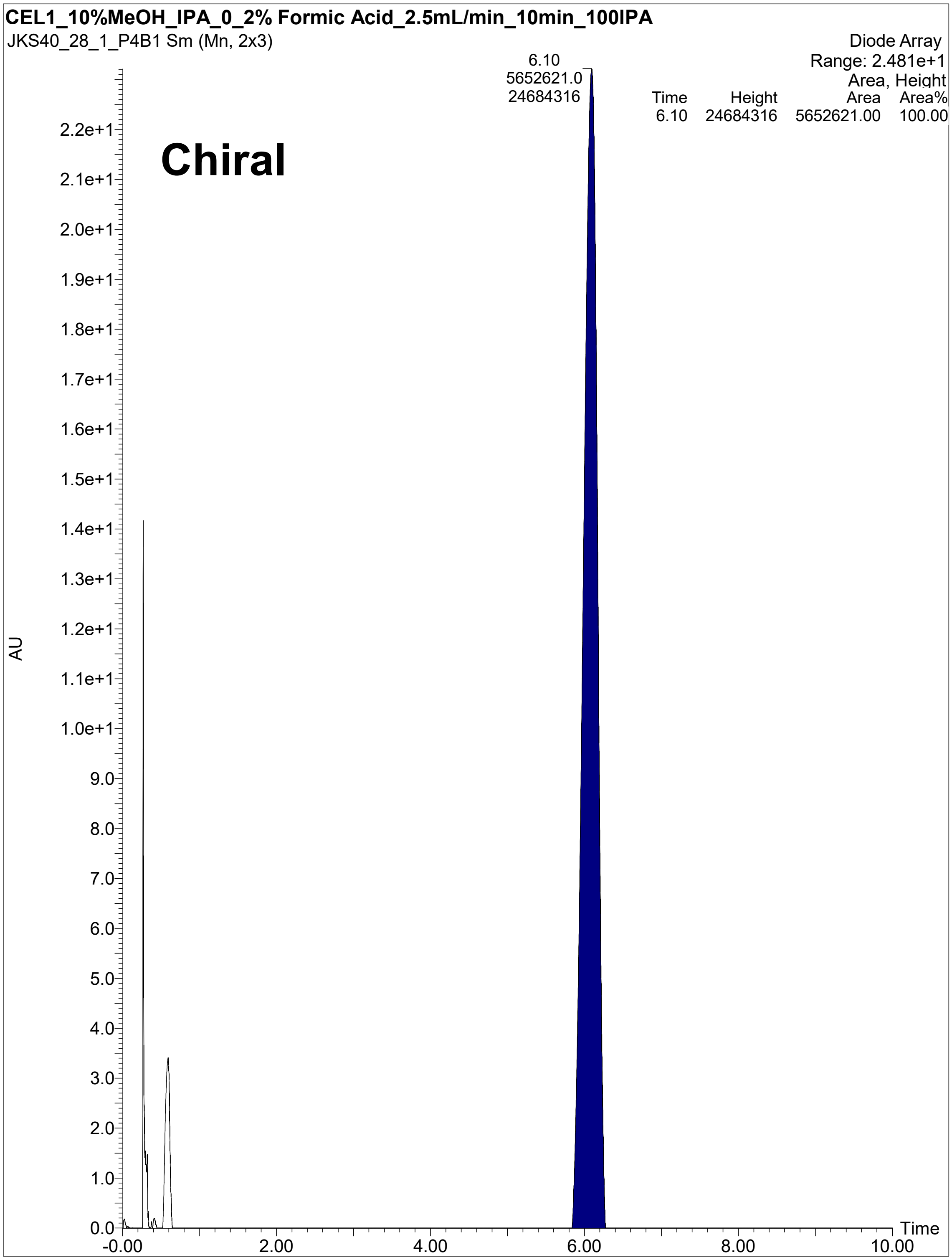
CEL1_10%MeOH_IPA_0_2% Formic Acid_2.5mL/min_10min_100IPA
JKS40_28_1_P4B1 Sm (Mn, 2x3)
Diode Array
Range: 2.481e+1
Area, Height
Time Height Area Area%
6.10 24684316 5652621.00 100.00
6.10
5652621.0
24684316
Chiral
AU
Time

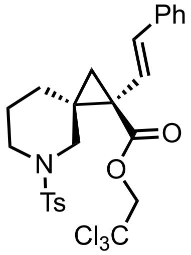

Compound 31

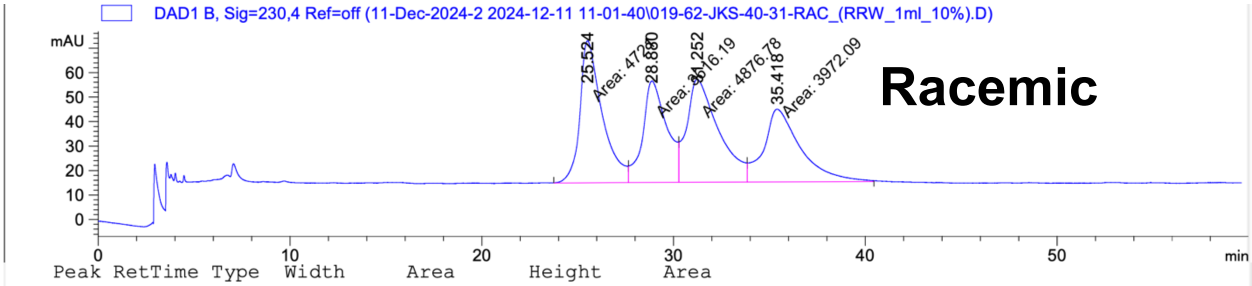

Peak #	RetTime [min]	Type	Width [min]	Area [mAU*s]	Height [mAU]	Area %
1	25.524	MF	1.3560	4721.00342	58.02432	27.4699
2	28.880	MF	1.4529	3616.19043	41.48373	21.0414
3	31.252	MF	1.9341	4876.78320	42.02508	28.3764
4	35.418	FM	2.2281	3972.08789	29.71251	23.1123

Totals : 1.71861e4 171.24564

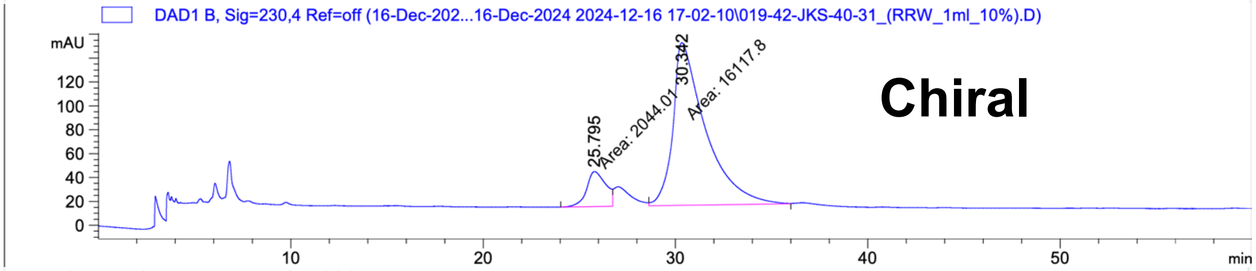

Signal 2: DAD1 B, Sig=230,4 Ref=off

Peak #	RetTime [min]	Type	Width [min]	Area [mAU*s]	Height [mAU]	Area %
1	25.795	MF	1.1652	2044.00745	29.23696	11.2544
2	30.342	FM	1.9708	1.61178e4	136.30537	88.7456

Totals : 1.81618e4 165.54234

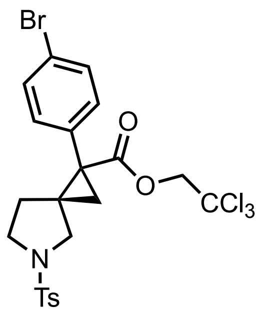
Br
O
O
CCl3
N
Ts

Compound 32

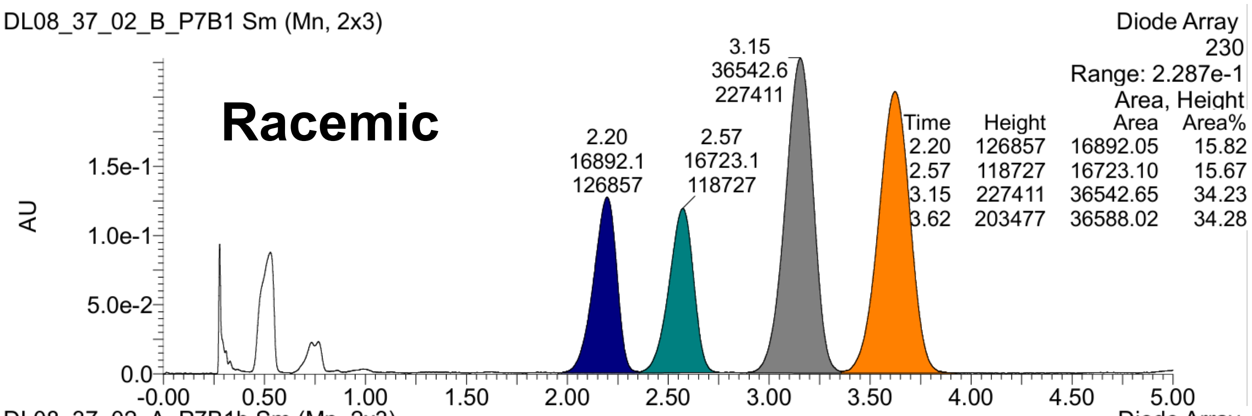
DL08_37_02_B_P7B1 Sm (Mn, 2x3)
Diode Array
230
Range: 2.287e-1
Area, Height
Racemic
Time Height Area Area%
2.20 126857 16892.05 15.82
2.57 118727 16723.10 15.67
3.15 227411 36542.65 34.23
3.62 203477 36588.02 34.28
2.20
16892.1
126857
2.57
16723.1
118727
3.15
36542.6
227411
AU
1.5e-1
1.0e-1
5.0e-2
0.0
-0.00 0.50 1.00 1.50 2.00 2.50 3.00 3.50 4.00 4.50 5.00

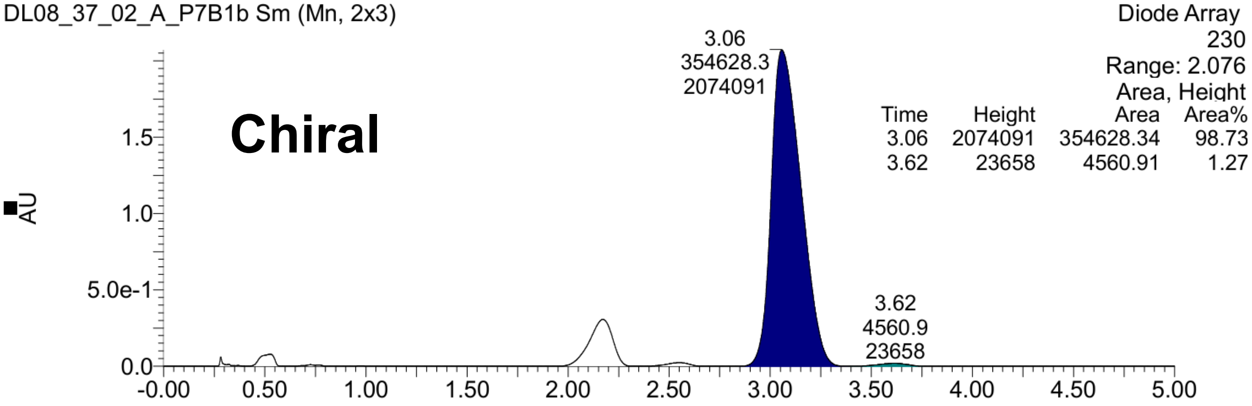
DL08_37_02_A_P7B1b Sm (Mn, 2x3)
Diode Array
230
Range: 2.076
Area, Height
Chiral
Time Height Area Area%
3.06 2074091 354628.34 98.73
3.62 23658 4560.91 1.27
3.06
354628.3
2074091
3.62
4560.9
23658
AU
1.5
1.0
5.0e-1
0.0
-0.00 0.50 1.00 1.50 2.00 2.50 3.00 3.50 4.00 4.50 5.00

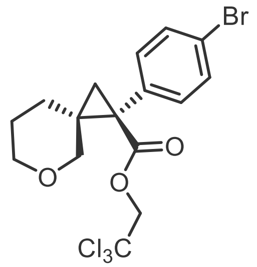

Compound 34

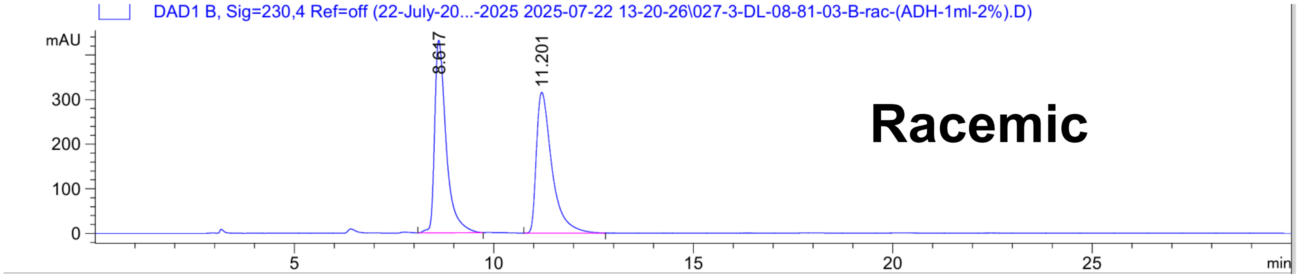

Signal 2: DAD1 B, Sig=230,4 Ref=off

Peak #	RetTime [min]	Type	Width [min]	Area [mAU*s]	Height [mAU]	Area %
1	8.617	BB	0.2946	8642.49121	431.60425	50.3052
2	11.201	VV R	0.3821	8537.63965	315.63525	49.6948

Totals : 1.71801e4 747.23950

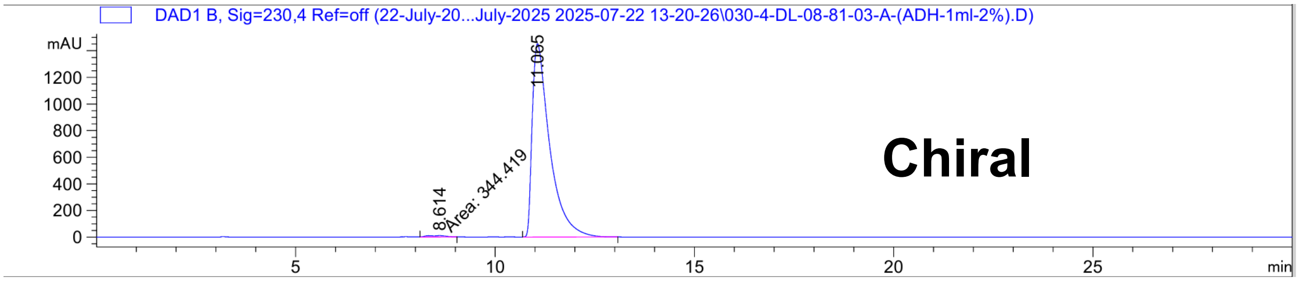

Signal 2: DAD1 B, Sig=230,4 Ref=off

Peak #	RetTime [min]	Type	Width [min]	Area [mAU*s]	Height [mAU]	Area %
1	8.614	MM	0.5481	344.41895	10.47309	0.7701
2	11.065	BV R	0.3616	4.43771e4	1452.05554	99.2299

Totals : 4.47215e4 1462.52863

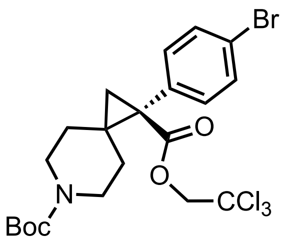
Br
O
O
CCl3
N
Boc

Compound 35

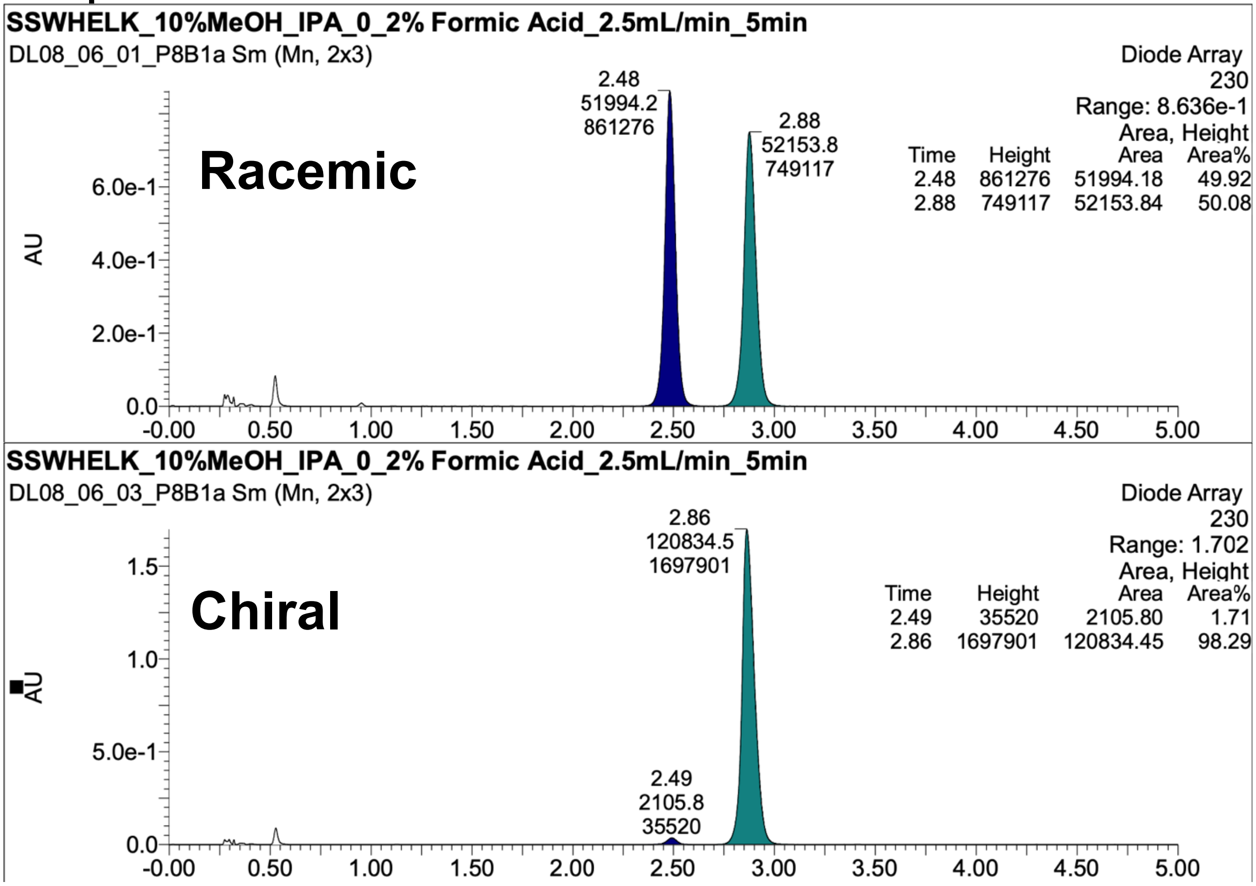
SSWHELK_10%MeOH_IPA_0_2% Formic Acid_2.5mL/min_5min
DL08_06_01_P8B1a Sm (Mn, 2x3)
Diode Array
230
Range: 8.636e-1
Area, Height
Racemic
2.48
51994.2
861276
2.88
52153.8
749117
Time Height Area Area%
2.48 861276 51994.18 49.92
2.88 749117 52153.84 50.08
AU
6.0e-1
4.0e-1
2.0e-1
0.0
-0.00 0.50 1.00 1.50 2.00 2.50 3.00 3.50 4.00 4.50 5.00
SSWHELK_10%MeOH_IPA_0_2% Formic Acid_2.5mL/min_5min
DL08_06_03_P8B1a Sm (Mn, 2x3)
Diode Array
230
Range: 1.702
Area, Height
Chiral
2.86
120834.5
1697901
Time Height Area Area%
2.49 35520 2105.80 1.71
2.86 1697901 120834.45 98.29
2.49
2105.8
35520
AU
1.5
1.0
5.0e-1
0.0
-0.00 0.50 1.00 1.50 2.00 2.50 3.00 3.50 4.00 4.50 5.00

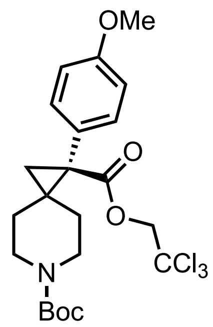

Compound 36

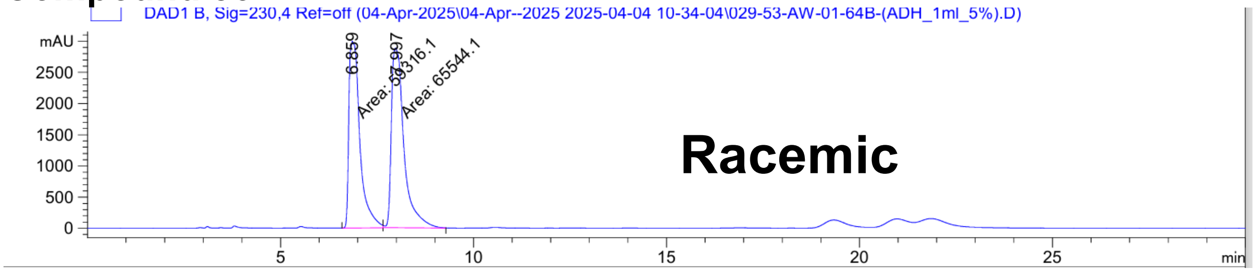

Signal 2: DAD1 B, Sig=230,4 Ref=off

Peak #	RetTime [min]	Type	Width [min]	Area [mAU*s]	Height [mAU]	Area %
1	6.859	MM	0.3299	5.93161e4	2996.92871	47.5060
2	7.997	MM	0.3790	6.55441e4	2882.43848	52.4940

Totals : 1.24860e5 5879.36719

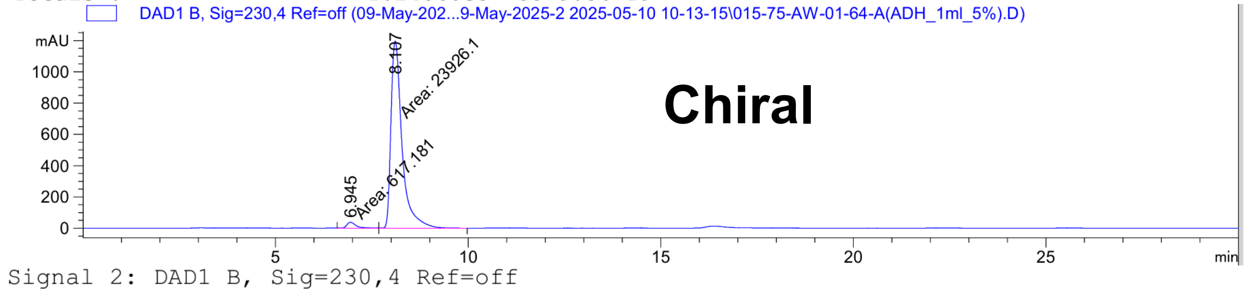

Signal 2: DAD1 B, Sig=230,4 Ref=off

Peak #	RetTime [min]	Type	Width [min]	Area [mAU*s]	Height [mAU]	Area %
1	6.945	MF	0.2744	617.18060	37.49046	2.5147
2	8.107	FM	0.3335	2.39261e4	1195.59338	97.4853

Totals : 2.45432e4 1233.08384

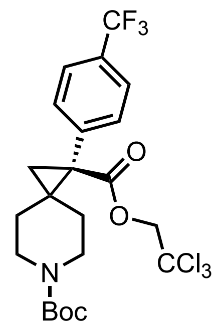

Compound 37

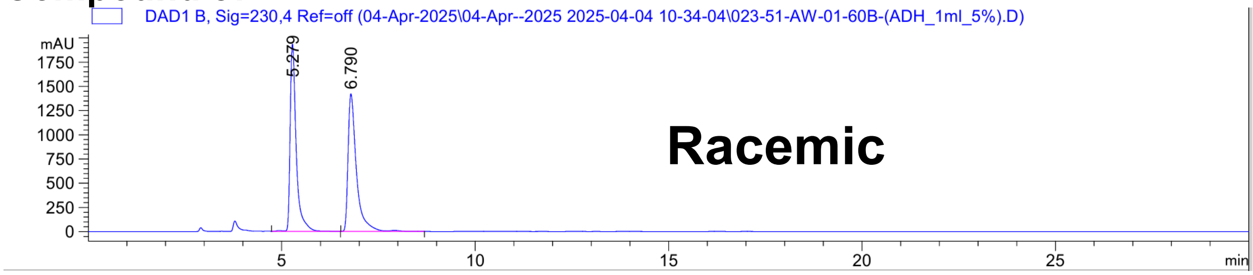

Signal 2: DAD1 B, Sig=230,4 Ref=off

Peak #	RetTime [min]	Type	Width [min]	Area [mAU*s]	Height [mAU]	Area %
1	5.279	VB R	0.1641	2.10609e4	1933.41809	49.6102
2	6.790	BV R	0.2191	2.13919e4	1420.19177	50.3898

Totals : 4.24528e4 3353.60986

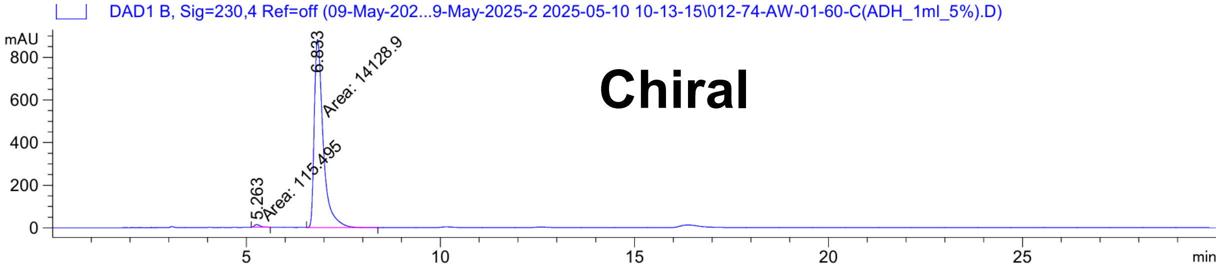

Signal 2: DAD1 B, Sig=230,4 Ref=off

Peak #	RetTime [min]	Type	Width [min]	Area [mAU*s]	Height [mAU]	Area %
1	5.263	MM	0.1576	115.49541	12.21349	0.8108
2	6.833	MM	0.2668	1.41289e4	882.55029	99.1892

Totals : 1.42444e4 894.76379

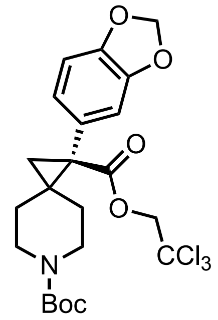

Compound 38

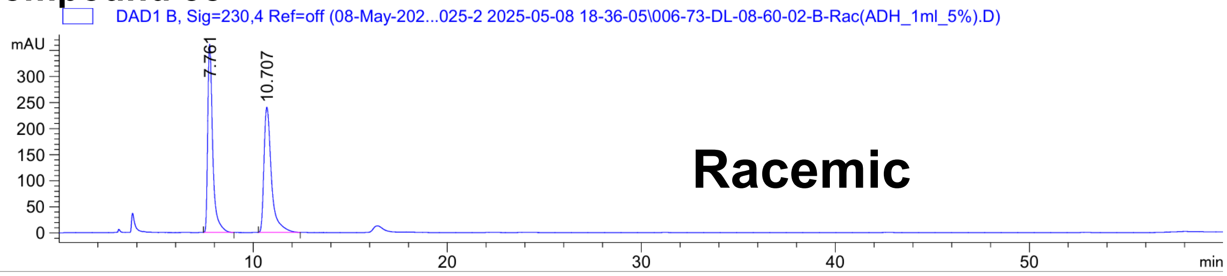

Signal 2: DAD1 B, Sig=230,4 Ref=off

Peak #	RetTime [min]	Type	Width [min]	Area [mAU*s]	Height [mAU]	Area %
1	7.761	BB	0.2704	6426.94092	360.73853	50.0387
2	10.707	BV R	0.3889	6417.00586	240.01241	49.9613
Totals :				1.28439e4	600.75093	

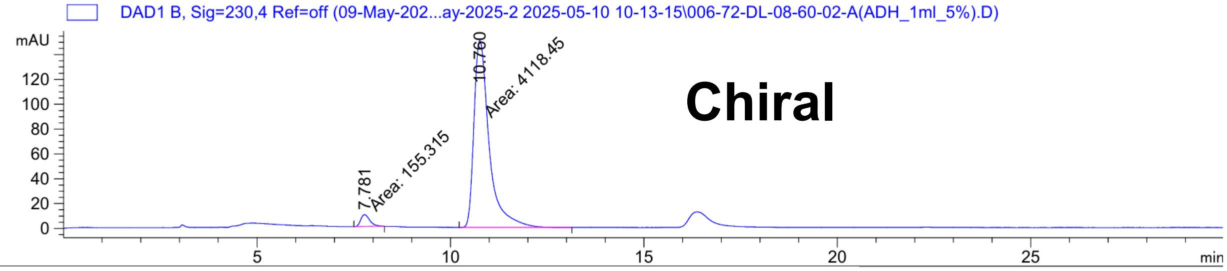

Signal 2: DAD1 B, Sig=230,4 Ref=off

Peak #	RetTime [min]	Type	Width [min]	Area [mAU*s]	Height [mAU]	Area %
1	7.781	MM	0.2707	155.31496	9.56109	3.6342
2	10.760	MM	0.4560	4118.44629	150.51447	96.3658
Totals :				4273.76125	160.07556	

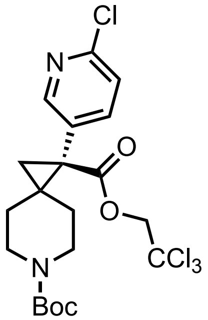

Compound 39

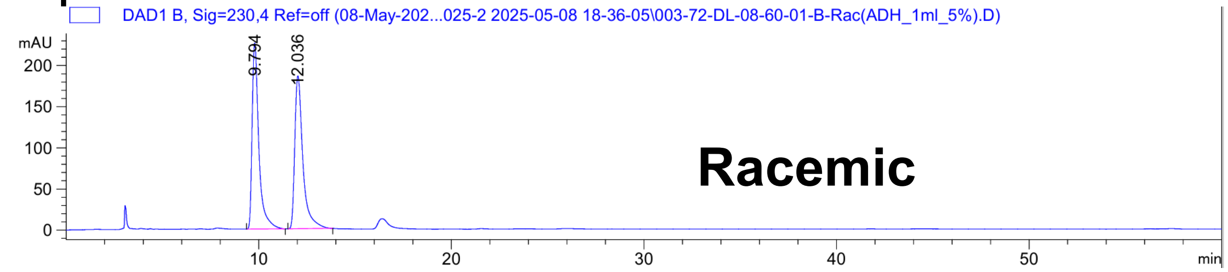

Signal 2: DAD1 B, Sig=230,4 Ref=off

Peak #	RetTime [min]	Type	Width [min]	Area [mAU*s]	Height [mAU]	Area %
1	9.794	BB	0.3561	5477.78760	226.11772	50.2705
2	12.036	BB	0.4073	5418.84424	185.80373	49.7295
Totals :				1.08966e4	411.92145	

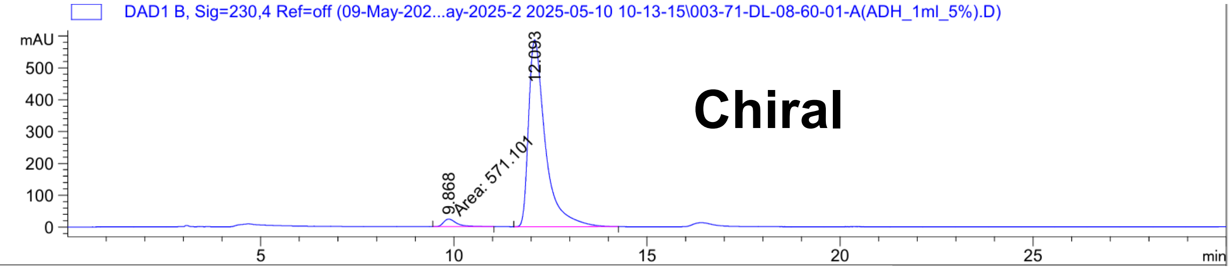

Signal 2: DAD1 B, Sig=230,4 Ref=off

Peak #	RetTime [min]	Type	Width [min]	Area [mAU*s]	Height [mAU]	Area %
1	9.868	MM	0.4012	571.10120	23.72598	3.1130
2	12.093	BB	0.4369	1.77745e4	586.79553	96.8870
Totals :				1.83456e4	610.52151	

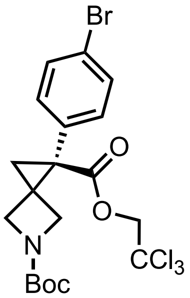
Br
O
O
N
Boc
CCl3

Compound 40

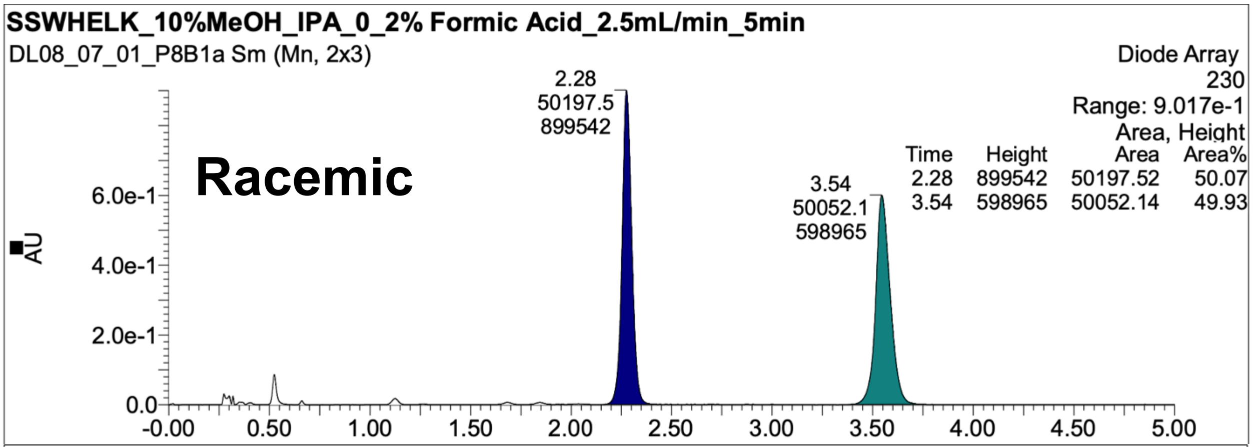
SSWHELK_10%MeOH_IPA_0_2% Formic Acid_2.5mL/min_5min
DL08_07_01_P8B1a Sm (Mn, 2x3)
Diode Array
230
Range: 9.017e-1
Area, Height
Racemic
Time Height Area Area%
2.28 899542 50197.52 50.07
3.54 598965 50052.14 49.93
2.28
50197.5
899542
3.54
50052.1
598965
AU
6.0e-1
4.0e-1
2.0e-1
0.0
-0.00 0.50 1.00 1.50 2.00 2.50 3.00 3.50 4.00 4.50 5.00

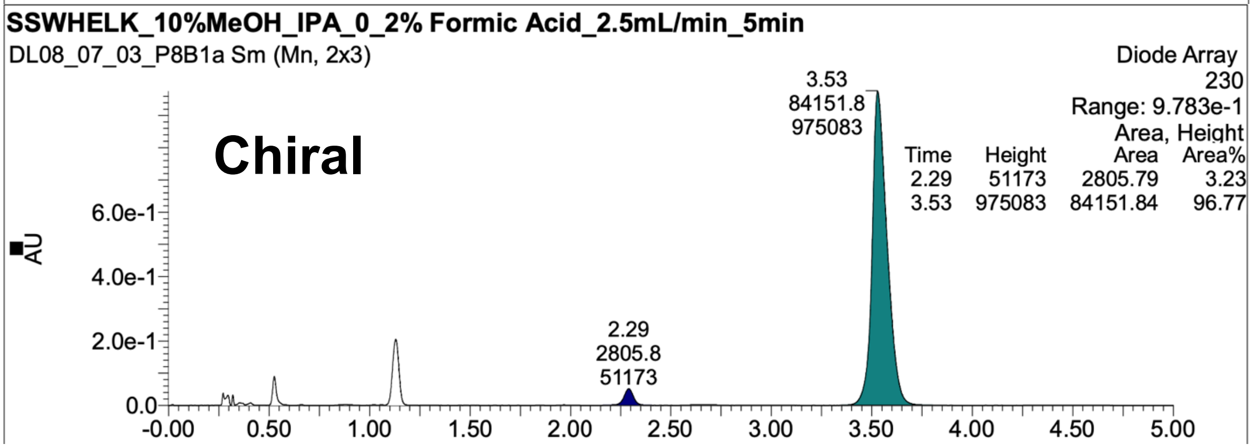
SSWHELK_10%MeOH_IPA_0_2% Formic Acid_2.5mL/min_5min
DL08_07_03_P8B1a Sm (Mn, 2x3)
Diode Array
230
Range: 9.783e-1
Area, Height
Chiral
Time Height Area Area%
2.29 51173 2805.79 3.23
3.53 975083 84151.84 96.77
3.53
84151.8
975083
2.29
2805.8
51173
AU
6.0e-1
4.0e-1
2.0e-1
0.0
-0.00 0.50 1.00 1.50 2.00 2.50 3.00 3.50 4.00 4.50 5.00

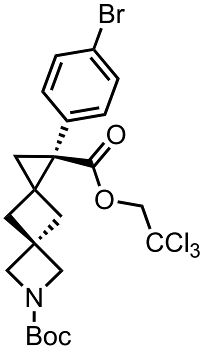

Compound 41

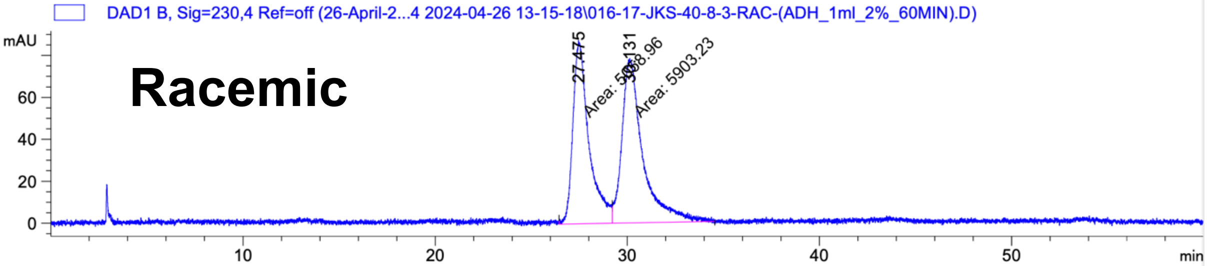

Signal 2: DAD1 B, Sig=230,4 Ref=off

Peak #	RetTime [min]	Type	Width [min]	Area [mAU*s]	Height [mAU]	Area %
1	27.475	MF	0.9633	5058.95850	87.52956	46.1492
2	30.131	FM	1.2537	5903.22852	78.47722	53.8508

Totals : 1.09622e4 166.00677

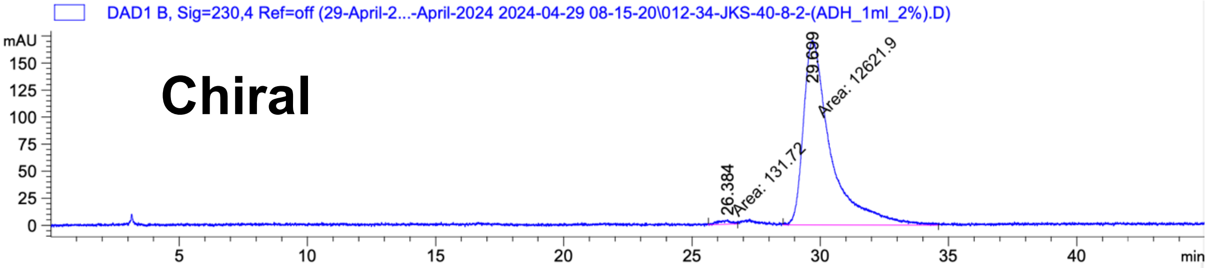

Signal 2: DAD1 B, Sig=230,4 Ref=off

Peak #	RetTime [min]	Type	Width [min]	Area [mAU*s]	Height [mAU]	Area %
1	26.384	MM	0.5105	131.71962	4.30054	1.0328
2	29.699	MM	1.2326	1.26219e4	170.67297	98.9672

Totals : 1.27536e4 174.97351

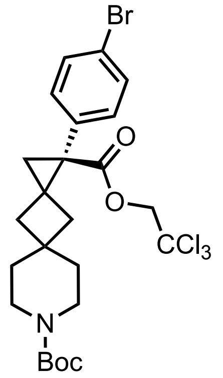
Br
O
O
CCl3
N
Boc

Compound 42

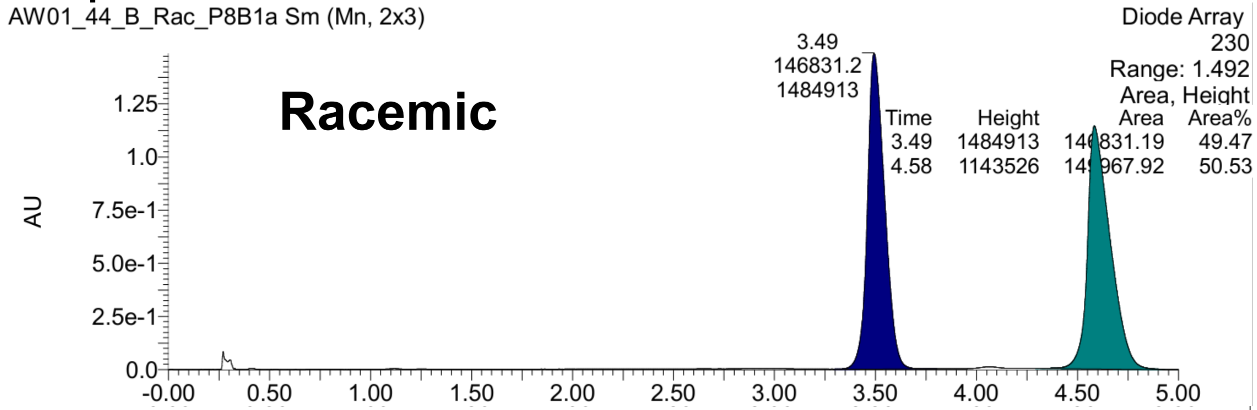
AW01_44_B_Rac_P8B1a Sm (Mn, 2x3)
Diode Array
230
Range: 1.492
Area, Height
Racemic
3.49
146831.2
1484913
Time Height Area Area%
3.49 1484913 831.19 49.47
4.58 1143526 967.92 50.53
AU
1.25
1.0
7.5e-1
5.0e-1
2.5e-1
0.0
-0.00 0.50 1.00 1.50 2.00 2.50 3.00 3.50 4.00 4.50 5.00

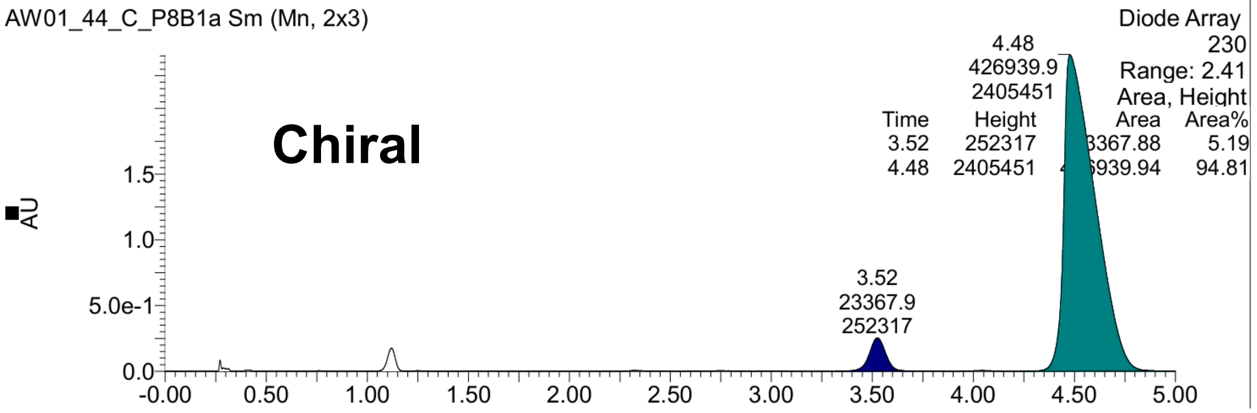
AW01_44_C_P8B1a Sm (Mn, 2x3)
Diode Array
230
Range: 2.41
Area, Height
4.48
426939.9
2405451
Chiral
Time Height Area Area%
3.52 252317 3367.88 5.19
4.48 2405451 939.94 94.81
AU
1.5
1.0
5.0e-1
0.0
3.52
23367.9
252317
-0.00 0.50 1.00 1.50 2.00 2.50 3.00 3.50 4.00 4.50 5.00

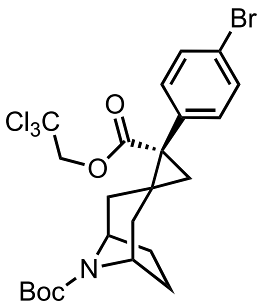

Compound 43

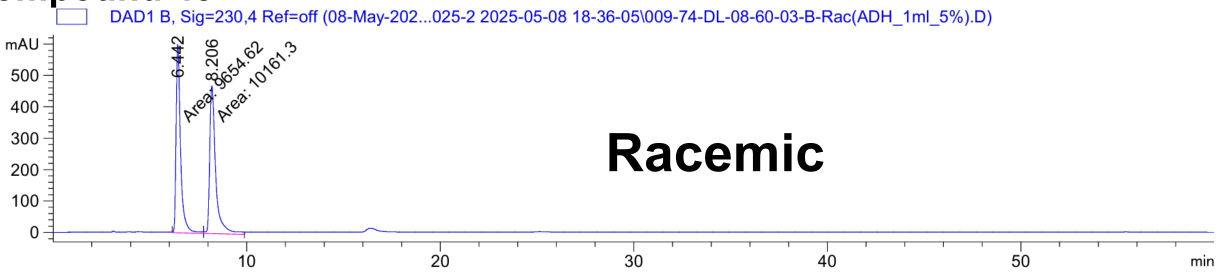

Signal 2: DAD1 B, Sig=230,4 Ref=off

Peak #	RetTime [min]	Type	Width [min]	Area [mAU*s]	Height [mAU]	Area %
1	6.442	MF	0.2685	9654.62012	599.39435	48.7216
2	8.206	FM	0.3601	1.01613e4	470.36270	51.2784
Totals :				1.98159e4	1069.75705	

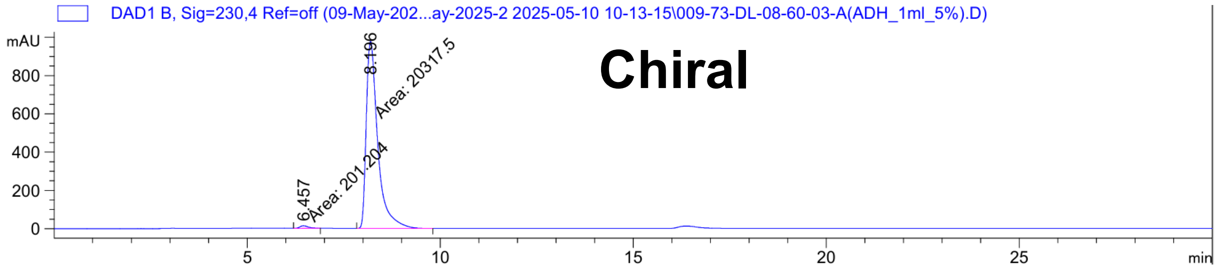

Signal 2: DAD1 B, Sig=230,4 Ref=off

Peak #	RetTime [min]	Type	Width [min]	Area [mAU*s]	Height [mAU]	Area %
1	6.457	MM	0.2507	201.20392	13.37454	0.9806
2	8.196	MM	0.3465	2.03175e4	977.17212	99.0194
Totals :				2.05187e4	990.54666	

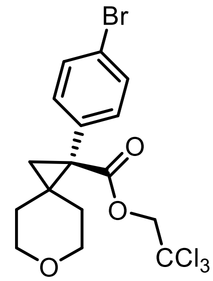

Compound 44

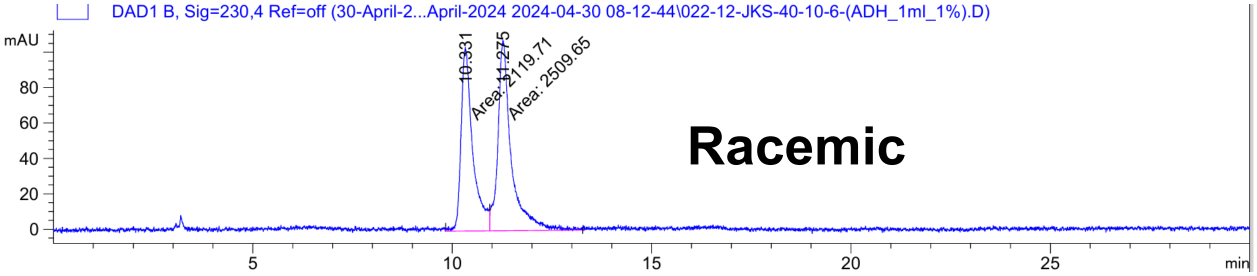

Signal 2: DAD1 B, Sig=230,4 Ref=off

Peak #	RetTime [min]	Type	Width [min]	Area [mAU*s]	Height [mAU]	Area %
1	10.331	MF	0.3404	2119.71240	103.78594	45.7884
2	11.275	FM	0.3886	2509.65112	107.62271	54.2116
Totals :				4629.36353	211.40865	

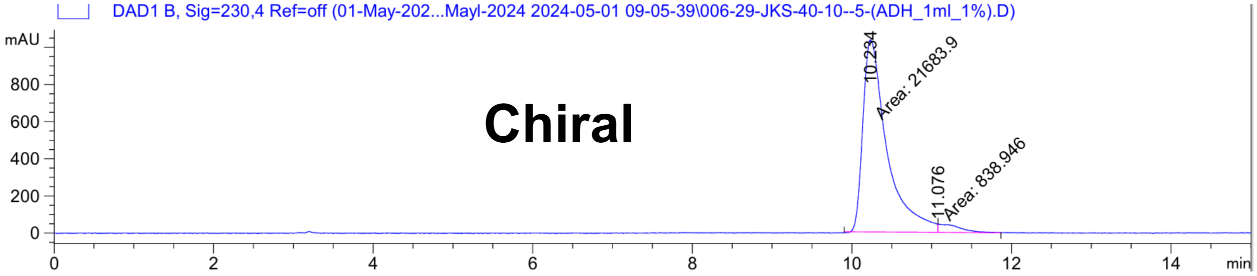

Signal 2: DAD1 B, Sig=230,4 Ref=off

Peak #	RetTime [min]	Type	Width [min]	Area [mAU*s]	Height [mAU]	Area %
1	10.234	MF	0.3490	2.16839e4	1035.54968	96.2751
2	11.076	FM	0.3145	838.94568	44.45222	3.7249
Totals :				2.25228e4	1080.00191	

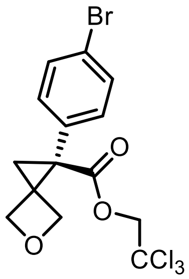

Compound 45

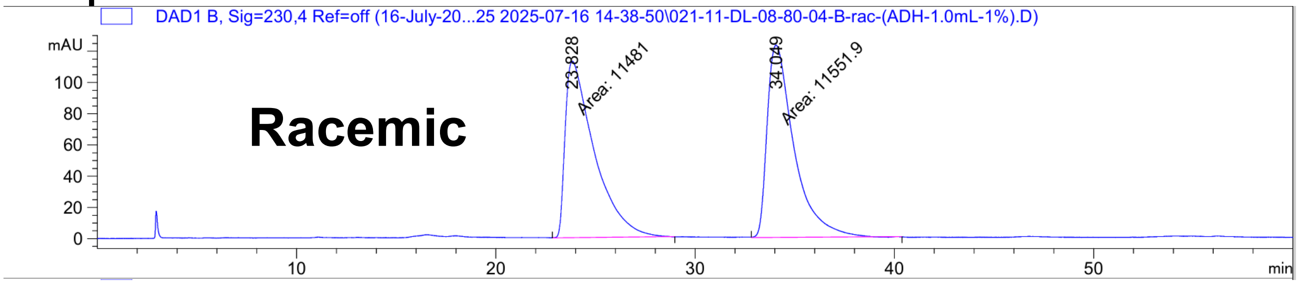

Signal 2: DAD1 B, Sig=230,4 Ref=off

Peak #	RetTime [min]	Type	Width [min]	Area [mAU*s]	Height [mAU]	Area %
1	23.828	MM	1.6925	1.14810e4	113.05989	49.8461
2	34.049	MM	1.5632	1.15519e4	123.16366	50.1539

Totals : 2.30329e4 236.22355

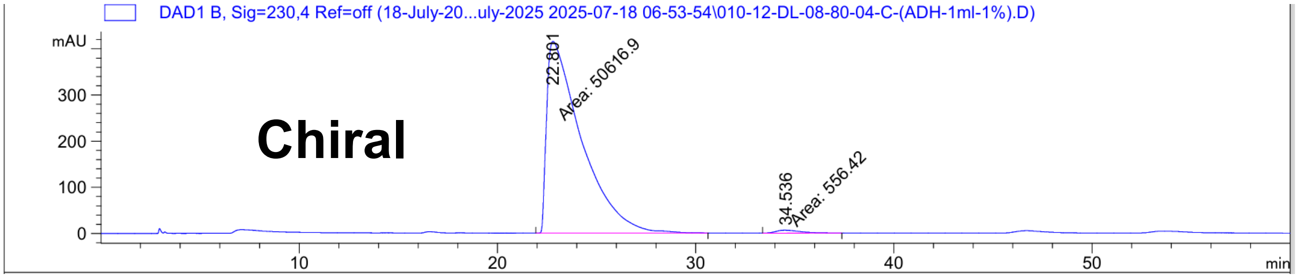

Signal 2: DAD1 B, Sig=230,4 Ref=off

Peak #	RetTime [min]	Type	Width [min]	Area [mAU*s]	Height [mAU]	Area %
1	22.801	MM	2.0320	5.06169e4	415.16367	98.9127
2	34.536	MM	1.4545	556.41992	6.37567	1.0873

Totals : 5.11733e4 421.53933

8. NMR of Novel Compounds

Compound **8b**

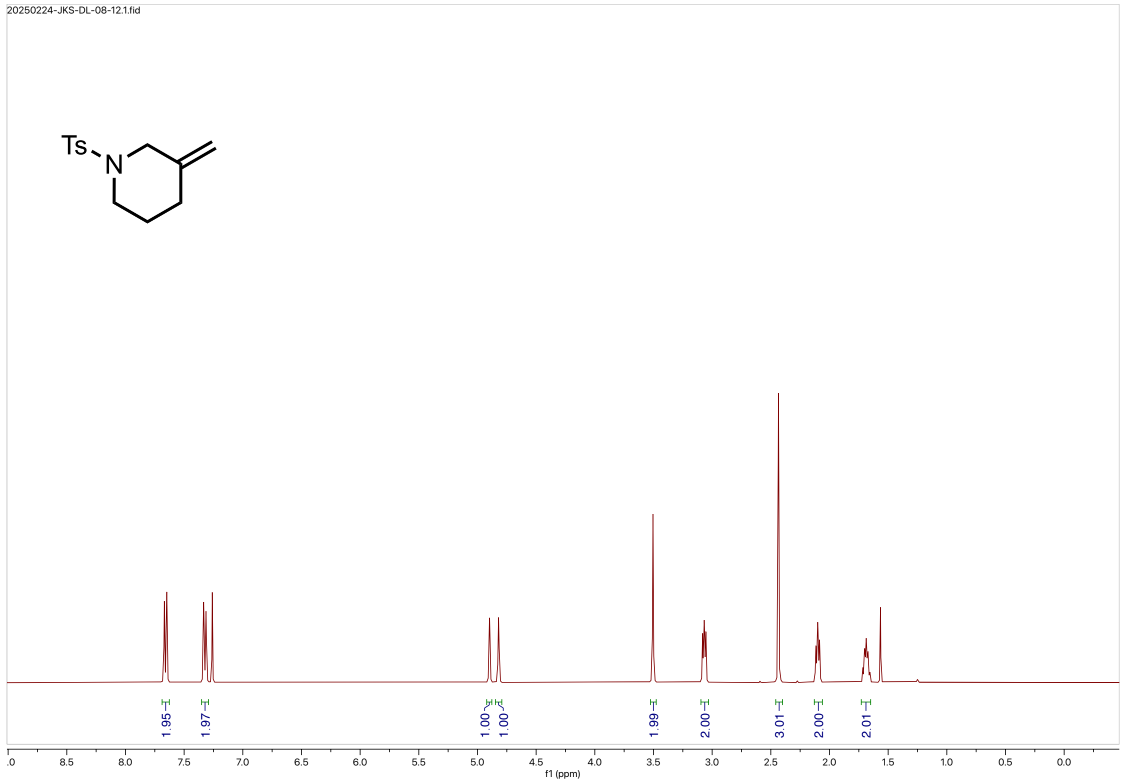

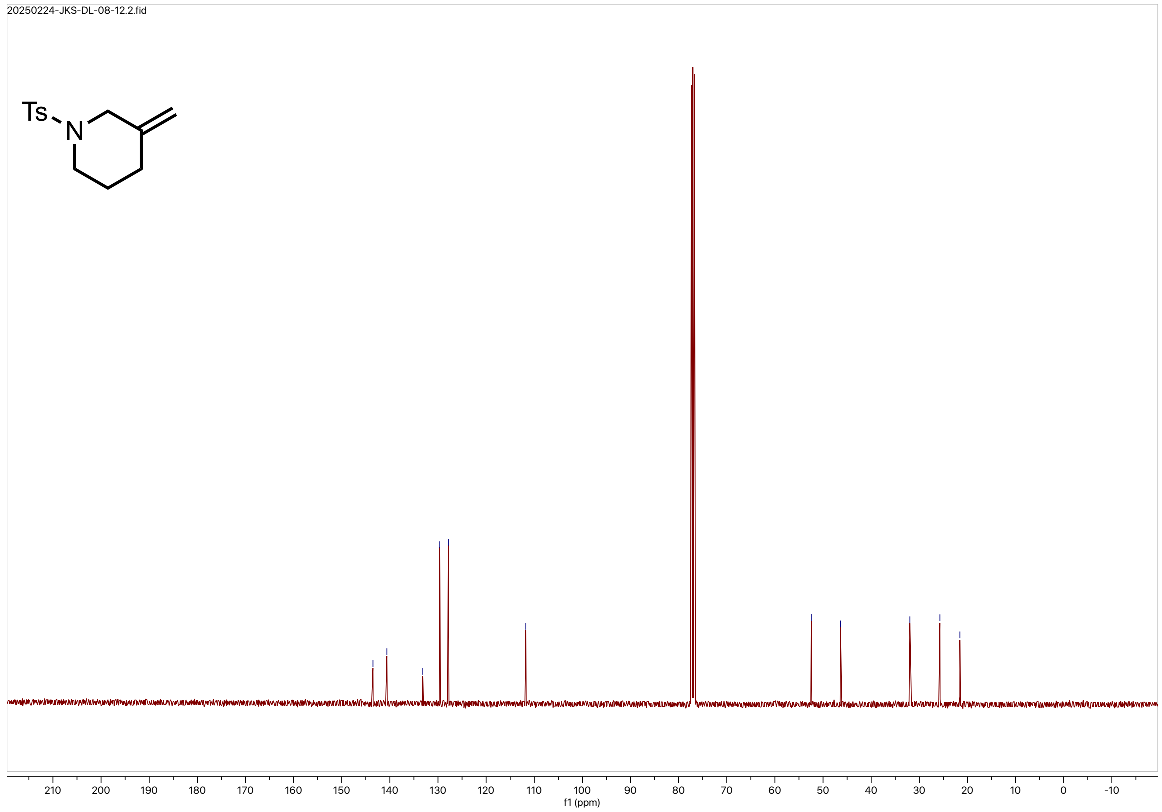

Compound **S1**

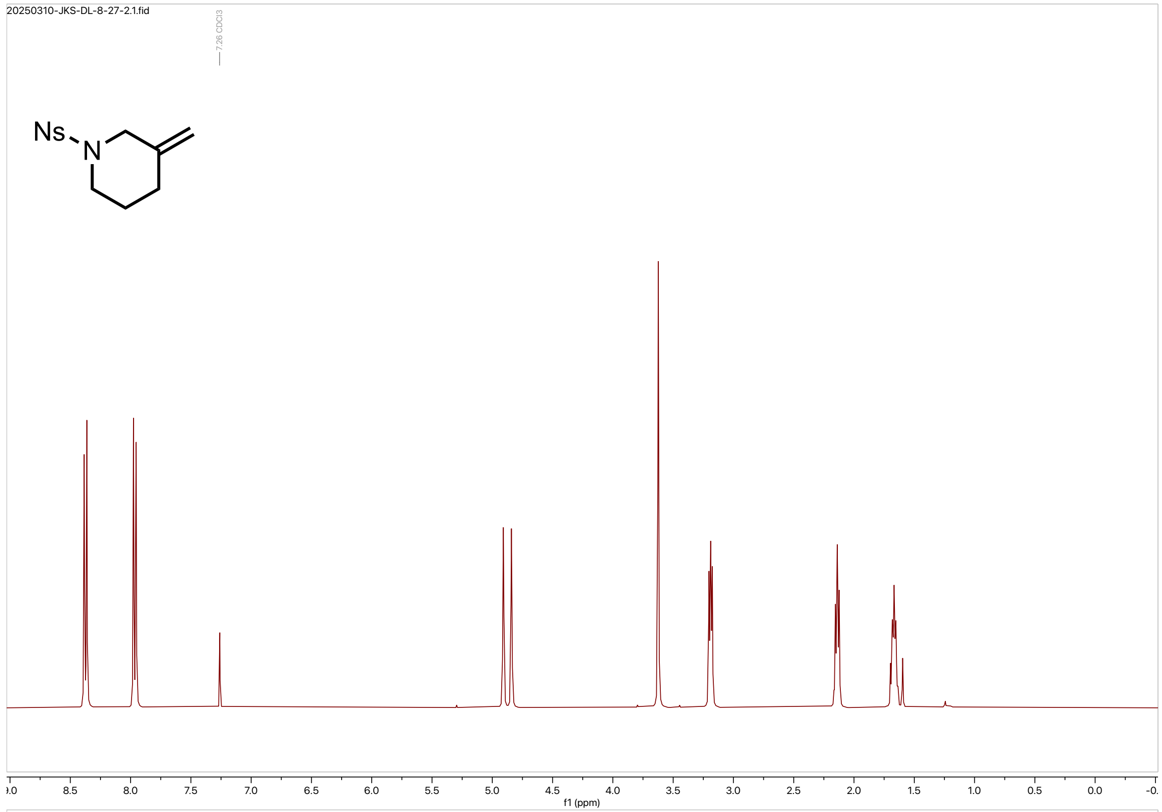
20250310-JKS-DL-8-27-2.1.fid
7.26 CDCl3
Ns
N
f1 (ppm)

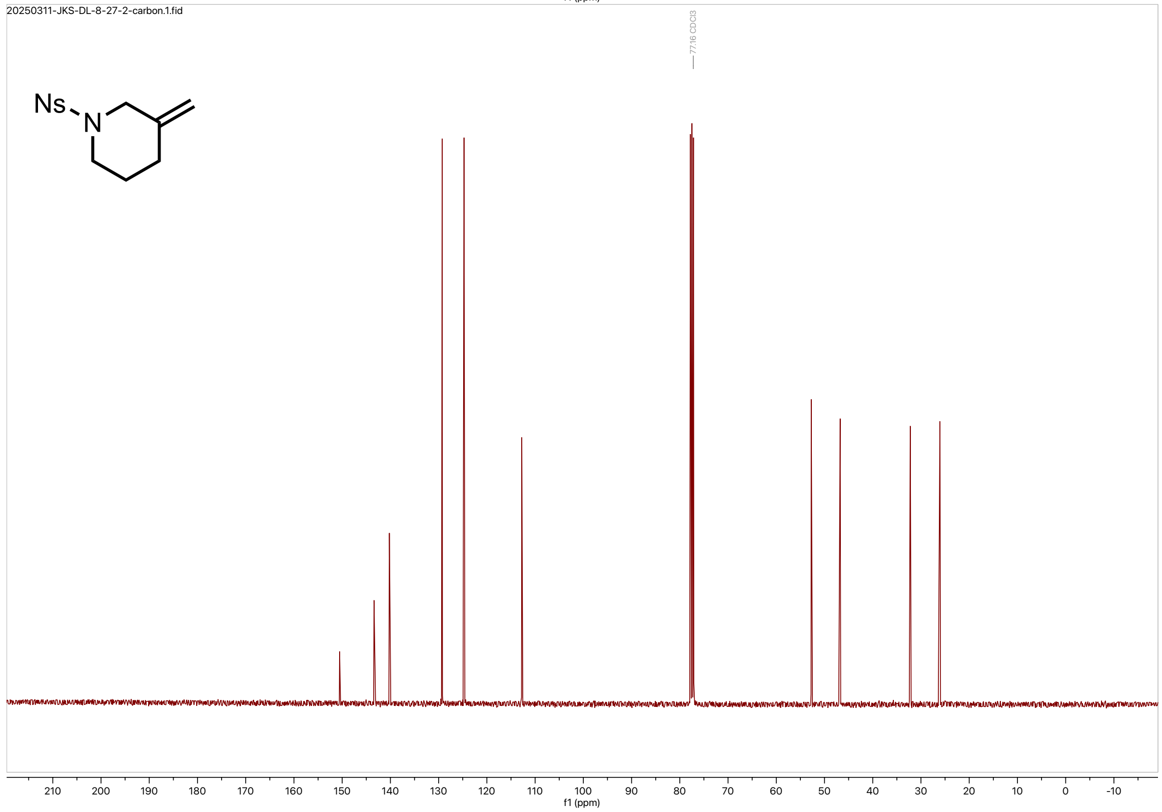
20250311-JKS-DL-8-27-2-carbon.1.fid
77.16 CDCl3
Ns
N
f1 (ppm)

Compound **9b**

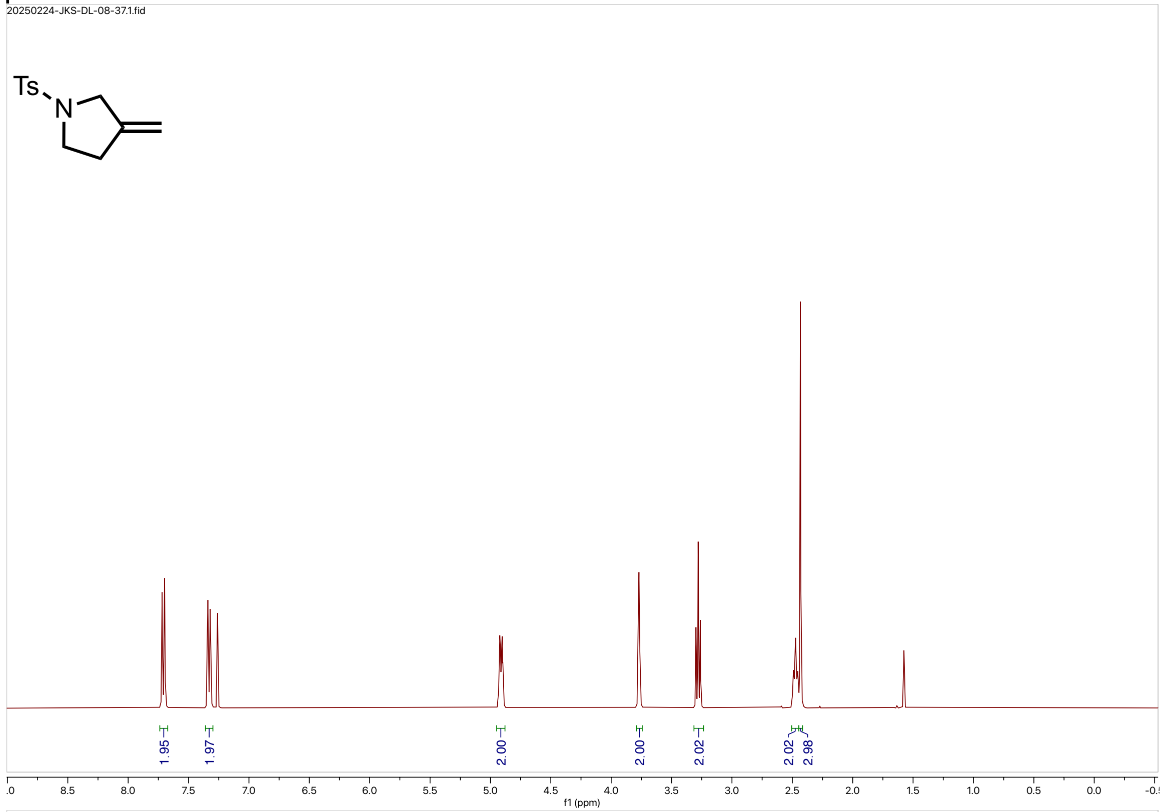
20250224-JKS-DL-08-37.1.fid
Ts
N
1.95
1.97
2.00
2.00
2.02
2.02
2.98
f1 (ppm)

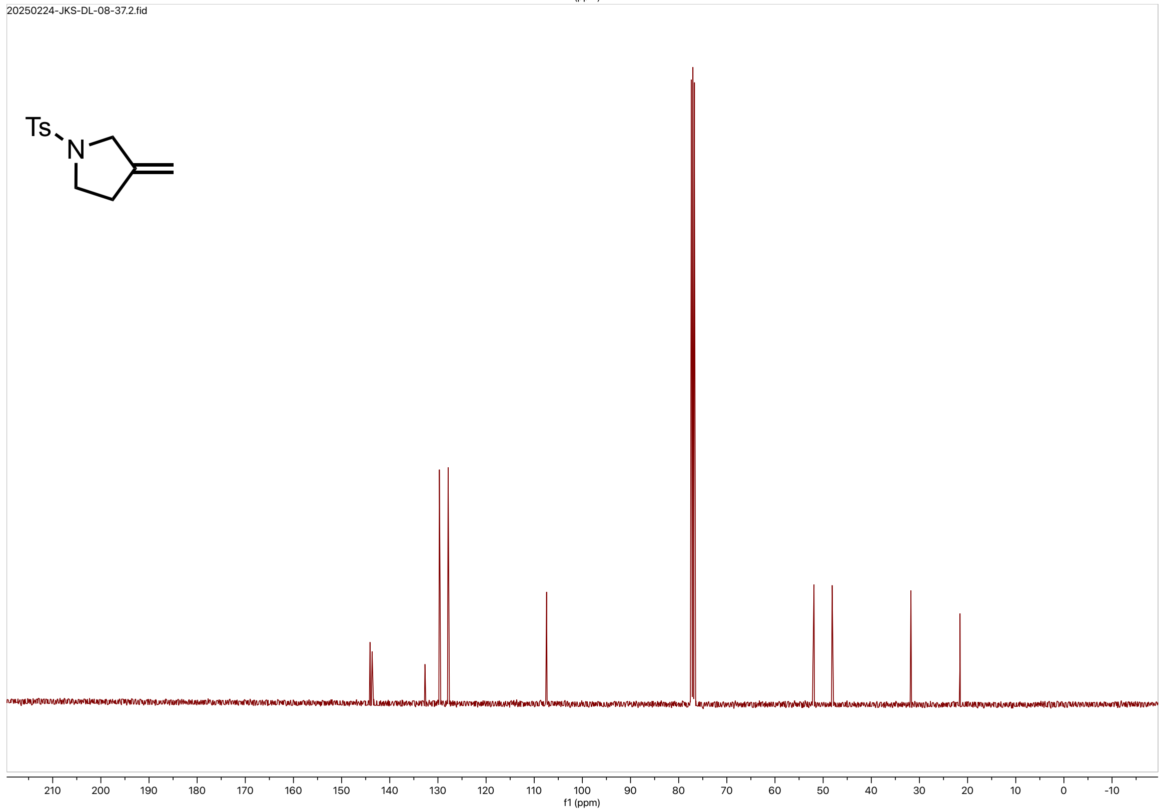
20250224-JKS-DL-08-37.2.fid
Ts
N
f1 (ppm)

Compound **15a**

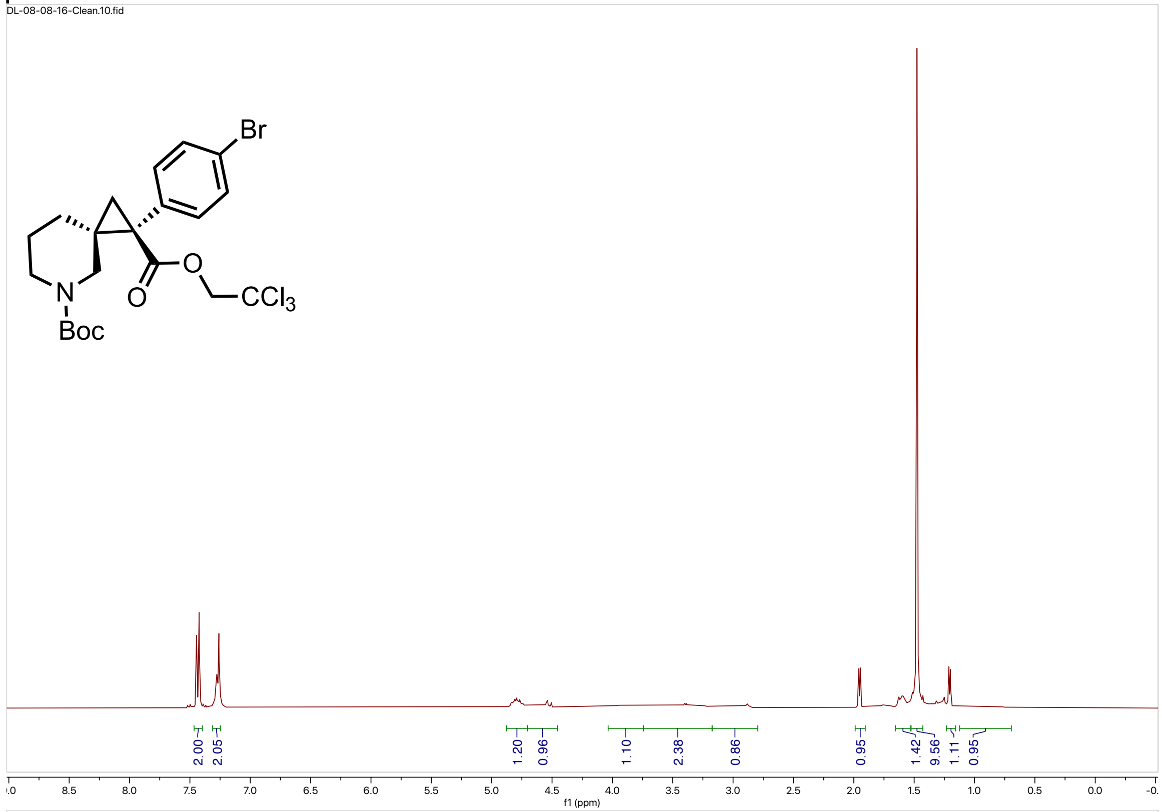

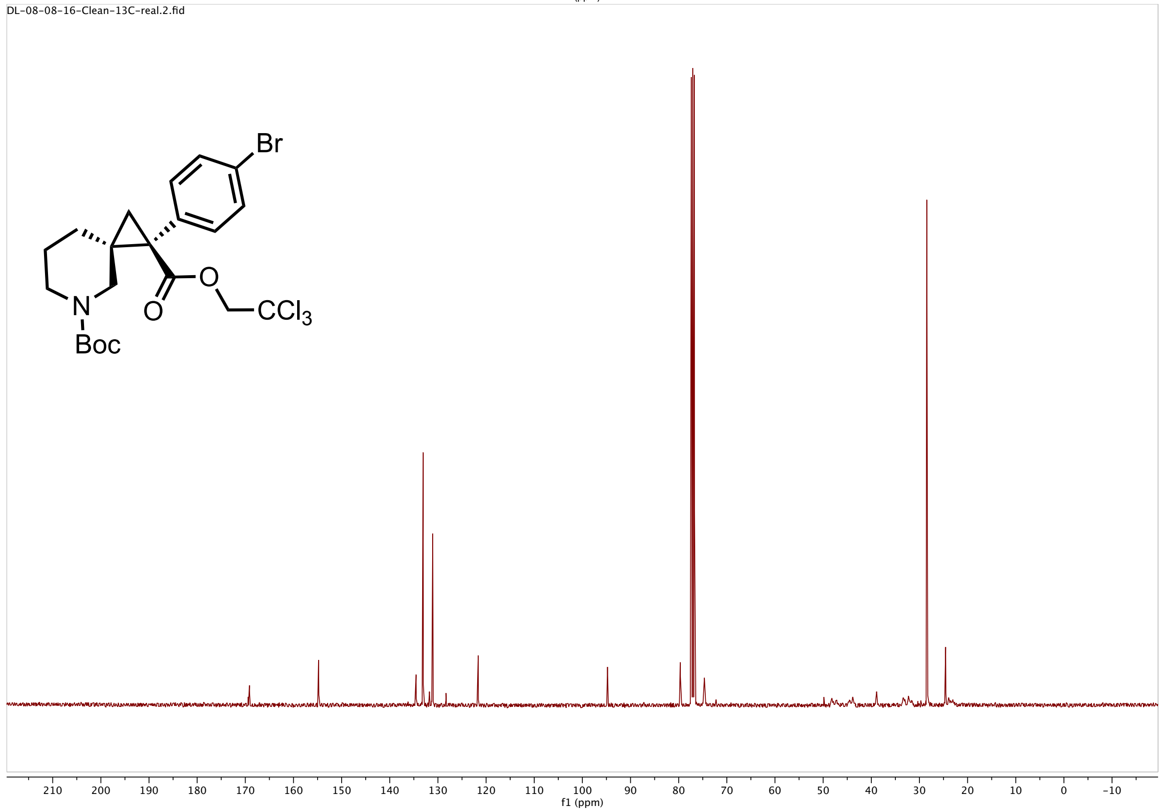

Compound **15b**

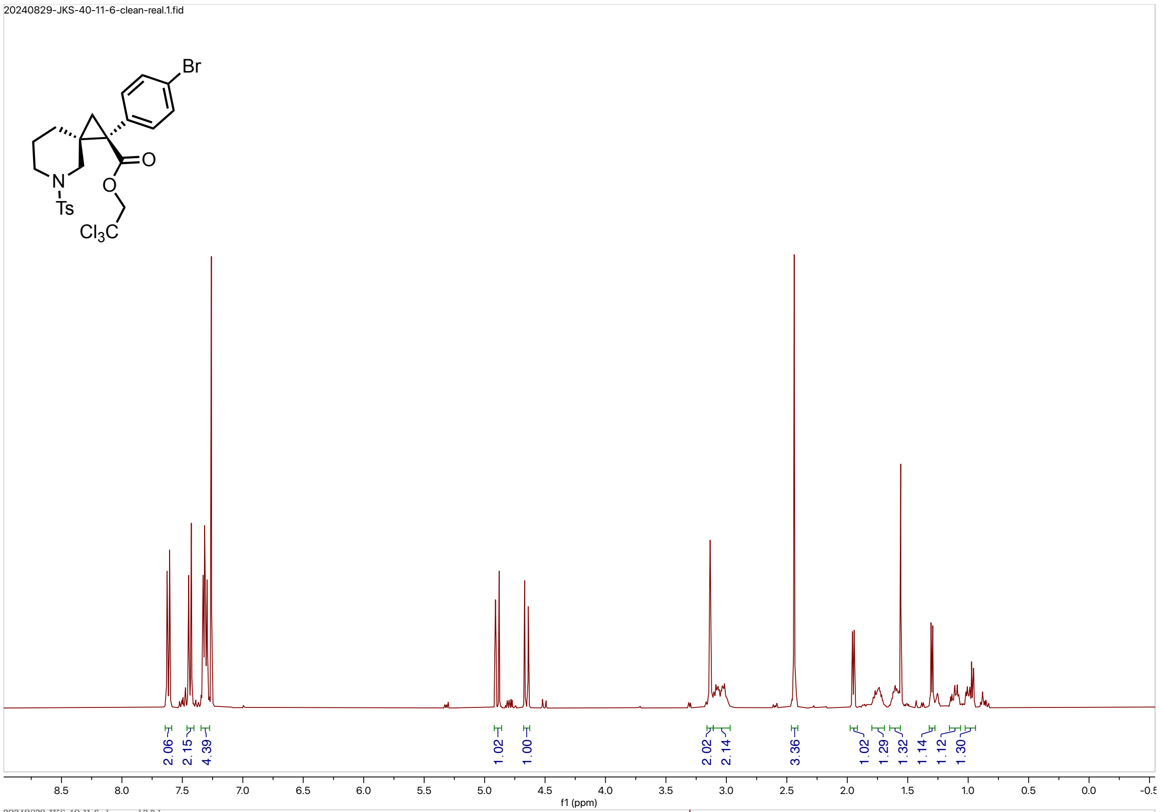

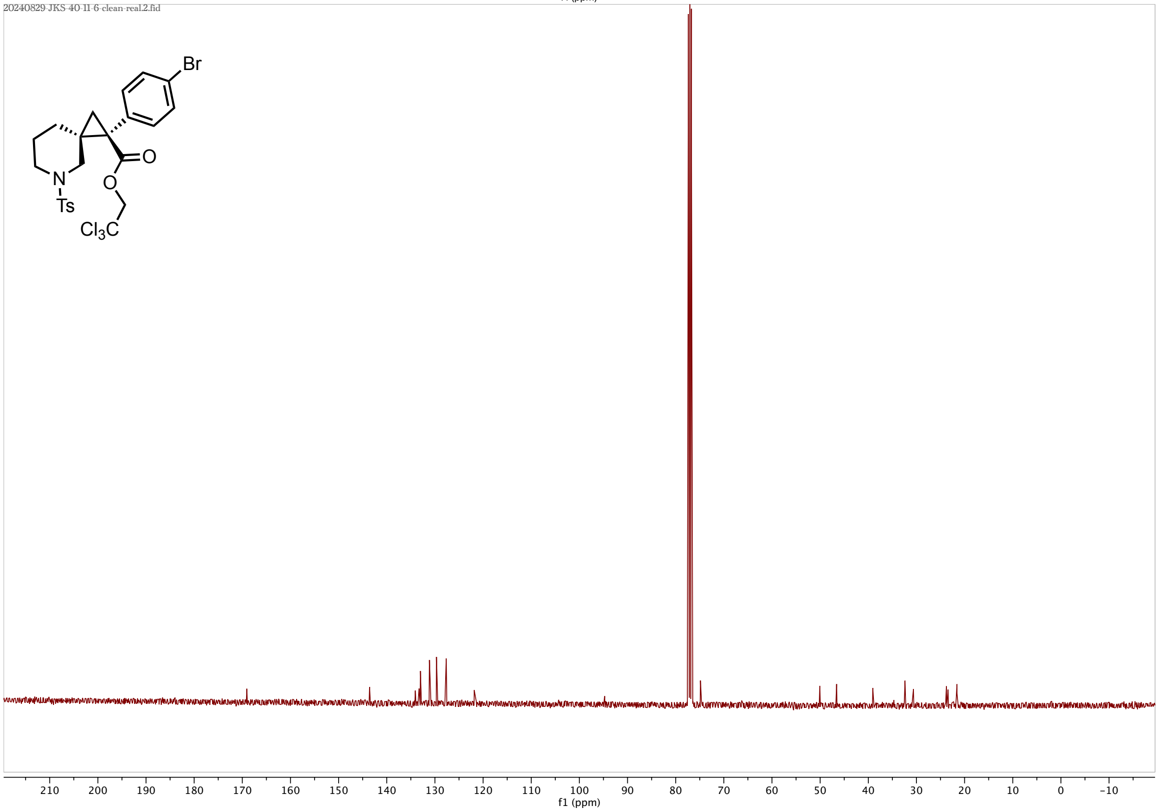

Compound **16**

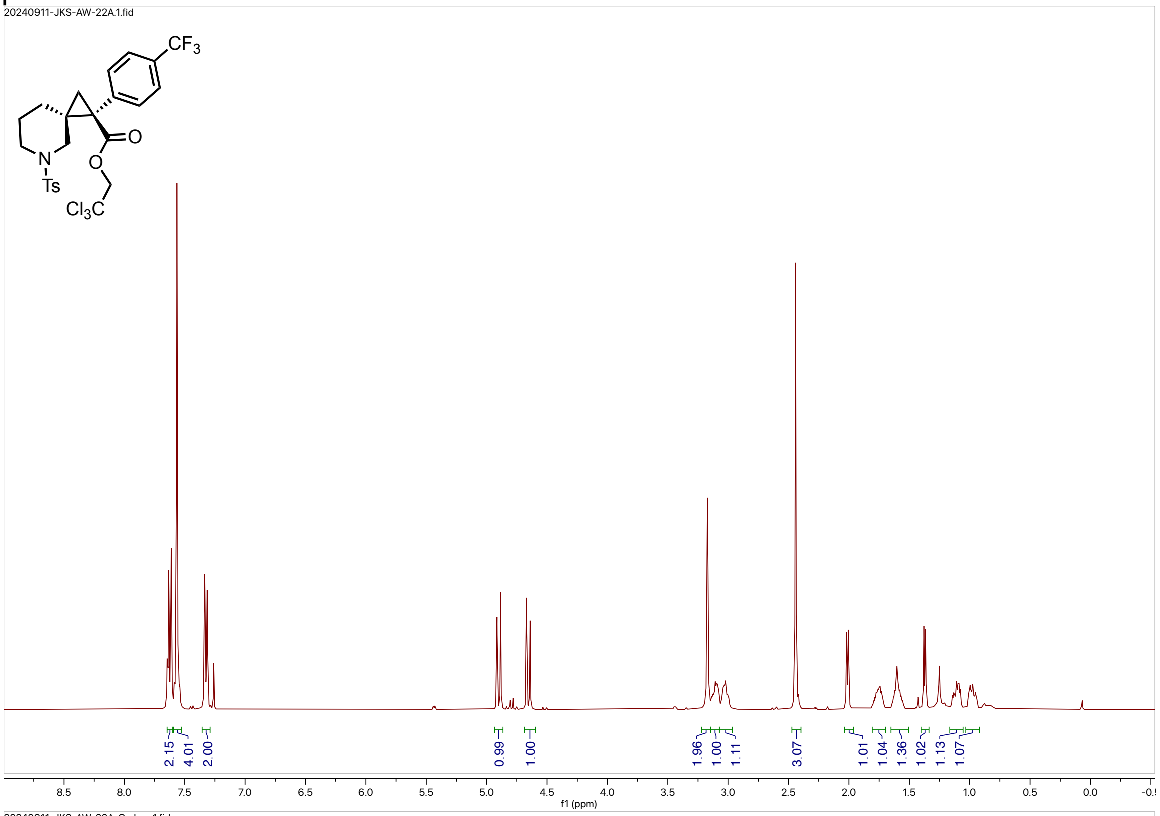
20240911-JKS-AW-22A.1.fid
CF3
N
Ts
O
O
Cl3C
2.15
4.01
2.00
0.99
1.00
1.96
1.00
1.11
3.07
1.01
1.04
1.36
1.02
1.13
1.07
8.5 8.0 7.5 7.0 6.5 6.0 5.5 5.0 4.5 4.0 3.5 3.0 2.5 2.0 1.5 1.0 0.5 0.0
f1 (ppm)

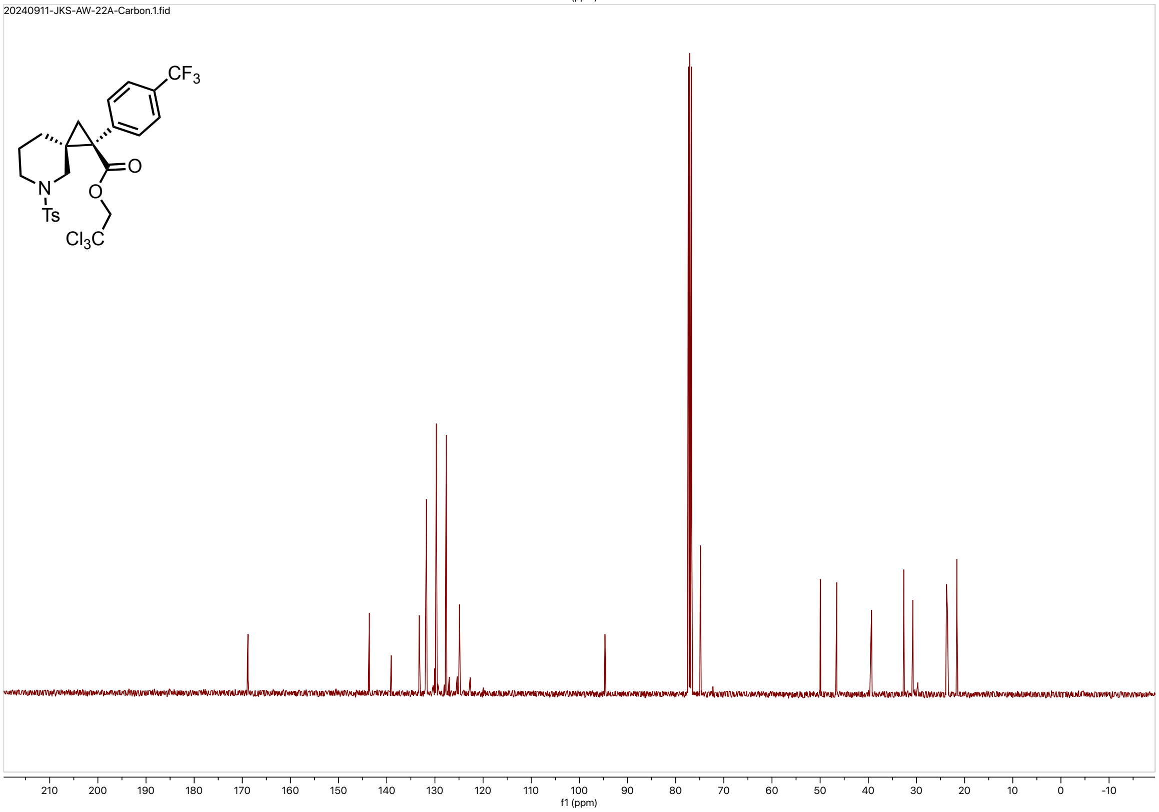
20240911-JKS-AW-22A-Carbon.1.fid
CF3
N
Ts
O
O
Cl3C
210 200 190 180 170 160 150 140 130 120 110 100 90 80 70 60 50 40 30 20 10 0 -10
f1 (ppm)

20240911-JKS-AW-22A.2.fid

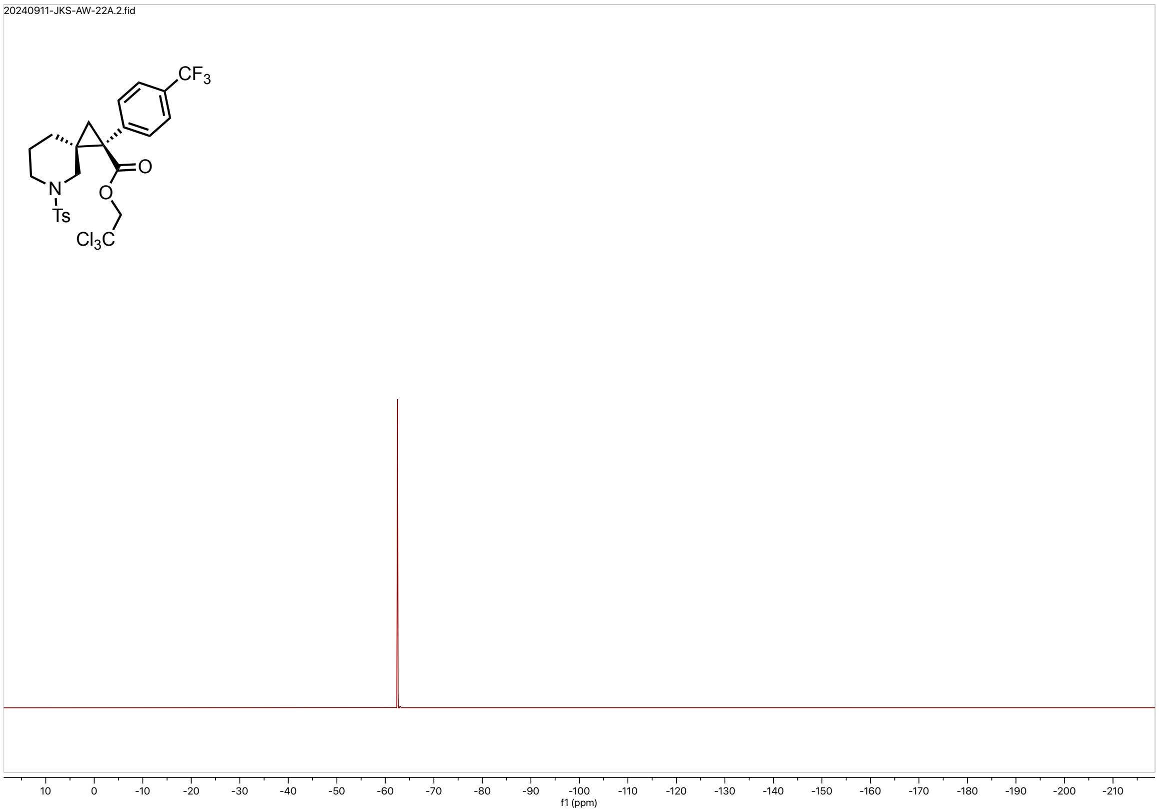

Compound **17**

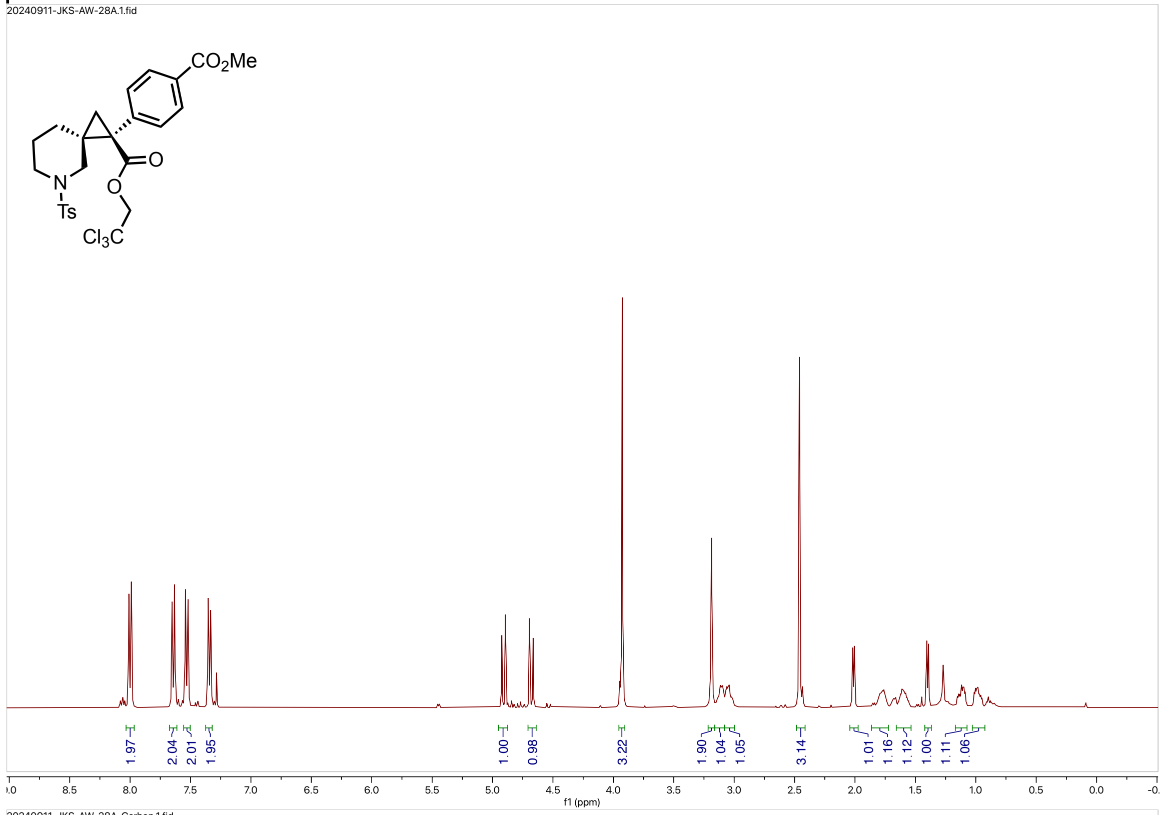
20240911-JKS-AW-28A.1.fid
CO_2Me
N
Ts
O
O
Cl_3C
1.97
2.04
2.01
1.95
1.00
0.98
3.22
1.90
1.04
1.05
3.14
1.01
1.16
1.12
1.00
1.11
1.06
f1 (ppm)

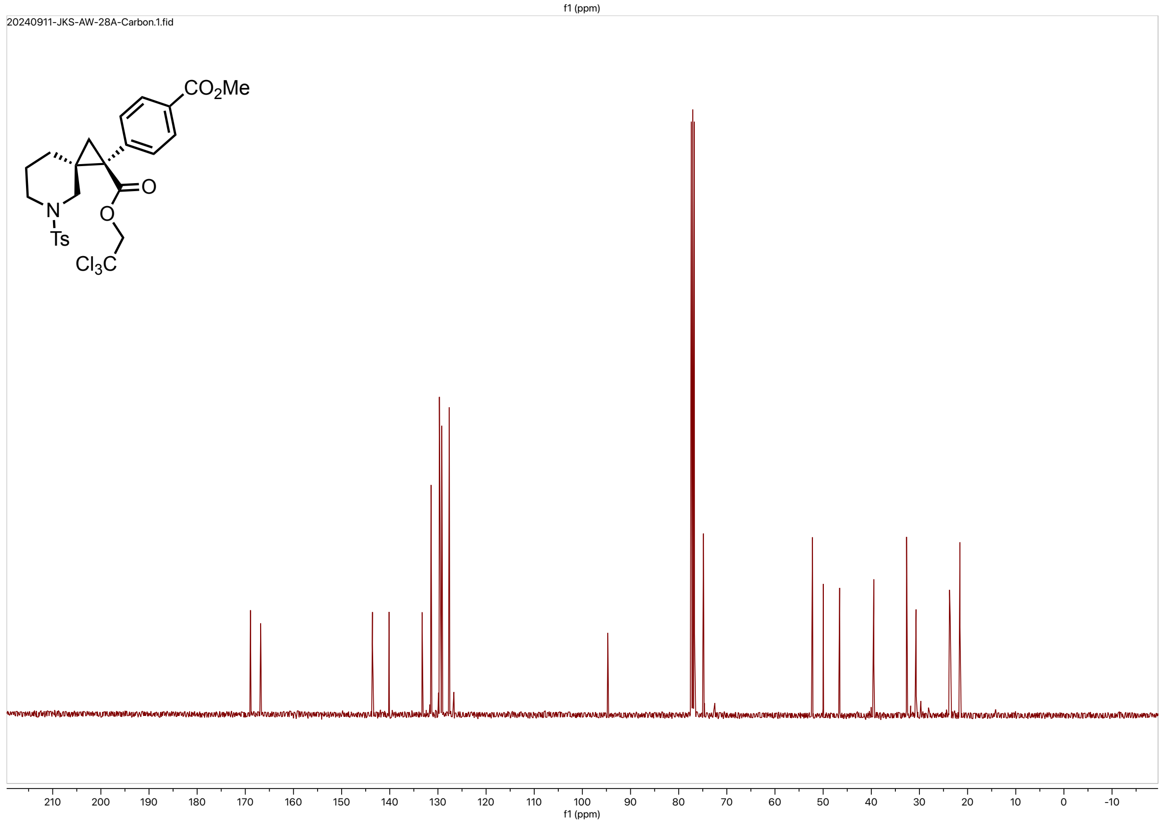
20240911-JKS-AW-28A-Carbon.1.fid
CO_2Me
N
Ts
O
O
Cl_3C
f1 (ppm)

Compound **18**

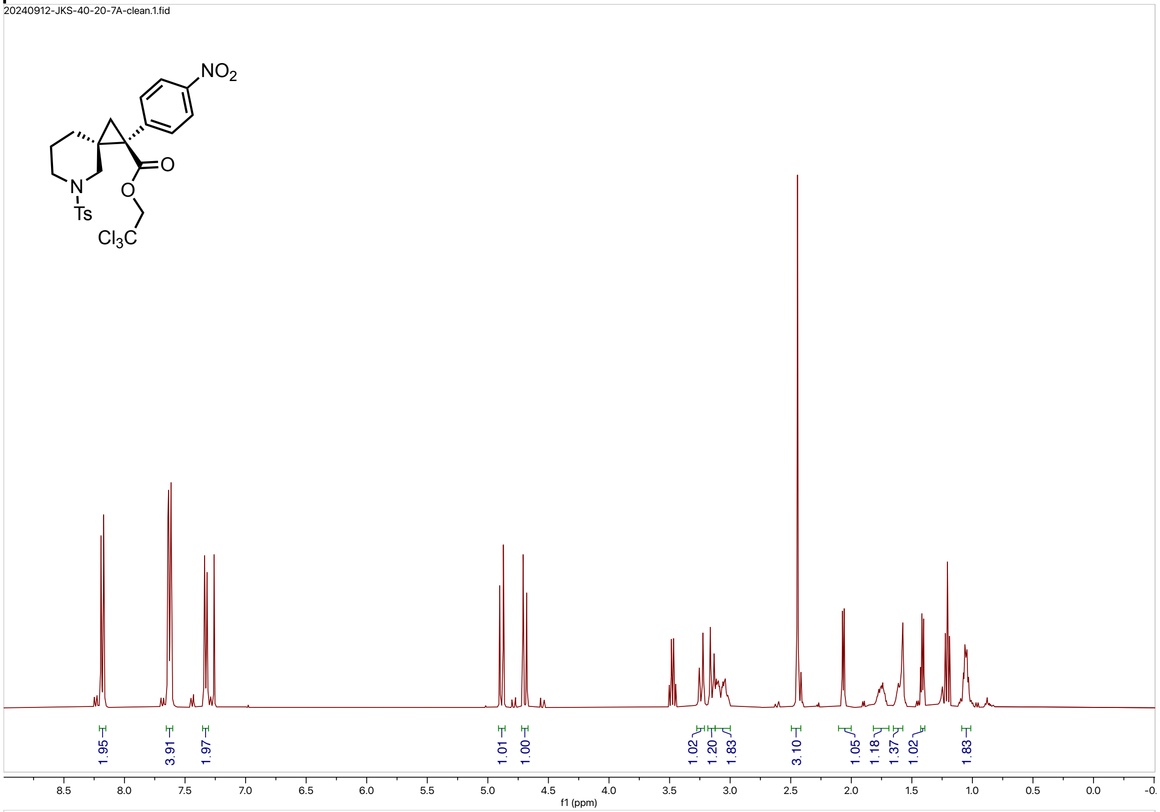
20240912-JKS-40-20-7A-clean.1.fid
NO2
N
Ts
O
O
Cl3C
1.95
3.91
1.97
1.01
1.00
1.02
1.20
1.83
3.10
1.05
1.18
1.37
1.02
1.83
8.5
8.0
7.5
7.0
6.5
6.0
5.5
5.0
4.5
4.0
3.5
3.0
2.5
2.0
1.5
1.0
0.5
0.0
f1 (ppm)

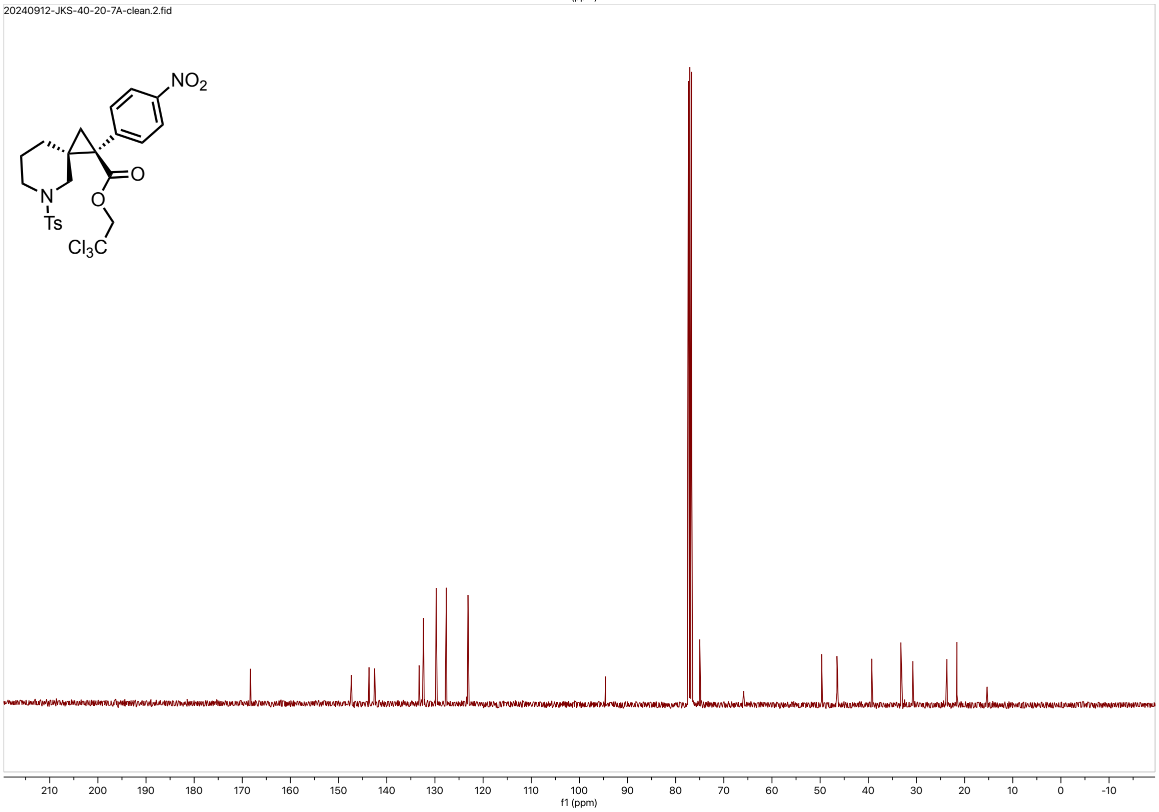
20240912-JKS-40-20-7A-clean.2.fid
NO2
N
Ts
O
O
Cl3C
210
200
190
180
170
160
150
140
130
120
110
100
90
80
70
60
50
40
30
20
10
0
-10
f1 (ppm)

Compound **19**

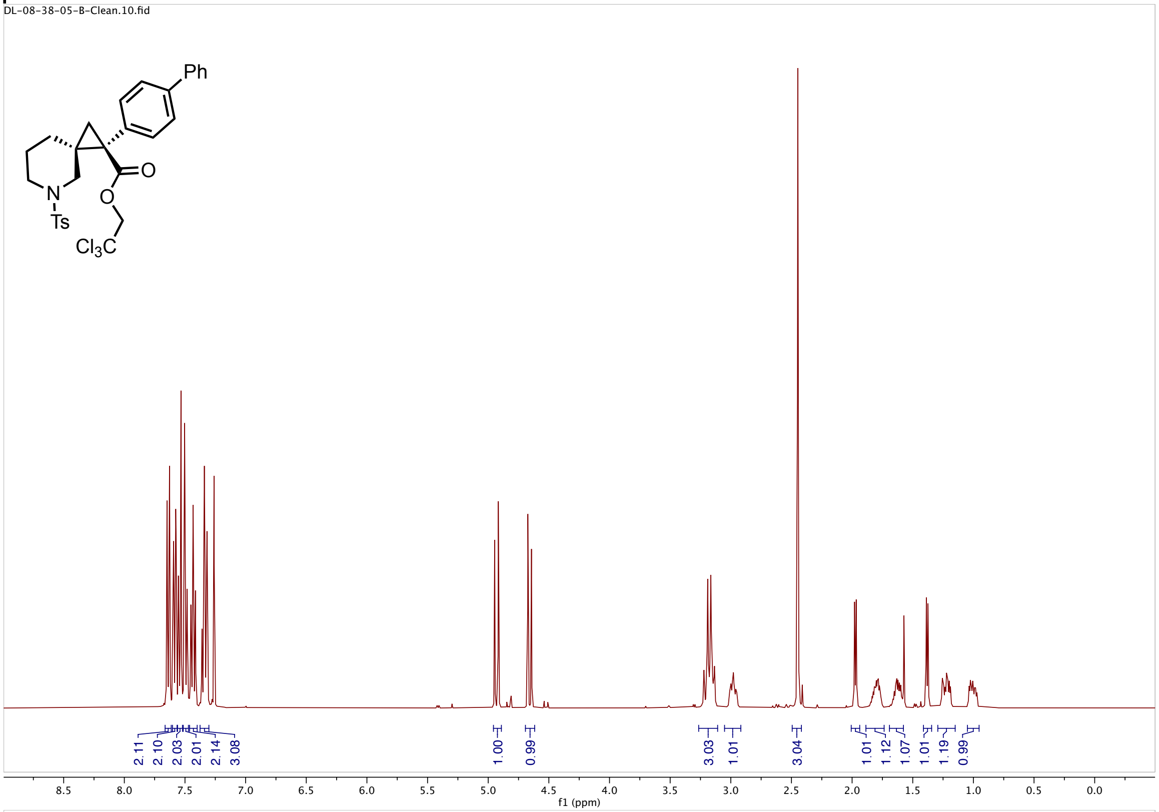
DL-08-38-05-B-Clean.10.fid
Ph
N
Ts
O
O
Cl3C
2.11
2.10
2.03
2.01
2.14
3.08
1.00
0.99
3.03
1.01
3.04
1.01
1.12
1.07
1.01
1.19
0.99
8.5
8.0
7.5
7.0
6.5
6.0
5.5
5.0
4.5
4.0
3.5
3.0
2.5
2.0
1.5
1.0
0.5
0.0
f1 (ppm)

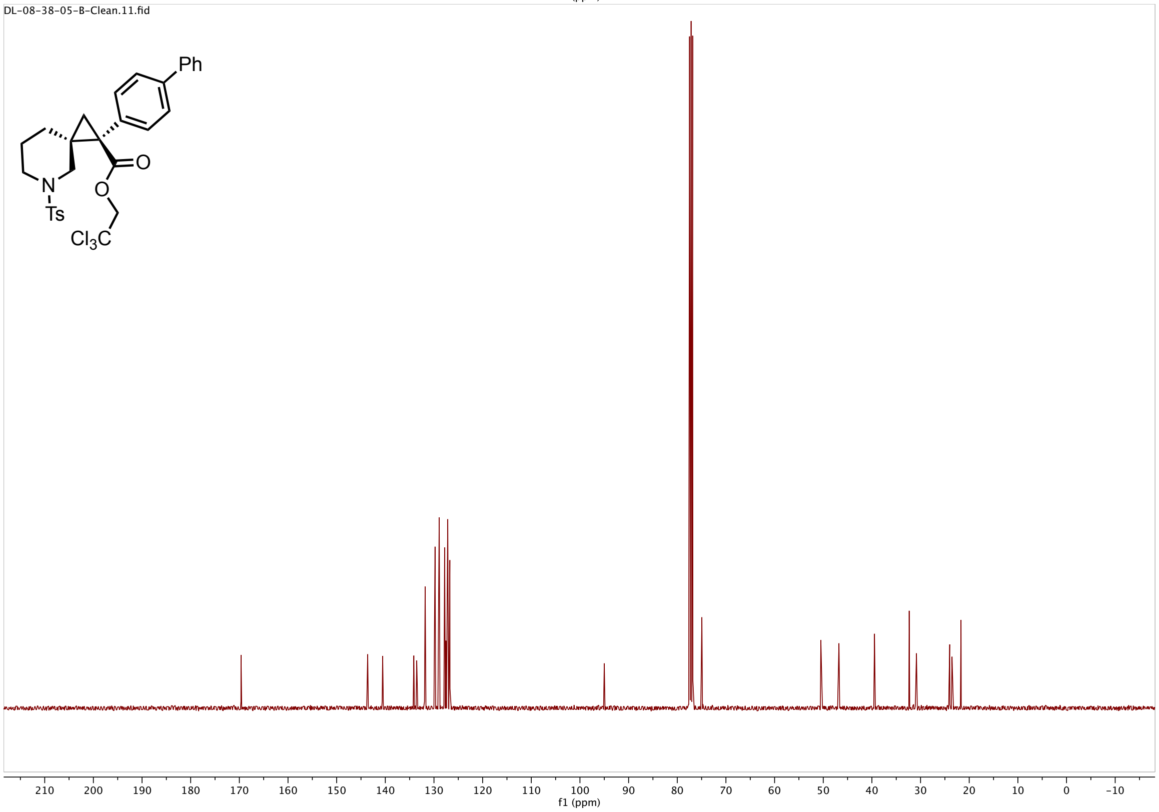
DL-08-38-05-B-Clean.11.fid
Ph
N
Ts
O
O
Cl3C
210
200
190
180
170
160
150
140
130
120
110
100
90
80
70
60
50
40
30
20
10
0
-10
f1 (ppm)

Compound **20**

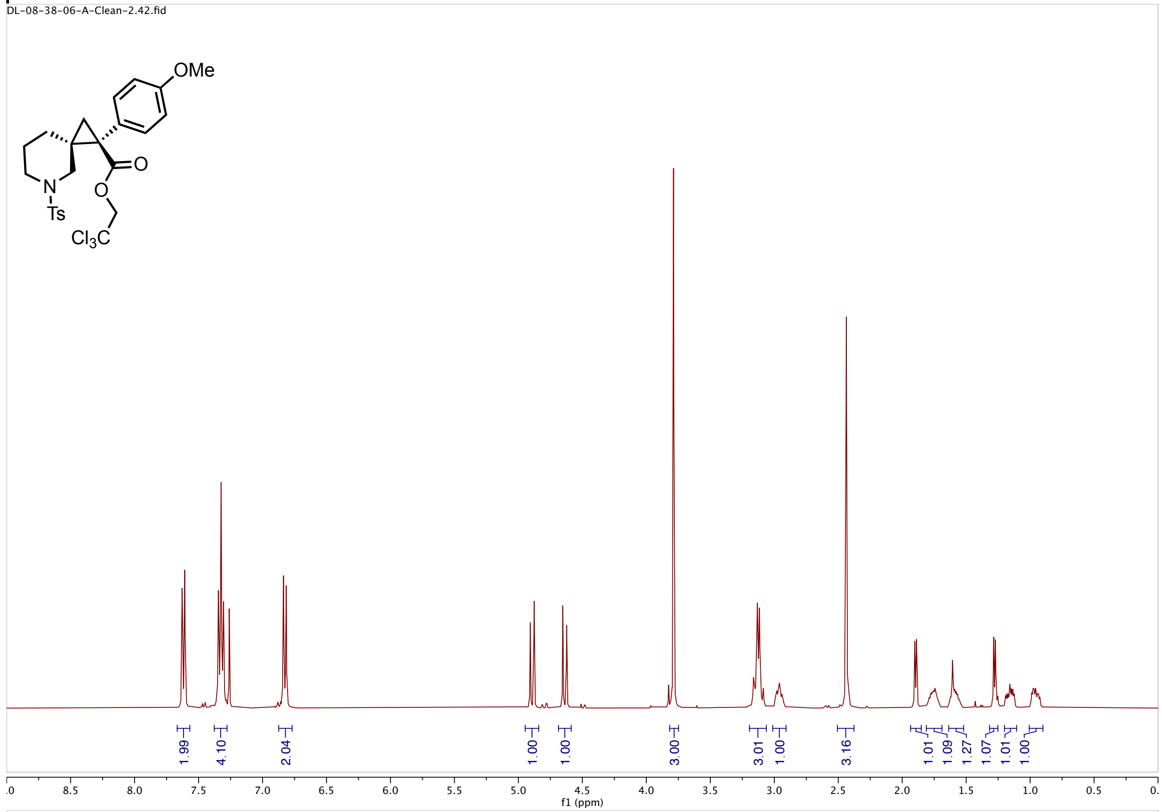
DL-08-38-06-A-Clean-2.42.fid
OMe
N
Ts
O
O
Cl3C
1.99
4.10
2.04
1.00
1.00
3.00
3.01
1.00
3.16
1.01
1.09
1.27
1.07
1.01
1.00
f1 (ppm)

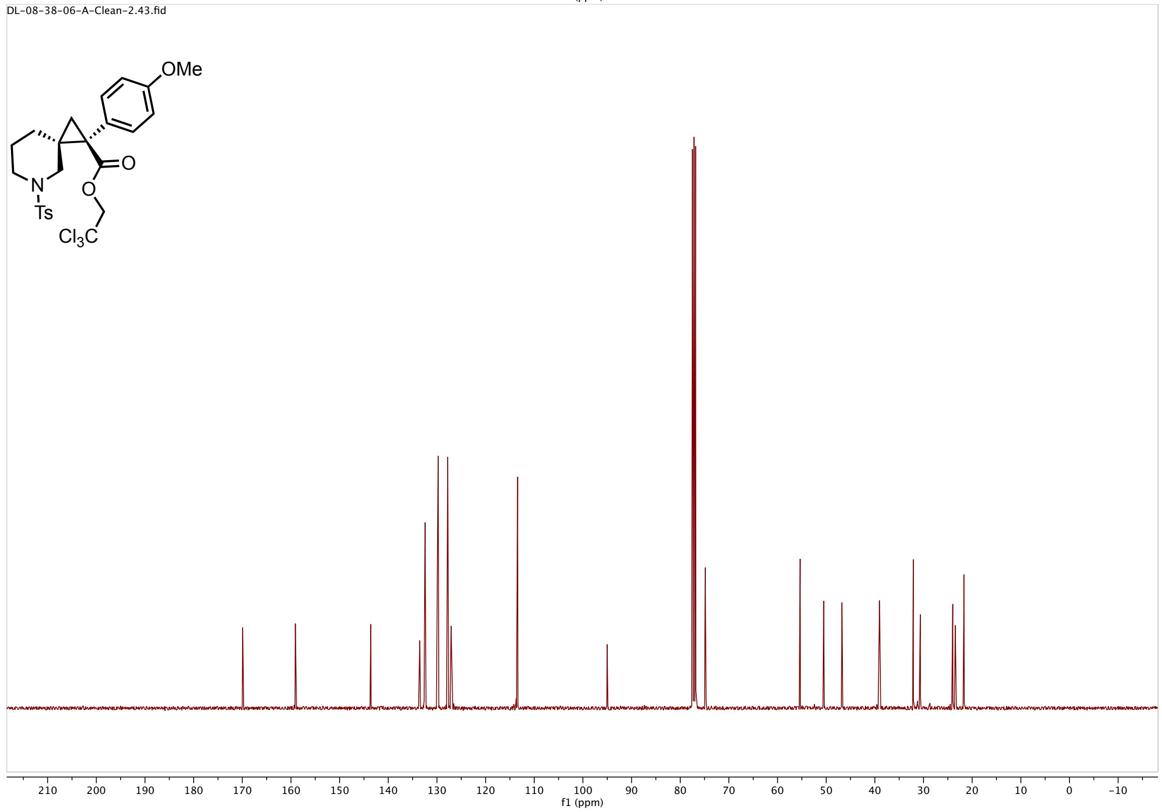
DL-08-38-06-A-Clean-2.43.fid
OMe
N
Ts
O
O
Cl3C
f1 (ppm)

Compound **21**

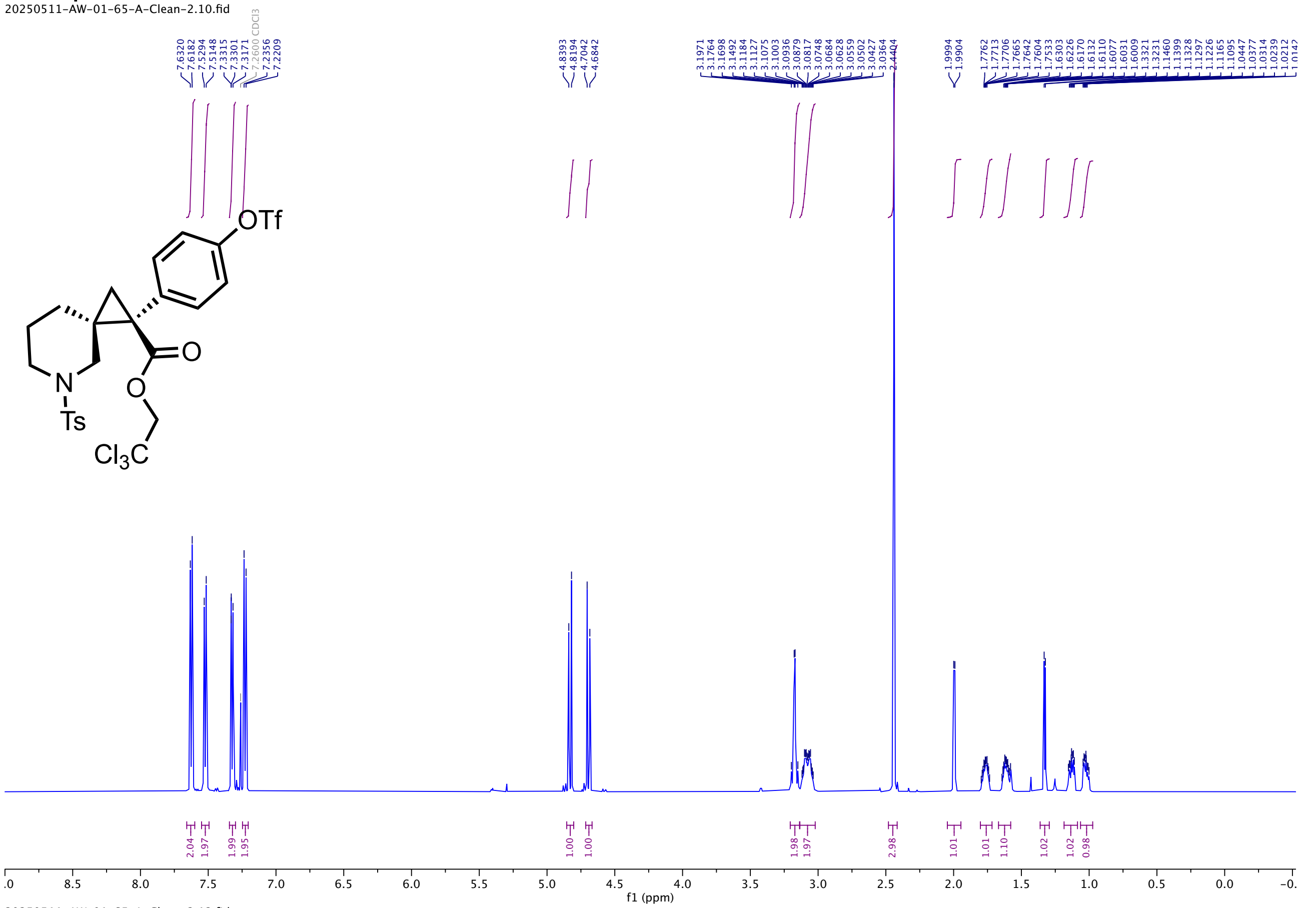
20250511-AW-01-65-A-Clean-2.10.fid
OTf
N
Ts
O
O
Cl3C
f1 (ppm)

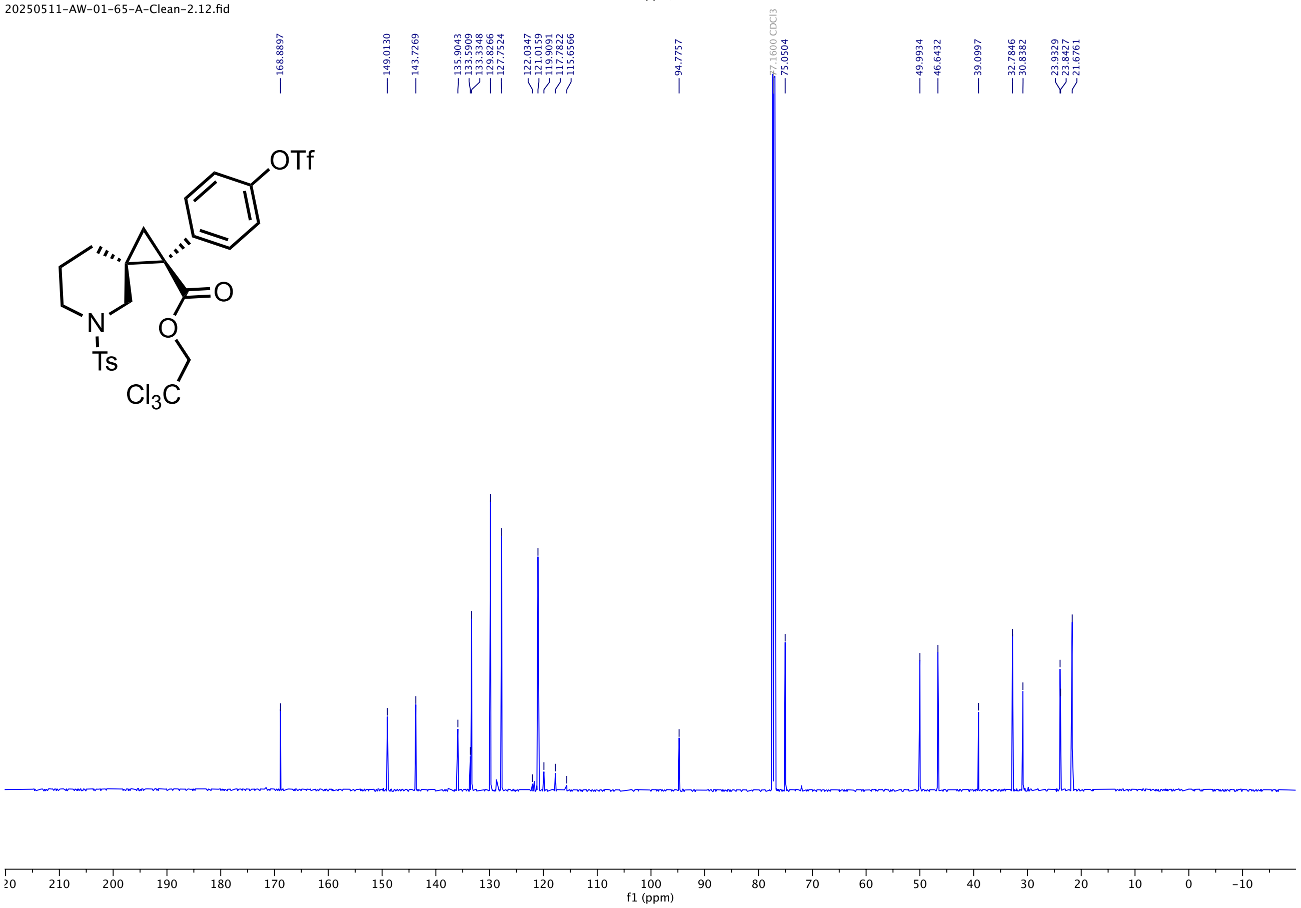
20250511-AW-01-65-A-Clean-2.12.fid
OTf
N
Ts
O
O
Cl3C
f1 (ppm)

20250511-AW-01-65-A-Clean-2.11.fid

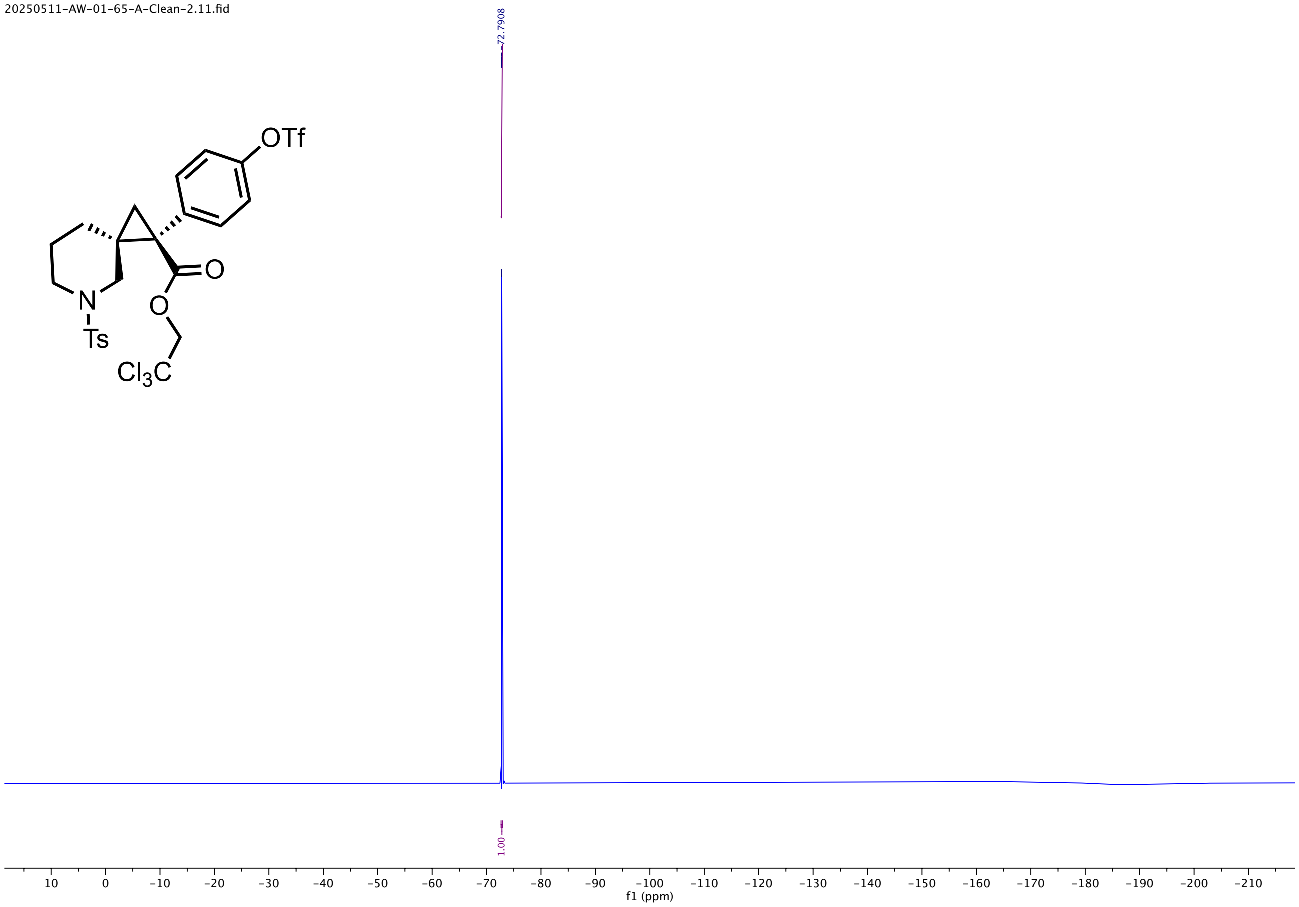

Compound **22**

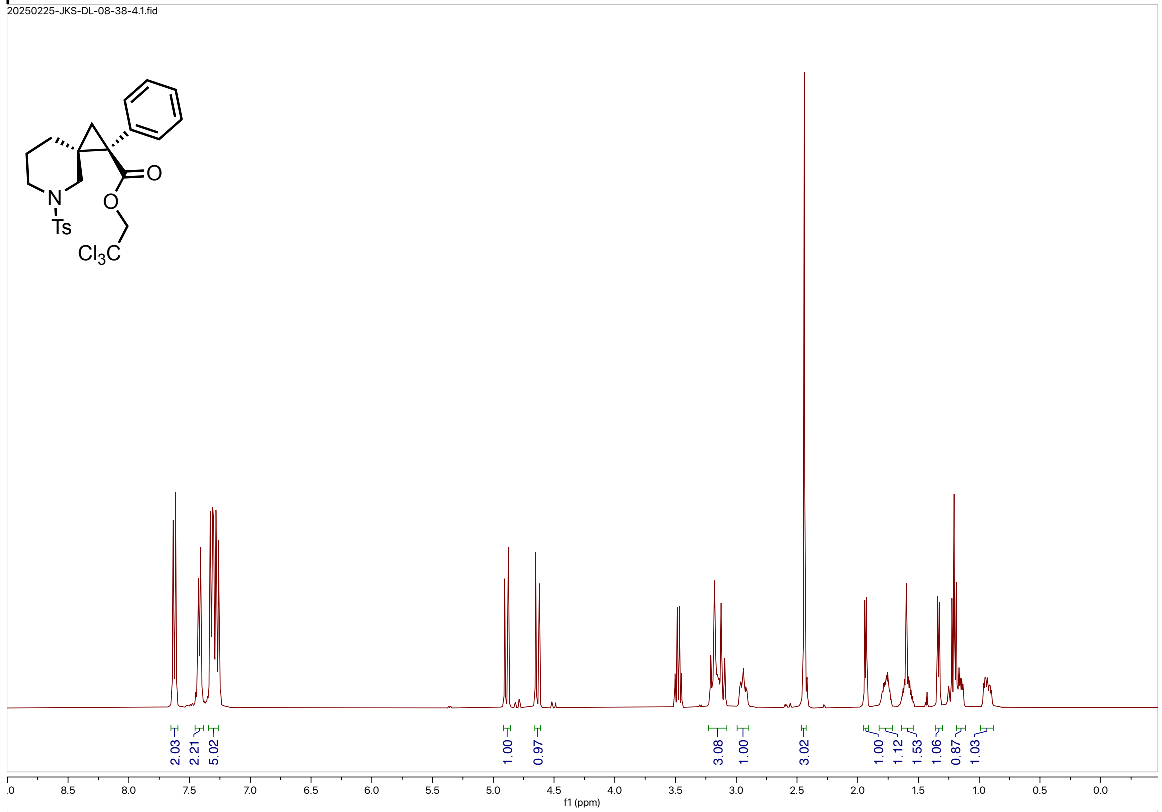
20250225-JKS-DL-08-38-4.1.fid
N
Ts
O
O
Cl3C
2.03
2.21
5.02
1.00
0.97
3.08
1.00
3.02
1.00
1.12
1.53
1.06
0.87
1.03
f1 (ppm)

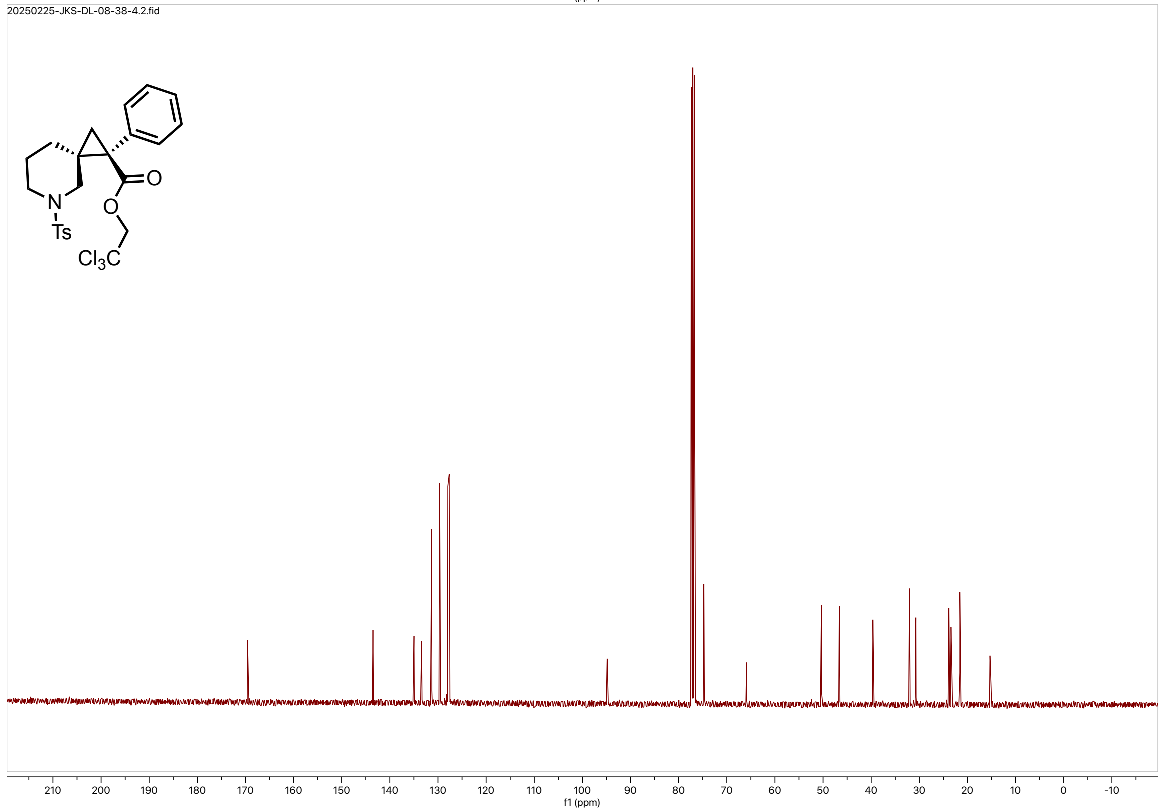
20250225-JKS-DL-08-38-4.2.fid
N
Ts
O
O
Cl3C
f1 (ppm)

Compound **23**

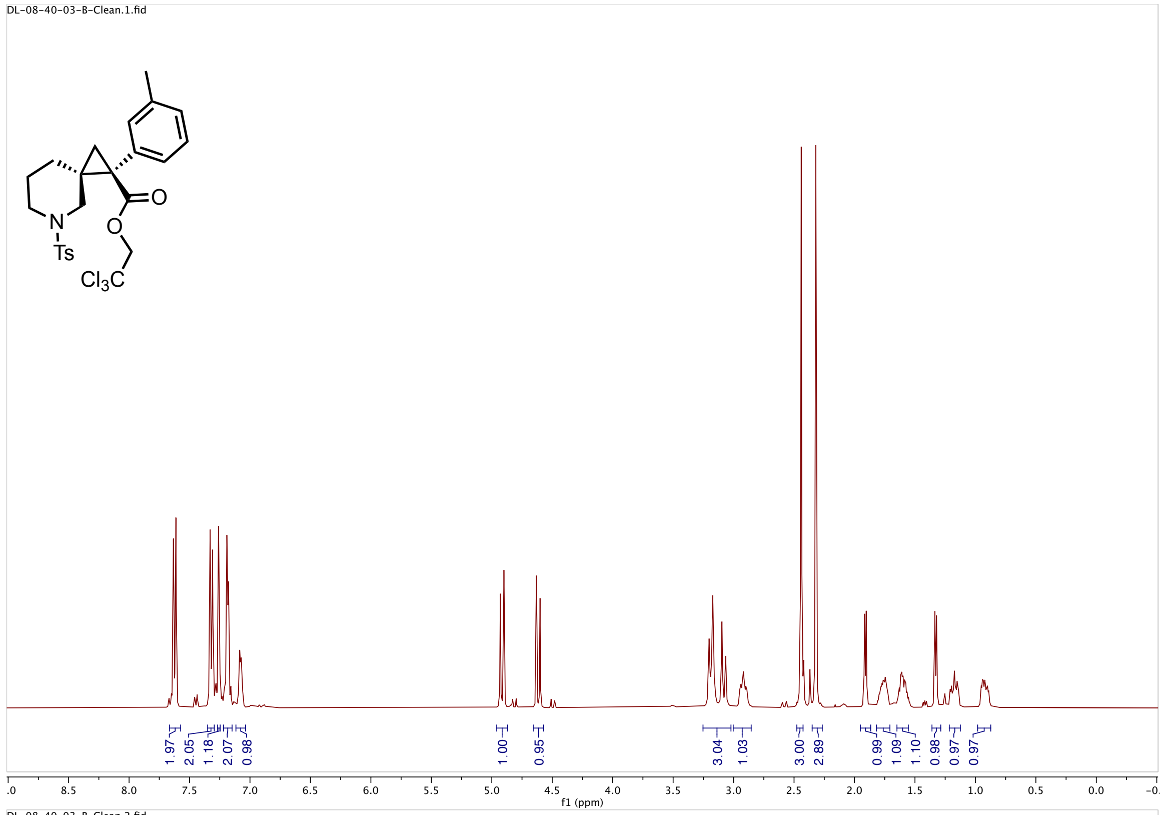
DL-08-40-03-B-Clean.1.fid
N
Ts
O
O
Cl3C
1.97
2.05
1.18
2.07
0.98
1.00
0.95
3.04
1.03
3.00
2.89
0.99
1.09
1.10
0.98
0.97
0.97
f1 (ppm)

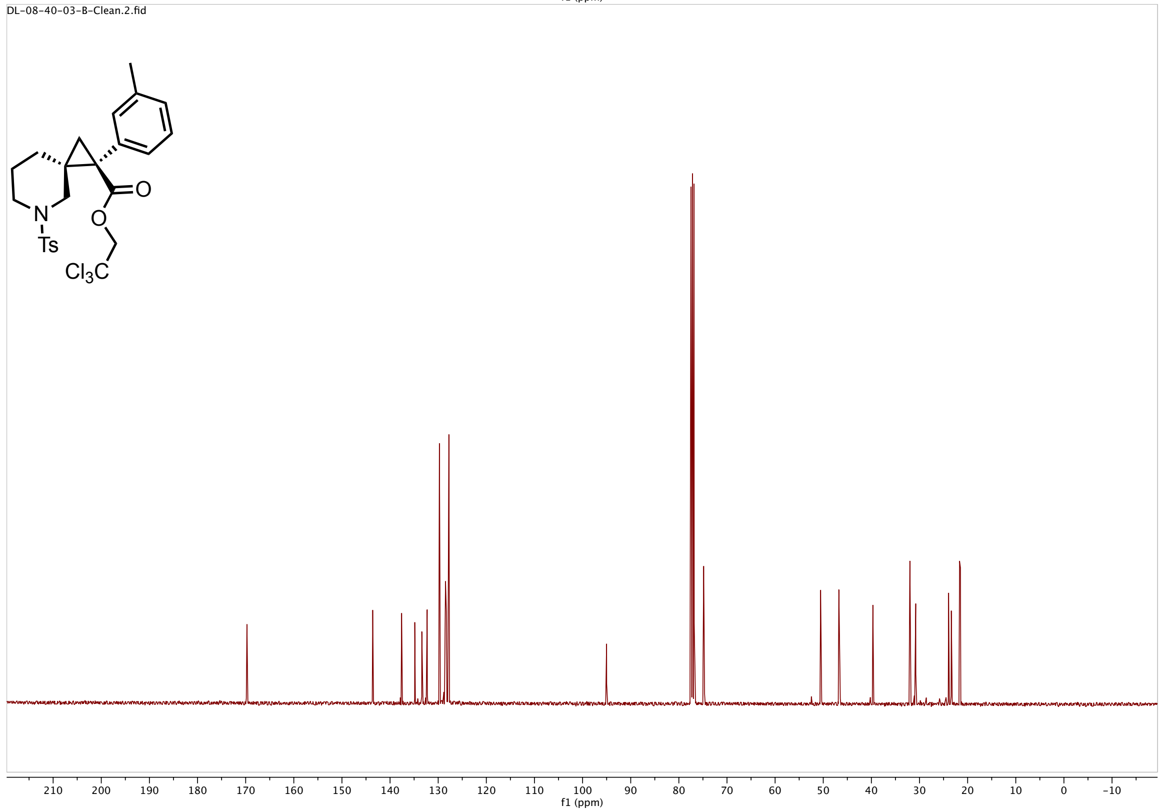
DL-08-40-03-B-Clean.2.fid
N
Ts
O
O
Cl3C
f1 (ppm)

Compound **24**

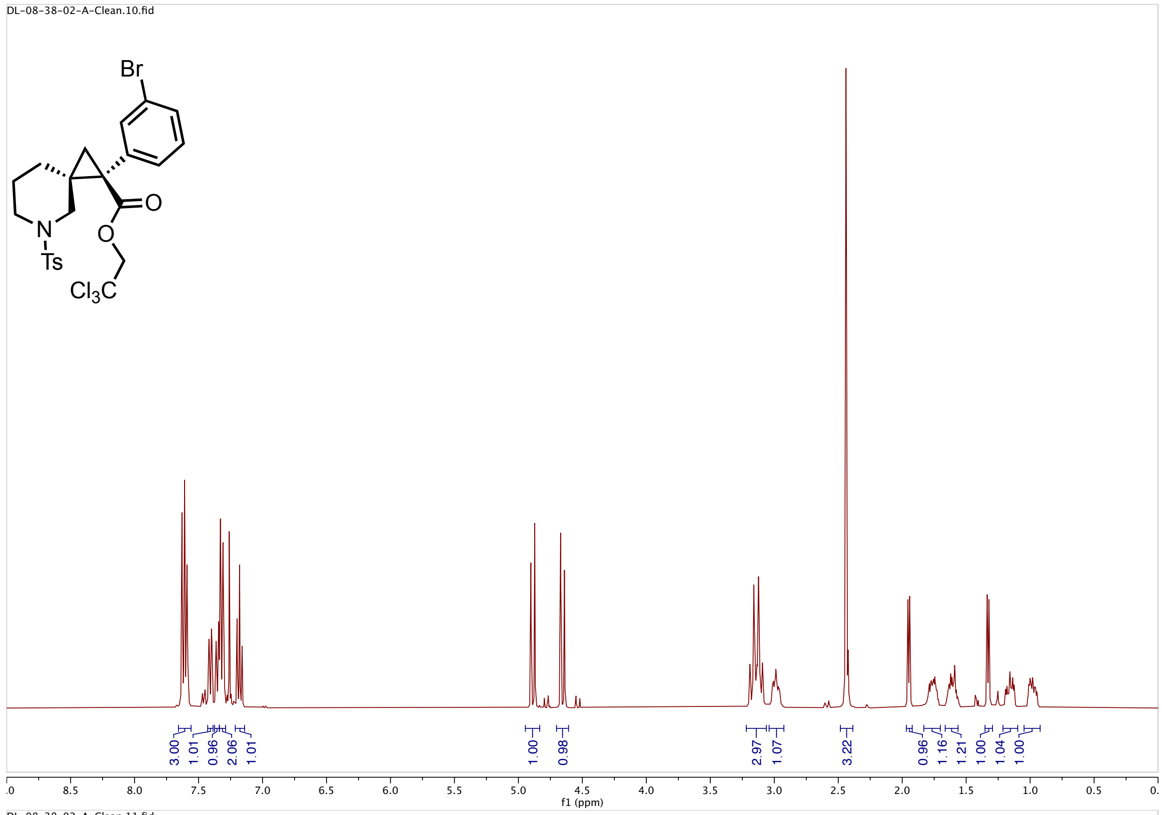
DL-08-38-02-A-Clean.10.fid
Br
N
Ts
O
O
Cl3C
3.00
1.01
0.96
2.06
1.01
1.00
0.98
2.97
1.07
3.22
0.96
1.16
1.21
1.00
1.04
1.00
f1 (ppm)

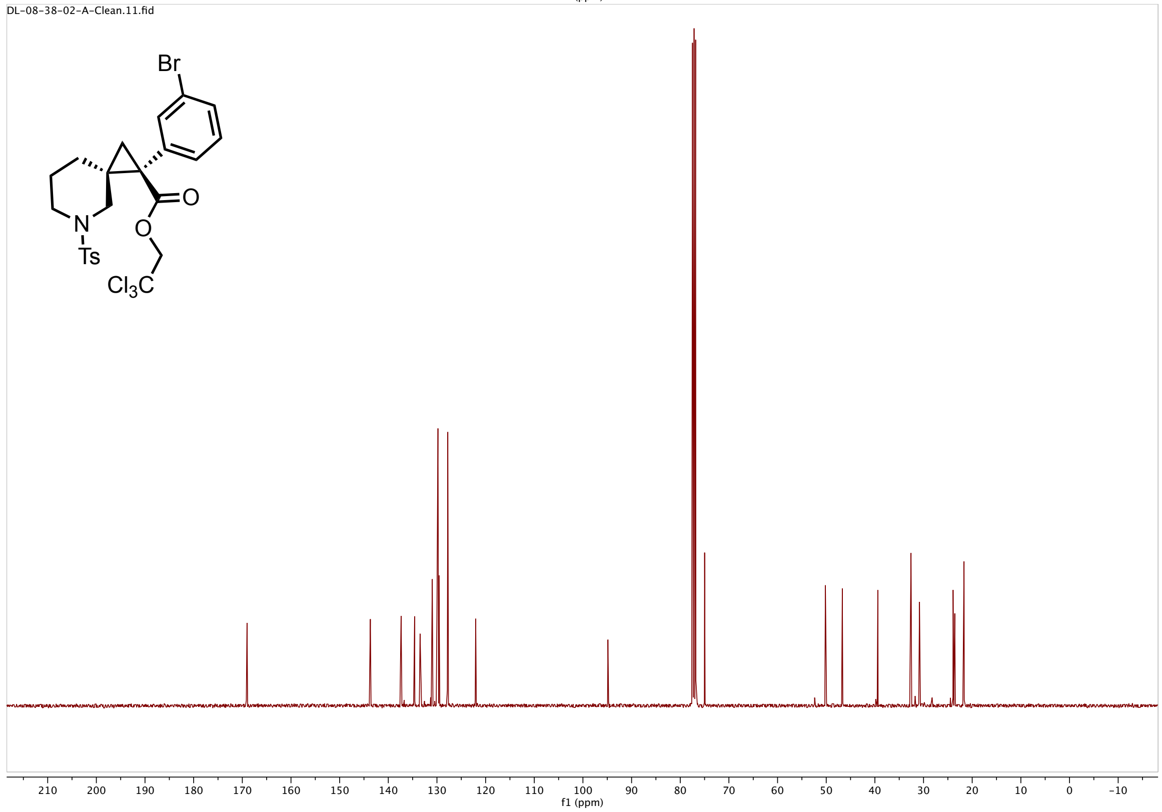
DL-08-38-02-A-Clean.11.fid
Br
N
Ts
O
O
Cl3C
f1 (ppm)

Compound **25**

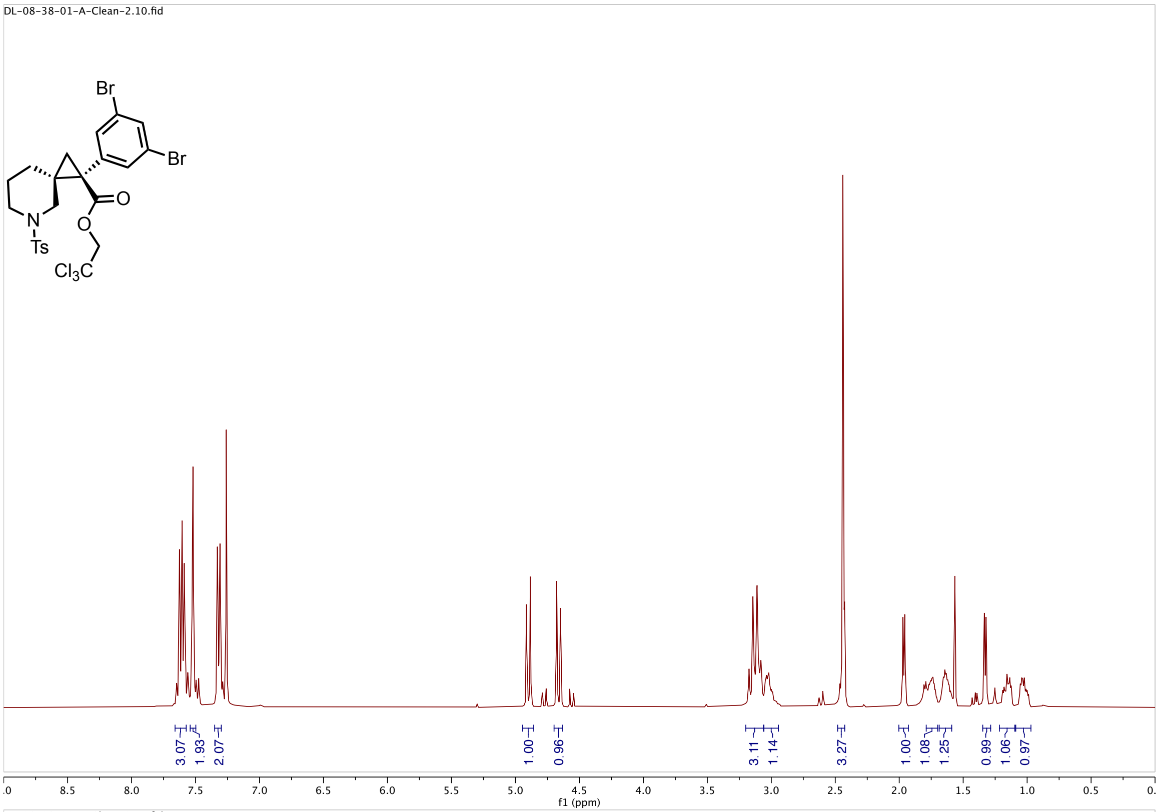
DL-08-38-01-A-Clean-2.10.fid
Br
Br
N
Ts
O
O
Cl_3C
3.07
1.93
2.07
1.00
0.96
3.11
1.14
3.27
1.00
1.08
1.25
0.99
1.06
0.97
f1 (ppm)

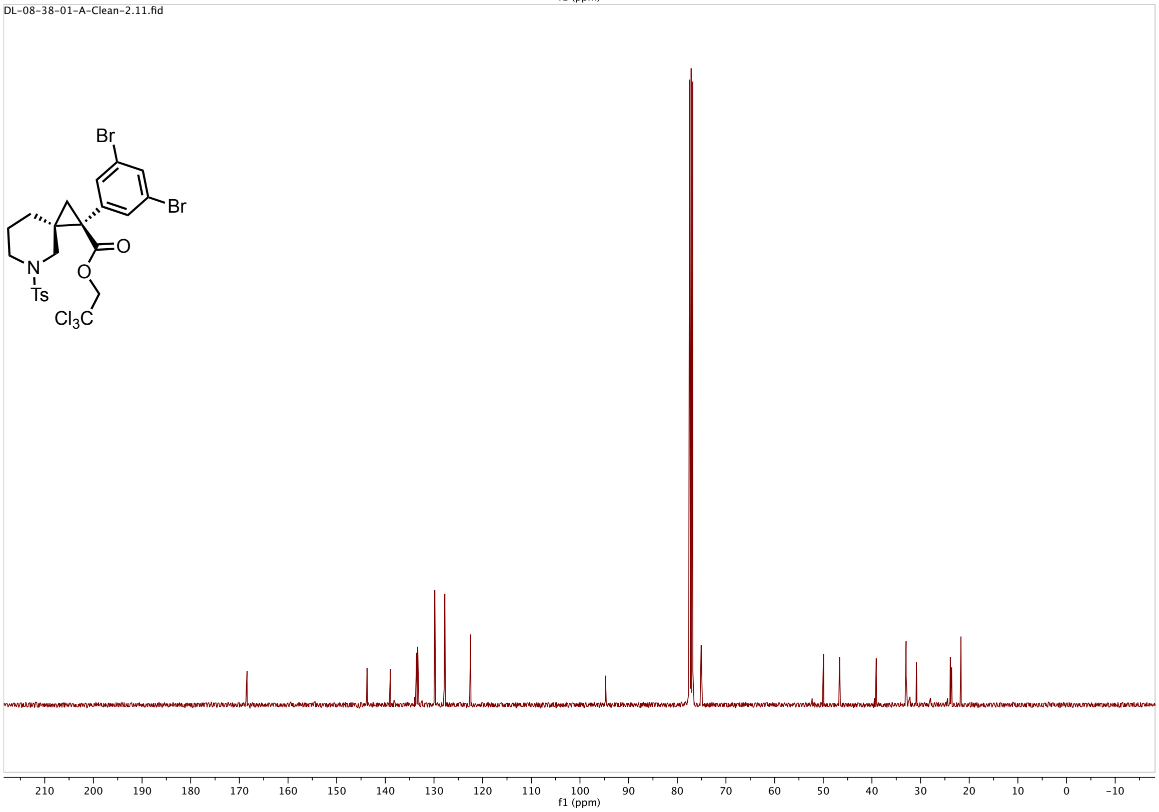
DL-08-38-01-A-Clean-2.11.fid
Br
Br
N
Ts
O
O
Cl_3C
f1 (ppm)

Compound **26**

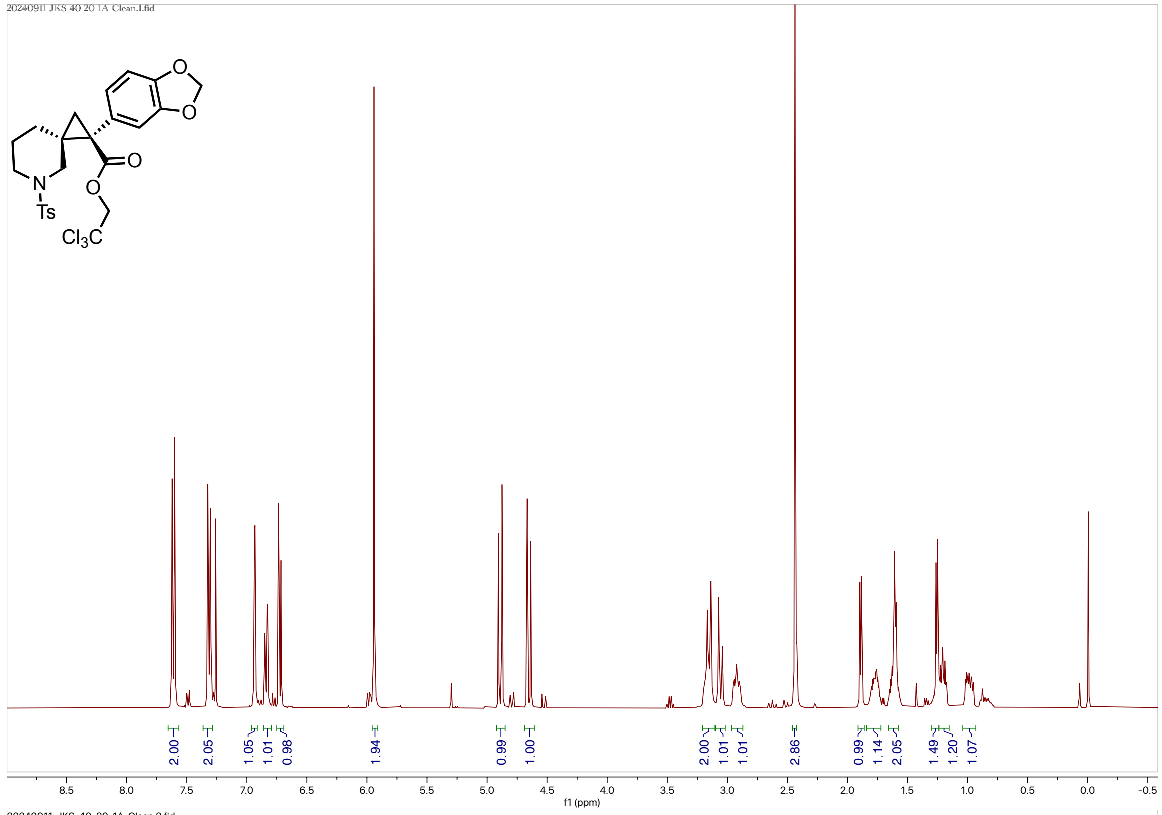
20240911-JKS-40-20-1A-Clean.1.fid
N
Ts
O
O
O
O
Cl3C
2.00
2.05
1.05
1.01
0.98
1.94
0.99
1.00
2.00
1.01
1.01
2.86
0.99
1.14
2.05
1.49
1.20
1.07
8.5
8.0
7.5
7.0
6.5
6.0
5.5
5.0
4.5
4.0
3.5
3.0
2.5
2.0
1.5
1.0
0.5
0.0
-0.5
f1 (ppm)

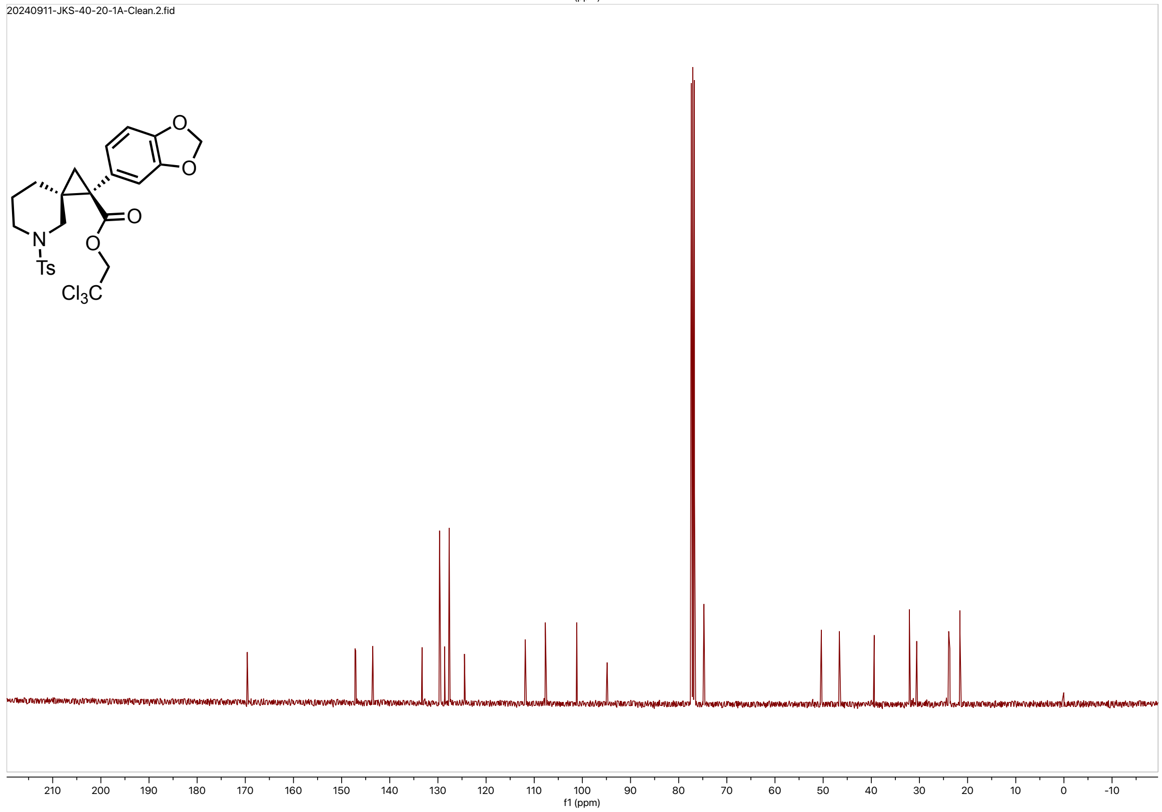
20240911-JKS-40-20-1A-Clean.2.fid
N
Ts
O
O
O
O
Cl3C
210
200
190
180
170
160
150
140
130
120
110
100
90
80
70
60
50
40
30
20
10
0
-10
f1 (ppm)

Compound **27**

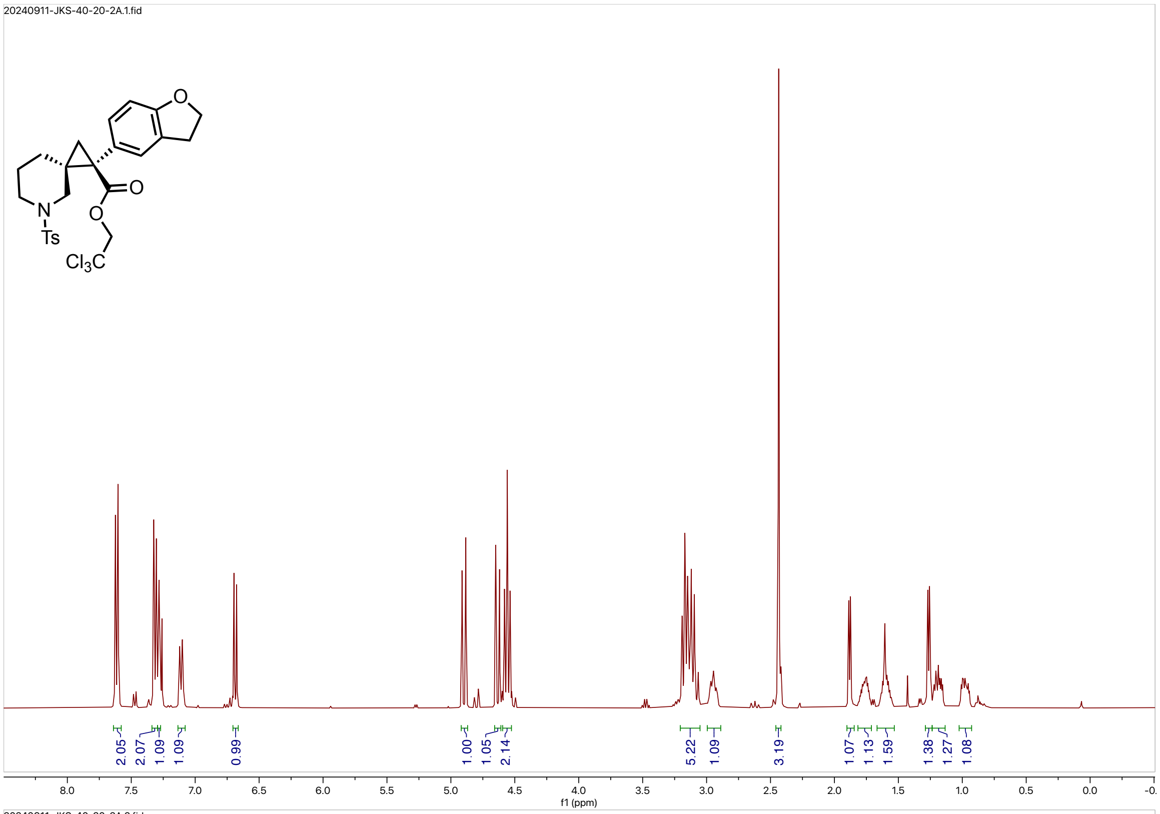
20240911-JKS-40-20-2A.1.fid
O
N
Ts
O
O
Cl_3C
2.05
2.07
1.09
1.09
0.99
1.00
1.05
2.14
5.22
1.09
3.19
1.07
1.13
1.59
1.38
1.27
1.08
f1 (ppm)

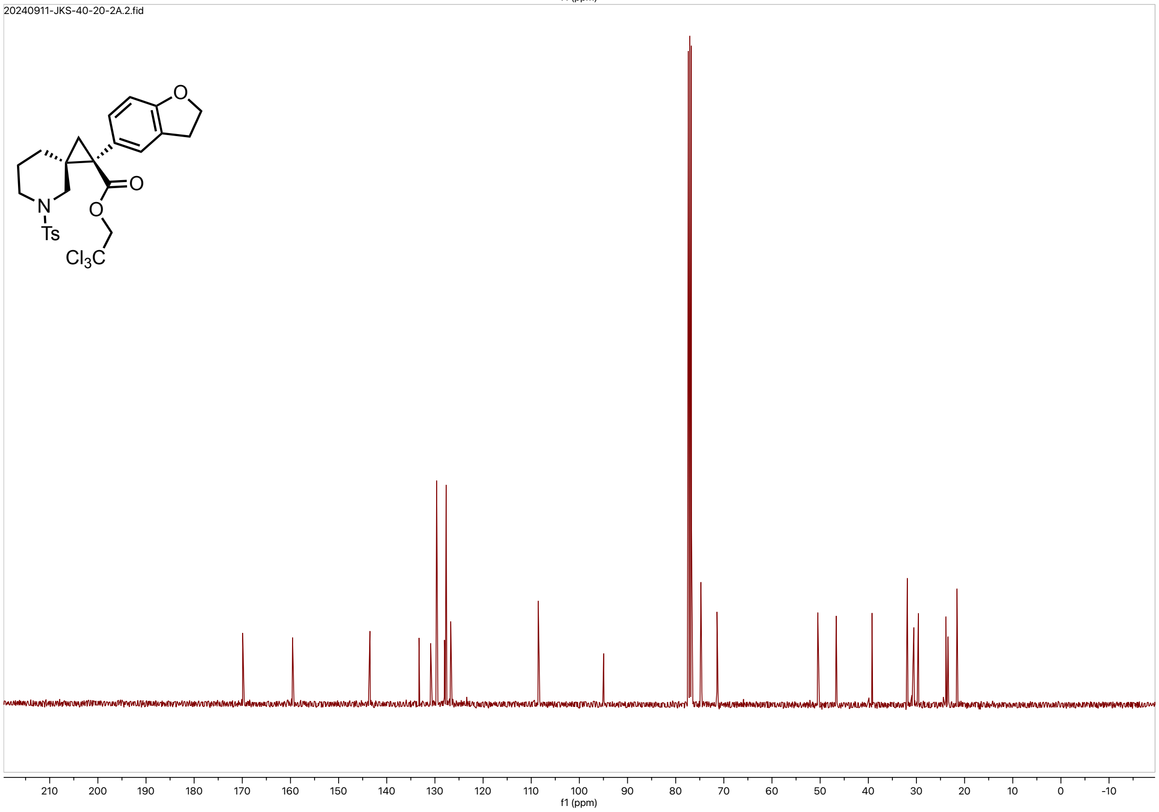
20240911-JKS-40-20-2A.2.fid
O
N
Ts
O
O
Cl_3C
f1 (ppm)

Compound **28**

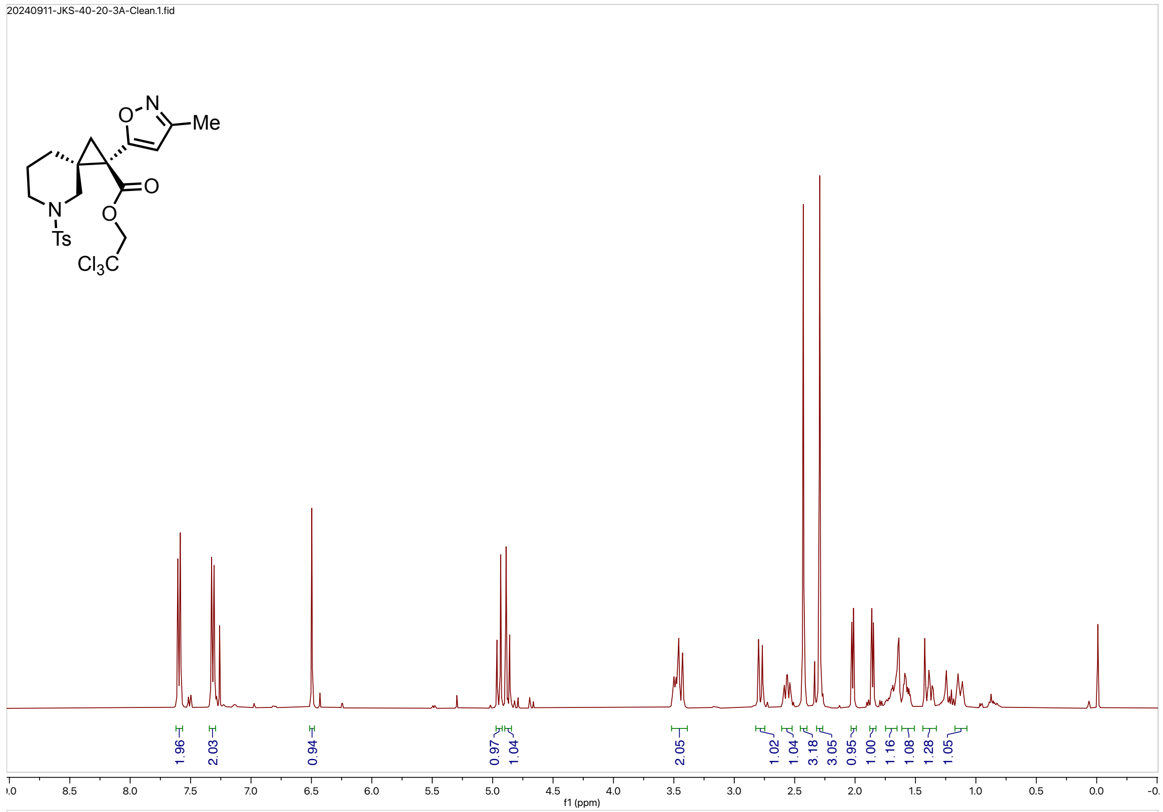
20240911-JKS-40-20-3A-Clean.1.fid
O
N
Me
N
Ts
O
O
Cl3C
1.96
2.03
0.94
0.97
1.04
2.05
1.02
1.04
3.18
3.05
0.95
1.00
1.16
1.08
1.28
1.05
f1 (ppm)

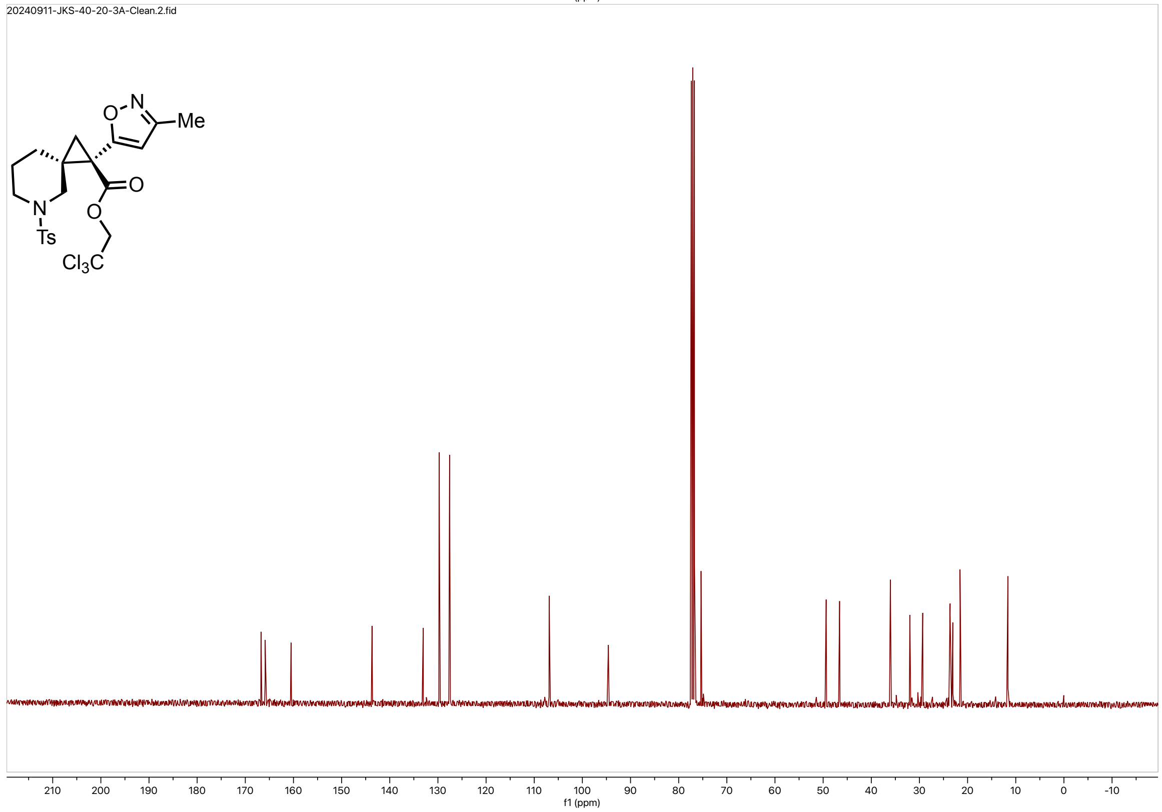
20240911-JKS-40-20-3A-Clean.2.fid
O
N
Me
N
Ts
O
O
Cl3C
f1 (ppm)

Compound **29**

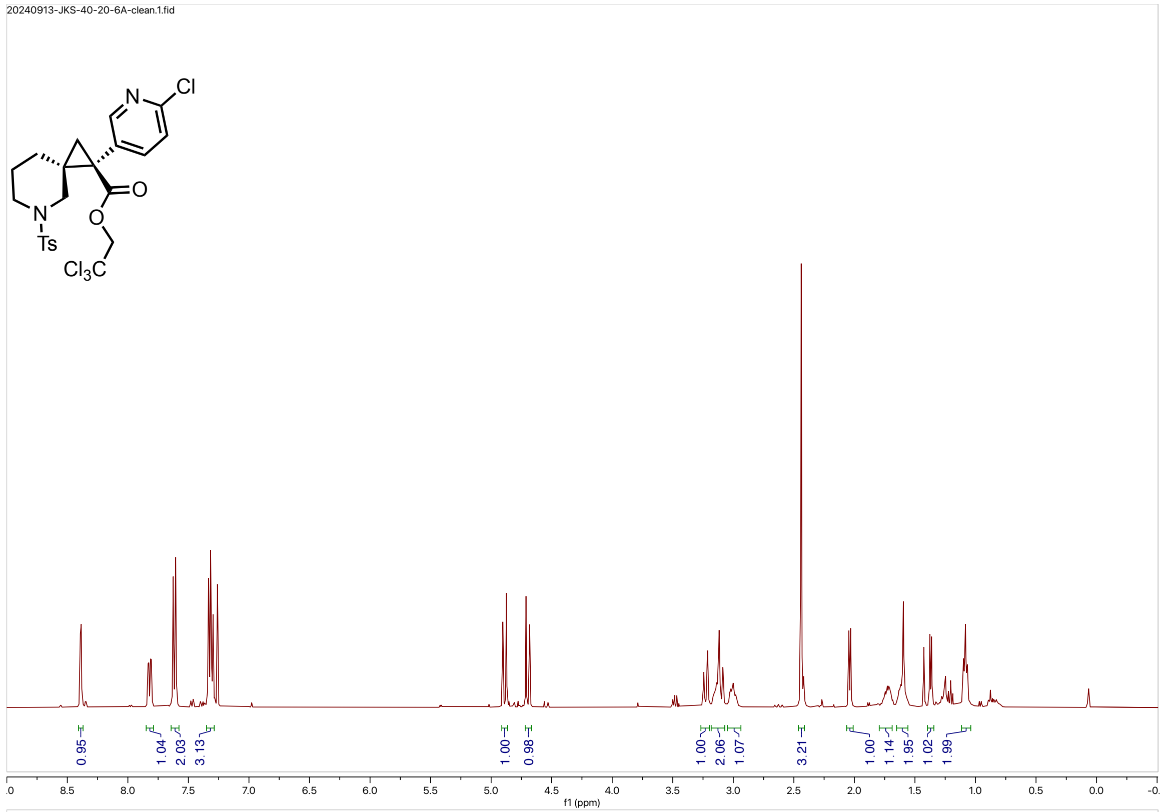
20240913-JKS-40-20-6A-clean.1.fid
Cl
N
O
O
N
Ts
Cl3C
0.95
1.04
2.03
3.13
1.00
0.98
1.00
2.06
1.07
3.21
1.00
1.14
1.95
1.02
1.99
f1 (ppm)

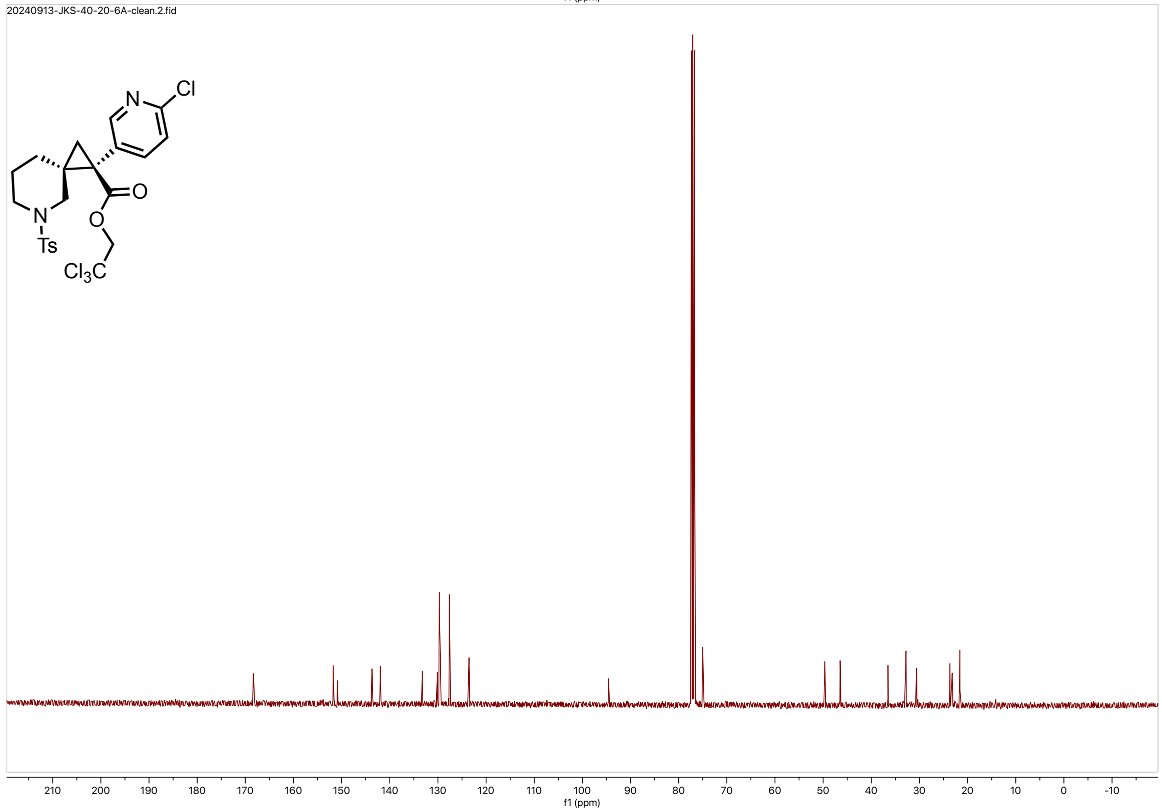
20240913-JKS-40-20-6A-clean.2.fid
Cl
N
O
O
N
Ts
Cl3C
f1 (ppm)

Compound **30**

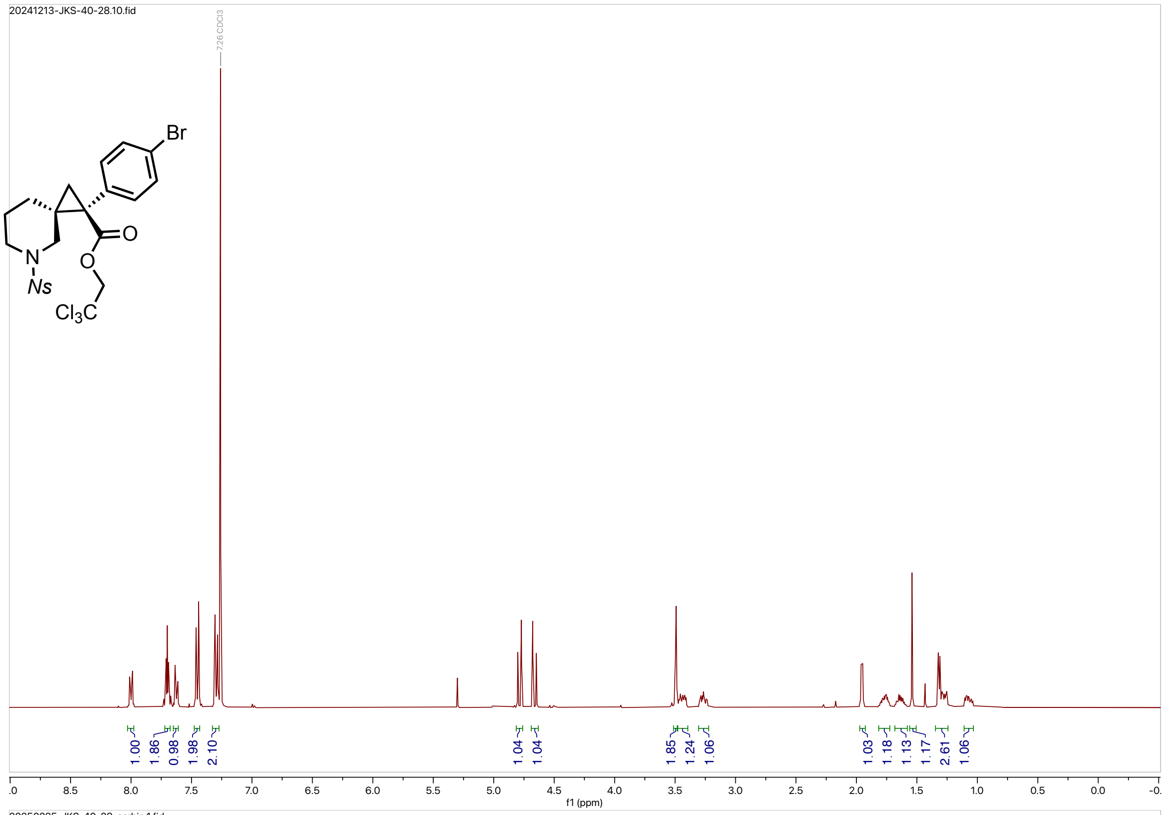
20241213-JKS-40-28.10.fid
7.26 CDCl3
Br
O
N
O
Ns
Cl3C
1.00
1.86
0.98
1.98
2.10
1.04
1.04
1.85
1.24
1.06
1.03
1.18
1.13
1.17
2.61
1.06
f1 (ppm)

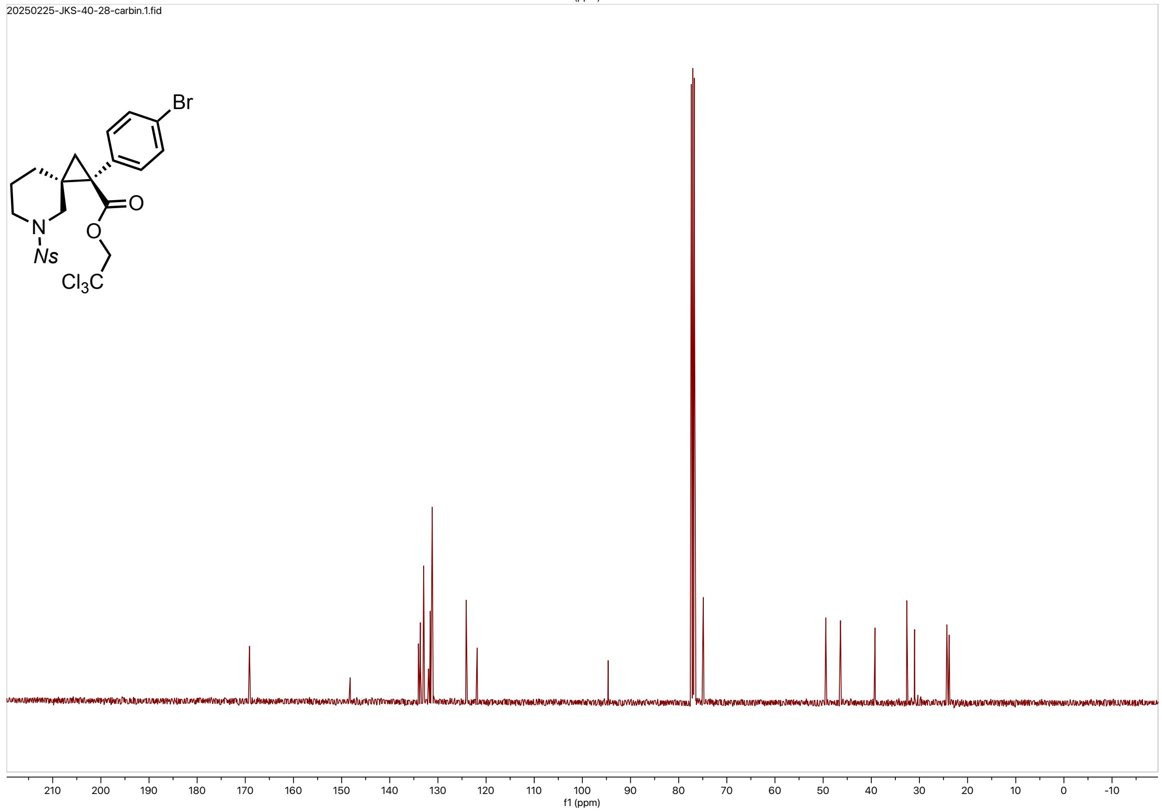
20250225-JKS-40-28-carbin.1.fid
Br
O
N
O
Ns
Cl3C
f1 (ppm)

Compound **31**

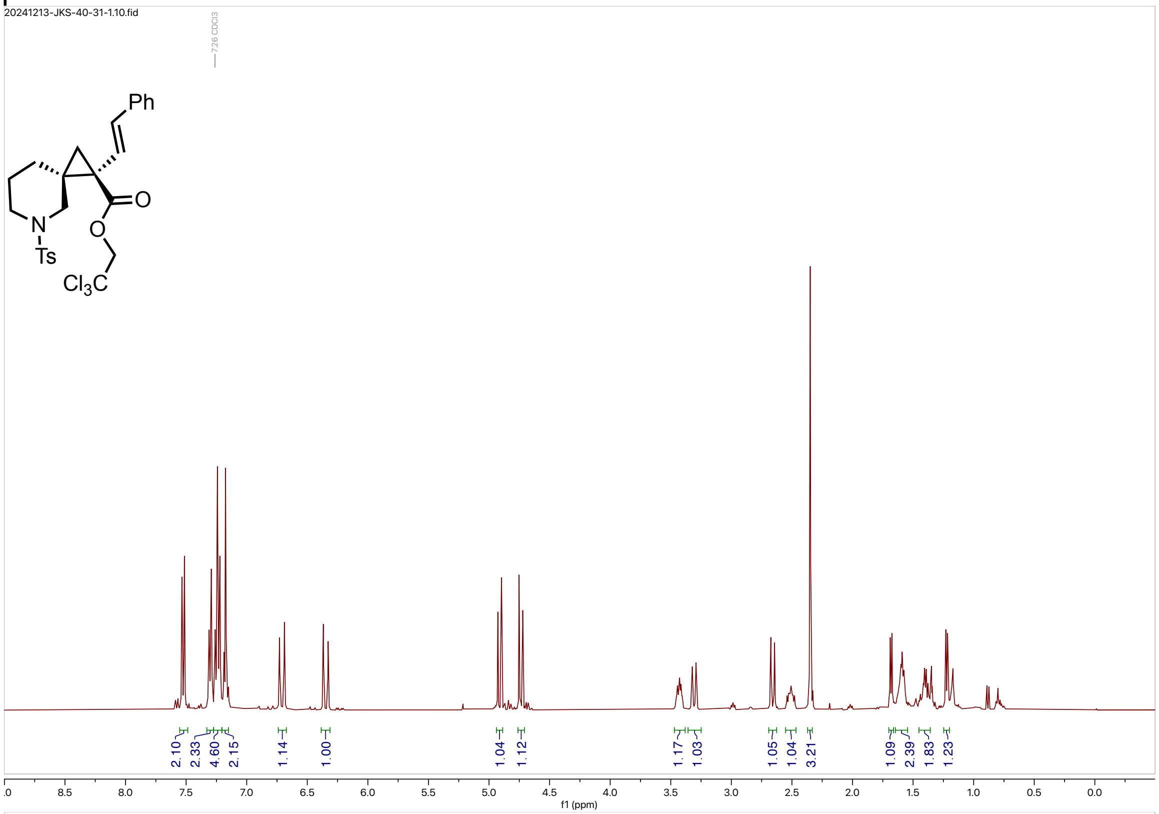
20241213-JKS-40-31-1.10.fid
Ph
O
N
Ts
Cl_3C
2.10
2.33
4.60
2.15
1.14
1.00
1.04
1.12
1.17
1.03
1.05
1.04
3.21
1.09
2.39
1.83
1.23
f1 (ppm)

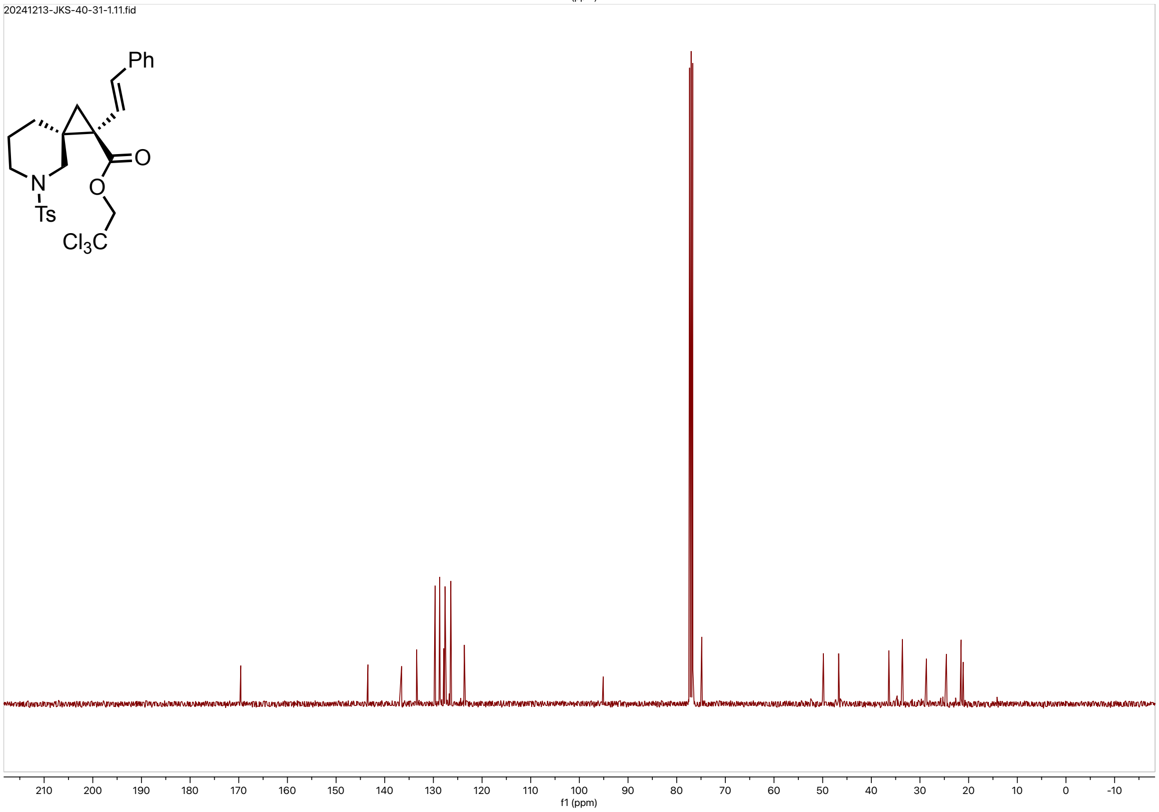
20241213-JKS-40-31-1.11.fid
Ph
O
N
Ts
Cl_3C
f1 (ppm)

Compound **32**

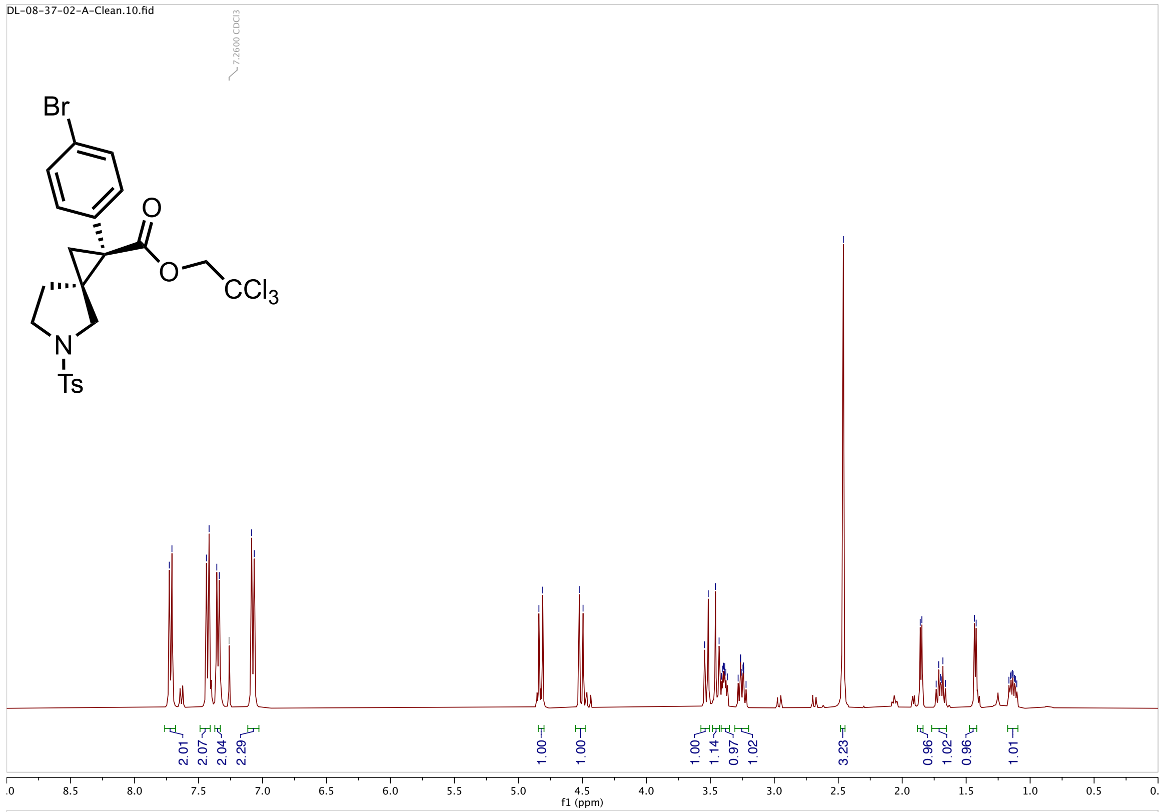
DL-08-37-02-A-Clean.10.fid
7.2600 CDCl3
Br
O
O
CCl3
N
Ts
2.01
2.07
2.04
2.29
1.00
1.00
1.00
1.14
0.97
1.02
3.23
0.96
1.02
0.96
1.01
f1 (ppm)

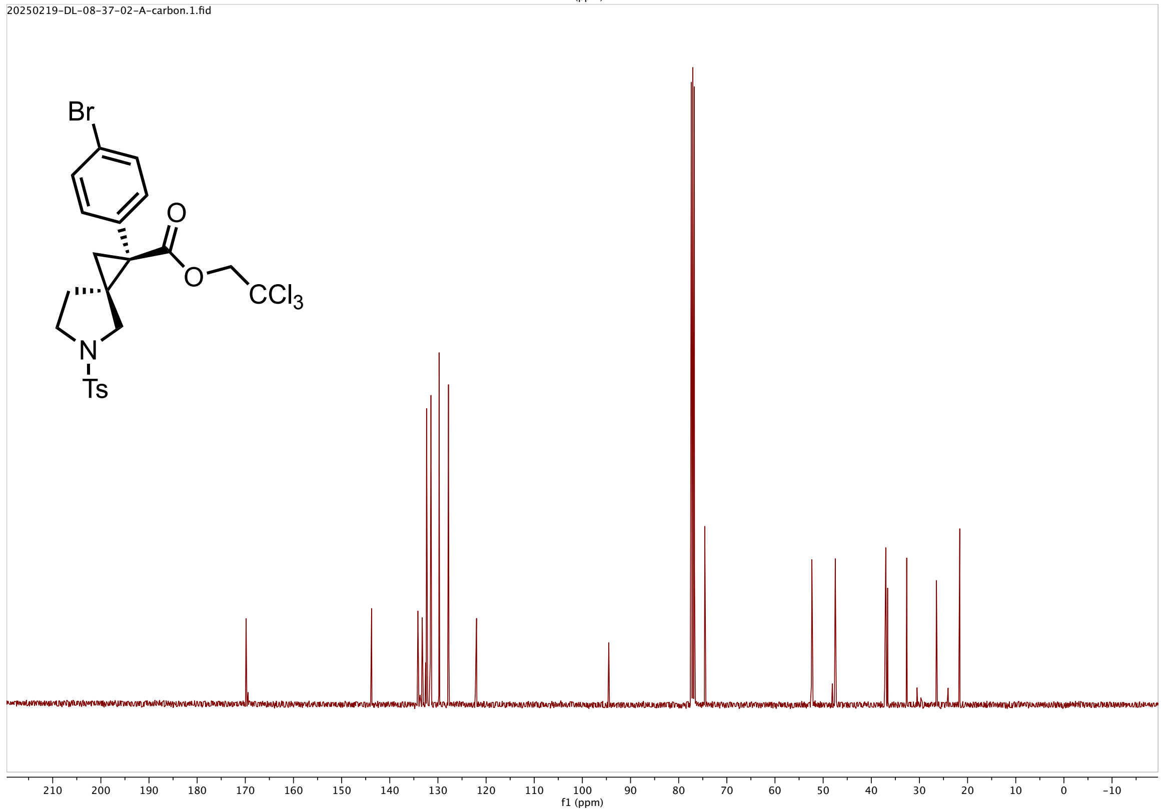
20250219-DL-08-37-02-A-carbon.1.fid
Br
O
O
CCl3
N
Ts
f1 (ppm)

Compound **34**

20250722-DL-08-81-03-A-Clean.10.fid

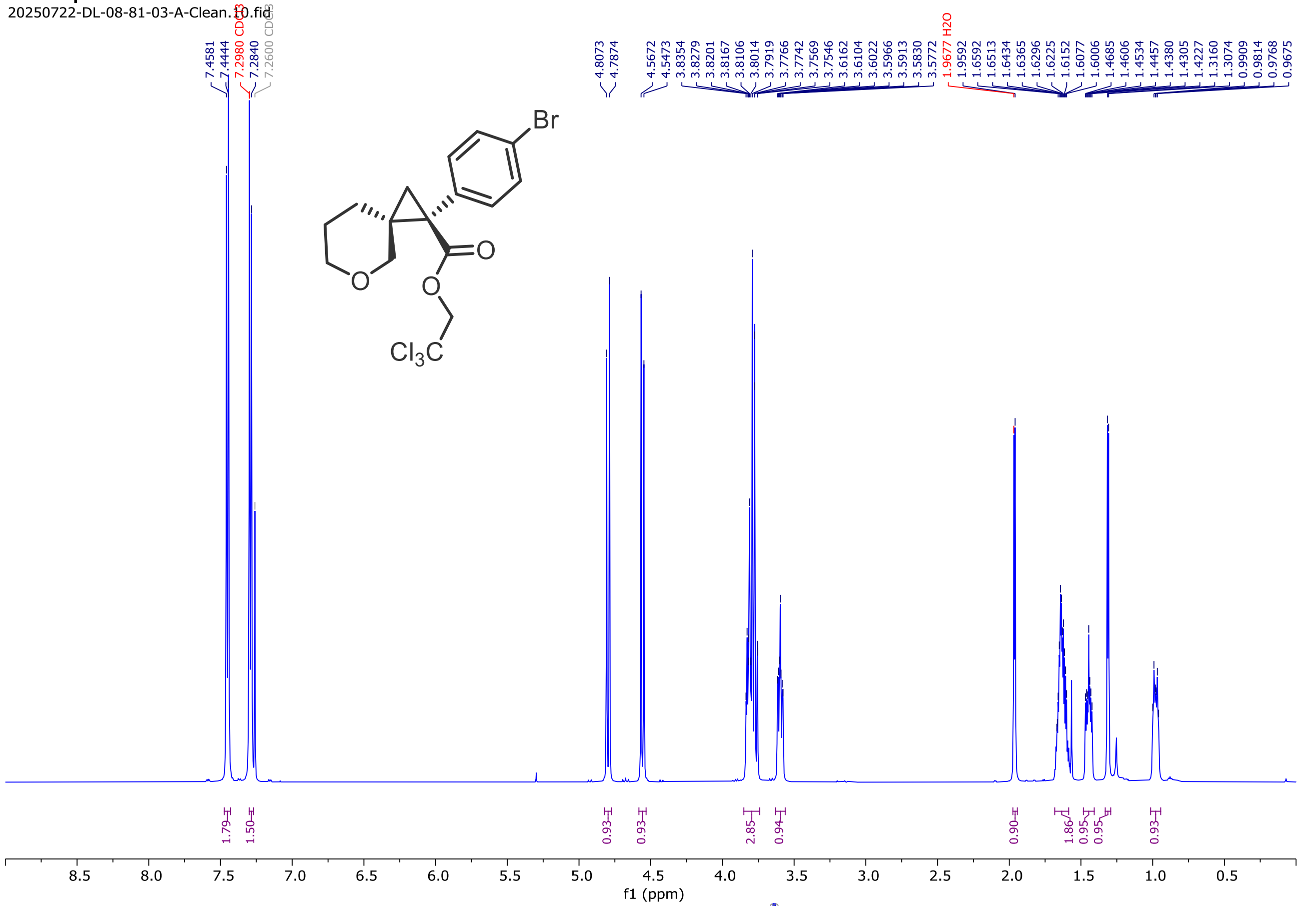

20250722-DL-08-81-03-A-Clean.11.fid

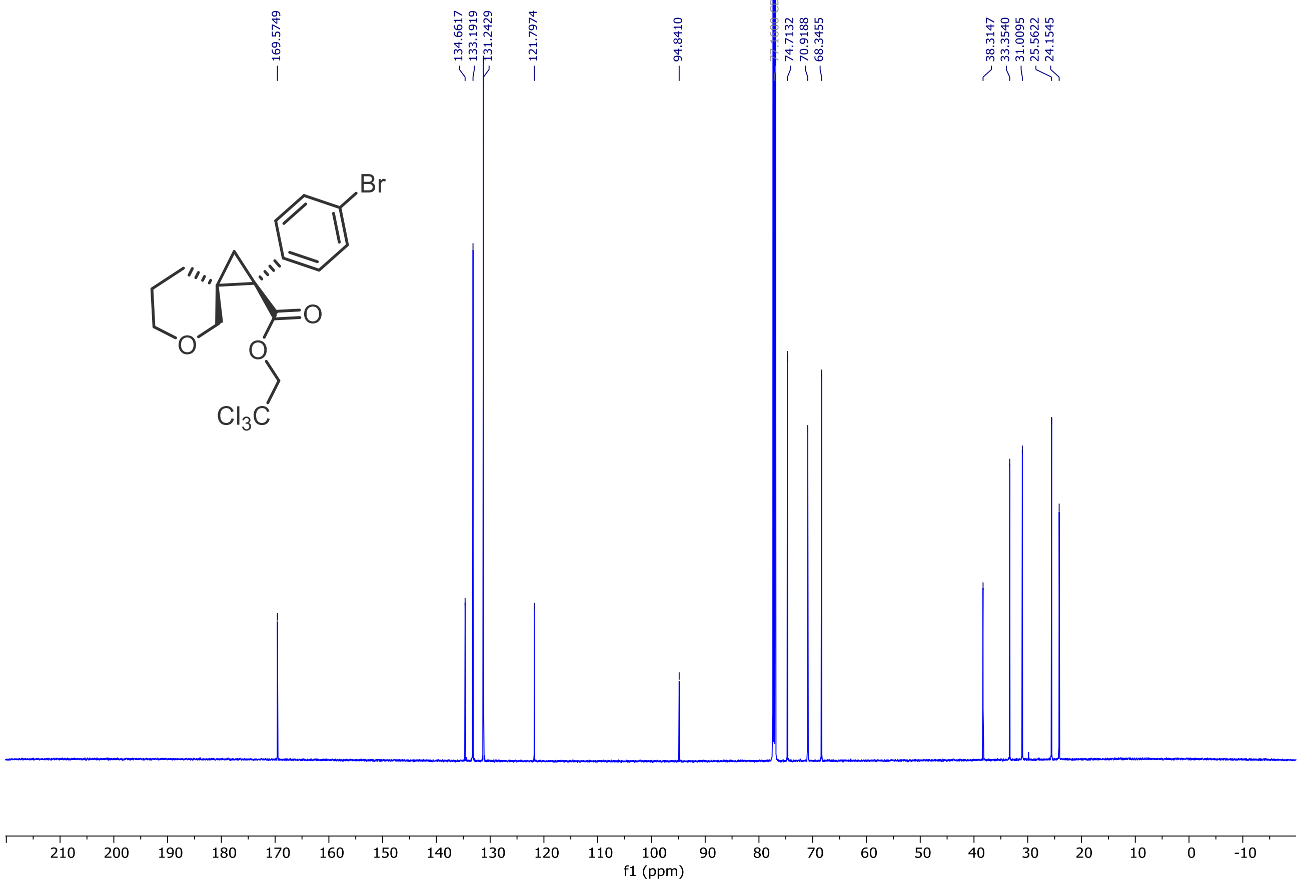

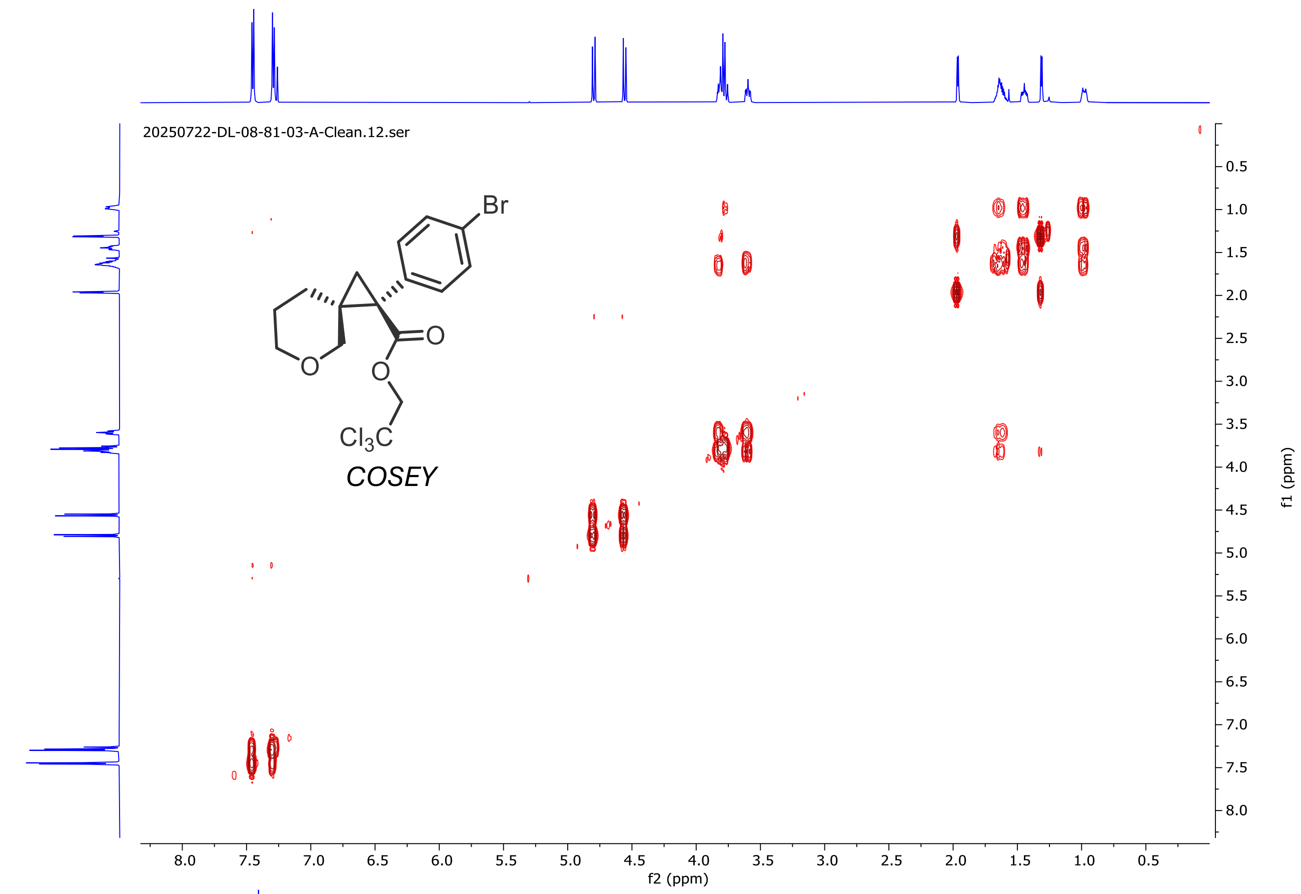
20250722-DL-08-81-03-A-Clean.12.ser
Br
O
O
O
Cl3C
COSEY
f2 (ppm)
f1 (ppm)

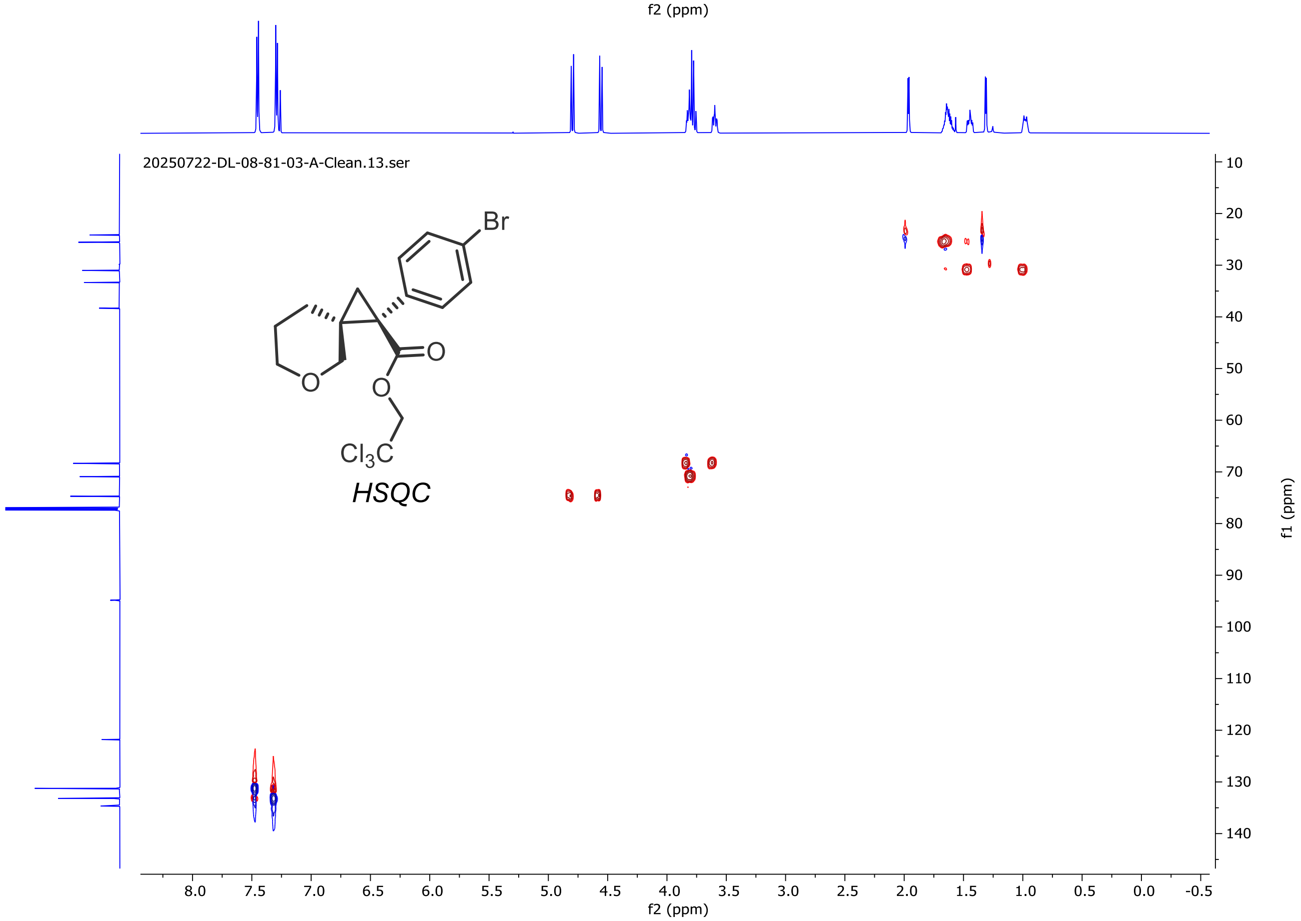
20250722-DL-08-81-03-A-Clean.13.ser
Br
O
O
O
Cl3C
HSQC
f2 (ppm)
f1 (ppm)

Compound **35**

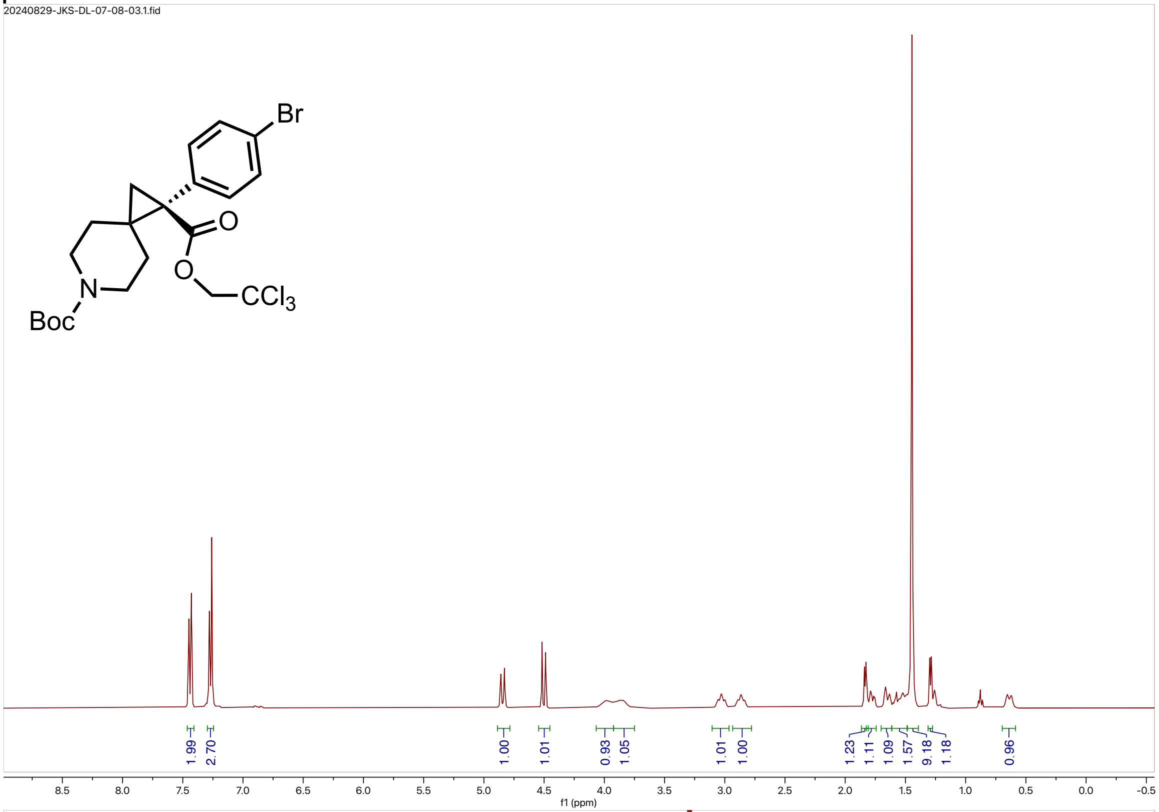
20240829-JKS-DL-07-08-03.1.fid
Br
O
O
CCl3
N
Boc
1.99
2.70
1.00
1.01
0.93
1.05
1.01
1.00
1.23
1.11
1.09
1.57
9.18
1.18
0.96
f1 (ppm)

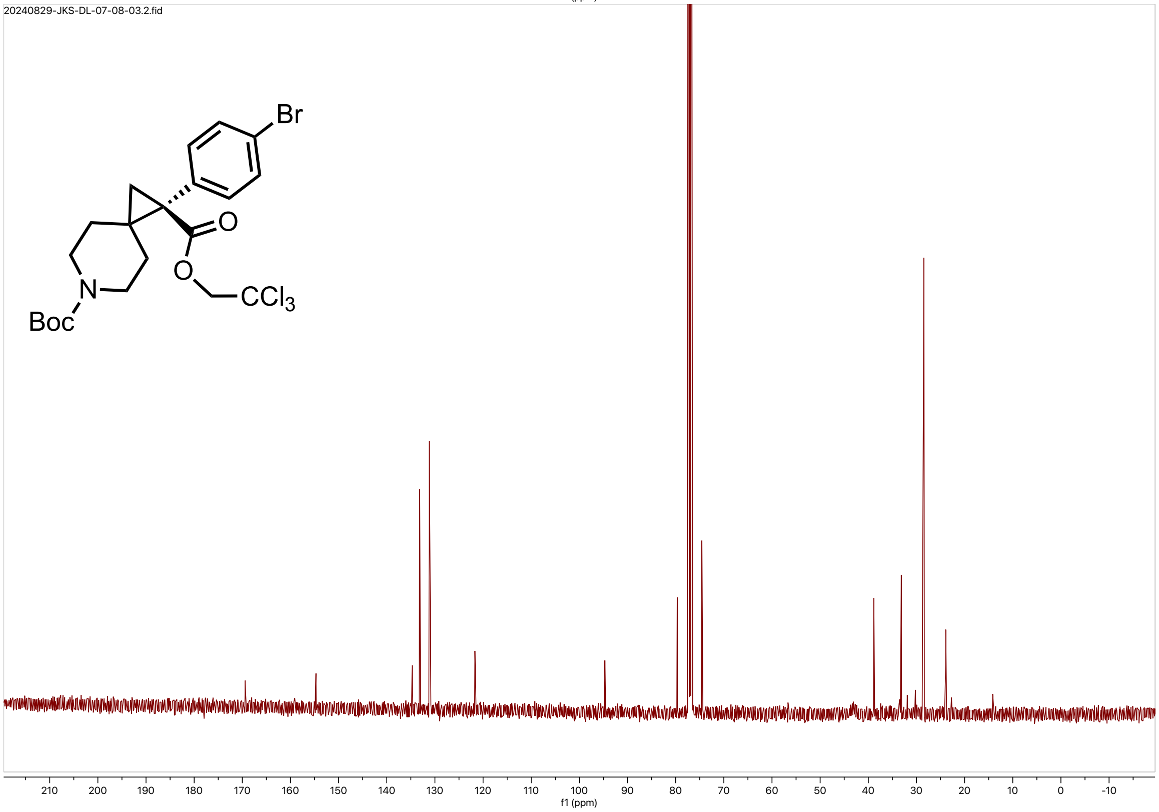
20240829-JKS-DL-07-08-03.2.fid
Br
O
O
CCl3
N
Boc
f1 (ppm)

Compound **36**

20250507-AW-01-64-A-Clean.10.fid

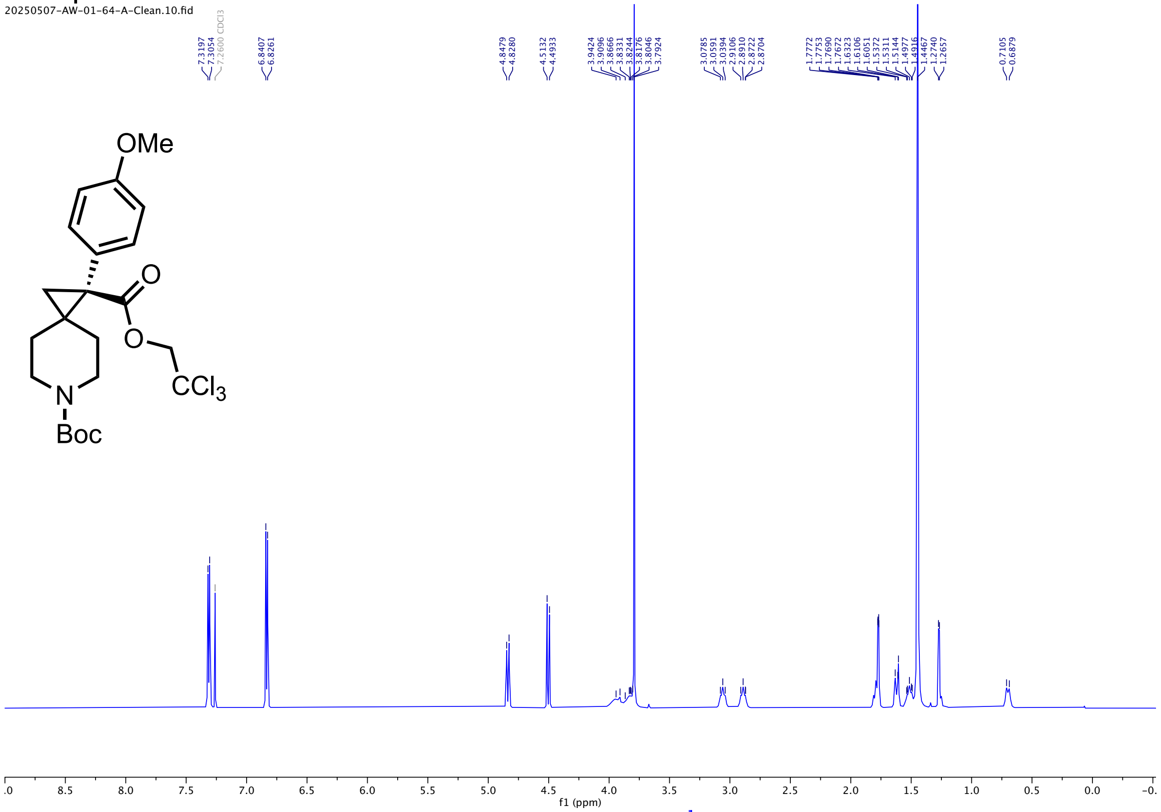

20250507-AW-01-64-A-Clean.11.fid

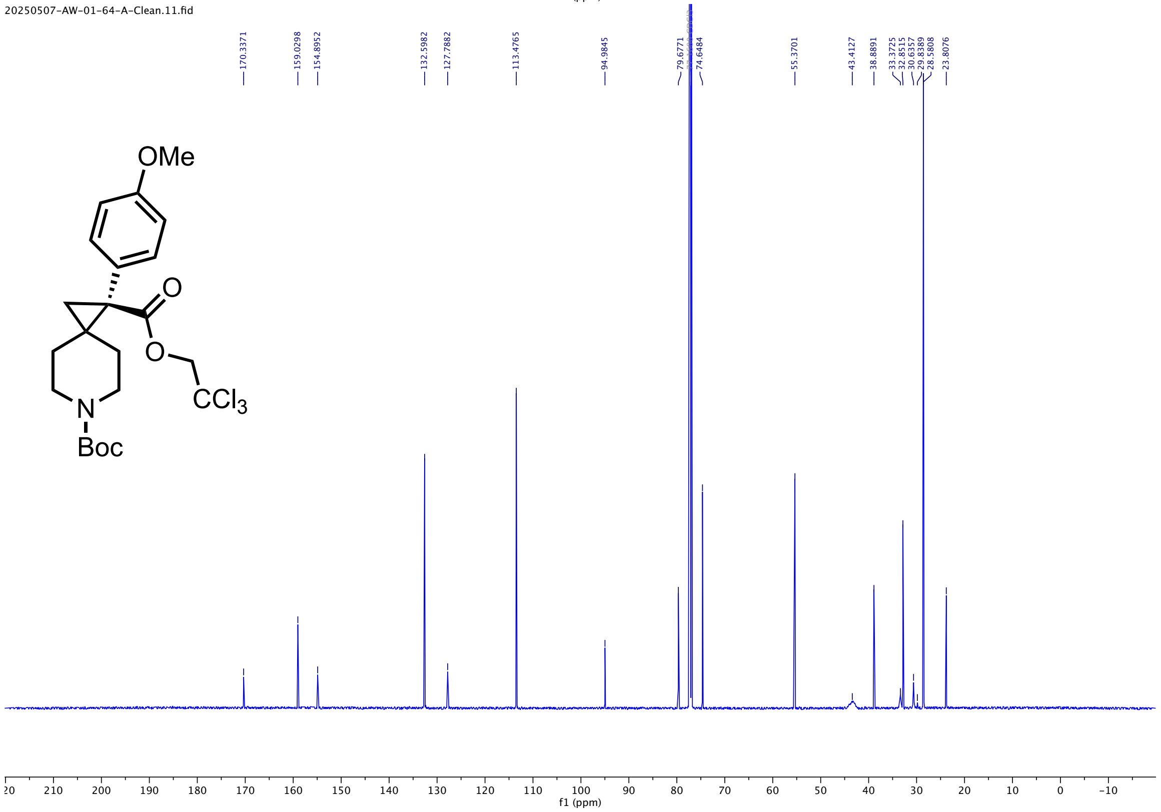

Compound **37**

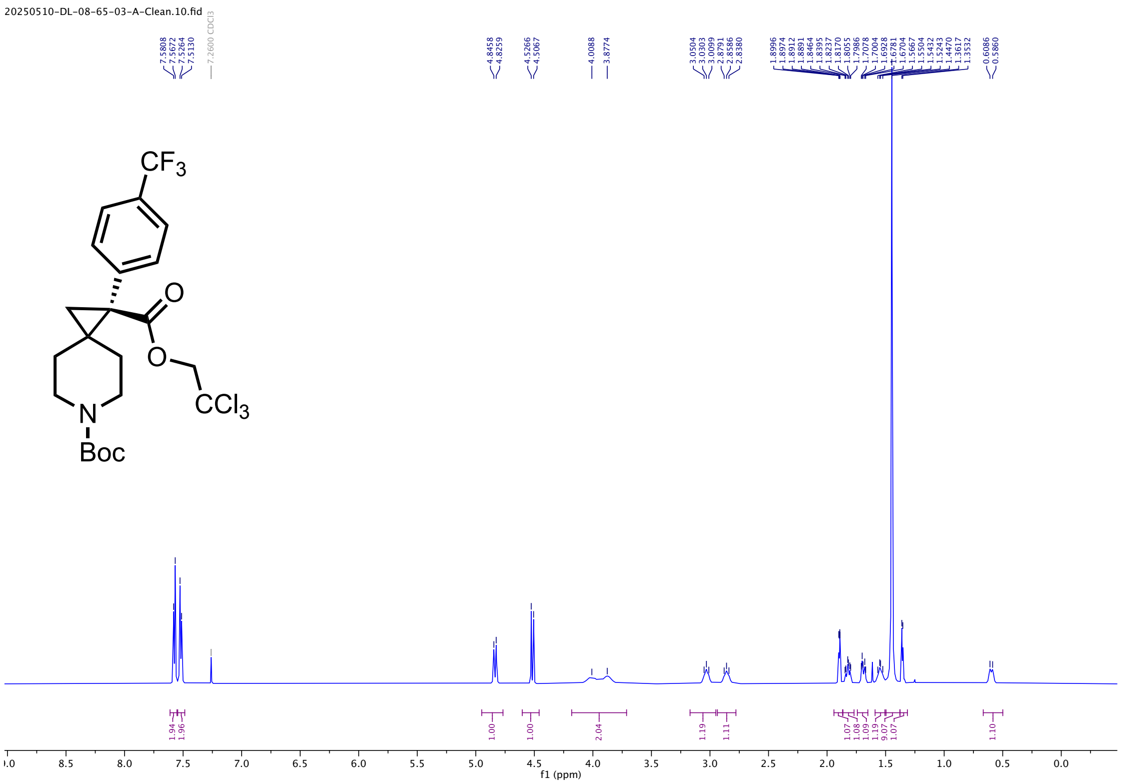
20250510-DL-08-65-03-A-Clean.10.fid
f1 (ppm)

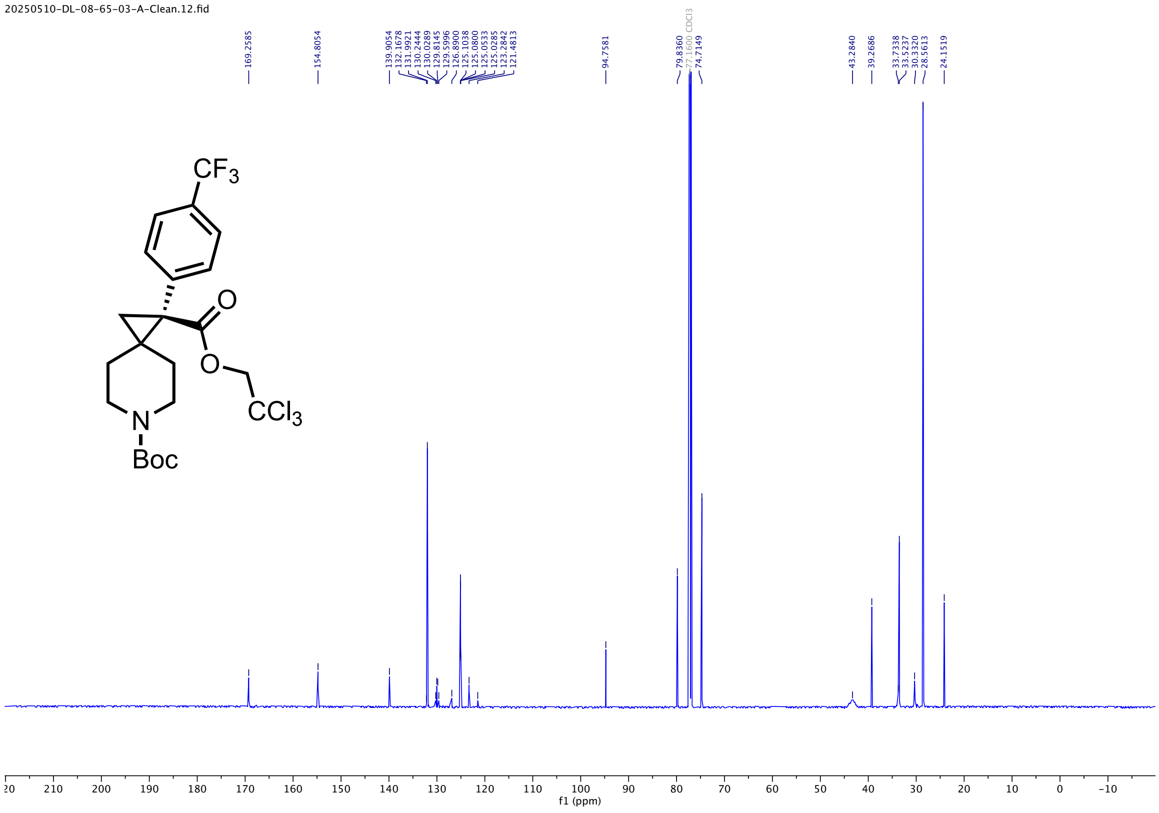
20250510-DL-08-65-03-A-Clean.12.fid
f1 (ppm)

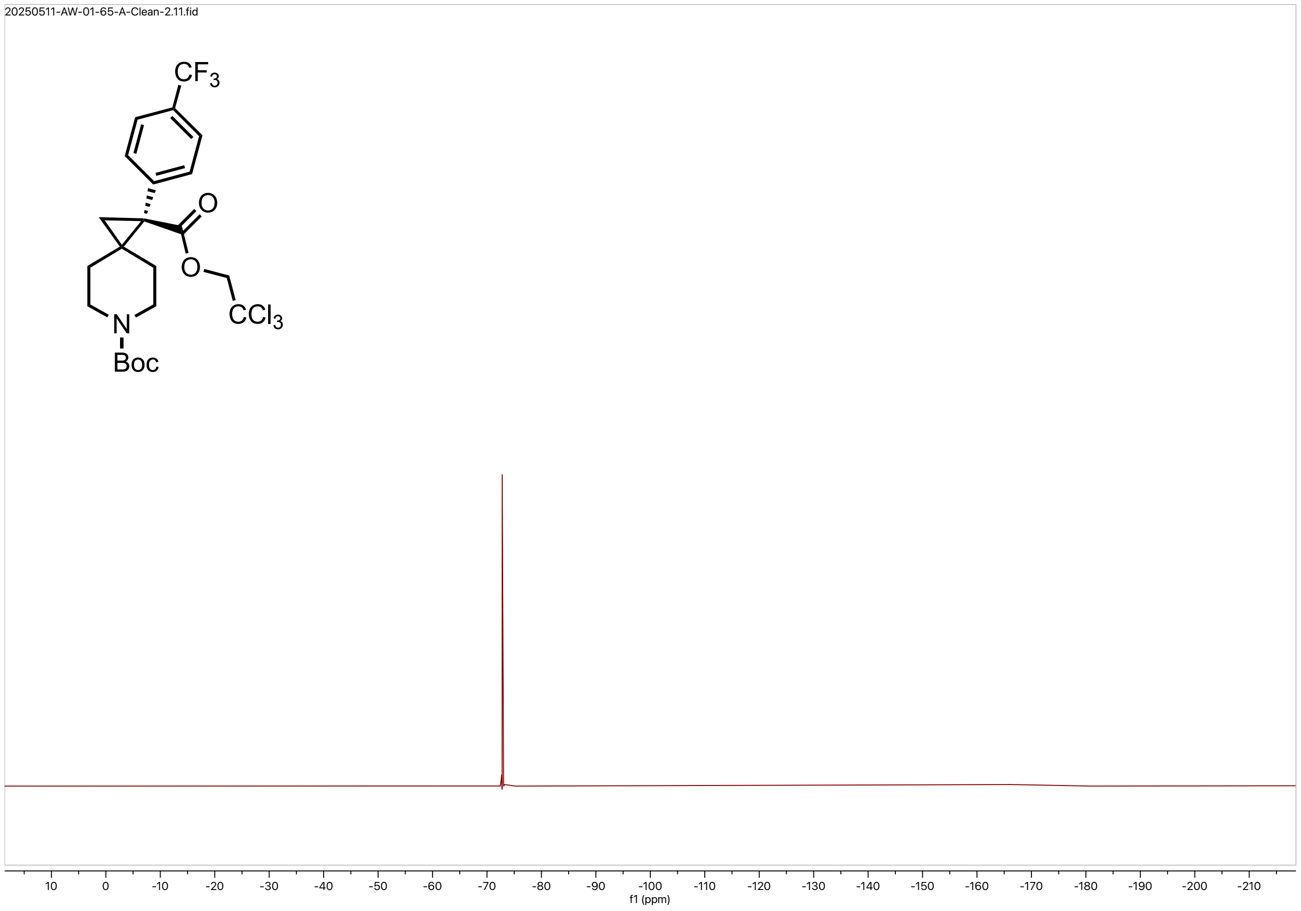

20250511-AW-01-65-A-Clean-2.11.fid
CF3
O
O
CCl3
N
Boc
10
0
-10
-20
-30
-40
-50
-60
-70
-80
-90
-100
-110
-120
-130
-140
-150
-160
-170
-180
-190
-200
-210
f1 (ppm)

Compound **38**

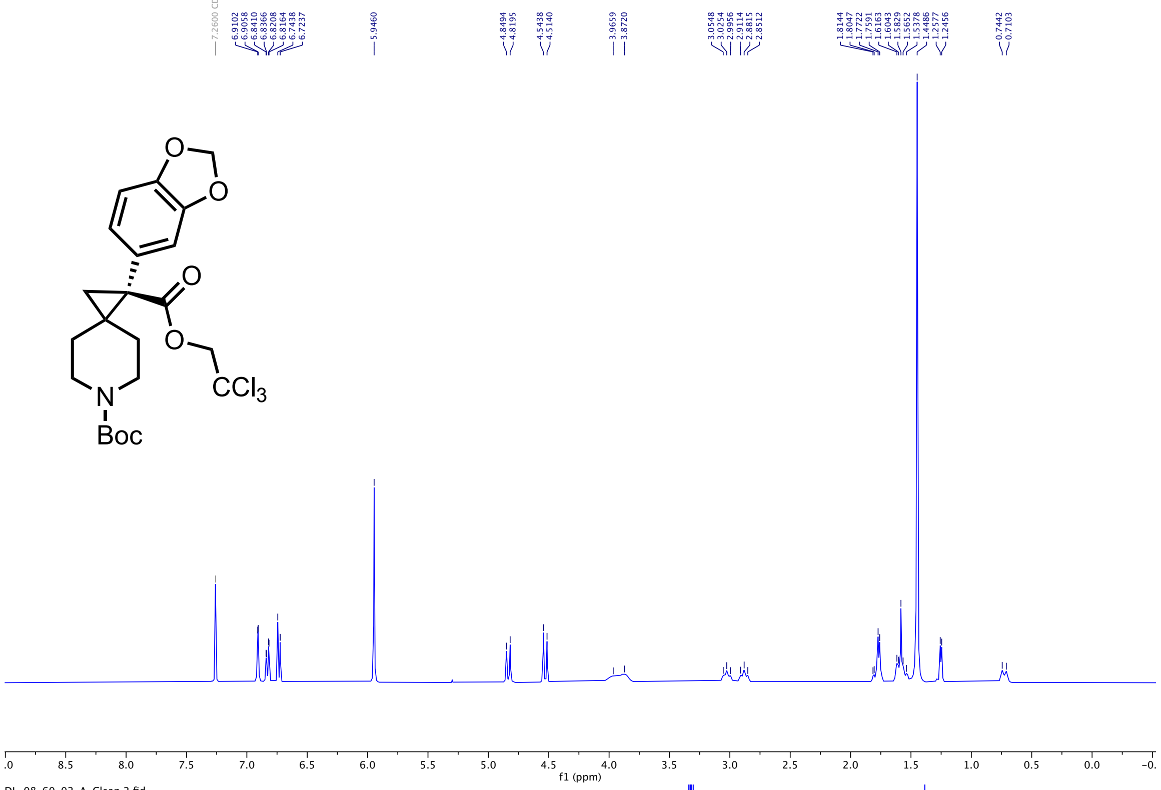
DL-08-60-02-A-Clean.1.fid
7.2600 CDCl3
6.9102
6.9058
6.8410
6.8366
6.8208
6.8164
6.7438
6.7237
5.9460
4.8494
4.8195
4.5438
4.5140
3.9659
3.8720
3.0548
3.0254
2.9956
2.9114
2.8815
2.8512
1.8144
1.8047
1.7722
1.7591
1.6163
1.6043
1.5829
1.5652
1.5378
1.4486
1.2577
1.2456
0.7442
0.7103
O
O
O
O
CCl3
N
Boc
f1 (ppm)

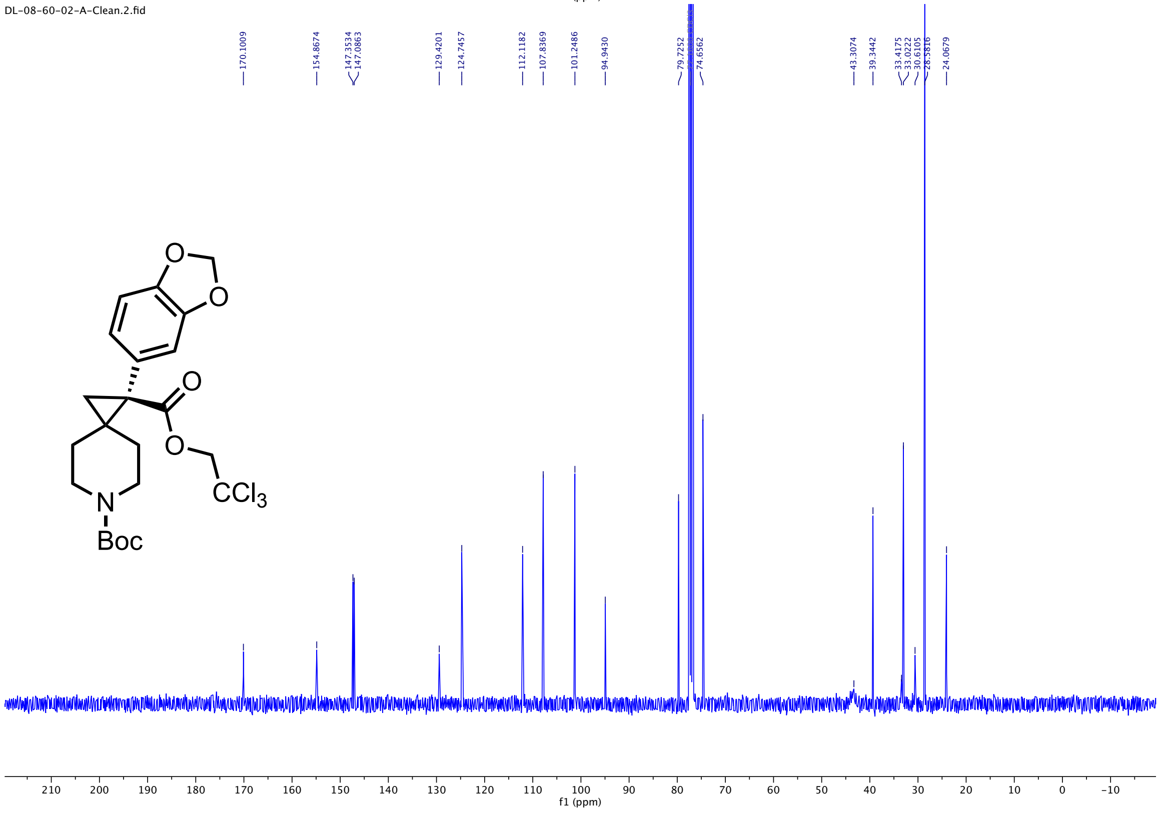
DL-08-60-02-A-Clean.2.fid
170.1009
154.8674
147.3534
147.0863
129.4201
124.7457
112.1182
107.8369
101.2486
94.9430
79.7252
74.6562
43.3074
39.3442
33.4175
33.0222
30.6105
28.3816
24.0679
O
O
O
O
CCl3
N
Boc
f1 (ppm)

Compound **39**

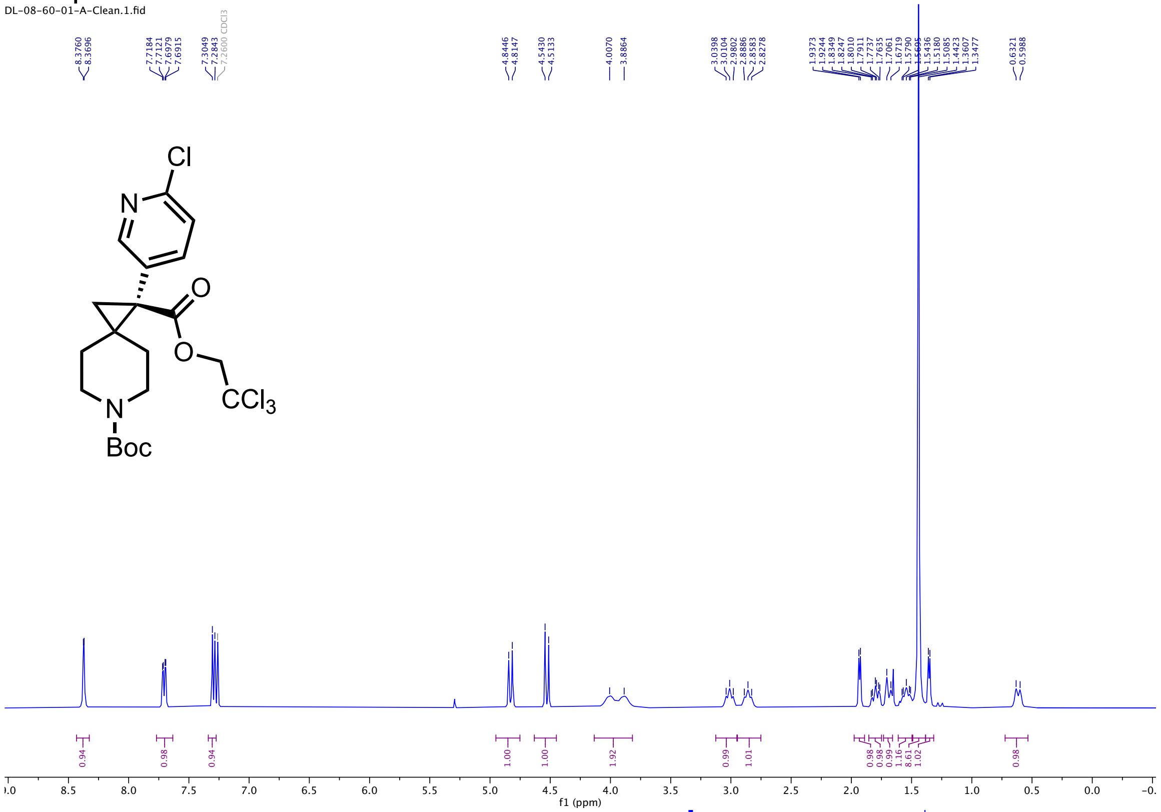

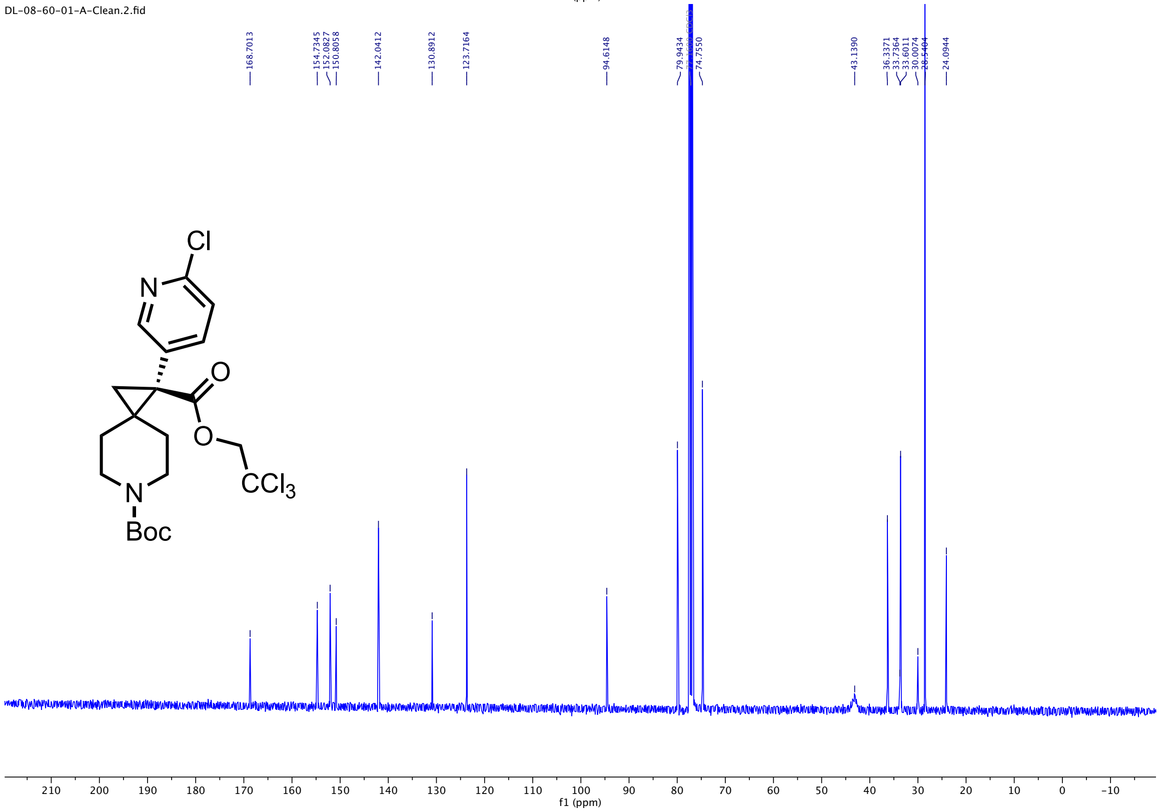

Compound **40**

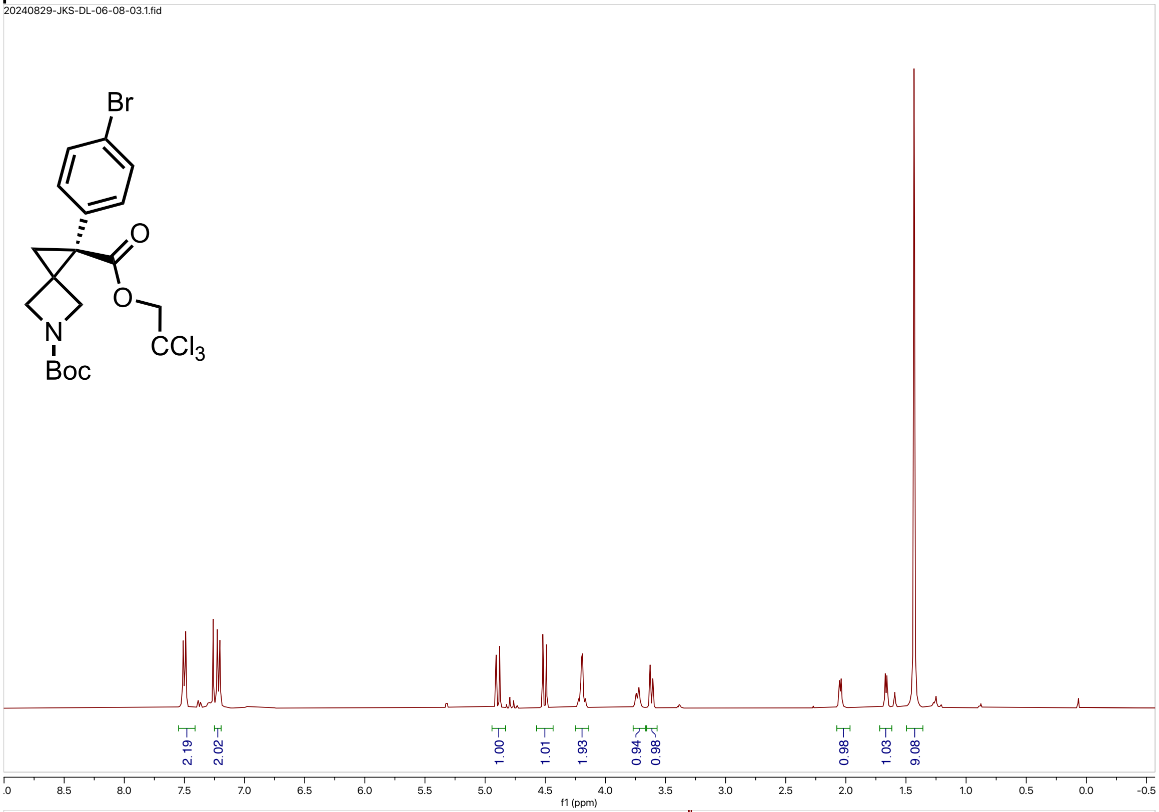

20240829-JKS-DL-06-08-03.1.fid
Br
O
O
N
Boc
CCl3
2.19
2.02
1.00
1.01
1.93
0.94
0.98
0.98
1.03
9.08
f1 (ppm)

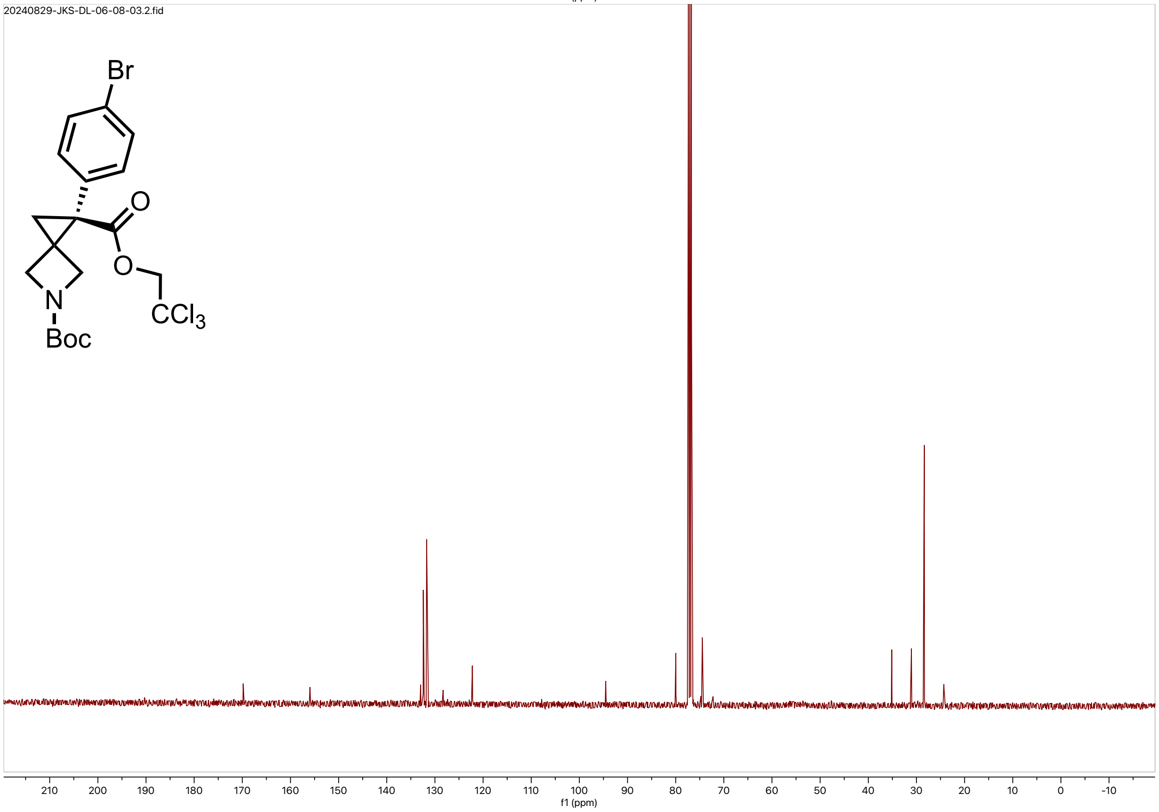

20240829-JKS-DL-06-08-03.2.fid
Br
O
O
N
Boc
CCl3
f1 (ppm)

Compound **41**

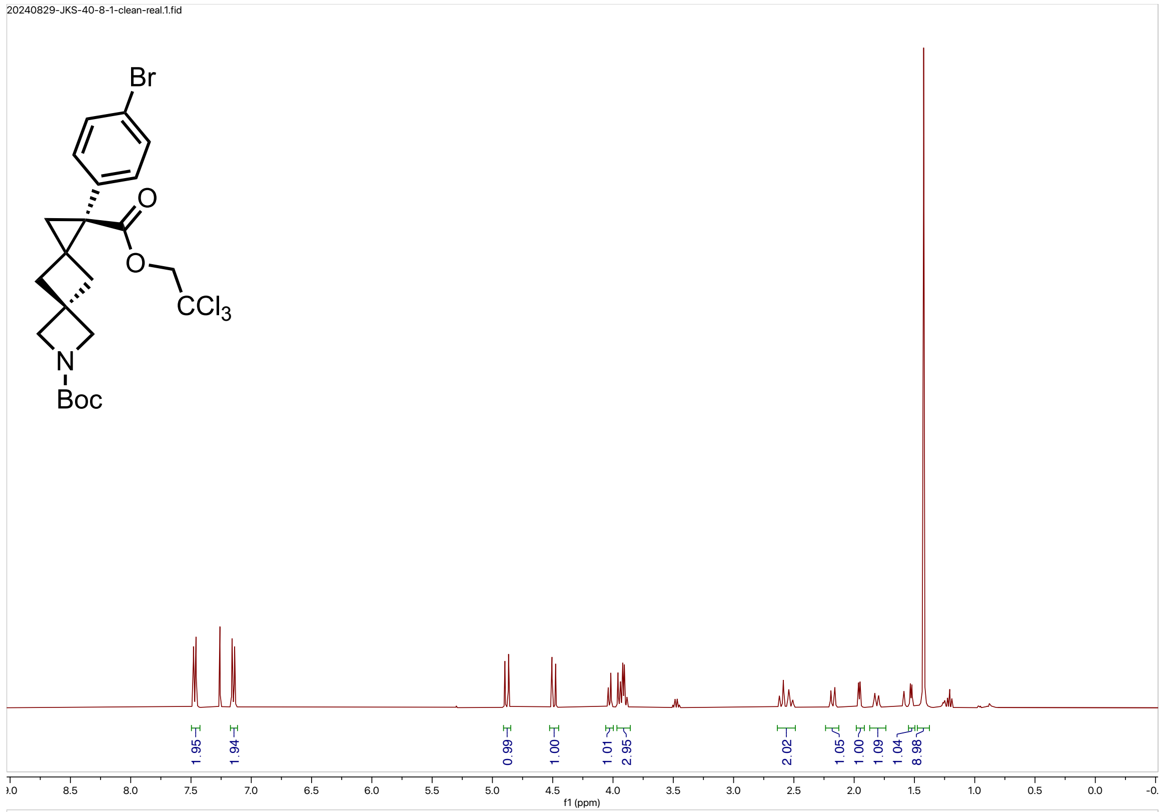
20240829-JKS-40-8-1-clean-real.1.fid
Br
O
O
CCl3
N
Boc
1.95
1.94
0.99
1.00
1.01
2.95
2.02
1.05
1.00
1.09
1.04
8.98
f1 (ppm)

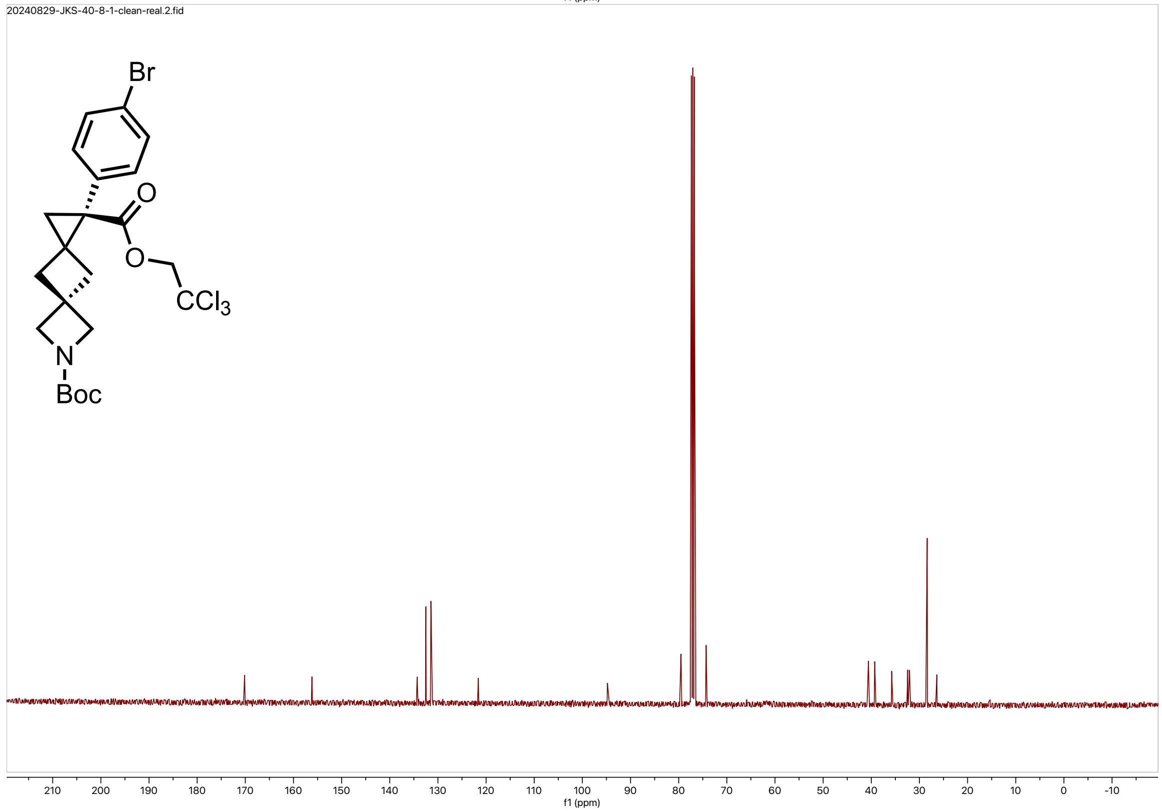
20240829-JKS-40-8-1-clean-real.2.fid
Br
O
O
CCl3
N
Boc
f1 (ppm)

Compound **42**

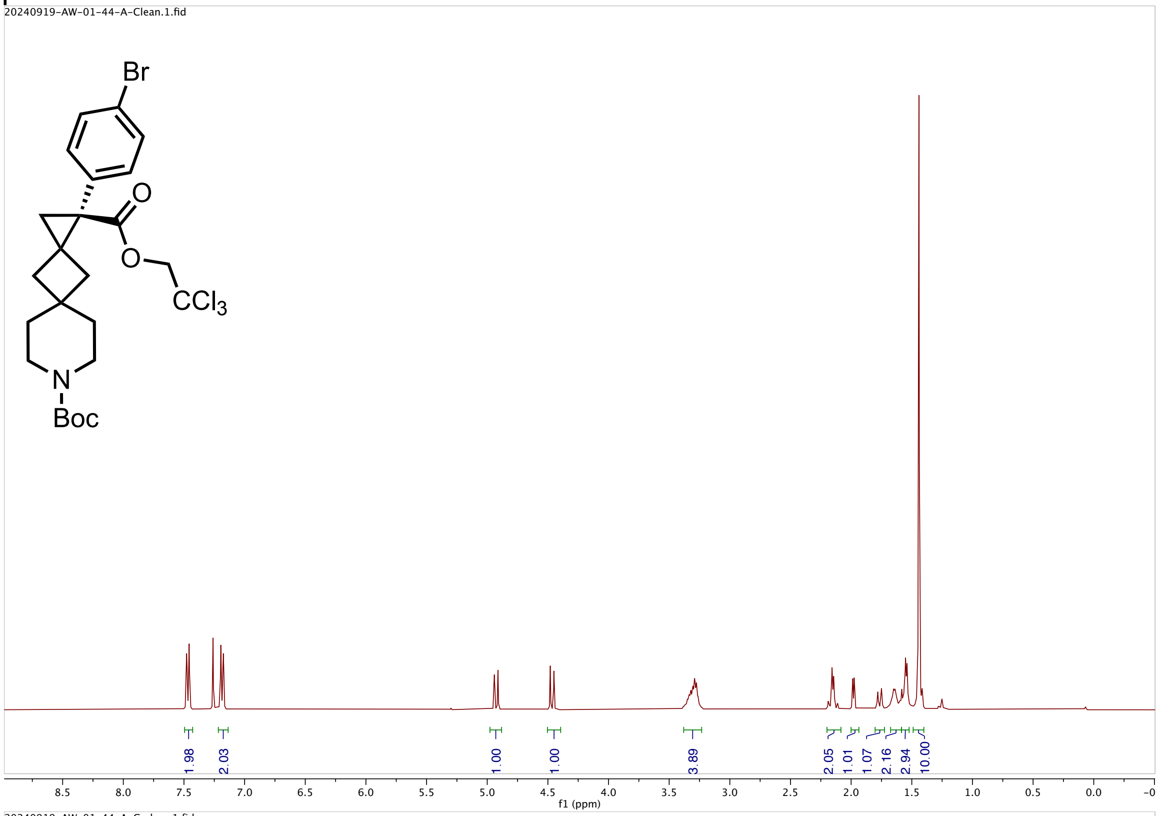
20240919-AW-01-44-A-Clean.1.fid
Br
O
O
CCl3
N
Boc
1.98
2.03
1.00
1.00
3.89
2.05
1.01
1.07
2.16
2.94
10.00
f1 (ppm)

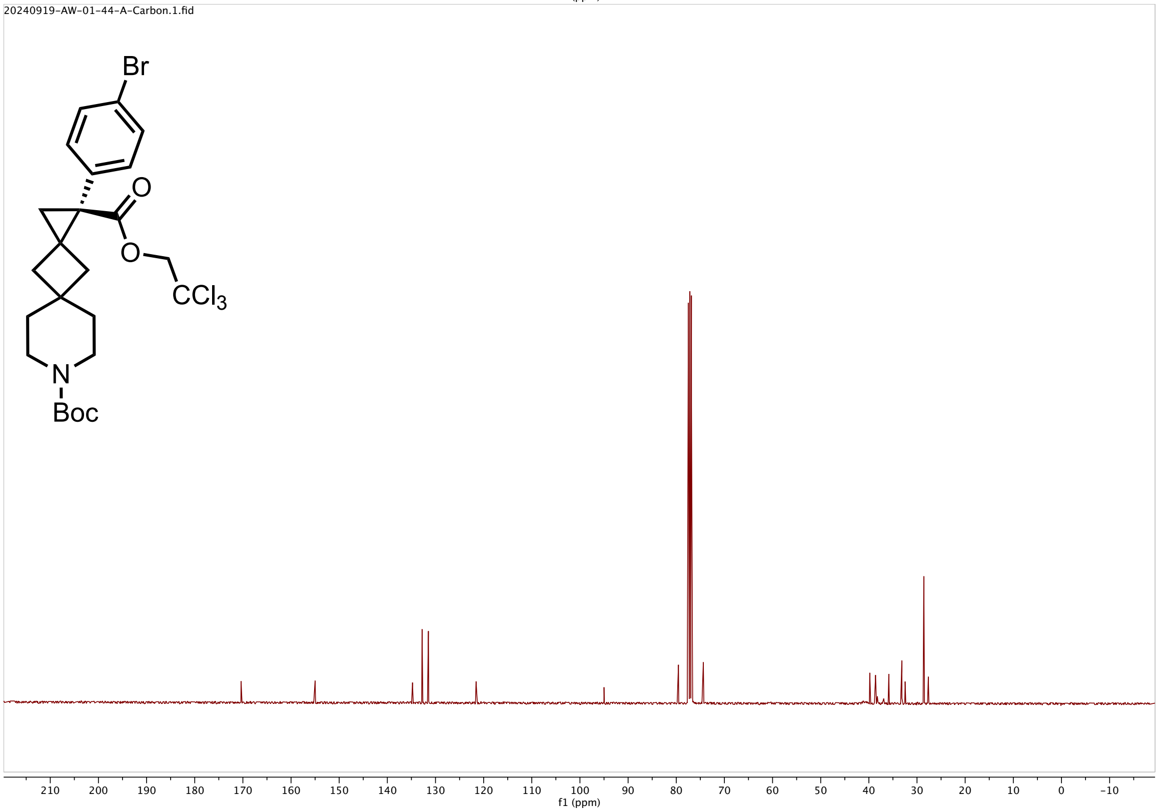
20240919-AW-01-44-A-Carbon.1.fid
Br
O
O
CCl3
N
Boc
f1 (ppm)

Compound **43**

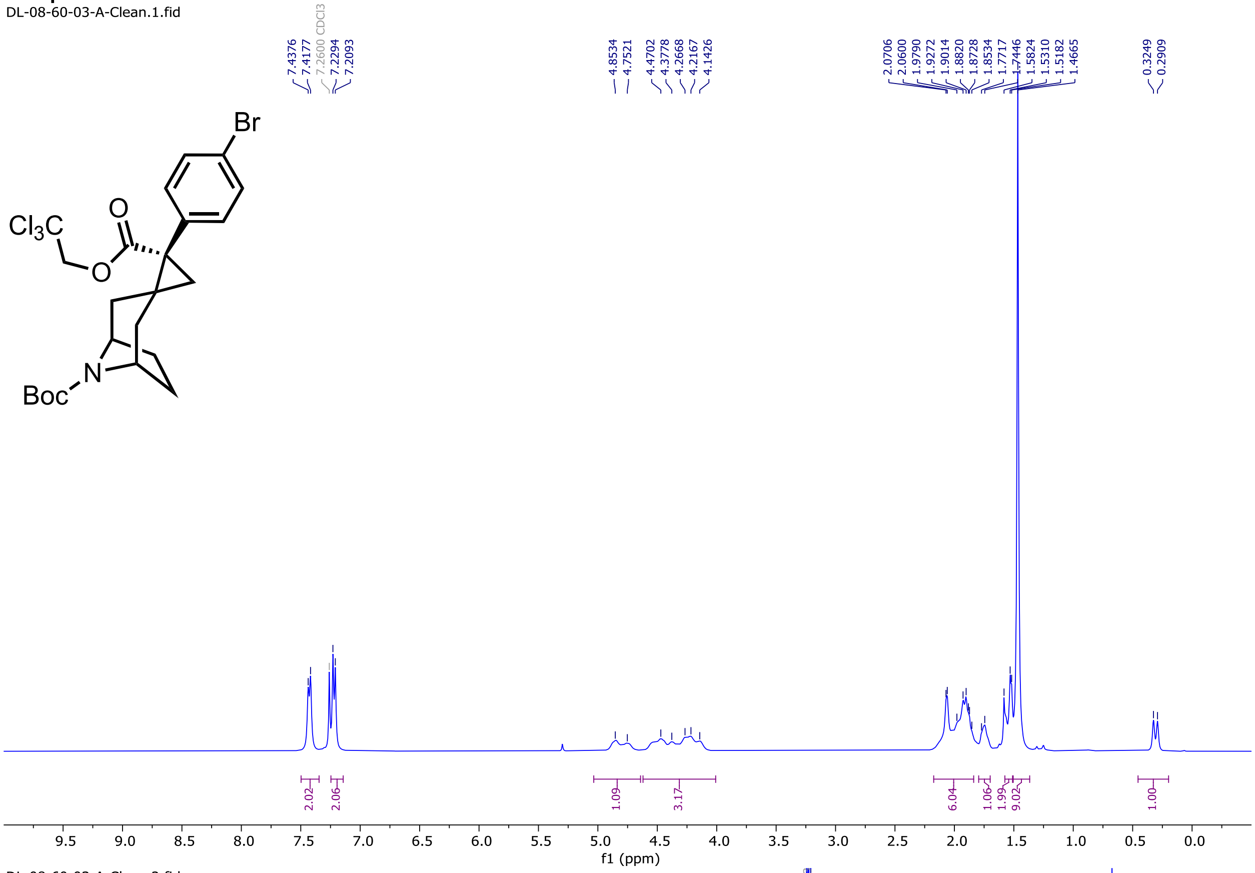
DL-08-60-03-A-Clean.1.fid
Br
Cl3C
O
O
N
Boc
7.4376
7.4177
7.2600 CDCl3
7.2294
7.2093
4.8534
4.7521
4.4702
4.3778
4.2668
4.2167
4.1426
2.0706
2.0600
1.9790
1.9272
1.9014
1.8820
1.8728
1.8534
1.7717
1.7446
1.5824
1.5310
1.5182
1.4665
0.3249
0.2909
2.02
2.06
1.09
3.17
6.04
1.06
1.99
9.02
1.00
f1 (ppm)

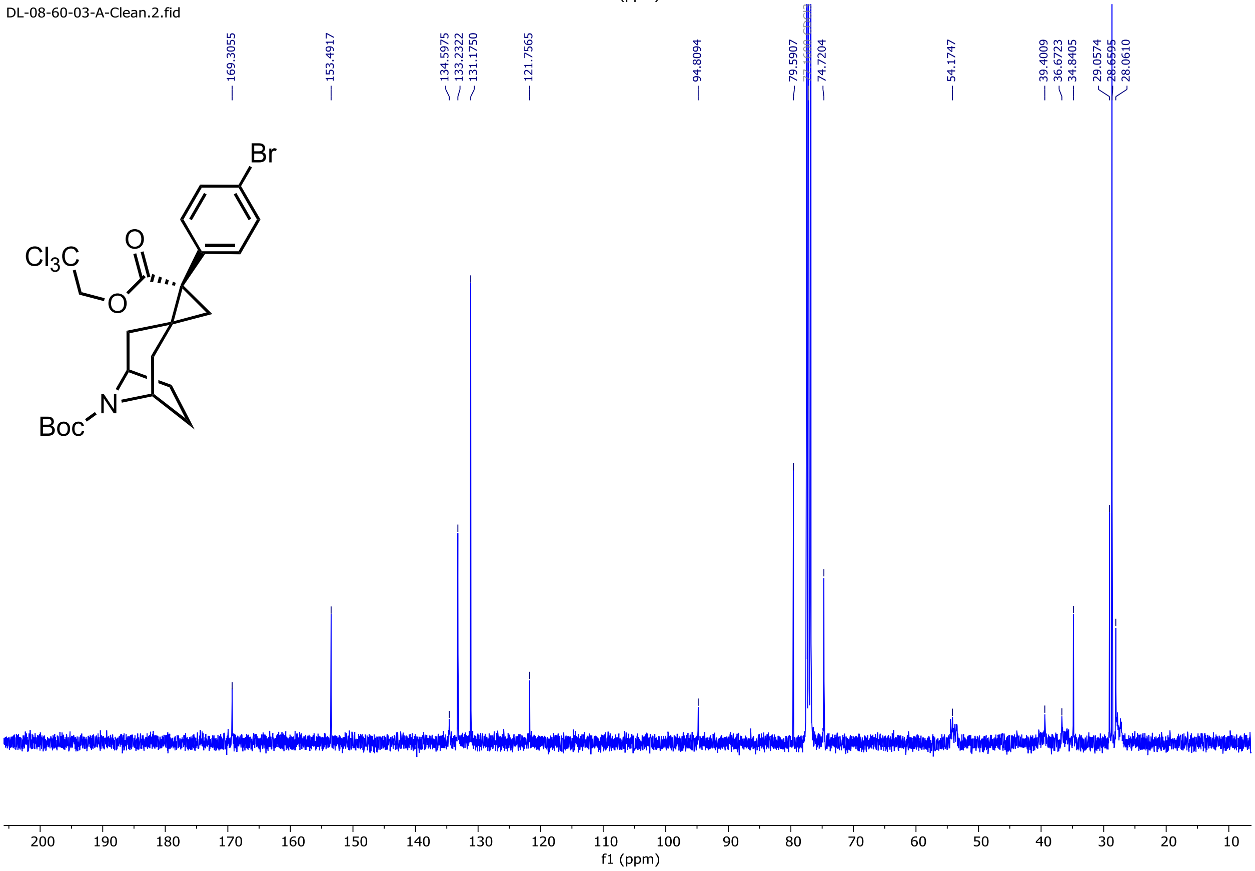
DL-08-60-03-A-Clean.2.fid
169.3055
153.4917
134.5975
133.2322
131.1750
121.7565
94.8094
79.5907
74.7204
54.1747
39.4009
36.6723
34.8405
29.0574
28.6595
28.0610
Br
Cl3C
O
O
N
Boc
f1 (ppm)

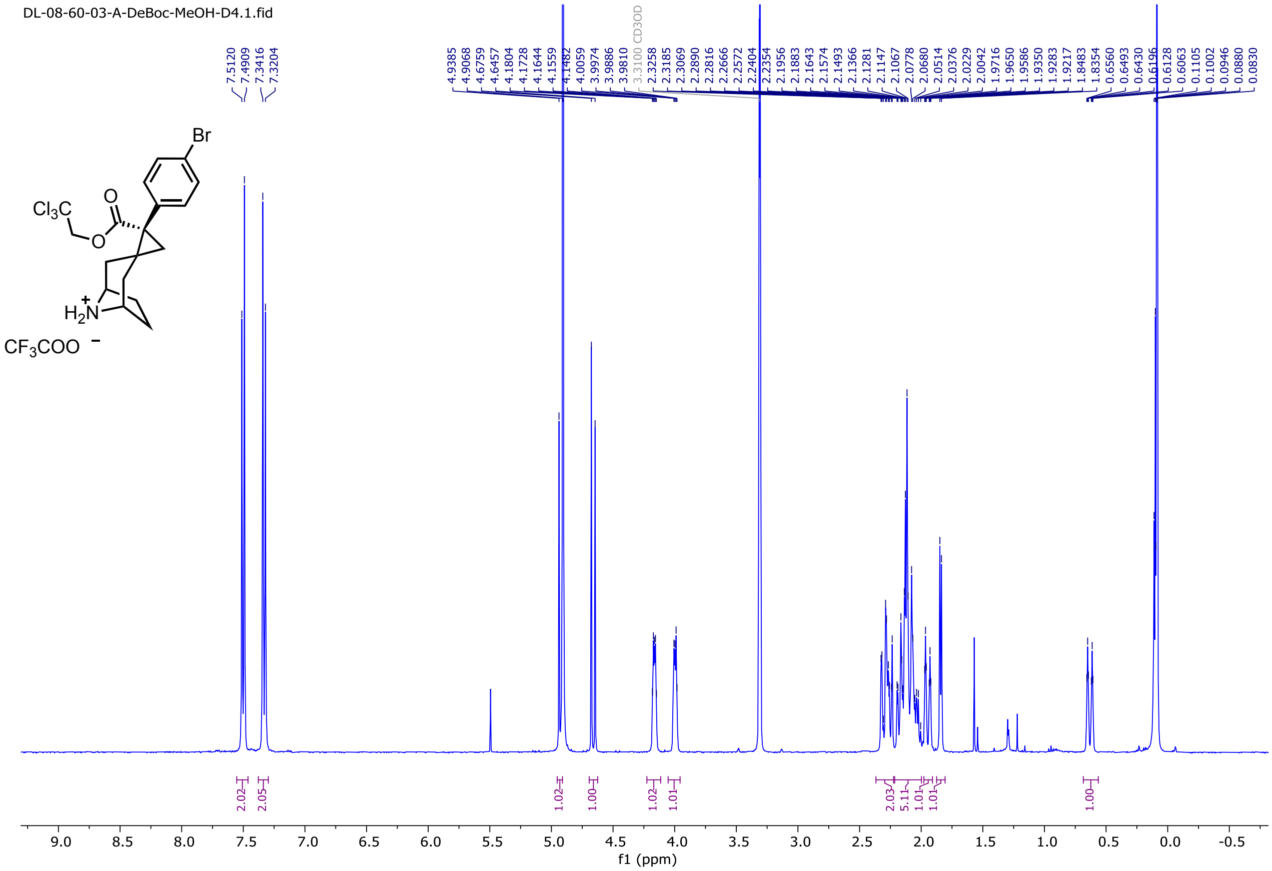
DL-08-60-03-A-DeBoc-MeOH-D4.1.fid
f1 (ppm)

Compound **44**

20240829-JKS-40-10-5-clean.1.fid

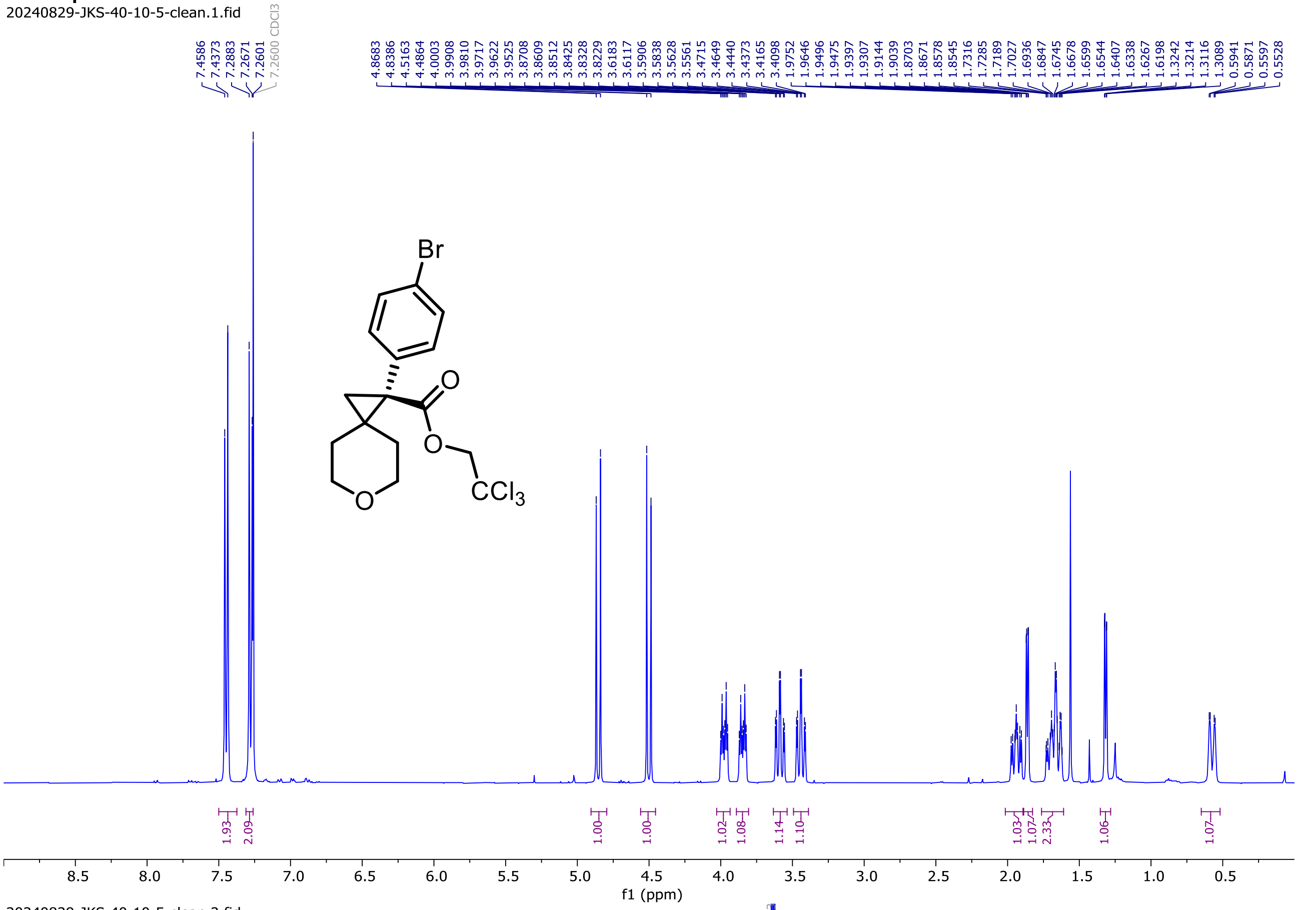

20240829-JKS-40-10-5-clean.2.fid

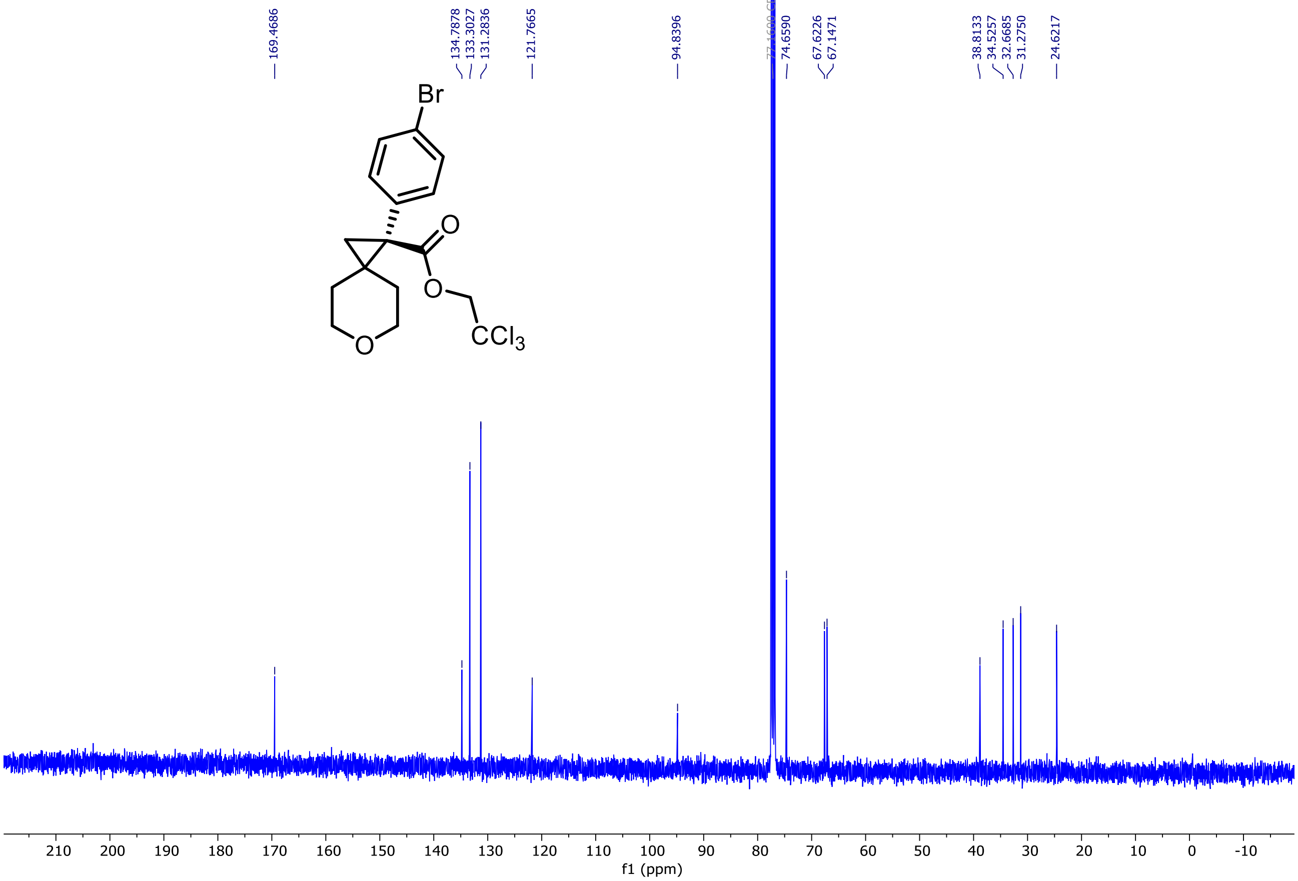

Compound **45**

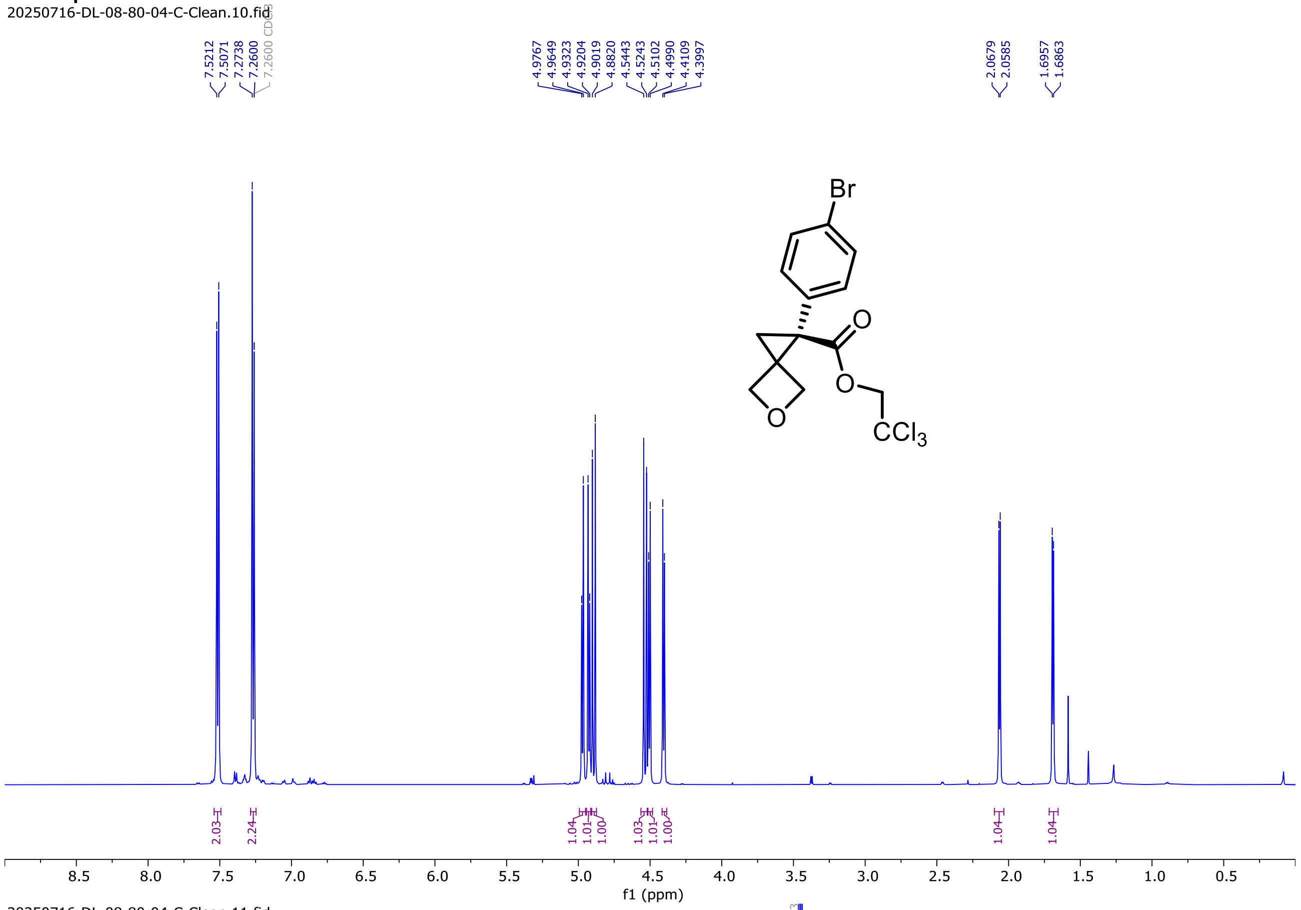

20250716-DL-08-80-04-C-Clean.10.fid
7.5212
7.5071
7.2738
7.2600
7.2600 CDCl3
4.9767
4.9649
4.9323
4.9204
4.9019
4.8820
4.5443
4.5243
4.5102
4.4990
4.4109
4.3997
2.0679
2.0585
1.6957
1.6863
Br
O
O
O
CCl_3
2.03
2.24
1.04
1.01
1.00
1.03
1.01
1.00
1.04
1.04
8.5
8.0
7.5
7.0
6.5
6.0
5.5
5.0
4.5
4.0
3.5
3.0
2.5
2.0
1.5
1.0
0.5
f1 (ppm)

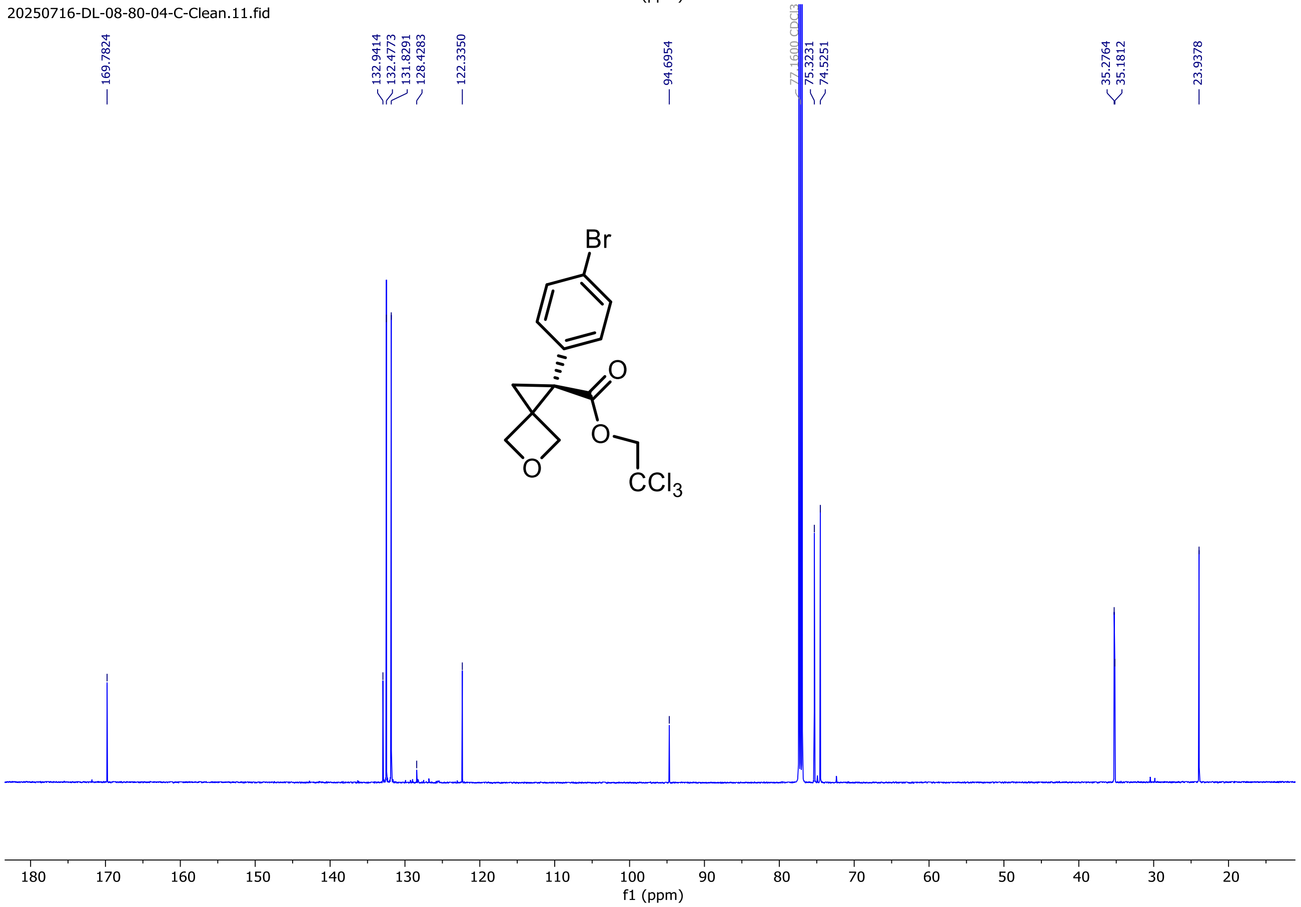

20250716-DL-08-80-04-C-Clean.11.fid
169.7824
132.9414
132.4773
131.8291
128.4283
122.3350
94.6954
77.1600 CDCl3
75.3231
74.5251
35.2764
35.1812
23.9378
Br
O
O
O
CCl_3
180
170
160
150
140
130
120
110
100
90
80
70
60
50
40
30
20
f1 (ppm)

9. X-Ray Crystallographic Data

Compound 30

Submitted by: **Joshua Sailor, Davies Lab**

Solved by: **John Bacsa**

***R_1*=1.21%**

Crystal Data and Experimental

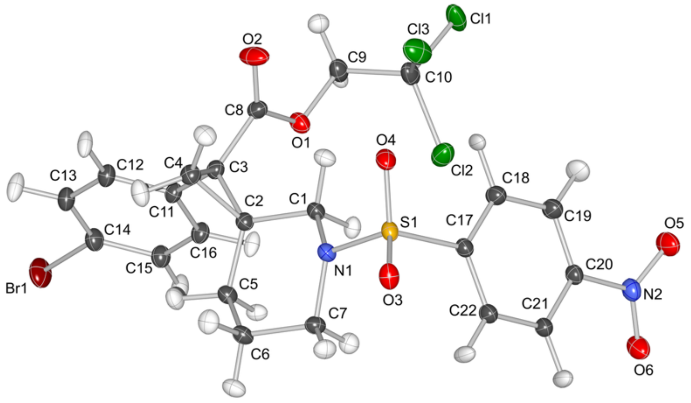

Experimental. Single colorless prism-shaped crystals of were chosen from the sample as supplied. A suitable crystal with dimensions 0.24 × 0.14 × 0.06 mm³ was selected and mounted on a loop with paratone on a XtaLAB Synergy, Dualflex, HyPix diffractometer. The crystal was kept at a steady T = 100.00(10) K during data collection. The structure was solved with the ShelXT (Sheldrick, 2015) solution program and by using Olex2 1.5-alpha (Dolomanov et al., 2009) as the graphical interface. The model was refined with olex2.refine 1.5-alpha (Bourhis et al., 2015) using full matrix least squares minimisation on F^2.

Crystal Data. $C_{22}H_{20}BrCl_3N_2O_6S$, M_r = 626.741, orthorhombic, $P2_12_12_1$ (No. 19), a = 6.45927(4) Å, b = 11.82710(6) Å, c = 32.87911(15) Å, $\alpha = \beta = \gamma$ = 90°, V = 2511.78(2) Å³, T = 100.00(10) K, Z = 4, Z' = 1, μ(Cu K_α) = 6.317, 56078 reflections measured, 5157 unique (R_{int} = 0.0295) which were used in all calculations. The final wR_2 was 0.0282 (all data) and R_1 was 0.0121 (I≥2 σ(I)).

**Compound**	JKS-40-25-3
Formula	$C_{22}H_{20}BrCl_3N_2O_6S$
$D_{calc.}$/ g cm^{-3}	1.657
μ/mm^{-1}	6.317
Formula Weight	626.741
Color	colorless
Shape	prism-shaped
Size/mm³	0.24×0.14×0.06
T/K	100.00(10)
Crystal System	orthorhombic
Flack Parameter	-0.011(2)
Hooft Parameter	-0.011(2)
Space Group	$P2_12_12_1$
a/Å	6.45927(4)
b/Å	11.82710(6)
c/Å	32.87911(15)
α/°	90
β/°	90
γ/°	90
V/Å³	2511.78(2)
Z	4
Z'	1
Wavelength/Å	1.54184
Radiation type	Cu K_α
Θ_{min}/°	2.69
Θ_{max}/°	76.68
Measured Refl's.	56078
Indep't Refl's	5157
Refl's I≥2 σ(I)	5131
R_{int}	0.0295
Parameters	562
Restraints	516
Largest Peak	0.1852
Deepest Hole	-0.1705
GooF	1.0990
wR_2 (all data)	0.0282
wR_2	0.0282
R_1 (all data)	0.0123
R_1	0.0121

Structure Quality Indicators

**Reflections:**	d min (CuKα) 2Θ=153.4° 0.79	I/σ(I) 85.5	Rint m=10.83 2.95%	Full 135.4° 99.5	
**Refinement:**	Shift 0.001	Max Peak 0.2	Min Peak -0.2	GooF 1.099	Hooft -.011(2)

A colorless prism-shaped-shaped crystal with dimensions 0.24 × 0.14 × 0.06 mm^3 was mounted on a loop with paratone. Data were collected using a XtaLAB Synergy, Dualflex, HyPix diffractometer operating at T = 100.00(10) K.

Data were measured using ω scans with Cu K_α radiation. The diffraction pattern was indexed and the total number of runs and images was based on the strategy calculation from the program CrysAlisPro system (CCD 44.57a 64-bit (release 20-06-2024)). The maximum resolution that was achieved was Θ = 76.68° (0.83 Å).

The unit cell was refined using CrysAlisPro 1.171.43.121a (Rigaku OD, 2024) on 46602 reflections, 83% of the observed reflections.

Data reduction, scaling and absorption corrections were performed using CrysAlisPro 1.171.43.121a (Rigaku OD, 2024). The final completeness is 99.47 % out to 76.68° in Θ. A numerical absorption correction based on gaussian integration over a multifaceted crystal model was performed using CrysAlisPro 1.171.42.74a (Rigaku Oxford Diffraction, 2022). An empirical absorption correction using spherical harmonics, implemented in SCALE3 ABSPACK scaling algorithm was also applied. The absorption coefficient μ of this material is 6.317 mm^{-1} at this wavelength (λ = 1.54184Å) and the minimum and maximum transmissions are 0.363 and 1.000.

The structure was solved and the space group $P2_12_12_1$ (# 19) determined by the ShelXT (Sheldrick, 2015) structure solution program and refined by full matrix least squares minimisation on $\boldsymbol{F}^2$ using version of olex2.refine 1.5-alpha (Bourhis et al., 2015). All atoms, even hydrogen atoms, were refined anisotropically. Hydrogen atom positions were located from the electron densities and freely refined using Hirshfeld scattering factors. Refinement was by using NoSpherA2, an implementation of non-spherical atom-form-factors (F. Kleemiss, H. Puschmann, O. Dolomanov, S.Grabowsky - https://doi.org/10.1039/D0SC05526C – 2020). NoSpherA2 implementation of HAR makes use of tailor-made aspherical atomic form factors calculated from a Hirshfeld-partitioned electron density (ED) not from spherical-atom form factors. The ED was calculated from a Gaussian basis set single determinant SCF wavefunction from DFT using selected functionals for a fragment of this crystal. This fragment was embedded in an electrostatic crystal field by employing cluster charges. SOFTWARE: ORCA 5.0 PARTITIONING: NoSpherA2 INT ACCURACY: Normal METHOD: PBE BASIS SET: def2-SVP CHARGE: 0 MULTIPLICITY: 1 SOLVATION: Chloroform DATE: 2024-12-02_17-17-27

There is a single formula unit in the asymmetric unit, which is represented by the reported sum formula. In other words: Z is 4 and Z' is 1. The moiety formula is C22 H20 Br Cl3 N2 O6 S.

The Flack parameter was refined to -0.011(2). Determination of absolute structure using Bayesian statistics on Bijvoet differences using the Olex2 results in -0.011(2). The chiral atoms in this structure are: C2(S), C3(R). Note: The Flack parameter is used to determine chirality of the crystal studied, the value should be near 0, a value of 1 means that the stereochemistry is wrong and the model should be inverted. A value of 0.5 means that the crystal consists of a racemic mixture of the two enantiomers.

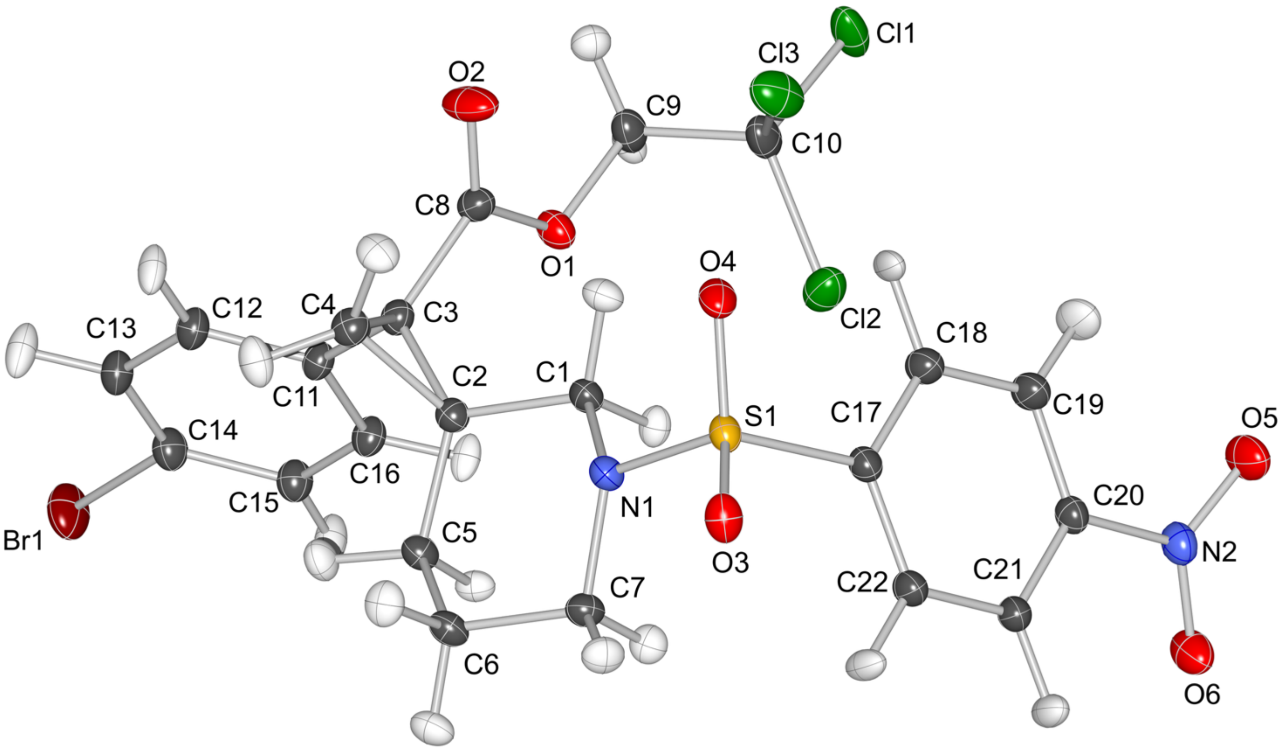

Figure 1 The molecular structure depicted using thermal ellipsoids (50% probability level for non-hydrogen atoms, 34% for hydrogen atoms). There are chiral atoms in this structure: C2(S), and C3(R).

Data Plots: Diffraction Data

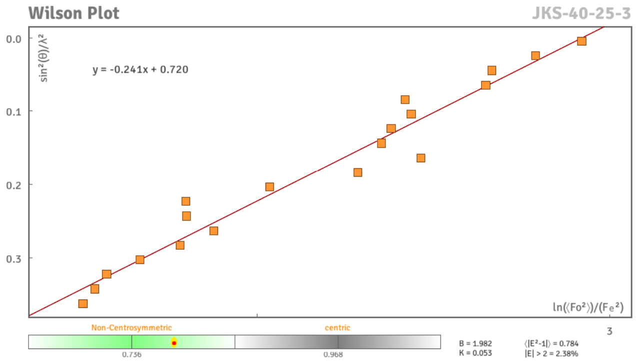

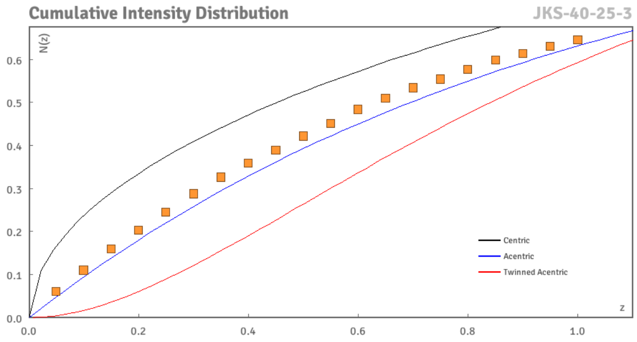

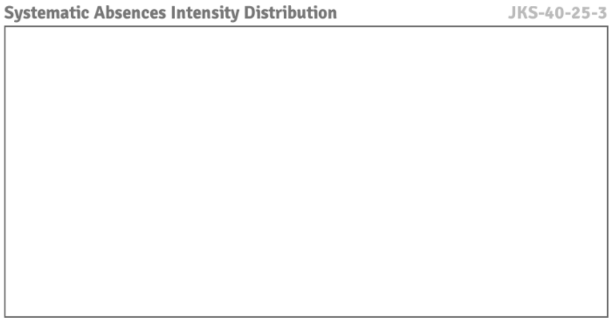

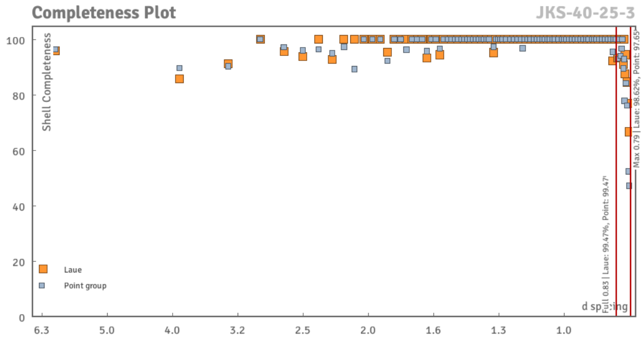

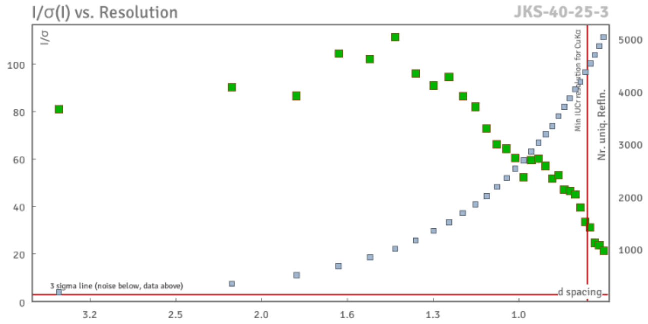

Data Plots: Refinement and Data

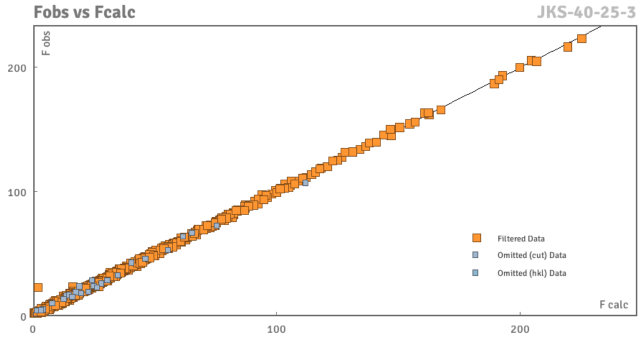

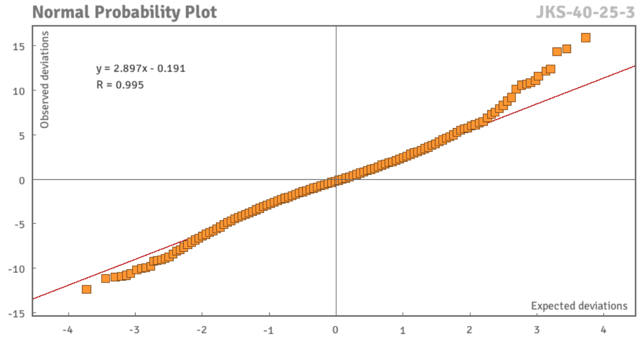

Reflection Statistics

Total reflections (after filtering)	55869	Unique reflections	5157
Completeness	0.977	Mean I/σ	65.57
hkl_{max} collected	(8, 14, 41)	hkl_{min} collected	(-8, -14, -36)
hkl_{max} used	(8, 14, 41)	hkl_{min} used	(-8, 0, 0)
Lim d_{max} collected	100.0	Lim d_{min} collected	0.77
d_{max} used	16.44	d_{min} used	0.79
Friedel pairs	2958	Friedel pairs merged	0
Inconsistent equivalents	10	R_{int}	0.0295
R_{sigma}	0.0116	Intensity transformed	0
Omitted reflections	0	Omitted by user (OMIT hkl)	209
Multiplicity	(2927, 1945, 1775, 1376, 1184, 959, 765, 543, 384, 283, 226, 169, 149, 96, 71, 58, 26, 15, 8, 5, 1)	Maximum multiplicity	32
Removed systematic absences	0	Filtered off (Shel/OMIT)	0

Table 1: Fractional Atomic Coordinates (×10^4) and Equivalent Isotropic Displacement Parameters (Å^2×10^3) for **COMPOUND 30.** U_{eq} is defined as 1/3 of the trace of the orthogonalised U_{ij}.

Atom	x	y	z	U_{eq}
Br1	5138.5(5)	-1425.8(2)	2623.61(9)	30.68(11)
Cl1	-406.0(12)	6619.3(5)	2663.0(2)	35.11(7)
Cl2	3012.2(11)	5956.5(6)	3177.4(2)	30.78(7)
Cl3	-1131.6(12)	6029.9(6)	3502.2(2)	34.09(7)
S1	1986.0(4)	5173.13(18)	4821.04(7)	14.78(5)

Atom	x	y	z	U_{eq}
O1	970.3(11)	3713.2(6)	3184.7(2)	19.53(14)
O2	-2064.8(12)	3317.5(7)	3487.2(2)	30.62(18)
O3	2617.9(12)	5084.3(6)	5236.94(19)	19.90(15)
O4	-144.2(12)	5369.1(5)	4722.3(2)	20.24(14)
O5	6251.9(13)	9462.5(6)	3818.8(2)	27.38(17)
O6	8989.1(11)	8606.8(7)	4036.8(2)	25.72(16)
N1	2673.5(12)	4001.6(7)	4596.6(2)	14.50(16)
N2	7112.8(14)	8715.7(7)	4010.4(2)	19.32(17)
C1	2103.4(15)	3877.3(8)	4163.4(3)	15.17(18)
C2	2158.4(15)	2634.5(8)	4052.8(3)	14.10(18)
C3	993.4(15)	2258.7(8)	3671.8(3)	14.92(18)
C4	202.6(16)	1945.0(8)	4089.1(3)	16.79(18)
C5	4213.0(15)	2077.7(8)	4151.2(3)	15.67(19)
C6	4758.6(16)	2290.3(8)	4597.1(3)	16.56(18)
C7	4745.1(15)	3544.4(8)	4695.0(3)	16.41(18)
C8	-244.1(15)	3136.6(8)	3448.4(3)	16.82(18)
C9	16.3(19)	4510.6(8)	2920.0(3)	19.96(19)
C10	360(3)	5722.2(17)	3065.9(6)	23.30(16)
C11	1966.3(15)	1385.7(8)	3401.1(2)	14.90(17)
C12	1027.4(16)	334.4(8)	3354.2(3)	17.84(19)
C13	1953.5(17)	-510.1(8)	3119.7(3)	18.77(19)
C14	3835.2(17)	-273.1(8)	2931.9(3)	19.3(2)
C15	4789.5(16)	775.6(8)	2965.6(3)	18.57(19)
C16	3837.5(15)	1606.0(8)	3201.7(3)	16.64(19)
C17	3460.4(14)	6269.1(8)	4595.5(3)	15.21(18)
C18	2588.4(16)	6922.1(8)	4287.0(3)	18.8(2)
C19	3774.7(16)	7753.6(9)	4098.5(3)	19.6(2)
C20	5815.3(15)	7877.8(8)	4219.0(3)	16.19(19)
C21	6709.5(15)	7233.4(8)	4524.6(3)	16.72(19)
C22	5502.2(14)	6424.6(8)	4720.4(3)	17.04(18)

Table 2: Anisotropic Displacement Parameters (×10^4) for **COMPOUND 30.** The anisotropic displacement factor exponent takes the form: $-2\pi^2[h^2a^{*2} \times U_{11} + ... +2hka^* \times b^* \times U_{12}]$

Atom	U_{11}	U_{22}	U_{33}	U_{23}	U_{13}	U_{12}
Br1	32.6(2)	19.87(17)	39.6(2)	3.41(16)	11.40(17)	-7.85(14)
Cl1	53.52(19)	18.10(11)	33.70(13)	2.30(11)	-19.28(12)	3.65(9)
Cl2	32.07(14)	29.53(13)	30.74(13)	-7.99(11)	-9.12(10)	5.72(10)
Cl3	37.47(15)	34.68(15)	30.12(13)	11.02(12)	-4.39(11)	-10.33(11)
S1	16.01(11)	11.67(10)	16.65(10)	0.52(9)	2.79(8)	0.29(8)
O1	19.2(3)	17.8(3)	21.5(3)	2.3(3)	2.3(3)	5.2(3)
O2	13.8(4)	39.9(5)	38.2(4)	0.6(3)	0.3(3)	16.8(4)
O3	27.7(4)	16.6(3)	15.3(3)	-1.2(3)	4.0(3)	-0.5(3)
O4	15.2(3)	16.1(3)	29.4(3)	1.8(3)	4.1(3)	0.3(3)
O5	25.9(4)	24.5(4)	31.7(4)	-1.4(3)	-0.3(3)	13.6(3)
O6	19.8(3)	22.9(4)	34.5(4)	0.6(3)	6.6(3)	9.7(3)
N1	14.7(4)	13.1(4)	15.7(4)	1.5(3)	-0.3(3)	0.4(3)
N2	21.3(4)	15.7(4)	21.0(4)	-0.5(3)	2.7(3)	3.8(3)
C1	16.1(4)	13.6(4)	15.8(4)	1.2(4)	-1.5(4)	1.5(3)
H1a	28(5)	20(5)	19(5)	-5(2)	3(2)	1(2)
H1b	22(3)	28(6)	26(6)	4.9(18)	-0.8(18)	4(3)
C2	14.1(4)	14.1(4)	14.1(4)	0.2(3)	1.1(3)	0.9(3)
C3	14.9(4)	14.5(4)	15.4(4)	-2.0(3)	0.5(3)	1.4(3)
C4	16.2(5)	18.3(4)	15.9(4)	-1.4(4)	2.5(4)	1.4(3)

Atom	U_{11}	U_{22}	U_{33}	U_{23}	U_{13}	U_{12}
H4a	45(7)	20(3)	25(6)	0.1(18)	7(4)	3.2(17)
H4b	24(4)	30(5)	28(6)	4(2)	7(2)	2(3)
C5	16.6(5)	15.7(5)	14.8(4)	2.5(4)	0.2(3)	0.2(3)
H5a	34(6)	19(3)	28(6)	3.2(15)	-2(3)	-2.3(15)
H5b	18(4)	28(5)	16(4)	1(2)	0(2)	3(2)
C6	18.0(5)	17.2(4)	14.4(4)	4.1(4)	-0.1(4)	2.2(3)
H6a	33(5)	29(6)	23(5)	-4(2)	6(2)	2(2)
H6b	25(3)	31(6)	26(6)	6.5(18)	-0.9(18)	7(3)
C7	15.4(4)	17.9(4)	16.0(4)	1.8(4)	-1.9(3)	-0.2(3)
H7a	26(6)	31(6)	20(3)	1(3)	-3.3(15)	-2.1(15)
H7b	26(4)	26(5)	22(5)	-1(2)	2(2)	1(2)
C8	14.6(5)	18.7(4)	17.1(4)	-1.7(4)	-1.4(3)	2.5(3)
C9	26.1(5)	14.8(3)	19.0(4)	1.0(2)	-2.8(4)	-1.0(2)
H9a	32(5)	19(5)	19(3)	5(3)	-1.2(16)	0.7(17)
H9b	26(2)	29(6)	26(6)	-0.9(11)	-4.5(12)	0(3)
C10	30.0(3)	14.3(3)	25.6(3)	2.30(15)	-7.95(13)	-3.23(14)
C11	16.5(4)	14.5(4)	13.7(4)	-3.7(4)	1.1(3)	0.4(3)
C12	20.3(5)	15.3(5)	17.9(4)	-3.9(4)	1.9(4)	0.2(3)
H12	30(4)	17(6)	44(7)	-7(2)	16(2)	-4(4)
C13	22.8(5)	14.0(5)	19.5(4)	-3.2(4)	2.6(4)	-0.7(3)
H13	37(6)	23(3)	44(8)	-12(2)	17(4)	-11(3)
C14	22.3(5)	15.4(4)	20.3(4)	0.2(4)	2.4(4)	-1.4(4)
C15	20.6(5)	16.6(4)	18.5(4)	-0.8(4)	2.9(4)	-1.0(3)
H15	32(4)	21(6)	45(7)	-5(3)	17(3)	-8(4)
C16	17.1(4)	16.0(5)	16.8(4)	-3.1(4)	3.3(3)	0.2(4)
H16	29(6)	20(3)	23(6)	-9(2)	13(4)	-5(2)
C17	15.8(4)	13.2(4)	16.6(4)	0.8(3)	0.3(3)	1.7(3)
C18	17.3(5)	18.9(5)	20.2(5)	-0.9(4)	-2.0(4)	3.9(4)
H18	23(3)	20(7)	45(8)	-5(2)	-12(2)	18(5)
C19	19.6(5)	19.2(5)	20.0(5)	-0.7(4)	-2.7(4)	5.0(4)
H19	40(7)	37(7)	38(6)	-5(4)	-13(3)	21(3)
C20	18.0(5)	13.7(4)	16.8(4)	-0.3(3)	0.6(3)	2.8(3)
C21	16.1(5)	14.1(4)	19.9(4)	-0.5(4)	-1.6(4)	2.6(3)
H21	23(3)	28(7)	46(8)	-8(2)	-11(2)	18(5)
C22	16.9(5)	15.1(4)	19.2(4)	-0.1(4)	-1.4(3)	3.7(4)
H22	21(6)	33(7)	39(6)	-9(3)	-10(3)	22(3)

Table 3: Bond Lengths in Å for **COMPOUND 30**.

Atom	Atom	Length/Å
Br1	C14	1.8959(10)
Cl1	C10	1.768(2)
Cl2	C10	1.774(2)
Cl3	C10	1.766(2)
S1	O3	1.4309(7)
S1	O4	1.4326(8)
S1	N1	1.6314(8)
S1	C17	1.7712(9)
O1	C8	1.3535(11)
O1	C9	1.4237(11)
O2	C8	1.2021(13)
O5	N2	1.2192(11)
O6	N2	1.2218(11)
N1	C1	1.4786(11)

Atom	Atom	Length/Å
N1	C7	1.4790(11)
N2	C20	1.4680(12)
C1	H1a	1.119(12)
C1	H1b	1.112(12)
C1	C2	1.5147(13)
C2	C3	1.5273(12)
C2	C4	1.5083(13)
C2	C5	1.5165(13)
C3	C4	1.5102(12)
C3	C8	1.5021(13)
C3	C11	1.5010(13)
C4	H4a	1.074(12)
C4	H4b	1.070(13)
C5	H5a	1.086(13)
C5	H5b	1.075(12)

Atom	Atom	Length/Å
C5	C6	1.5286(12)
C6	H6a	1.081(13)
C6	H6b	1.084(13)
C6	C7	1.5178(13)
C7	H7a	1.036(10)
C7	H7b	1.063(12)
C9	H9a	1.103(12)
C9	H9b	1.059(13)
C9	C10	1.527(2)
C11	C12	1.3920(13)
C11	C16	1.3996(13)
C12	H12	1.069(12)
C12	C13	1.3963(14)
C13	H13	1.054(13)
C13	C14	1.3918(15)

Atom	Atom	Length/Å
C14	C15	1.3895(14)
C15	H15	1.067(13)
C15	C16	1.3946(14)
C16	H16	1.068(12)
C17	C18	1.3938(13)
C17	C22	1.3935(13)
C18	H18	1.125(13)
C18	C19	1.3923(14)
C19	H19	1.090(13)
C19	C20	1.3842(14)
C20	C21	1.3870(13)
C21	H21	1.099(13)
C21	C22	1.3919(13)
C22	H22	1.081(12)

Table 4: Bond Angles in ° for **COMPOUND 30**.

Atom	Atom	Atom	Angle/°
O4	S1	O3	120.16(4)
N1	S1	O3	106.99(4)
N1	S1	O4	107.24(4)
C17	S1	O3	107.49(4)
C17	S1	O4	107.64(4)
C17	S1	N1	106.61(4)
C9	O1	C8	118.33(8)
C1	N1	S1	116.89(6)
C7	N1	S1	117.25(6)
C7	N1	C1	113.55(7)
O6	N2	O5	124.44(9)
C20	N2	O5	118.05(8)
C20	N2	O6	117.51(8)
H1a	C1	N1	108.8(6)
H1b	C1	N1	108.1(6)
H1b	C1	H1a	110.0(9)
C2	C1	N1	108.79(7)
C2	C1	H1a	110.3(6)
C2	C1	H1b	110.8(7)
C3	C2	C1	117.89(8)
C4	C2	C1	119.08(8)
C4	C2	C3	59.67(6)
C5	C2	C1	113.00(8)
C5	C2	C3	118.67(8)
C5	C2	C4	118.77(8)
C4	C3	C2	59.54(6)
C8	C3	C2	117.51(8)
C8	C3	C4	115.72(8)
C11	C3	C2	118.69(8)
C11	C3	C4	120.75(8)
C11	C3	C8	114.11(7)
C3	C4	C2	60.79(6)
H4a	C4	C2	120.1(8)
H4a	C4	C3	118.1(7)
H4b	C4	C2	116.8(7)
H4b	C4	C3	116.7(7)

Atom	Atom	Atom	Angle/°
H4b	C4	H4a	114.2(10)
H5a	C5	C2	109.1(7)
H5b	C5	C2	108.4(6)
H5b	C5	H5a	108.7(9)
C6	C5	C2	109.57(8)
C6	C5	H5a	110.4(7)
C6	C5	H5b	110.6(6)
H6a	C6	C5	111.0(7)
H6b	C6	C5	110.1(7)
H6b	C6	H6a	105.1(10)
C7	C6	C5	111.28(8)
C7	C6	H6a	110.0(7)
C7	C6	H6b	109.1(7)
C6	C7	N1	108.43(8)
H7a	C7	N1	106.5(7)
H7a	C7	C6	111.4(7)
H7b	C7	N1	110.0(7)
H7b	C7	C6	110.7(7)
H7b	C7	H7a	109.7(9)
O2	C8	O1	123.04(9)
C3	C8	O1	110.67(8)
C3	C8	O2	126.28(9)
H9a	C9	O1	105.5(6)
H9b	C9	O1	110.5(7)
H9b	C9	H9a	112.2(10)
C10	C9	O1	111.50(10)
C10	C9	H9a	108.3(6)
C10	C9	H9b	108.9(7)
Cl2	C10	Cl1	109.34(12)
Cl3	C10	Cl1	109.42(12)
Cl3	C10	Cl2	109.07(11)
C9	C10	Cl1	106.69(12)
C9	C10	Cl2	110.59(13)
C9	C10	Cl3	111.68(13)
C12	C11	C3	119.86(8)
C16	C11	C3	120.75(8)
C16	C11	C12	119.38(9)

Atom	Atom	Atom	Angle/°
H12	C12	C11	120.4(7)
C13	C12	C11	120.90(9)
C13	C12	H12	118.7(7)
H13	C13	C12	120.2(7)
C14	C13	C12	118.36(9)
C14	C13	H13	121.4(7)
C13	C14	Br1	118.70(8)
C15	C14	Br1	119.17(8)
C15	C14	C13	122.13(9)
H15	C15	C14	119.6(7)
C16	C15	C14	118.51(9)
C16	C15	H15	121.9(7)
C15	C16	C11	120.70(9)
H16	C16	C11	118.2(6)
H16	C16	C15	121.1(6)
C18	C17	S1	119.53(7)
C22	C17	S1	118.82(7)

Atom	Atom	Atom	Angle/°
C22	C17	C18	121.59(9)
H18	C18	C17	121.9(6)
C19	C18	C17	119.53(9)
C19	C18	H18	118.6(6)
H19	C19	C18	122.8(8)
C20	C19	C18	118.14(9)
C20	C19	H19	119.0(8)
C19	C20	N2	118.78(9)
C21	C20	N2	118.15(9)
C21	C20	C19	123.07(9)
H21	C21	C20	120.5(7)
C22	C21	C20	118.64(9)
C22	C21	H21	120.9(7)
C21	C22	C17	118.99(9)
H22	C22	C17	121.7(7)
H22	C22	C21	119.3(7)

Table 5: Torsion Angles in ° for **COMPOUND 30**.

Atom	Atom	Atom	Atom	Angle/°
Br1	C14	C13	C12	178.51(7)
Br1	C14	C15	C16	-178.43(7)
Cl1	C10	C9	O1	167.95(9)
Cl2	C10	C9	O1	49.14(13)
Cl3	C10	C9	O1	-72.53(13)
S1	N1	C1	C2	-160.03(7)
S1	N1	C7	C6	158.14(7)
S1	C17	C18	C19	-177.44(7)
S1	C17	C22	C21	175.66(7)
O1	C8	C3	C2	85.17(8)
O1	C8	C3	C4	152.62(8)
O1	C8	C3	C11	-60.54(8)
O2	C8	C3	C2	-95.61(11)
O2	C8	C3	C4	-28.16(12)
O2	C8	C3	C11	118.68(11)
O5	N2	C20	C19	18.92(10)
O5	N2	C20	C21	-162.11(9)
O6	N2	C20	C19	-160.17(9)
O6	N2	C20	C21	18.80(10)
N1	C1	C2	C3	160.98(7)
N1	C1	C2	C4	92.05(8)
N1	C1	C2	C5	-54.49(8)
N1	C7	C6	C5	57.67(8)
N2	C20	C19	C18	177.26(9)
N2	C20	C21	C22	-179.05(8)
C1	C2	C3	C4	-109.11(12)
C1	C2	C3	C8	-3.96(10)
C1	C2	C3	C11	140.15(8)
C1	C2	C4	C3	107.15(11)
C1	C2	C5	C6	53.53(8)
C2	C3	C11	C12	115.42(9)
C2	C3	C11	C16	-63.12(9)
C2	C4	C3	C8	-108.16(7)
C2	C4	C3	C11	107.35(7)

Atom	Atom	Atom	Atom	Angle/˚
C2	C5	C6	C7	-54.83(8)
C3	C11	C12	C13	-176.90(8)
C3	C11	C16	C15	176.96(8)
C11	C12	C13	C14	-0.36(11)
C11	C16	C15	C14	0.21(11)
C12	C13	C14	C15	-1.07(11)
C13	C14	C15	C16	1.14(11)
C17	C18	C19	C20	1.79(11)
C17	C22	C21	C20	1.75(11)
C18	C19	C20	C21	-1.66(12)
C19	C20	C21	C22	-0.13(11)

Table 6: Hydrogen Fractional Atomic Coordinates (×10^4) and Equivalent Isotropic Displacement Parameters (Å^2×10^3) for **COMPOUND 30.** U_{eq} is defined as 1/3 of the trace of the orthogonalised U_{ij}.

Atom	x	y	z	U_{eq}
H1a	3240(20)	4361(10)	3974(3)	22(3)
H1b	520(19)	4229(11)	4121(4)	25(3)
H4a	280(20)	1073(10)	4179(4)	30(3)
H4b	-1150(20)	2379(11)	4193(4)	27(3)
H5a	4100(20)	1176(11)	4092(4)	27(3)
H5b	5377(19)	2429(10)	3955(3)	21(2)
H6a	3710(20)	1847(11)	4797(4)	28(3)
H6b	6280(20)	1947(11)	4665(4)	27(3)
H7a	4975(19)	3686(10)	5003(3)	26(2)
H7b	5897(19)	3977(11)	4524(4)	25(3)
H9a	800(20)	4408(10)	2624(4)	24(3)
H9b	-1600(20)	4357(11)	2900(4)	27(3)
H12	-410(20)	156(10)	3500(4)	30(3)
H13	1260(20)	-1313(11)	3095(4)	35(3)
H15	6240(20)	919(11)	2818(4)	33(3)
H16	4507(19)	2427(10)	3234(4)	24(3)
H18	940(20)	6804(11)	4186(4)	29(3)
H19	3190(20)	8271(12)	3849(4)	38(4)
H21	8340(20)	7361(12)	4610(4)	32(3)
H22	6170(20)	5934(11)	4965(4)	31(3)

Citations

CrysAlisPro (ROD), Rigaku Oxford Diffraction, Poland (?).

CrysAlisPro Software System, Rigaku Oxford Diffraction, (2024).

L.J. Bourhis and O.V. Dolomanov and R.J. Gildea and J.A.K. Howard and H. Puschmann, The Anatomy of a Comprehensive Constrained, Restrained, Refinement Program for the Modern Computing Environment - Olex2 Disected, *Acta Cryst. A*, (2015), **A71**, 59-71.

O.V. Dolomanov and L.J. Bourhis and R.J. Gildea and J.A.K. Howard and H. Puschmann, Olex2: A complete structure solution, refinement and analysis program, *J. Appl. Cryst.*, (2009), **42**, 339-341.

Sheldrick, G.M., ShelXT-Integrated space-group and crystal-structure determination, *Acta Cryst.*, (2015), **A71**, 3-8.

10. DFT calculations

All calculations were performed using Gaussian 16 suite of programs. [1] Images of 3D structures were rendered using VMD[2], CYLView[3]. Geometry optimizations were carried out using the density functional B3LYP-D3(BJ)[4-9] in conjunction with Lanl2dz[10, 11] basic set for rhodium and 6-31G(d,p)[12, 13] basic set for other atoms. Solvent effects were incorporated into all calculated properties at the Conductor-like Polarizable Continuum Solvation Model (CPCM) level of theory using dichloromethane (CH_2Cl_2) as solvent.[14] Ground and transition state geometries were validated by vibrational analysis at the same level of theory. Gibbs free energy and Zero-point energy corrections were calculated at the standard reaction conditions (T = 298.15K, and pressure = 1 atm). To confirm that all located saddle points correspond to relevant transformations, Intrinsic Reaction Coordinate (IRC) calculations[15, 16] were performed (total of 50 points via each direction) followed by subsequent optimization of the end points with the previously mentioned optimization method. The choice of computational approach was based on prior studies showing that the [B3LYP-D3(BJ) + PCM(CH_2Cl_2)]/[6-31G(d,p) + Lanl2dz] approach describes appropriately the geometries of organic and organometallic species. [17-20] The distortion-interaction analysis was performed following the Houk-Bickelhaupt protocol[21]. IGMH analysis[22] and RDG analysis[23] were performed with Multiwfn 3.8(dev) [24] and visualized by VMD[2] using the default parameters and a grid resolution of 0.15 Bohr with the isovalue of 0.007 (for IGMH) and 0.4 (for RDG). The input for IGMH and RDG analysis is obtained from the computational output (.fchk) from Gaussian 16. sobEDAw[25] calculation was also performed with Multiwfn 3.8(dev) [24].

Reference

(1) Frisch, M. J.; Trucks, G. W.; Schlegel, H. B.; Scuseria, G. E.; Robb, M. A.; Cheeseman, J. R.; Scalmani, G.; Barone, V.; Petersson, G. A.; Nakatsuji, H.; et al. Gaussian 16 Rev. C.01. **2016**.
(2) Humphrey, W.; Dalke, A.; Schulten, K. VMD: Visual molecular dynamics. *J. Mol. Graph.* **1996**, *14*, 33-38.
(3) Legault, C. Y. CYLview20. **2020**.
(4) Lee, C.; Yang, W.; Parr, R. G. Development of the Colle-Salvetti correlation-energy formula into a functional of the electron density. *Phys. Rev. B* **1988**, *37*, 785-789.
(5) Becke, A. D. A new mixing of Hartree–Fock and local density-functional theories. *J. Chem. Phys.* **1993**, *98*, 1372-1377.
(6) Becke, A. D. Density-functional thermochemistry. III. The role of exact exchange. *J. Chem. Phys.* **1993**, *98*, 5648-5652.
(7) Grimme, S.; Hansen, A.; Brandenburg, J. G.; Bannwarth, C. Dispersion-Corrected Mean-Field Electronic Structure Methods. *Chem. Rev.* **2016**, *116*, 5105-5154.

(8) Grimme, S.; Antony, J.; Ehrlich, S.; Krieg, H. A consistent and accurate ab initio parametrization of density functional dispersion correction (DFT-D) for the 94 elements H-Pu. *J. Chem. Phys.* **2010**, *132*, 154104.
(9) Johnson, E. R.; Becke, A. D. A post-Hartree-Fock model of intermolecular interactions: Inclusion of higher-order corrections. *J. Chem. Phys.* **2006**, *124*, 174104.
(10) Hay, P. J.; Wadt, W. R. Ab initio effective core potentials for molecular calculations. Potentials for K to Au including the outermost core orbitals. *J. Chem. Phys.* **1985**, *82*, 299-310.
(11) Roy, L. E.; Hay, P. J.; Martin, R. L. Revised Basis Sets for the LANL Effective Core Potentials. *J. Chem. Theory Comput.* **2008**, *4*, 1029-1031.
(12) Hariharan, P. C.; Pople, J. A. The influence of polarization functions on molecular orbital hydrogenation energies. *Theoretica chimica acta* **1973**, *28*, 213-222.
(13) Hehre, W. J.; Ditchfield, R.; Pople, J. A. Self—Consistent Molecular Orbital Methods. XII. Further Extensions of Gaussian—Type Basis Sets for Use in Molecular Orbital Studies of Organic Molecules. *J. Chem. Phys.* **1972**, *56*, 2257-2261.
(14) Cossi, M.; Rega, N.; Scalmani, G.; Barone, V. Energies, structures, and electronic properties of molecules in solution with the C-PCM solvation model. *J. Comput. Chem.* **2003**, *24*, 669-681.
(15) Fukui, K. The path of chemical reactions - the IRC approach. *Acc. Chem. Res.* **1981**, *14*, 363-368.
(16) Fukui, K. Formulation of the reaction coordinate. *J. Phys. Chem.* **1970**, *74*, 4161-4163.
(17) Musaev, D. G.; Figg, T. M.; Kaledin, A. L. Versatile reactivity of Pd-catalysts: mechanistic features of the mono-N-protected amino acid ligand and cesium-halide base in Pd-catalyzed C–H bond functionalization. *Chem. Soc. Rev.* **2014**, *43*, 5009-5031.
(18) Ren, Z.; Musaev, D. G.; Davies, H. M. L. Key Selectivity Controlling Elements in Rhodium-Catalyzed C–H Functionalization with Donor/Acceptor Carbenes. *ACS Catal.* **2022**, *12*, 13446-13456.
(19) Hansen, J.; Autschbach, J.; Davies, H. M. L. Computational Study on the Selectivity of Donor/Acceptor-Substituted Rhodium Carbenoids. *J. Org. Chem.* **2009**, *74*, 6555-6563.
(20) Nakamura, E.; Yoshikai, N.; Yamanaka, M. Mechanism of C−H Bond Activation/C−C Bond Formation Reaction between Diazo Compound and Alkane Catalyzed by Dirhodium Tetracarboxylate. *J. Am. Chem. Soc.* **2002**, *124*, 7181-7192.
(21) Bickelhaupt, F. M.; Houk, K. N. Analyzing Reaction Rates with the Distortion/Interaction-Activation Strain Model. *Angew. Chem., Int. Ed.* **2017**, *56*, 10070-10086.
(22) Lu, T.; Chen, Q. Independent gradient model based on Hirshfeld partition: A new method for visual study of interactions in chemical systems. *J. Comput. Chem.* **2022**, *43*, 539-555.
(23) Johnson, E. R.; Keinan, S.; Mori-Sánchez, P.; Contreras-García, J.; Cohen, A. J.; Yang, W. Revealing Noncovalent Interactions. *J. Am. Chem. Soc.* **2010**, *132*, 6498-6506.
(24) Lu, T.; Chen, F. Multiwfn: A multifunctional wavefunction analyzer. *J. Comput. Chem.* **2012**, *33*, 580-592.
(25) Lu, T.; Chen, Q. Simple, Efficient, and Universal Energy Decomposition Analysis Method Based on Dispersion-Corrected Density Functional Theory. *J. Phys. Chem. A* **2023**, *127*, 7023-7035.
(26) Liao, K.; Liu, W.; Niemeyer, Z. L.; Ren, Z.; Bacsa, J.; Musaev, D. G.; Sigman, M. S.; Davies, H. M. L. Site-Selective Carbene-Induced C–H Functionalization Catalyzed by Dirhodium Tetrakis(triarylcyclopropanecarboxylate) Complexes. *ACS Catal.* **2018**, *8*, 678-682.
(27) Ren, Z.; Musaev, D. G.; Davies, H. M. L. Influence of Aryl Substituents on the Alignment of Ligands in the Dirhodium Tetrakis(1,2,2-Triarylcyclopropane- carboxylate) Catalysts. *ChemCatChem* **2021**, *13*, 174-179.

10.1. Structure of Rh_2(*S*-pPhTPCP)$_4$

The optimization structures were built upon the reported X-ray structure of Rh_2(*S*-pPhTPCP)$_4$.[26] The overlay of the DFT-optimized structure and Xray structure are shown in **Figure S2**. The DFT-optimized structure shown a good alignment with the experimental obtain structure, supporting our selection of computational methods. As previous computational study in our laboratory on Rh_2(TPCP)$_4$ class showed that the most energetically favorable conformation of Rh_2(*S*-pPhTPCP)$_4$ catalyst is (α,α,α,α') configuration,[27] the subsequent analysis on Rh_2(*S*-pPhTPCP)$_4$ system will only start with this (α,α,α,α') configuration

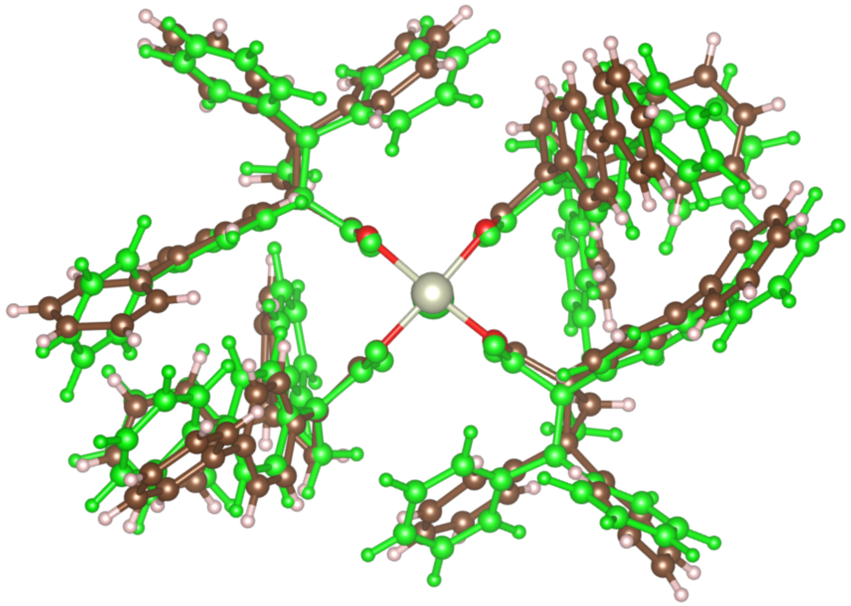

Figure S2. A comparison of a DFT-optimized structure (Green) and Xray structure (Colorful) of Rh_2(*S*-pPhTPCP)$_4$.

10.2. A Model studies of cyclopropanation reaction with $Rh_2(HCOO)_4$ as a catalyst

The cyclopropanation reaction was first studied using $Rh_2(HCOO)_4$ as a chiral catalyst. (**Figure S3**) The substrate can either approach the metal-carbene intermediate **S1** from the carbonyl side (**TS-S3**, **TS-S5**) or trichloroethyl side (**TS-S2**, **TS-4**). The results show that the former approach is more energetically more favored in the formation of both diastereomers. With the achiral catalyst $Rh_2(HCOO)_4$, the transition states leading to the

major diastereomer and the minor diastereomer are energetically equivalent, indicating a non-selective reaction. The cyclopanation barrier which is 4-5 kcal/mol is much lower than the rotational barrier of the ester group, indicating that the reaction is under non-Curtin-Hammet control.

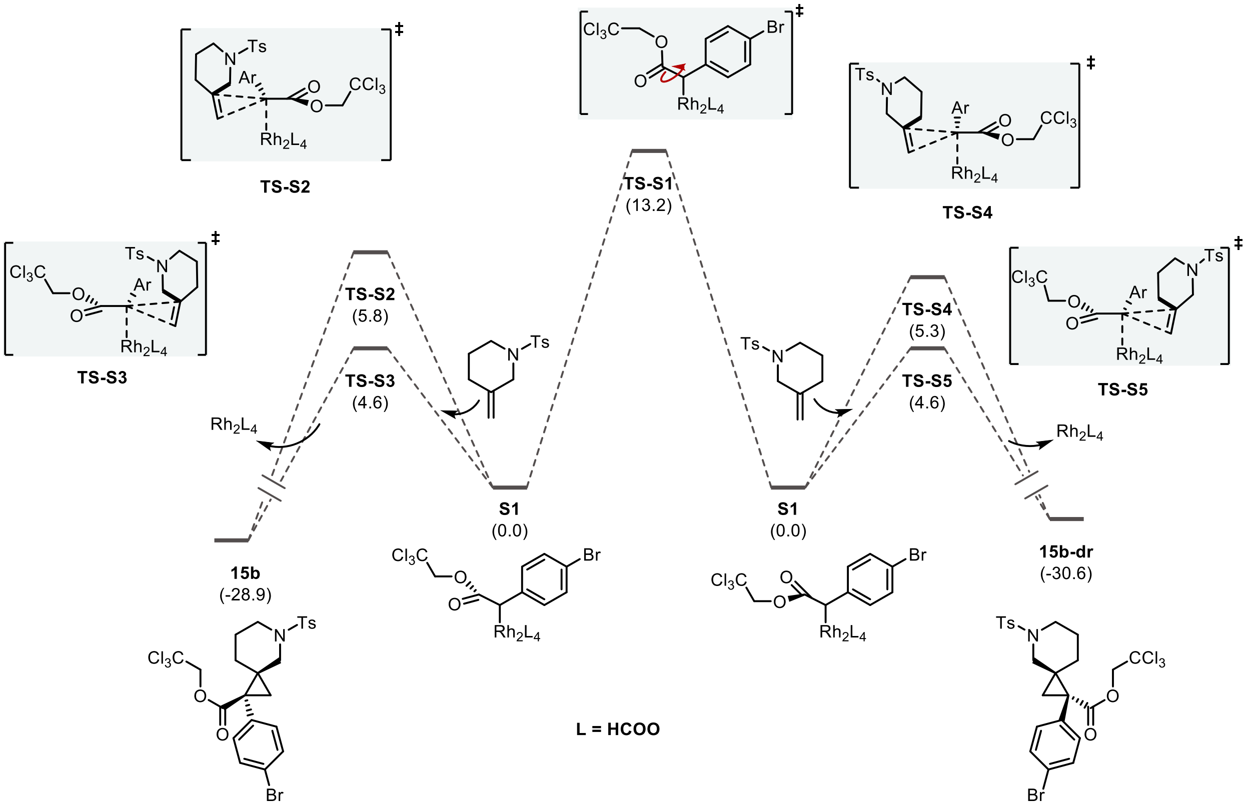

Figure S3. A model cyclopropanation study with $Rh_2(HCOO)_4$. All reported energies are free Gibbs energy in kcal/mol.

10.3. Transition states of cyclopropanation of Rh_2(*S*-pPhTPCP)$_4$

As demonstrated by distortion-interaction analysis, the distortion component of rhodium-carbene fragment was a major factor making **TS3** and **TS1** energetically disfavored than **TS2**. The energy decomposition of **TS2** and **TS4** showed that the Pauli-exchange repulsion was one of the critical factors destabilizing **TS4** over **TS2**. Both the distortion component and the Pauli-exchange repulsion are rooted from steric repulsion. Therefore, RDG maps were generated to analyze the attractive and repulsive region in **TS1**-**TS4**. (**Figure S4**) There is a significant steric repulsion in **TS1** between the *N*-tosyl group of the substrate and the aryl of carbene fragment with the catalyst wall, which explained its

highest Gibbs energy. Similarly, **TS3** has steric repulsion between the *N*-Tosyl group and catalyst wall, and **TS4** has steric repulsion between the aryl of the carbene and catalyst wall.

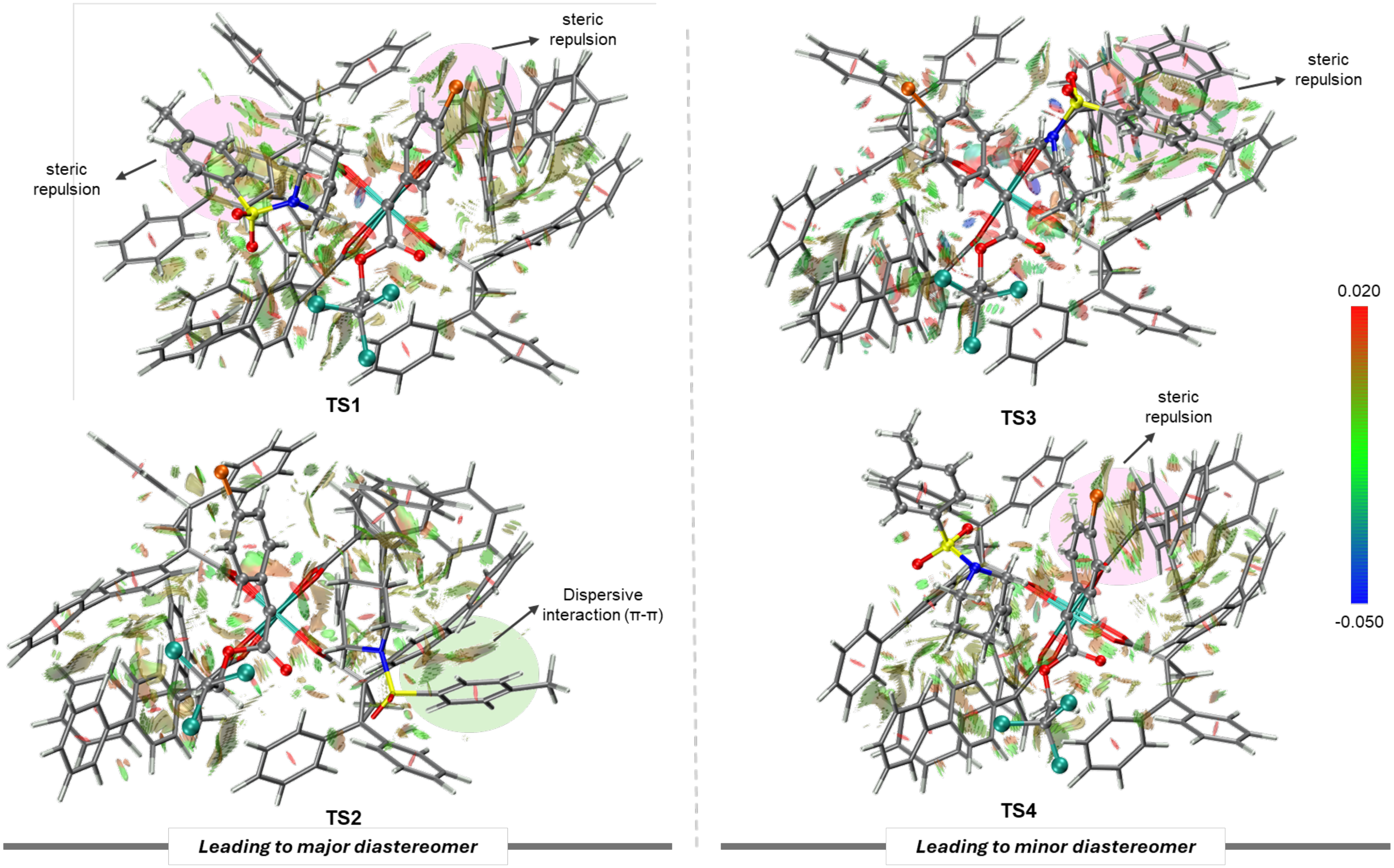

Figure S4. RDG maps of transition states leading to 4 possible stereoisomers. Isovalue = 0.40, color bar: -0.05 to 0.02. Pink highlighted regions are repulsive. Green highlighted regions are attractive. The RDG maps were generated by Multiwfn and VMD programs.

10.4. Summary of energy and coordination

Table S7. Zero-point correction (ZPE), thermal correction to enthalpy (TCH), thermal correction to Gibbs free energy (TCG), energies (E), enthalpies (H), and Gibbs free energies (G) (in Hartree) of the structures calculated at the [B3LYP-D3(BJ) +CPCM(CH_2Cl_2)] level of theory

Structure	ZPE	TCH	TCG	E	H	G	Imaginary Frequency
**8b**	0.282180	0.299501	0.235559	-1109.022292	-1108.722790	-1108.786733	-
**15b**	0.425404	0.457669	0.357275	-5596.200962	-5595.743294	-5595.843687	-
**15b-dr**	0.425512	0.458715	0.354638	-5596.201090	-5595.742375	-5595.846452	-
**$Rh_2(OOCH)_4$**	0.101153	0.116108	0.060641	-975.888674	-975.772566	-975.828034	-
**S1**	0.240552	0.272840	0.174074	-5463.013048	-5462.740208	-5462.838974	-
**TS-S1**	0.240400	0.271791	0.175644	-5462.993615	-5462.721825	-5462.817971	52.70i
**TS-S2**	0.524391	0.573882	0.435246	-6572.051729	-6571.477846	-6571.616483	80.41i
**TS-S3**	0.524213	0.573671	0.435194	-6572.053620	-6571.479949	-6571.618426	119.55i
**TS-S4**	0.524187	0.573811	0.434489	-6572.051799	-6571.477988	-6571.617310	127.75i
**TS-S5**	0.524148	0.573687	0.434356	-6572.052724	-6571.479037	-6571.618368	123.26i
**Rh_2(*S*-pPhTPCP)$_4$ (I)**	1.648807	1.750369	1.501859	-5140.200981	-5138.450612	-5138.699122	-
**II**	1.789877	1.908279	1.620919	-9627.357376	-9625.449098	-9625.736458	-
**III**	1.789526	1.907989	1.621752	-9627.356287	-9625.448298	-9625.734535	-
**TS1**	2.075551	2.210051	1.894609	-10736.414027	-10734.203976	-10734.519418	120.32i
**TS2**	2.075865	2.210405	1.892444	-10736.422560	-10734.212155	-10734.530116	26.28i
**TS3**	2.074559	2.209247	1.891962	-10736.414163	-10734.204917	-10734.522201	89.65i
**TS4**	2.074387	2.209571	1.886167	-10736.411412	-10734.201841	-10734.525246	45.81i

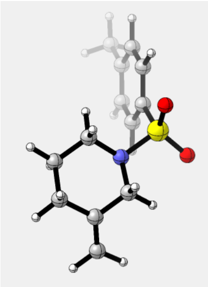

8b

C	-6.09021500	-1.35793300	0.27264600
C	-4.64542100	-1.17023300	-0.22151800
C	-4.47175700	0.88171500	1.25322700
C	-6.27066900	-0.76397900	1.66885400
H	-3.98037000	-1.77796200	0.40957900
H	-4.53505300	-1.52641900	-1.24957900
H	-6.78351600	-0.86147100	-0.41495200
H	-6.35043900	-2.42070800	0.30175400
H	-3.80478700	0.40618100	1.99098300
H	-4.27879700	1.95352500	1.25117500
H	-5.65876300	-1.30701900	2.40379700
H	-7.31514800	-0.83612000	1.98073000
C	-4.24047700	0.27769600	-0.11028400
C	-3.73022100	0.99064200	-1.11576700
H	-3.55292500	0.54526800	-2.09086900
H	-3.46393500	2.03693900	-0.99523500
N	-5.89010900	0.66310300	1.63431400
S	-6.39322700	1.54119200	2.98253600
O	-5.83406500	2.89409800	2.82498400
O	-6.13688400	0.82150800	4.24542000
C	-8.15280500	1.57838600	2.71469000
C	-9.00643200	1.08896000	3.70001800
C	-8.64987200	2.13551800	1.53210700
C	-10.38455900	1.15809900	3.49348500
H	-8.59418000	0.65813600	4.60483900
C	-10.02492300	2.19316000	1.34346500
H	-7.96781900	2.50162400	0.77318200
C	-10.91265600	1.70820900	2.31974500
H	-11.05721500	0.77555900	4.25498900
H	-10.42081400	2.61817900	0.42576700
C	-12.40113700	1.78849100	2.09845100
H	-12.95172600	1.28259900	2.89477000
H	-12.73372300	2.83205400	2.06646000
H	-12.68130800	1.33288000	1.14311900

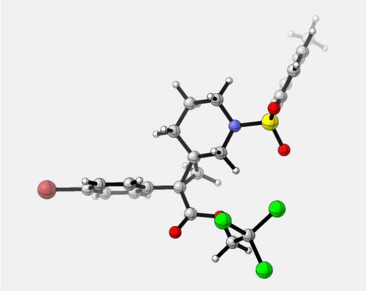

15b

C	-3.29745800	0.89135500	-0.03367800
C	-2.35413300	1.74300100	0.75616800
O	-2.03538900	2.92188900	0.13986300
C	-1.12096300	3.77277200	0.82236900
H	-0.40146400	4.13586700	0.08781300
H	-0.61888900	3.22710100	1.62084400
O	-1.88131600	1.41926600	1.82471000
C	-3.42277900	-0.48290300	0.56597000
C	-2.84654500	-1.59816300	-0.04625300
C	-2.96454300	-2.86967100	0.51872800
C	-3.66656200	-3.01231300	1.71162200
C	-4.24435800	-1.91549700	2.35049600
C	-4.11458900	-0.65592700	1.77193900
H	-4.55653600	0.20471800	2.26317800
H	-4.78553300	-2.04370500	3.28039800
H	-2.51606600	-3.73064400	0.03744100
H	-2.29602000	-1.48147500	-0.97387600
C	-3.37741300	1.05822000	-1.54942600
H	-3.45116900	0.14722500	-2.13406800
H	-2.75831800	1.83436600	-1.98463500
C	-4.55289700	1.47663500	-0.73295500
C	-5.80005300	0.59955300	-0.72787900
C	-4.89603700	2.95618300	-0.64833500
C	-6.78176200	1.04446600	-1.81784800
H	-6.28426400	0.67692200	0.25418400
H	-5.53474000	-0.44986700	-0.86802900
H	-5.38421100	3.17867500	0.31208000
H	-4.01421200	3.58087600	-0.74338800
N	-5.80177300	3.26112200	-1.77666600
C	-7.08390300	2.53603000	-1.71000600
H	-6.35386700	0.84547400	-2.80665800
H	-7.71461300	0.47765000	-1.73724900
S	-5.92535100	4.88193100	-2.20656700
H	-7.60721300	2.76614700	-0.77064100
H	-7.71739000	2.85893500	-2.53837600
O	-4.55310400	5.41404700	-2.23063700
O	-6.93991800	5.59844100	-1.40992600
C	-6.53247900	4.73036700	-3.87395900
C	-7.70892000	5.38081900	-4.23661800
C	-5.79127400	3.99374300	-4.80349200
C	-8.15083300	5.28844200	-5.55678100
H	-8.26653000	5.94015200	-3.49461500
C	-6.24937500	3.91035800	-6.11231000
H	-4.88373600	3.48608700	-4.49705500
C	-7.43286000	4.55669000	-6.50980000
H	-9.06893900	5.78965300	-5.84750100
H	-5.68481200	3.33508000	-6.84022500
C	-7.90753000	4.46056000	-7.93681800
H	-8.88492000	4.93101800	-8.06554200
H	-7.20207200	4.95312600	-8.61528600
H	-7.98375000	3.41598000	-8.25590100
C	-1.84240000	4.98532900	1.42648000
Cl	-3.06404600	4.46262400	2.63954900
Cl	-0.58849600	5.99769900	2.23209300
Cl	-2.66219300	5.92613600	0.14589000
Br	-3.84396500	-4.75085100	2.49398800

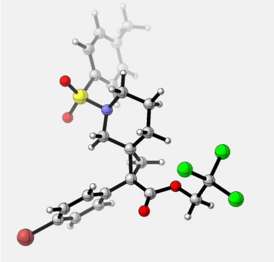

15b-dr

C	-3.29277600	1.00438600	-0.18424600

C	-2.37780900	1.84524600	0.65196300
O	-1.94467900	2.96697700	-0.00101500
C	-1.04735500	3.81859400	0.69820700
H	-0.21503500	4.04306800	0.02969500
H	-0.69189200	3.33435900	1.60791800
O	-2.02390500	1.56605900	1.77706800
C	-3.36041300	-0.41768300	0.30001000
C	-2.78902900	-1.45887600	-0.43615200
C	-2.86046300	-2.77915000	0.01170700
C	-3.51040700	-3.04709800	1.21276500
C	-4.08261500	-2.02759700	1.97268800
C	-4.00057900	-0.71670400	1.51025800
H	-4.43968700	0.08386000	2.09547300
H	-4.58497200	-2.25367500	2.90570000
H	-2.41738300	-3.58193200	-0.56546600
H	-2.28136700	-1.24394400	-1.37056800
C	-3.44521400	1.31033000	-1.66815500
H	-3.55029100	0.45887100	-2.33240600
H	-2.85376600	2.12851200	-2.06093600
C	-1.72905100	5.13922200	1.07590600
Cl	-3.10042200	4.85204200	2.19812900
Cl	-0.49164500	6.16437300	1.88403000
Cl	-2.33409900	5.98102100	-0.39495000
Br	-3.62360000	-4.85389900	1.83504700
C	-4.58423500	1.63640000	-0.75925700
C	-4.97772100	3.07892800	-0.49087200
C	-5.79705700	0.72111400	-0.80942600
C	-6.00148700	3.53768300	-1.53880400
H	-5.41874300	3.15074300	0.51163900
H	-4.10976400	3.73436300	-0.51320800
H	-6.30274700	0.71239400	0.16780300
H	-5.51995300	-0.30041400	-1.05907100
N	-6.69588700	1.22514500	-1.86421800
C	-7.19674700	2.58927200	-1.60813900
H	-5.52452900	3.57595700	-2.52448200
H	-6.35701200	4.54539900	-1.30225200
S	-7.86013000	0.13389300	-2.39930400
H	-7.77148200	2.61622600	-0.67122300
H	-7.86533900	2.87463000	-2.42350100
O	-7.19204100	-1.17622000	-2.47338200
O	-9.11658100	0.23780600	-1.63308500
C	-8.16033700	0.75502900	-4.04024700
C	-9.45928900	1.07838600	-4.42384200
C	-7.08825200	0.86984300	-4.93110000
C	-9.68607600	1.52686000	-5.72540100
H	-10.27065700	0.98581900	-3.71153800
C	-7.33361000	1.32025000	-6.22203500
H	-6.08278000	0.62501100	-4.60807000
C	-8.63399900	1.65309600	-6.63994800
H	-10.69514900	1.78510900	-6.03162000
H	-6.50685700	1.41923800	-6.91932300
C	-8.88002500	2.12926000	-8.04836900
H	-8.21118800	2.95667200	-8.30630500
H	-9.91065200	2.46547000	-8.18246000
H	-8.68982800	1.32560900	-8.76868500

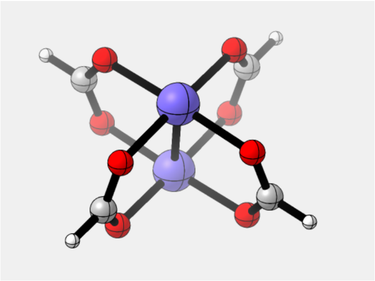

$Rh_2(HCOO)_4$

Rh	0.34227900	-0.12304000	0.45907600
O	0.49531700	1.72208700	-0.46539200
C	1.53531100	1.96327400	-1.14773200
O	2.52412200	1.19589900	-1.34716300
Rh	2.48688100	-0.68036800	-0.47992700
O	1.49574900	-1.42655700	-2.13559800
C	0.22965400	-1.36820000	-2.14589000
O	-0.53049400	-0.89949700	-1.24657700
H	-0.26739900	-1.77060800	-3.03804100
O	3.35972300	0.09600500	1.22577500
C	2.59951900	0.56480800	2.12503000
O	1.33346400	0.62322600	2.11466200
H	3.09656300	0.96722800	3.01718600
O	2.33385600	-2.52555900	0.44449600
C	1.29387000	-2.76665300	1.12695800
O	0.30514700	-1.99923600	1.32640100
H	1.24366800	-3.75558900	1.60068200
H	1.58547800	2.95217600	-1.62153300

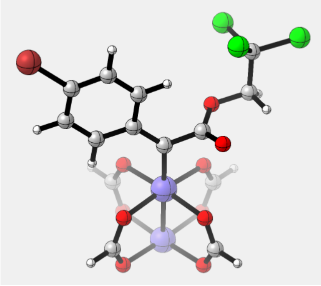

S1

Rh	0.30288100	-0.11448500	0.46937900
C	-1.52174500	0.36240400	1.20993100
C	-1.59508100	1.74565900	1.71950600
O	-2.01102000	2.59534600	0.74891400
C	-1.92479900	3.99219200	1.01592700
H	-1.39698400	4.45384700	0.18086100
H	-1.39900200	4.17296400	1.95391200
O	-1.26849900	2.06605100	2.84266100
C	-2.68318900	-0.44124200	1.26797200
C	-2.66776200	-1.78152500	0.78291900
C	-3.81032400	-2.55915400	0.81651500
C	-4.98960900	-2.01458900	1.34024900
C	-5.04904000	-0.70141600	1.82970900
C	-3.90811800	0.07403100	1.79048700
H	-3.95076100	1.09075300	2.16339300
H	-5.97630800	-0.30794500	2.22691400
H	-3.79933000	-3.57627300	0.44512000
H	-1.74974000	-2.18564900	0.38366300
O	0.49995900	1.73551600	-0.45333100
C	1.54688600	1.96782100	-1.13688900
O	2.52484700	1.20241100	-1.35374800
Rh	2.50867900	-0.68870800	-0.49388600

O	1.49312100	-1.43865900	-2.14721500
C	0.23461200	-1.37882700	-2.15385600
O	-0.53595600	-0.90201200	-1.25996700
H	-0.27513700	-1.78603800	-3.03793300
O	3.36711900	0.09923800	1.22402000
C	2.60283300	0.56572900	2.11246800
O	1.33288600	0.62333800	2.10955700
H	3.08499400	0.97806200	3.00962500
O	2.33491700	-2.53731200	0.44459000
C	1.30192300	-2.76875000	1.12708100
O	0.30801100	-2.00077600	1.33580600
H	1.23796200	-3.75368200	1.60966600
H	1.59217100	2.96653000	-1.59295300
C	-3.32969200	4.59745200	1.10517500
Cl	-4.23073200	3.90050700	2.49808100
Cl	-3.13707100	6.36764200	1.34034300
Cl	-4.24719300	4.28248600	-0.40592700
Br	-6.55099100	-3.07863100	1.38778700

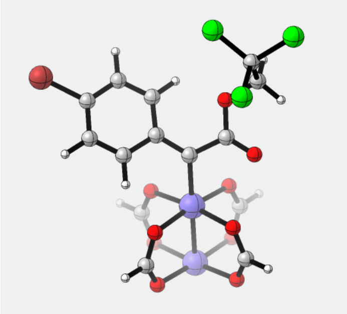

TS-S1

Rh	0.30042300	-0.11965400	0.59852700
C	-1.44991800	0.43825200	1.43258100
C	-1.47829900	1.85762100	1.95418900
O	-2.72804800	2.37354100	2.12486100
C	-2.85458700	3.62097100	2.79736600
H	-3.46446600	4.28011800	2.17849100
H	-1.87539500	4.06232400	2.98140400
O	-0.47872700	2.47185400	2.24784700
C	-2.62893700	-0.36025600	1.34081200
C	-2.46634300	-1.77853000	1.38417400
C	-3.54870000	-2.63617900	1.31475400
C	-4.82658100	-2.09871000	1.13396600
C	-5.03280400	-0.71645100	1.03832500
C	-3.95602200	0.14080700	1.15994600
H	-4.12574800	1.20185900	1.07833900
H	-6.02922800	-0.32484300	0.87492600
H	-3.40981100	-3.70801300	1.38325600
H	-1.47382200	-2.18592000	1.51489500
O	0.25930200	1.67173500	-0.45416000
C	1.15985200	1.90672900	-1.31979900
O	2.12462700	1.17262400	-1.66459200
Rh	2.32202100	-0.67406200	-0.73835700
O	1.05513700	-1.52223400	-2.15269400
C	-0.18464000	-1.50035700	-1.92956200
O	-0.79657400	-1.00631000	-0.92984600
H	-0.83185000	-1.96323900	-2.68748900
O	3.45218200	0.20027000	0.76384700
C	2.84929400	0.63502300	1.78482600
O	1.60258800	0.64206700	2.02192200
H	3.47676800	1.06546300	2.57793300
O	2.38875100	-2.48751000	0.27891800
C	1.51997900	-2.71405000	1.16059100

O	0.56337200	-1.95936900	1.53263500
H	1.58314600	-3.67698500	1.68545500
H	1.07415700	2.87878000	-1.82503500
C	-3.57197900	3.39262600	4.13276200
Cl	-2.60233400	2.29842000	5.17754100
Cl	-3.75930600	4.99068300	4.92932400
Cl	-5.19087100	2.66033700	3.85475700
Br	-6.31155500	-3.26323300	1.00142100

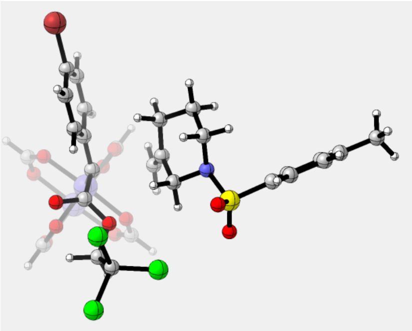

TS-S2

Rh	0.01245100	0.00878000	0.15292200
C	-1.98400600	0.44276700	0.62648300
C	-2.00958300	1.71009500	1.39515600
O	-1.81096400	2.83849400	0.67137600
C	-1.32460300	3.96315100	1.40328500
H	-0.47680100	4.36333600	0.84626300
H	-1.01812000	3.65481400	2.40165800
O	-2.03087100	1.68147800	2.61233200
C	-2.97877600	-0.55309900	0.93144500
C	-2.85440500	-1.86060700	0.40115700
C	-3.78937000	-2.84232500	0.68795000
C	-4.86407100	-2.53418700	1.52743100
C	-5.02509900	-1.25744600	2.07022500
C	-4.09798800	-0.27353900	1.75923300
H	-4.22503100	0.71716400	2.17647200
H	-5.86876500	-1.03681300	2.71248500
H	-3.68853100	-3.83794700	0.27344600
H	-2.02078100	-2.08709800	-0.24862500
O	0.31330100	1.58671000	-1.16875800
C	1.45506600	1.73360600	-1.70583300
O	2.49035000	1.03080400	-1.54438800
Rh	2.39168500	-0.57459200	-0.22838700
O	1.75451600	-1.80793900	-1.77717900
C	0.52194800	-1.86746500	-2.02805200
O	-0.42802500	-1.25574300	-1.44181100
H	0.21591600	-2.52425600	-2.85385800
O	2.87192600	0.70187300	1.33662300
C	1.93390900	1.31459900	1.91526800
O	0.68668700	1.24504200	1.68194400
H	2.21880800	1.99733200	2.72771300
O	2.13030400	-2.13735600	1.11744100
C	0.99408800	-2.29089900	1.64294000
O	-0.05698600	-1.59962700	1.46222700
H	0.88748900	-3.12638400	2.34879100
H	1.54142900	2.58373900	-2.39687500
C	-2.61225500	0.97593400	-1.68987000
H	-2.35986600	-0.05620300	-1.89816800
H	-1.82011100	1.70444300	-1.80433000
C	-3.89433900	1.35430800	-1.48186700
C	-5.03469600	0.39117800	-1.37260600
C	-4.26579900	2.80494500	-1.32977500

C	-6.22055500	0.82029700	-2.25718400
H	-5.37182300	0.39009300	-0.32446300
H	-4.71411600	-0.62597200	-1.60786400
H	-4.58682500	3.00853800	-0.29579800
H	-3.42203700	3.44858200	-1.56018600
N	-5.36411000	3.09574300	-2.28101300
C	-6.57209000	2.28792400	-2.02502900
H	-5.96421500	0.68162300	-3.31246000
H	-7.09279500	0.19776200	-2.03791600
S	-5.64966800	4.73942500	-2.53693500
H	-6.93398600	2.44940500	-0.99932700
H	-7.35727200	2.60170700	-2.71566600
O	-4.33080200	5.35586300	-2.75834500
O	-6.53151900	5.31730600	-1.50554600
C	-6.54547700	4.68088500	-4.07334400
C	-7.82287600	5.23097000	-4.14251500
C	-5.93326100	4.11400500	-5.19574800
C	-8.49956400	5.20984700	-5.36235500
H	-8.27427500	5.66010600	-3.25573300
C	-6.62462400	4.09974500	-6.40044800
H	-4.94092500	3.68459900	-5.11778400
C	-7.91519000	4.64774300	-6.50341900
H	-9.49695900	5.63389000	-5.42561600
H	-6.16087400	3.65715800	-7.27707600
C	-8.64372500	4.62955000	-7.82232900
H	-9.66442800	5.00484700	-7.72045900
H	-8.12570100	5.25126700	-8.56102100
H	-8.68913000	3.61509200	-8.23170600
C	-2.38113900	5.06670300	1.53157500
Cl	-3.86132000	4.44843100	2.34484100
Cl	-1.64533400	6.37411100	2.53104000
Cl	-2.82964100	5.71566600	-0.07405000
Br	-6.14203500	-3.88048100	1.93101600

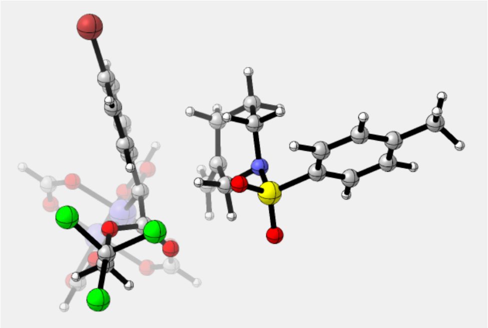

TS-S3

Rh	0.08544400	-0.22822100	0.41875500
C	-1.90127600	0.40999300	0.37368600
C	-1.95324100	1.83245500	0.81836100
O	-2.24715600	1.86848700	2.15264400
C	-2.14369400	3.12872300	2.80404900
H	-1.67289900	3.86204500	2.14959100
H	-1.55774700	2.98609000	3.71229300
O	-1.67560000	2.81920600	0.17165900
C	-3.01719600	-0.46390500	0.62709400
C	-2.91266800	-1.84179400	0.31072300
C	-3.95664400	-2.71822200	0.56297100
C	-5.13035300	-2.23059100	1.14433200
C	-5.28341800	-0.87707000	1.45294200
C	-4.24069400	-0.00276700	1.18203900
H	-4.36773700	1.04147600	1.43008100
H	-6.20445300	-0.51339700	1.89147800
H	-3.86530500	-3.76888600	0.31605600
H	-2.00449200	-2.20593800	-0.14896800
O	0.80213500	1.12413500	-0.98289800
C	2.04284700	1.12985700	-1.25475000
O	2.95157600	0.39814200	-0.77377200
Rh	2.42375800	-1.01001500	0.65974000
O	2.02643800	-2.39153700	-0.84473300
C	0.87812500	-2.40117000	-1.36225100
O	-0.11671700	-1.65763400	-1.08158600
H	0.70045900	-3.13825400	-2.15739200
O	2.66395200	0.42365300	2.14167100
C	1.68185600	1.16299200	2.42245400
O	0.51573000	1.14735700	1.91818300
H	1.84277000	1.92023900	3.20226300
O	1.73529500	-2.36261200	2.08016400
C	0.50369100	-2.36690100	2.35231100
O	-0.41321800	-1.63599000	1.86266000
H	0.16962800	-3.09147400	3.10805800
H	2.35388800	1.86954800	-2.00559700
C	-1.99944000	0.79528600	-2.02019000
H	-1.56943900	-0.16805100	-2.26974400
H	-1.31499400	1.61891000	-1.87193600
C	-3.34239100	0.97888800	-2.06134400
C	-4.30144600	-0.13743500	-2.32847000
C	-3.96144700	2.29018800	-1.66549900
C	-5.49743200	0.32321800	-3.17741100
H	-4.68765800	-0.47302800	-1.35301300
H	-3.78960800	-0.99034000	-2.77978700
H	-4.31478900	2.18856800	-0.62509600
H	-3.23342400	3.09594200	-1.69089700
N	-5.10574400	2.64143500	-2.53762100
C	-6.13097900	1.57647800	-2.58078000
H	-5.17254700	0.53857300	-4.20034100
H	-6.24737800	-0.47129100	-3.22438400
S	-5.72269900	4.16468300	-2.10037500
H	-6.52155100	1.37199500	-1.57312800
H	-6.95851400	1.91858700	-3.20480400
O	-4.58138200	5.09166900	-2.13606300
O	-6.51643900	4.08198300	-0.85678100
C	-6.82132300	4.50019600	-3.45534200
C	-8.18818500	4.62278900	-3.21516500
C	-6.28556000	4.66786200	-4.73589100
C	-9.03404500	4.91649300	-4.28401600
H	-8.57406600	4.49035300	-2.21132400
C	-7.14608000	4.95591000	-5.78807600
H	-5.21825400	4.57217200	-4.89973600
C	-8.53018300	5.08365400	-5.57989500
H	-10.10047100	5.01675100	-4.10733200
H	-6.74191900	5.08793500	-6.78726300
C	-9.44828600	5.37951700	-6.73740200
H	-10.44745000	5.65736700	-6.39427100
H	-9.05494100	6.19305800	-7.35451200
H	-9.54750600	4.50152000	-7.38607500
C	-3.54061900	3.62984400	3.19325500
Cl	-4.35247700	2.43625000	4.26892700
Cl	-3.32574400	5.18393100	4.06937600
Cl	-4.56381800	3.89180000	1.73823900
Br	-6.55753500	-3.43109000	1.50501800

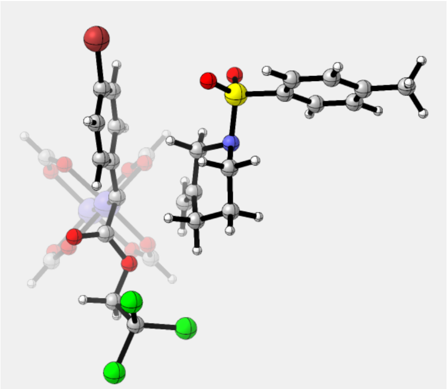

TS-S4

Rh	-0.05795100	0.05047700	0.26510800
C	-2.06667400	0.60698200	0.53888700
C	-2.08890100	1.81033600	1.41224200
O	-1.63941200	2.94194300	0.81751400
C	-1.16245600	3.97023300	1.67640100
H	-0.12873500	4.17691800	1.39508900
H	-1.22214700	3.65624900	2.71794000
O	-2.32383400	1.72445900	2.60377200
C	-3.14136800	-0.34928400	0.66535600
C	-3.04965200	-1.59548200	-0.00033300
C	-4.08238800	-2.51956300	0.05782000
C	-5.22634200	-2.21747000	0.80070800
C	-5.35356900	-1.00345300	1.47978600
C	-4.32511600	-0.07432600	1.39925200
H	-4.42872100	0.86720200	1.92205400
H	-6.25085100	-0.78284200	2.04471800
H	-4.00771600	-3.46206200	-0.47056800
H	-2.16888000	-1.81269800	-0.58845700
O	0.51321900	1.71712800	-0.84142100
C	1.72455200	1.83291800	-1.20357800
O	2.68514000	1.04423800	-0.98392000
Rh	2.30516100	-0.67735600	0.11356800
O	1.79736800	-1.67999600	-1.63553000
C	0.60506500	-1.61998600	-2.03711400
O	-0.37352600	-1.00686700	-1.50100100
H	0.36767400	-2.15862600	-2.96470100
O	2.65786800	0.38392500	1.86525200
C	1.69587900	1.00990200	2.38706700
O	0.48931500	1.06973900	1.99222800
H	1.91609300	1.57901600	3.30091300
O	1.76854800	-2.35093300	1.22402400
C	0.56851700	-2.46083100	1.59716300
O	-0.39978100	-1.66716100	1.37934200
H	0.31687400	-3.35356900	2.18655400
H	1.95637100	2.73867100	-1.78143700
C	-2.34249800	1.49570500	-1.65875600
H	-1.93174400	0.59777300	-2.10475000
H	-1.64958100	2.29560800	-1.43924400
C	-1.98513900	5.24735600	1.49574000
Cl	-3.69984600	4.96326100	1.94668500
Cl	-1.27427100	6.49746300	2.57442300
Cl	-1.90252700	5.81556500	-0.20959100
Br	-6.65240800	-3.47023200	0.86408200
C	-3.68568800	1.68390600	-1.58928800
C	-4.32525600	2.92564600	-1.04862200
C	-4.61889000	0.56272700	-1.93551700
C	-5.55349200	3.32633700	-1.88024000
H	-4.66532300	2.71271600	-0.02297500
H	-3.60051400	3.73620000	-0.98274400
H	-4.94213600	0.10793300	-0.98485800
H	-4.11693200	-0.20266700	-2.52349400
N	-5.80115700	1.03824400	-2.69076500
C	-6.51252700	2.14922400	-2.02290600
H	-5.24079600	3.65943100	-2.87521200
H	-6.07307500	4.15825000	-1.39665700
S	-6.81949100	-0.25873900	-3.09970500
H	-6.88863900	1.83498500	-1.03877400
H	-7.36869800	2.42593700	-2.64164400
O	-5.94654400	-1.29310100	-3.67672000
O	-7.69826000	-0.62928900	-1.97412200
C	-7.81179800	0.48105100	-4.37488700
C	-9.18695300	0.60431300	-4.18883600
C	-7.19349900	0.90423500	-5.55535900
C	-9.95550800	1.16581200	-5.20797900
H	-9.63830000	0.27055600	-3.26194300
C	-7.97634500	1.46412700	-6.55742300
H	-6.12111400	0.80337800	-5.67782300
C	-9.36638600	1.60120800	-6.40144000
H	-11.02753900	1.26932700	-5.07145900
H	-7.50617300	1.80215400	-7.47604700
C	-10.20118800	2.19066500	-7.50865600
H	-9.72744500	3.08316300	-7.92834000
H	-11.19922500	2.46101300	-7.15614400
H	-10.31849900	1.47172700	-8.32785300

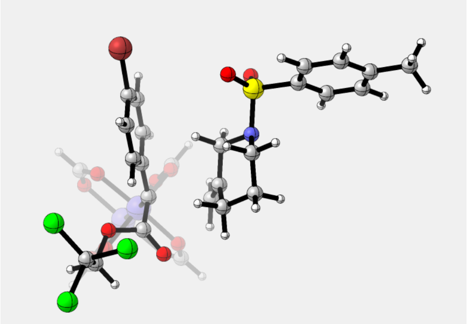

TS-S5

Rh	-0.23487800	0.16364300	0.30806600
C	-2.25695800	0.66028800	0.53626300
C	-2.35798600	1.90819000	1.34121900
O	-2.47604200	1.55140000	2.65683500
C	-2.30941500	2.57565100	3.62635700
H	-1.98102500	3.50226200	3.15556900
H	-1.57558400	2.22989600	4.35500200
O	-2.24351300	3.05477100	0.96685800
C	-3.30835100	-0.32077800	0.62535300
C	-3.16708600	-1.56872500	-0.03049500
C	-4.17655200	-2.51851300	0.00911700
C	-5.34832300	-2.24008000	0.71813800
C	-5.52769000	-1.02313600	1.38058400
C	-4.52109600	-0.06993200	1.32070700
H	-4.67193900	0.87388200	1.82770300
H	-6.44595700	-0.81997100	1.91749900
H	-4.06379000	-3.46328400	-0.50828000
H	-2.26591400	-1.76609300	-0.59393100
O	0.31371800	1.86859900	-0.74266300
C	1.52944000	2.01940900	-1.07829700
O	2.50237100	1.24668900	-0.85787300
Rh	2.14602200	-0.50959300	0.19395900

O	1.69489200	-1.48487500	-1.58644400
C	0.51088400	-1.44037200	-2.01322200
O	-0.49128200	-0.85762000	-1.48660700
H	0.30434800	-1.96443500	-2.95638300
O	2.43893400	0.51430800	1.97761100
C	1.45140800	1.10339200	2.49536400
O	0.25293100	1.14800100	2.07610600
H	1.63942300	1.65005400	3.42995100
O	1.62768200	-2.21977800	1.25461500
C	0.42237800	-2.36511100	1.59770800
O	-0.55860200	-1.58859000	1.37579700
H	0.17835800	-3.27654400	2.16113400
H	1.75150900	2.94308300	-1.63075900
C	-2.51438600	1.61421400	-1.65099900
H	-2.03324100	0.76183700	-2.11572200
H	-1.88230800	2.44056800	-1.35718000
C	-3.63980600	2.82411900	4.34588300
Cl	-4.22668300	1.31101000	5.11827700
Cl	-3.35280400	4.07466900	5.60176200
Cl	-4.88601400	3.40786000	3.18455300
Br	-6.74056000	-3.52973900	0.75500800
C	-3.86701200	1.71649400	-1.62167500
C	-4.60649200	2.89113400	-1.05611900
C	-4.71363500	0.55362800	-2.04622500
C	-5.81879400	3.25073600	-1.92976000
H	-4.97919700	2.60681200	-0.05895600
H	-3.93618400	3.73901100	-0.91313200
H	-5.04543900	0.04273200	-1.12747600
H	-4.14113300	-0.15622100	-2.63977200
N	-5.89270500	0.98369900	-2.83349600
C	-6.69725600	2.02503400	-2.15899500
H	-5.48358300	3.64012800	-2.89660900
H	-6.41099700	4.03022100	-1.44216500
S	-6.81319700	-0.35635000	-3.32633300
H	-7.09592900	1.65163700	-1.20482400
H	-7.54114100	2.27465800	-2.80560400
O	-5.85720400	-1.31609500	-3.90062900
O	-7.71132800	-0.81740300	-2.25041100
C	-7.79779500	0.36562600	-4.61761700
C	-9.18238500	0.42255500	-4.47471900
C	-7.16177300	0.84410900	-5.76714600
C	-9.94260800	0.97283000	-5.50620800
H	-9.64766400	0.04646800	-3.57121200
C	-7.93669400	1.39135400	-6.78224600
H	-6.08258700	0.79352000	-5.85654600
C	-9.33586000	1.46216200	-6.66953300
H	-11.02210700	1.02460100	-5.40325700
H	-7.45321800	1.77121900	-7.67732800
C	-10.16040300	2.03960100	-7.79067900
H	-10.21092600	1.33948900	-8.63267800
H	-9.71899400	2.96673500	-8.16851800
H	-11.18298100	2.24871500	-7.46816800

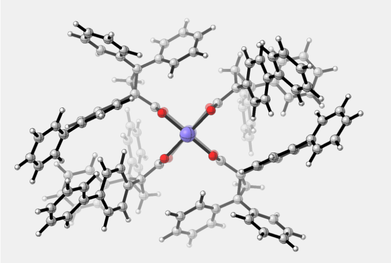

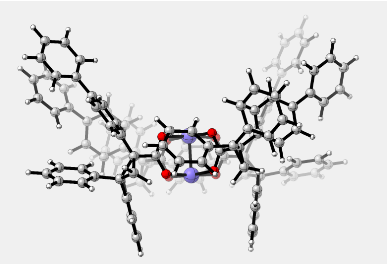

Rh_2(*S*-pPhTPCP)$_4$ (I)

Rh	2.60566900	15.96327400	4.96479300
Rh	2.77385700	16.91379800	2.77008700
O	1.73955400	15.26106000	2.06804200
O	1.44147000	14.46860400	4.16185900
O	4.46769000	15.79348200	2.46455400
O	0.89447600	17.10943000	5.23844400
O	4.33712200	14.90498800	4.53937200
O	1.07919500	18.00229300	3.17071200
O	3.77931800	17.50431400	5.65239800
O	3.81067400	18.48955500	3.62131700
C	2.02100900	13.07469300	0.16675700
H	2.93369600	13.24808200	0.72563400
C	4.63736000	19.70952000	5.49370800
C	4.86258800	15.01086200	3.38813200
C	0.81788600	24.37302300	3.36306700
C	-0.26102500	20.30037600	4.29527700
C	6.02632000	14.11121500	3.05034300
C	5.65908000	11.92725800	1.82649300
H	5.70400600	11.40261500	2.77498500
C	0.53738500	17.91010400	4.31954600
C	2.08213700	12.97521900	-1.21686800
H	3.04034200	13.08299800	-1.70944000
C	6.81494400	13.53622400	4.19408600
H	7.17090700	12.51770400	4.10156300
H	6.48856500	13.81277200	5.18923600
C	-0.07577100	20.71420000	2.96802900
H	-0.17247700	19.99071500	2.16861300
C	-2.04279800	18.37976600	4.32094200
C	5.57681100	13.23430900	-0.62438200
H	5.53533400	13.75763900	-1.57443200
C	5.81355200	13.31659100	1.79143000
C	0.79467500	12.98279200	0.84290400
C	1.80319300	25.01701000	4.12892400
H	2.26106100	24.48977600	4.95784900
C	-0.59658000	18.86680600	4.60171000
C	5.45209600	11.20041800	0.65401600
H	5.35308900	10.12111200	0.70655300
C	5.39997200	11.84046500	-0.59232200
C	2.21281300	10.06819200	1.84399300
H	3.09229900	10.69064600	1.95244200
C	-1.53030800	18.52320400	5.72907300
H	-1.31692500	17.60719300	6.26600000
H	-1.89674100	19.33447700	6.34569400
C	-3.00222600	19.40988900	3.77413600
C	4.93259300	23.28255400	3.05876800
C	5.77425400	13.95885300	0.54505800
H	5.89030300	15.03409000	0.49891900
C	0.92408500	12.75992000	-1.98535800
C	4.79015300	20.89940500	4.59462000
C	6.03725900	22.82804700	3.79211600
H	6.96617600	23.38931200	3.77064600
C	1.36272800	14.36579900	2.89495100

C	-0.38083300	12.52936100	3.15095500
H	-0.65074600	13.09078000	4.03622700
H	-1.21960900	12.12085900	2.60375800
C	0.76807200	13.10398100	2.33721600
C	0.28627600	25.04541300	2.25090700
H	-0.48523100	24.56582700	1.65598100
C	4.02984700	18.48336600	4.87780000
C	-0.13412000	21.25405500	5.31021500
H	-0.29634900	20.96735300	6.34391900
C	0.90529500	11.77933800	3.16814600
C	7.45724100	14.61738200	3.36526400
C	5.63281400	19.43099600	6.61114800
H	5.80803700	18.38392000	6.82343100
H	6.51554800	20.05393100	6.66064400
C	0.24339500	22.03357400	2.67084000
H	0.40286700	22.32527200	1.63744200
C	8.53237800	14.24439400	2.37263600
C	3.68781700	21.34518500	3.84829700
H	2.75523800	20.79423600	3.87948900
C	0.40158100	22.98912000	3.68945800
C	3.19359100	22.27784200	7.05038100
H	2.39037900	21.86889300	6.44988000
C	0.99786600	12.66739100	-3.46281800
C	8.81313800	15.10218500	1.29860900
H	8.27004800	16.03618600	1.20452800
C	0.98795000	10.49556200	2.37520900
C	-0.29498300	12.63779100	-1.30321100
H	-1.20570700	12.43518000	-1.85746000
C	3.76518300	22.50132300	3.08418900
H	2.89030300	22.83565900	2.54340000
C	0.72711900	26.32542500	1.91678600
H	0.30326300	26.83215900	1.05487000
C	4.28610200	21.47048400	7.39461600
C	5.96727100	21.65241800	4.53921700
H	6.84317100	21.33482400	5.09297800
C	2.24771900	26.29331700	3.79042600
H	3.03369700	26.76090500	4.37421800
C	4.96314300	24.56136800	2.31358800
C	4.00467300	9.27874500	-3.01045200
H	3.28243500	8.46818500	-3.00009800
C	0.19756000	22.57696500	5.01335400
H	0.28256100	23.30020700	5.81814300
C	4.32064300	20.01180300	7.00413700
C	9.26277700	13.05676600	2.48451800
H	9.08134500	12.38540100	3.31634200
C	-3.75208800	20.24173300	4.61246900
H	-3.67551300	20.13785200	5.68890300
C	5.58343500	10.66740200	-4.19551000
H	6.11206600	10.92751300	-5.10749800
C	3.13892200	23.60857000	7.45970200
H	2.28341400	24.22075900	7.19125500
C	-3.14846700	19.55574000	2.38639900
H	-2.58952800	18.90934300	1.71892800
C	4.68057200	9.60161500	-4.18933200
H	4.49774500	9.03556000	-5.09723700
C	9.78010700	14.77084200	0.35229900
H	9.97529400	15.44579300	-0.47565500
C	4.26696100	24.70960200	1.10331700
H	3.73448800	23.86073900	0.68627900
C	-0.13238600	9.67314400	2.21775100
H	-1.08545300	9.97611300	2.63813300
C	2.31208300	8.84782200	1.17916700
H	3.27260200	8.52090800	0.79299800
C	4.17841200	24.15780300	8.21607000
H	4.13558400	25.19604100	8.53051600
C	5.26740800	23.36027800	8.56651500
H	6.07869800	23.77311500	9.15857300
C	-0.06840700	13.09963500	-4.27029600
H	-0.95050600	13.53106500	-3.80778600
C	5.81875400	11.39630400	-3.02962100
H	6.54002700	12.20748400	-3.03778400
C	-0.35671500	12.75167200	0.08561400
H	-1.31863700	12.64964000	0.57517600
C	2.14178700	12.14909600	-4.09351000
H	2.96927400	11.78958200	-3.49548000
C	4.23611500	10.00994800	-1.84674800
H	3.67232400	9.78442400	-0.94953000
C	-4.61165500	21.20642000	4.08180300
H	-5.18457400	21.84019000	4.75208400
C	1.18465300	8.03766100	1.01690000
H	1.26366700	7.08874300	0.49553400
C	10.23620000	12.72329100	1.53977600
H	10.79202200	11.79663300	1.64731500
C	1.71199300	26.95219800	2.68295300
H	2.07100500	27.93905800	2.40983700
C	5.65851200	25.67068600	2.82259800
H	6.17858200	25.58394200	3.77155900
C	4.25996300	25.92772700	0.42775600
H	3.71303300	26.02117100	-0.50565000
C	5.31676800	22.02451100	8.16015300
H	6.16272100	21.40721200	8.44553800
C	10.49516700	13.57567500	0.46697100
H	11.24890400	13.31572300	-0.26990800
C	5.15466100	11.07298300	-1.83512400
C	4.95372800	27.02360700	0.94517800
H	4.94620800	27.97360700	0.41951300
C	5.65509400	26.88895100	2.14510200
H	6.19113600	27.73703500	2.56096400
C	-4.00161700	20.51945300	1.85366300
H	-4.09251300	20.61985200	0.77624900
C	-4.73615100	21.35341300	2.70069100
H	-5.40117200	22.10538100	2.28722100
C	-0.03890800	8.45611900	1.53791800
H	-0.92186300	7.83407300	1.42605100
C	1.14590800	12.48905200	-6.27314700
H	1.20207600	12.41965200	-7.35527200
C	2.21416800	12.06035500	-5.48235300
H	3.10603700	11.64510100	-5.94140900
C	0.00406200	13.01013300	-5.65973700
H	-0.82918500	13.35614700	-6.26425800
C	-2.27272000	16.99626000	3.77318700
C	-3.19191100	16.15475800	4.41267800
C	-1.67910100	16.56811400	2.57855300
C	-3.53076700	14.92066100	3.85814200
H	-3.65199200	16.48110200	5.34087400
C	-2.01824100	15.33514300	2.02106800
H	-0.94602000	17.19617400	2.08695600
C	-2.95352600	14.51440100	2.65266500
H	-4.25013700	14.28127300	4.36085400
H	-1.54696000	15.01718000	1.09732100
H	-3.22837100	13.56004200	2.21437300
C	3.34326100	19.18997500	7.80037800
C	3.78844800	18.49954600	8.93276000
C	1.98035000	19.17413900	7.49054200

C	2.88900200	17.79728000	9.73661100
H	4.84527300	18.51279700	9.18247600
C	1.07808000	18.47927500	8.29299700
H	1.62965500	19.68360900	6.60142100
C	1.52839200	17.78695600	9.41976200
H	3.24929600	17.26487800	10.61202400
H	0.02551700	18.46965200	8.03310500
H	0.82524200	17.24606900	10.04599500
C	1.71471200	11.71586100	4.43571400
C	1.08999500	11.33051800	5.62661300
C	3.09333300	11.94682100	4.43923100
C	1.82822200	11.18595500	6.80241600
H	0.01986600	11.14498300	5.62960000
C	3.83547300	11.79949800	5.60912100
H	3.58292500	12.27419700	3.53031200
C	3.20589500	11.41827200	6.79646200
H	1.32934300	10.88748900	7.71980400
H	4.90185700	11.99598900	5.59613600
H	3.78353300	11.30298700	7.70871600
C	7.66151800	15.97718800	3.98026400
C	8.47242000	16.08991000	5.11726500
C	7.16615700	17.14380200	3.38285200
C	8.80493100	17.34259300	5.63201100
H	8.85567600	15.18802600	5.58575700
C	7.49699200	18.39821100	3.89718200
H	6.51606600	17.07154000	2.51942300
C	8.32818700	18.50062600	5.01312000
H	9.44135500	17.41533600	6.50878200
H	7.09976300	19.29314300	3.42992400
H	8.59850200	19.47617900	5.40504600

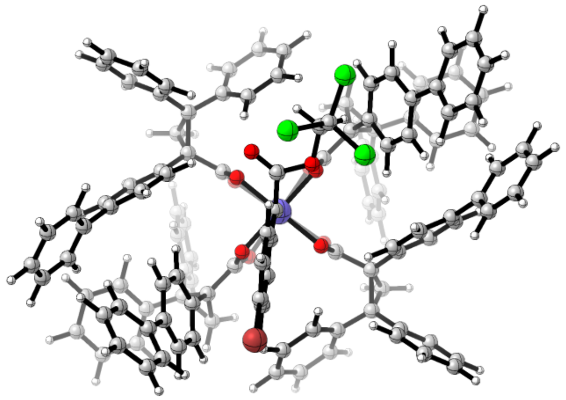

Intermediate II

Rh	2.62610300	15.92587800	5.08757800
Rh	2.81580500	16.84345100	2.81397400
O	1.88484100	15.10964500	2.20691600
O	1.45700900	14.39290800	4.30672900
O	4.56585900	15.81177600	2.65796800
O	0.91833500	17.10893400	5.27200700
O	4.36471800	14.82942400	4.68615600
O	1.03049400	17.82283100	3.12612600
O	3.80529500	17.50933800	5.69728100
O	3.74915800	18.50396600	3.66397600
C	1.97891700	13.11704100	0.24465900
H	2.91259000	13.24166600	0.77919200
C	4.62316200	19.72405000	5.50785600
C	4.91028100	14.97550500	3.55538500
C	0.74852800	24.32508800	3.16882900
C	-0.20474800	20.24064700	4.19993900
C	6.05508800	14.07720400	3.15106700
C	5.68756800	11.95868700	1.81018100
H	5.79078300	11.37723500	2.72044500
C	0.53696300	17.82711600	4.30428100
C	1.98918100	13.12935500	-1.14265000
H	2.93052800	13.27257600	-1.65723100
C	6.87900300	13.46504900	4.24875800
H	7.24631200	12.45597800	4.10567400
H	6.57492700	13.69810300	5.26234200
C	0.12022300	20.59632800	2.88124200
H	0.14931100	19.83018600	2.11715700
C	-2.02503300	18.35289600	4.26796000
C	5.45098800	13.41391000	-0.54456700
H	5.34806900	13.99460800	-1.45621200
C	5.79954600	13.35141800	1.85754900
C	0.77697200	12.98500000	0.95687200
C	1.52573300	25.11525600	4.03259800
H	1.90715700	24.68311500	4.95109700
C	-0.57565700	18.82336700	4.54255100
C	5.44378400	11.30051400	0.60332100
H	5.37368100	10.21758000	0.59070300
C	5.31637900	12.01599400	-0.59539800
C	2.25456600	10.03427200	1.81855600
H	3.13413300	10.65114200	1.95646000
C	-1.52156800	18.52634000	5.67454900
H	-1.31613700	17.62855400	6.24419400
H	-1.89543300	19.35898000	6.25757700
C	-2.99038800	19.37436600	3.71786000
C	5.06377700	23.30822400	3.09705800
C	5.67850300	14.07181600	0.65783500
H	5.74105600	15.15222500	0.67426300
C	0.80447200	12.97985300	-1.88463300
C	4.81373900	20.89850000	4.59614100
C	6.17771200	22.70733100	3.70431900
H	7.16177000	23.14929900	3.59696100
C	1.42530500	14.26229600	3.04860400
C	-0.33080900	12.42595400	3.27424600
H	-0.57602400	12.94954500	4.18966200
H	-1.18276500	12.03845600	2.73224800
C	0.79646200	13.03123400	2.45691600
C	0.32468800	24.88235600	1.95140900
H	-0.28762100	24.29073000	1.27789800
C	4.01203600	18.48571500	4.91606100
C	-0.20566100	21.24610200	5.17269200
H	-0.45378000	21.00350000	6.20021600
C	0.95684800	11.67720000	3.22910100
C	7.48896100	14.58017000	3.43932900
C	5.59494100	19.48092700	6.65066900
H	5.76196100	18.44193900	6.90441100
H	6.47817200	20.10359800	6.69197600
C	0.41829900	21.91277500	2.54987600
H	0.68433300	22.15606500	1.52595100
C	8.54830800	14.23974400	2.41857600
C	3.70735200	21.46432300	3.94657700
H	2.73459200	20.99405700	4.02935200
C	0.41170500	22.92669400	3.52355600
C	3.06359300	22.29201000	7.04809000
H	2.22752400	21.81986200	6.54713500
C	0.83078200	12.99156100	-3.36593600
C	8.71874700	15.04919000	1.28540500
H	8.08627500	15.91867000	1.14717100

C	1.03232600	10.43112500	2.37907400
C	-0.39011200	12.80948400	-1.17016900
H	-1.32180600	12.65090500	-1.70326200
C	3.83162900	22.63962700	3.21677600
H	2.94746700	23.06171300	2.76444600
C	0.65601200	26.19474100	1.61512600
H	0.31250100	26.61130000	0.67302400
C	4.21522900	21.54612000	7.33747100
C	6.05038100	21.53306100	4.44485300
H	6.93306900	21.11936600	4.91753600
C	1.85754500	26.42524500	3.69507900
H	2.47354800	27.01356500	4.36824000
C	5.17354200	24.59631100	2.36881400
C	3.84190400	9.60891800	-3.12350900
H	3.12240600	8.79595400	-3.14070500
C	0.09955800	22.56641400	4.84173500
H	0.07025200	23.32891300	5.61331500
C	4.27440200	20.07394000	7.00166100
C	9.38802600	13.13323400	2.58074300
H	9.28757500	12.50528200	3.45951400
C	-3.92348600	20.00340500	4.54839700
H	-3.95352600	19.75699700	5.60459700
C	5.37420700	11.07904200	-4.27091100
H	5.86888300	11.40089000	-5.18230200
C	2.98463700	23.64021500	7.38911400
H	2.08083700	24.19950900	7.16658200
C	-2.98632800	19.69289600	2.35223400
H	-2.27745200	19.20941100	1.68987700
C	4.47391700	10.01158100	-4.30230800
H	4.25869200	9.50615900	-5.23846700
C	9.68827300	14.74683500	0.33203700
H	9.79743200	15.38090000	-0.54278400
C	4.20016700	24.99410200	1.43682800
H	3.34735300	24.35866000	1.23704300
C	-0.08868600	9.61813900	2.18375500
H	-1.03966100	9.89745700	2.62474300
C	2.35065800	8.85262300	1.08675500
H	3.30872400	8.54982300	0.67539700
C	4.05910800	24.27203000	8.02199700
H	3.99787700	25.32376300	8.28414800
C	5.20663700	23.53754800	8.31883500
H	6.04583100	24.01344600	8.81726600
C	-0.27741400	13.43107100	-4.10892800
H	-1.15831600	13.79668600	-3.59307200
C	5.64918600	11.73130900	-3.06864900
H	6.36608200	12.54625500	-3.04860400
C	-0.40102500	12.81682900	0.22497000
H	-1.34501600	12.68164100	0.73961300
C	1.97167600	12.56244700	-4.06636200
H	2.83140800	12.19316400	-3.52209300
C	4.11482400	10.26185000	-1.92272700
H	3.58713300	9.97371600	-1.02121200
C	-4.82315800	20.93969000	4.03326000
H	-5.54034300	21.41626100	4.69483700
C	1.22247600	8.05154000	0.88770100
H	1.29855000	7.13270900	0.31455100
C	10.36435200	12.82953900	1.62858200
H	11.00671200	11.96625800	1.77488000
C	1.42097300	26.97175800	2.48686100
H	1.68688800	27.98968100	2.22037800
C	6.25050600	25.47035200	2.60431900
H	6.99851500	25.21811300	3.34797300

C	4.30935400	26.20215100	0.75253000
H	3.53819800	26.48137700	0.04173800
C	5.28018000	22.18356600	7.98153300
H	6.17339300	21.61946200	8.22897000
C	10.51499300	13.63219000	0.49824600
H	11.27037800	13.39561500	-0.24488100
C	5.03048200	11.32620100	-1.87469600
C	5.39362400	27.04971300	0.98357000
H	5.47891200	27.99122300	0.44966300
C	6.36211700	26.67828000	1.91878100
H	7.20290000	27.33446800	2.12338900
C	-3.87906000	20.62936700	1.83553600
H	-3.85336400	20.86955800	0.77686400
C	-4.80103500	21.25943600	2.67585700
H	-5.49615100	21.99035700	2.27423000
C	0.00194400	8.43962000	1.43860300
H	-0.88115800	7.82351500	1.29843000
C	0.89363400	13.01455200	-6.18557900
H	0.91758300	13.02465900	-7.27107700
C	2.00244100	12.57399800	-5.45919200
H	2.89374100	12.22698200	-5.97300400
C	-0.24670700	13.44300600	-5.50249000
H	-1.11159300	13.79878500	-6.05456700
C	-2.25963600	16.96106600	3.74562500
C	-3.04797500	16.06482700	4.47644100
C	-1.78214500	16.57315300	2.48677800
C	-3.37081800	14.81191400	3.95285000
H	-3.41890900	16.35910700	5.45365700
C	-2.10528500	15.32394100	1.96088600
H	-1.14947000	17.24929200	1.92537700
C	-2.90900200	14.44374500	2.68807300
H	-3.98851300	14.12880200	4.52785300
H	-1.72602300	15.03108800	0.98834900
H	-3.17226700	13.47532900	2.27457100
C	3.29070300	19.26698200	7.80566800
C	3.72223900	18.65617800	8.98864800
C	1.93852800	19.19026900	7.45968500
C	2.82122200	17.97593200	9.80886300
H	4.77071700	18.71651900	9.26531100
C	1.03423100	18.51709500	8.27892500
H	1.59631800	19.63060800	6.53103700
C	1.47086200	17.90749200	9.45711600
H	3.17166200	17.50639600	10.72336900
H	-0.00925500	18.45982400	7.99120100
H	0.76539300	17.38386700	10.09533900
C	1.79875100	11.57681400	4.47242500
C	1.21364200	11.11202100	5.65539100
C	3.16894400	11.85180400	4.45731500
C	1.98356100	10.92871300	6.80485400
H	0.14972800	10.89403800	5.67174400
C	3.94269300	11.66602500	5.60131200
H	3.62790500	12.23939800	3.55584900
C	3.35349500	11.20262500	6.77969400
H	1.51583300	10.56850500	7.71645100
H	5.00176700	11.89606800	5.57502600
H	3.95601300	11.05779000	7.67140900
C	7.69116000	15.93099300	4.07395200
C	8.43117100	16.03373800	5.25808800
C	7.25530000	17.10352200	3.44015600
C	8.75405300	17.28355000	5.78842900
H	8.76803000	15.12776800	5.75349900
C	7.58195400	18.35208400	3.96726400

H	6.65556100	17.04302100	2.54046900
C	8.34170200	18.44644500	5.13459100
H	9.33507400	17.34899100	6.70342000
H	7.23829900	19.25096900	3.46690100
H	8.60901900	19.41860200	5.53740600
C	3.02852500	17.51508400	0.92250300
C	2.16888400	17.35343000	-0.19004900
C	4.31914500	18.20264100	0.73008700
C	0.92573400	16.67016300	-0.05697000
C	2.51287100	17.88505400	-1.46888700
O	5.37175400	17.63547100	0.50969100
O	4.17277700	19.53554800	0.90453100
C	0.07158300	16.53434100	-1.13562500
H	0.66756900	16.23948600	0.89813300
C	1.65865100	17.75844700	-2.54612900
H	3.45406200	18.40651700	-1.60209800
C	5.34833300	20.33988800	0.94355600
C	0.43868000	17.08975800	-2.36716300
H	-0.86768700	16.00586700	-1.03675300
H	1.91743100	18.17169300	-3.51295000
H	5.25878800	20.99670500	1.80567300
H	6.24070100	19.71799500	1.01541300
C	5.43394900	21.19733500	-0.32226500
Br	-0.74918500	16.95493200	-3.83045900
Cl	6.80763900	22.33205600	-0.11103600
Cl	3.91159100	22.11816700	-0.56125700
Cl	5.72018600	20.16789500	-1.77459600

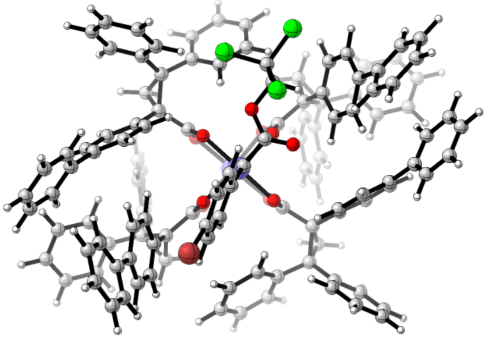

Intermediate III

Rh	2.57532000	15.90830600	5.10510400
Rh	2.74262100	16.88386500	2.85051700
O	1.76713900	15.16766000	2.20008800
O	1.41180700	14.40105700	4.29633100
O	4.47223700	15.81211300	2.62209700
O	0.86010800	17.09222000	5.33980500
O	4.30950400	14.82027600	4.65163400
O	1.00316500	17.88866600	3.22679400
O	3.74746800	17.47398600	5.77351500
O	3.73826900	18.47883100	3.74783800
C	1.86737400	13.12461200	0.21657300
H	2.79986000	13.35235000	0.71846300
C	4.60245900	19.68286100	5.61174200
C	4.82853100	14.96645400	3.50884600
C	1.07542500	24.21860700	3.21790100
C	-0.25083900	20.24944900	4.27521300
C	5.95912700	14.06576000	3.07333900
C	5.55127400	12.00402300	1.67062500
H	5.62904500	11.39860900	2.56718000
C	0.49210400	17.84731400	4.39565300
C	1.84984800	13.04122600	-1.16931300
H	2.76806800	13.23273400	-1.70811000
C	6.78015300	13.36779900	4.11976800
H	7.10908800	12.35598700	3.91743700
H	6.50617500	13.55582300	5.15094000
C	0.01989100	20.59510800	2.94245100
H	-0.05153200	19.83826000	2.17271300
C	-2.06958500	18.35624700	4.32176100
C	5.37988300	13.51536100	-0.65320200
H	5.29967600	14.11944600	-1.55169700
C	5.69967900	13.39182100	1.75211800
C	0.69371300	12.95283800	0.96656100
C	2.03213700	24.85490900	4.02597600
H	2.38317500	24.36089400	4.92428700
C	-0.62925400	18.83599500	4.62635400
C	5.31257900	11.37820500	0.44763600
H	5.22425200	10.29739700	0.41117300
C	5.21625900	12.12143200	-0.73666400
C	2.16030700	10.11352900	1.74343400
H	3.00674600	10.78883800	1.76842200
C	-1.59301400	18.53573900	5.73930900
H	-1.39721400	17.63735700	6.31194500
H	-1.97336700	19.36926000	6.31719100
C	-3.02033800	19.37499400	3.74007900
C	5.16145800	22.98544600	2.86409100
C	5.61588600	14.13907800	0.56675600
H	5.72791000	15.21466000	0.60913600
C	0.66897000	12.74810000	-1.87536000
C	4.82326300	20.83413900	4.67770100
C	6.21749500	22.56385800	3.68366500
H	7.18819700	23.04194300	3.60033000
C	1.35171500	14.29340000	3.03636000
C	-0.37786700	12.44378800	3.30770600
H	-0.61546300	12.98494200	4.21462700
H	-1.23324100	12.03302600	2.78892800
C	0.73409200	13.04730100	2.46335000
C	0.68711400	24.84347000	2.02203000
H	-0.05880800	24.37084400	1.39035700
C	3.97941800	18.45275500	5.00415900
C	-0.14649900	21.24738300	5.24972600
H	-0.37281900	21.01635300	6.28545900
C	0.91446000	11.70687100	3.25910700
C	7.40967900	14.51927000	3.37990100
C	5.56324500	19.41228800	6.75899100
H	5.70798400	18.36805500	7.00410100
H	6.45760600	20.01888000	6.81502100
C	0.41084500	21.88400500	2.60386900
H	0.64819300	22.11279400	1.56992100
C	8.42220200	14.26713400	2.29120100
C	3.75570700	21.28738100	3.89041100
H	2.78755200	20.80907100	3.96427700
C	0.55692500	22.87987300	3.58458600
C	3.14296900	22.30071200	7.09106900
H	2.33510700	21.87823600	6.50621000
C	0.64932200	12.69215300	-3.35757300
C	8.68970500	15.28196200	1.35983200
H	8.19326300	16.24104100	1.45677100
C	0.98375100	10.44869100	2.42723900
C	-0.49284800	12.53739300	-1.11720800
H	-1.42250100	12.26964800	-1.60686600
C	3.92718700	22.32999000	2.99248700
H	3.09060700	22.65171300	2.38894600
C	1.23836000	26.06837600	1.64636700

H	0.92413900	26.53737400	0.71879500
C	4.23222800	21.49706500	7.45381100
C	6.04910600	21.50164800	4.57214400
H	6.89345200	21.19226200	5.17749800
C	2.58538600	26.07687400	3.64856100
H	3.34087300	26.54030800	4.27560500
C	5.31923700	24.07697200	1.87866900
C	3.80878000	9.72369600	-3.31063300
H	3.11053400	8.89304200	-3.34415500
C	0.25468300	22.54154200	4.91107700
H	0.32466100	23.29828300	5.68598900
C	4.25293800	20.02892700	7.10185400
C	9.08716100	13.04451800	2.15552800
H	8.91052500	12.24869000	2.87044200
C	-3.87930100	20.12125800	4.55363400
H	-3.87885500	19.96303900	5.62651700
C	5.30739500	11.25005500	-4.42803500
H	5.79858400	11.59783400	-5.33174300
C	3.09446000	23.64155200	7.46706400
H	2.23991800	24.25008800	7.18708700
C	-3.06613400	19.58290400	2.35351300
H	-2.42129400	19.00313800	1.70290300
C	4.43664700	10.15953700	-4.47958400
H	4.23943700	9.66243000	-5.42404500
C	9.58273100	15.07294200	0.31180200
H	9.77098100	15.86989300	-0.40176600
C	4.70108200	23.98618800	0.62029100
H	4.14382300	23.08999900	0.36615100
C	-0.09998200	9.56693900	2.37210300
H	-1.01320400	9.80609100	2.90756000
C	2.24784400	8.92405200	1.02337100
H	3.17002600	8.67112200	0.50901400
C	4.13898800	24.20566600	8.20537800
H	4.10107200	25.25147100	8.49426800
C	5.22634900	23.41297400	8.57126000
H	6.04157700	23.83740300	9.14954000
C	-0.55037300	12.84773400	-4.07438800
H	-1.47875800	13.02653700	-3.54330900
C	5.55922500	11.89167300	-3.21491700
H	6.25704800	12.72238700	-3.17952900
C	-0.48078400	12.65000400	0.27167500
H	-1.40588100	12.48562400	0.81121000
C	1.83356300	12.50992500	-4.09215700
H	2.77412500	12.36207100	-3.57841700
C	4.05705000	10.36642500	-2.09961200
H	3.53009800	10.05176300	-1.20626000
C	-4.74971300	21.06429600	4.00162000
H	-5.40877900	21.63137100	4.65220000
C	1.15673500	8.05189900	0.96595900
H	1.22561000	7.12708800	0.40153600
C	9.98577600	12.83301200	1.10702900
H	10.49221900	11.87634900	1.02028800
C	2.19028100	26.68969600	2.45749500
H	2.63039900	27.63545300	2.15722500
C	6.06692800	25.22604900	2.17901800
H	6.53329900	25.31830800	3.15514000
C	4.81973300	25.01993400	-0.30673000
H	4.33915300	24.93079000	-1.27643800
C	5.26843000	22.06660100	8.20020200
H	6.11200700	21.45350800	8.50082500
C	10.23383600	13.84355900	0.17830900
H	10.92986500	13.67809100	-0.63828900

C	4.94508300	11.45350400	-2.03028200
C	5.55991700	26.16283400	0.00607100
H	5.65032300	26.96948200	-0.71498600
C	6.18577800	26.26014600	1.25108300
H	6.75903500	27.14710600	1.50406600
C	-3.92958700	20.52527700	1.79968200
H	-3.94197400	20.67623100	0.72440300
C	-4.77472600	21.27395000	2.62322700
H	-5.44773700	22.00918500	2.19288500
C	-0.01782500	8.37756000	1.64349900
H	-0.87072400	7.70624100	1.61149100
C	0.62126100	12.65234400	-6.18213000
H	0.61090100	12.64021300	-7.26778200
C	1.82001800	12.49276200	-5.48486800
H	2.75191200	12.34385700	-6.02137700
C	-0.56590500	12.82542700	-5.46703000
H	-1.50582800	12.95817000	-5.99458300
C	-2.28799600	16.96269400	3.79341700
C	-3.15904900	16.09840600	4.46762700
C	-1.72356700	16.54467700	2.57989400
C	-3.47892000	14.85048900	3.93156100
H	-3.59684600	16.41603800	5.40941800
C	-2.04488700	15.29973600	2.04046400
H	-1.02364900	17.19012400	2.06321500
C	-2.93171900	14.45448700	2.70942900
H	-4.16090200	14.19299700	4.46221700
H	-1.60053300	14.98764700	1.10138300
H	-3.19309300	13.49038000	2.28475000
C	3.24376000	19.23971600	7.89122000
C	3.64817200	18.59467300	9.06522100
C	1.89286900	19.21245400	7.53457700
C	2.71998500	17.92773200	9.86604600
H	4.69596100	18.61689300	9.35001000
C	0.96176100	18.55284300	8.33433200
H	1.57343800	19.68426300	6.61335500
C	1.37084900	17.90770500	9.50358000
H	3.04860500	17.43053200	10.77400800
H	-0.08096600	18.53374400	8.03884700
H	0.64491000	17.39422700	10.12690000
C	1.76497000	11.61897400	4.49734200
C	1.17522900	11.22127200	5.70235800
C	3.14553100	11.83121000	4.45701600
C	1.95030600	11.04377800	6.84914900
H	0.10332000	11.05058200	5.73845700
C	3.92435000	11.64898000	5.59813400
H	3.60748500	12.16748300	3.53682700
C	3.33055200	11.25442600	6.79904500
H	1.47847900	10.73579000	7.77763600
H	4.99280500	11.82559900	5.55258400
H	3.93788300	11.11234600	7.68792000
C	7.67040300	15.78227600	4.15715800
C	8.54848400	15.71693500	5.24731200
C	7.15579500	17.02693200	3.77273100
C	8.92386100	16.87132600	5.93285600
H	8.94561600	14.75161700	5.54748900
C	7.53373800	18.18146500	4.45940800
H	6.45592400	17.09265400	2.94801600
C	8.42269400	18.11134800	5.53212600
H	9.60862700	16.80387100	6.77278100
H	7.12235600	19.14009500	4.16796500
H	8.71614000	19.01446700	6.05815400
C	2.96811200	17.68782100	1.00307900

C	2.42277400	17.31069900	-0.25098500
C	3.82457600	18.88906900	1.01090700
C	1.26914100	16.48340500	-0.33696500
C	3.01252100	17.77769500	-1.46385700
O	3.32238500	19.99433500	0.96008500
O	5.14409900	18.63341300	1.13673400
C	0.72075800	16.15780700	-1.56556600
H	0.82414800	16.10995000	0.57328900
C	2.49222400	17.42193500	-2.69104100
H	3.89484400	18.40662600	-1.42767700
C	5.99721800	19.75333800	1.37212000
C	1.33964500	16.62276000	-2.73154000
H	-0.15945200	15.53132300	-1.62697900
H	2.95159200	17.76271600	-3.61052800
H	5.41699800	20.63265800	1.64711600
H	6.67453700	19.47642800	2.17673700
C	6.83862800	20.06190400	0.13290600
Br	0.61273000	16.16657500	-4.41438000
Cl	7.95500100	21.39799300	0.56920800
Cl	5.80064700	20.57362500	-1.24327500
Cl	7.78529700	18.60996700	-0.35460500

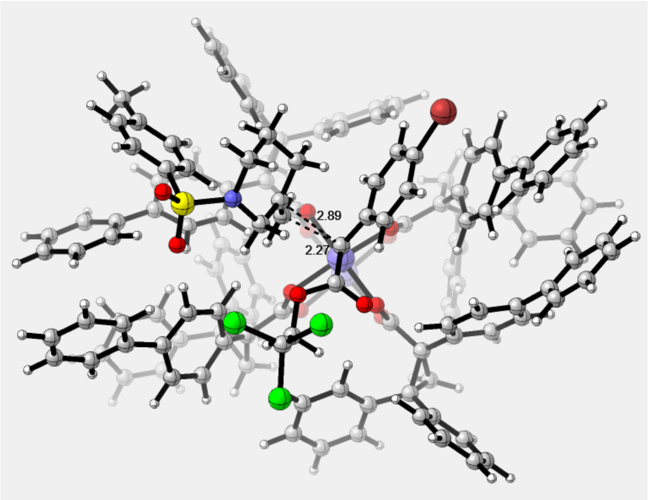

TS1

Rh	2.48257500	16.27894000	4.81820800
Rh	2.79743300	17.11527000	2.52732700
O	1.76048400	15.44242800	1.93055600
O	1.22519200	14.82141900	4.03813100
O	4.47429100	15.91042000	2.45219200
O	0.85851800	17.54775500	5.00322800
O	4.12007300	15.04956100	4.51519300
O	1.02843100	18.17904500	2.84063300
O	3.76753400	17.78137900	5.41335500
O	3.88160000	18.70678200	3.35739100
C	1.98219800	13.24137900	0.15708800
H	2.85390300	13.41857400	0.77528200
C	4.92020700	19.83157800	5.21516000
C	4.71859900	15.09474900	3.40102700
C	1.47654400	24.74765600	4.05994200
C	-0.10001500	20.70690000	4.10450200
C	5.80491700	14.07645900	3.13349500
C	5.36137300	11.92080100	1.87951600
H	5.29760100	11.39423000	2.82597000
C	0.51465000	18.24615500	4.00978800
C	2.15573700	12.99160000	-1.19393200
H	3.15686300	12.96480800	-1.59560000
C	6.45270900	13.42558700	4.32562800
H	6.71528200	12.37768300	4.24787300
H	6.08733600	13.73170600	5.29812400
C	1.16625300	21.01274300	3.58807500
H	1.81141300	20.22182000	3.24184700
C	-1.98566300	18.86865800	3.70672600
C	5.55266100	13.22831300	-0.56241100
H	5.60811000	13.75394400	-1.51105500
C	5.61233700	13.29667800	1.86167000
C	0.70151700	13.24844800	0.72974100
C	1.17378900	25.67403100	5.07116400
H	0.47778200	25.40051000	5.85786300
C	-0.57437900	19.27942700	4.20054800
C	5.19043000	11.20869100	0.69117600
H	5.00273800	10.14043400	0.73058700
C	5.27867600	11.84988900	-0.55190700
C	1.82460400	10.33846100	1.79317900
H	2.72551400	10.92969800	1.90026000
C	-1.67678000	18.93969900	5.17363700
H	-1.56747100	18.00529100	5.71063300
H	-2.09226000	19.74730400	5.76370600
C	-2.83367200	19.92215500	3.04702000
C	6.22104100	23.37433900	3.12452300
C	5.70663300	13.94134700	0.61998600
H	5.87406800	15.00815400	0.58325200
C	1.06671400	12.71211700	-2.03427500
C	5.51662500	20.89382900	4.33630100
C	7.00552200	22.81552900	4.14384700
H	7.87158400	23.35539900	4.51330900
C	1.23471200	14.64913100	2.78174000
C	-0.66210100	12.95135400	2.96211600
H	-0.94718500	13.55179400	3.81673200
H	-1.49023400	12.58299600	2.37158200
C	0.57148500	13.42621400	2.21349000
C	2.40853600	25.11837000	3.07234000
H	2.63331600	24.44006700	2.25761800
C	4.13489200	18.69360500	4.61234200
C	-0.86202500	21.78295000	4.59521800
H	-1.85738300	21.61646400	4.98703500
C	0.56702300	12.11715200	3.07705900
C	7.25167800	14.43779300	3.55005700
C	5.56941000	19.48647200	6.54434600
H	5.46322900	18.45190400	6.84063900
H	6.54047900	19.91069600	6.75838600
C	1.63932900	22.31691700	3.56115400
H	2.62785900	22.50382400	3.17069000
C	8.35700500	13.96608500	2.63694900
C	4.83062200	21.38320800	3.21434700
H	3.98367200	20.83094600	2.84451400
C	0.89528800	23.38696200	4.06966400
C	4.02480300	22.84782700	6.13554900
H	3.37322200	22.58094600	5.31587300
C	1.29688300	12.36969300	-3.45544600
C	8.73600000	14.75209800	1.53846700
H	8.23399800	15.69590100	1.35686500
C	0.61275900	10.81569300	2.31138700
C	-0.21526300	12.74822400	-1.46762800
H	-1.08736600	12.58313000	-2.09206200
C	5.17348500	22.59183900	2.61688600
H	4.57768500	22.96081100	1.78971400
C	3.04466500	26.35689100	3.11658300
H	3.77838300	26.60920600	2.35788300

C	4.62897400	21.83923500	6.89733500
C	6.66659400	21.59912100	4.72764200
H	7.27372200	21.24443100	5.55040800
C	1.79866100	26.92052900	5.10698300
H	1.56190800	27.61651800	5.90618200
C	6.41704800	24.76904300	2.66916400
C	3.69957400	9.55504200	-3.11646000
H	2.86293500	8.86569400	-3.17604300
C	-0.37313200	23.08862300	4.59028000
H	-1.00278600	23.88395100	4.97601600
C	4.37634500	20.37338800	6.62383900
C	9.02746000	12.75913900	2.85760600
H	8.76350500	12.14398700	3.71104900
C	-4.06651600	20.28921000	3.59551500
H	-4.38731600	19.84051000	4.53103300
C	5.53116900	10.72494700	-4.16480300
H	6.14140200	10.93101300	-5.03912400
C	4.29957800	24.19145300	6.37963600
H	3.84383000	24.95344900	5.75684100
C	-2.42581000	20.50412500	1.84001600
H	-1.45900700	20.24436700	1.42068600
C	4.47762200	9.81226500	-4.24624600
H	4.25580500	9.31721300	-5.18627900
C	9.74425900	14.33233200	0.67419300
H	10.01607300	14.95135900	-0.17560400
C	6.18031200	25.15099400	1.33896800
H	5.89317400	24.40624500	0.60796300
C	-0.53715700	10.03586300	2.15297000
H	-1.48064400	10.38257000	2.56186300
C	1.88074500	9.11426200	1.13033100
H	2.82884600	8.75432900	0.74258400
C	5.17101800	24.54972300	7.41181200
H	5.39143800	25.59615000	7.60120800
C	5.75622800	23.55493900	8.19726000
H	6.42987700	23.82341200	9.00577000
C	0.42721700	11.50777800	-4.14306900
H	-0.41749800	11.07115400	-3.61967600
C	5.80378500	11.37729000	-2.96318700
H	6.63093600	12.07825700	-2.90304900
C	-0.39162500	13.01478200	-0.10830600
H	-1.39704100	13.03243500	0.29654600
C	2.40455600	12.88491400	-4.14919700
H	3.08427400	13.56258800	-3.64529800
C	3.97383700	10.20358800	-1.91418500
H	3.33478100	10.03763200	-1.05443600
C	-4.87935400	21.22387100	2.95097100
H	-5.83231900	21.50369700	3.38970000
C	0.72397400	8.34663500	0.96638900
H	0.76883500	7.39474000	0.44624400
C	10.04214900	12.33675300	1.99496600
H	10.55113200	11.39647100	2.18487300
C	2.74487000	27.26237900	4.13755500
H	3.24599500	28.22466500	4.17736300
C	6.76483700	25.76371700	3.59948800
H	6.90415500	25.49138700	4.64095000
C	6.29012900	26.48584300	0.95143100
H	6.10151700	26.75984100	-0.08245700
C	5.48714900	22.20914400	7.93857400
H	5.95758000	21.43794400	8.54144600
C	10.40129200	13.11903200	0.89778900
H	11.18703400	12.79021800	0.22453800
C	5.03139500	11.12265800	-1.81868800
C	6.63844000	27.46583200	1.88455100
H	6.71847100	28.50565200	1.58249300
C	6.87551400	27.09794000	3.21107800
H	7.13217600	27.85302200	3.94823000
C	-3.23902600	21.43096200	1.18949000
H	-2.90474400	21.88098600	0.25995300
C	-4.46926000	21.79464600	1.74424700
H	-5.10095200	22.51974300	1.24050200
C	-0.48547900	8.81150900	1.48178200
H	-1.39034500	8.22206100	1.36784300
C	1.75127400	11.70900400	-6.15874200
H	1.92625100	11.45461800	-7.19970700
C	2.63027200	12.55799400	-5.48295600
H	3.49385100	12.96920100	-5.99676600
C	0.64867400	11.18423100	-5.48112100
H	-0.03546100	10.51277200	-5.99177900
C	-2.22590700	17.50036100	3.11580800
C	-3.00293200	16.57309300	3.82122300
C	-1.80031500	17.17639100	1.82071800
C	-3.36659400	15.35644500	3.24234600
H	-3.34430100	16.81683600	4.82204600
C	-2.15552800	15.95800900	1.24453000
H	-1.20015400	17.88530600	1.26320800
C	-2.95074400	15.05083500	1.94701000
H	-3.98015100	14.65520600	3.79956700
H	-1.81950100	15.71005400	0.24499900
H	-3.24406300	14.11304500	1.48659000
C	3.08790600	19.87694600	7.22756400
C	3.09091100	18.86012400	8.19027000
C	1.86840300	20.48818500	6.90766200
C	1.90681000	18.45123200	8.80462700
H	4.02646100	18.38481100	8.46402900
C	0.68408700	20.08304900	7.52009000
H	1.83814500	21.28600000	6.17795400
C	0.69566100	19.06160200	8.47107900
H	1.93359100	17.66301000	9.55160100
H	-0.24635900	20.57020200	7.24928100
H	-0.22670700	18.74811800	8.95088000
C	1.29465000	12.03112500	4.39127300
C	0.57740300	11.72186900	5.55209400
C	2.68436100	12.16208100	4.46907400
C	1.23565700	11.55255600	6.77121300
H	-0.50197700	11.61460900	5.49664500
C	3.34631000	11.98889900	5.68289100
H	3.24771200	12.43144100	3.58397600
C	2.62500600	11.68292500	6.83937300
H	0.66553200	11.31390000	7.66430500
H	4.42345800	12.10387000	5.72713800
H	3.14094900	11.54745400	7.78523100
C	7.54763900	15.76729300	4.19058000
C	8.24470400	15.79716000	5.40487900
C	7.25490600	16.97588900	3.54540300
C	8.66573100	17.00842500	5.95398700
H	8.47014400	14.86179600	5.90870000
C	7.68417600	18.18654900	4.08866100
H	6.68098800	16.95945500	2.62660300
C	8.39794500	18.20584300	5.28764400
H	9.21028600	17.01635900	6.89336700
H	7.45862800	19.11900100	3.58575500
H	8.73883900	19.14924200	5.70222100
C	3.14057100	17.71678700	0.47750100
C	4.58437200	18.00487100	0.31627600

C	2.45502900	17.09392300	-0.63992700
C	2.13787200	19.75135500	0.63050100
O	4.99143100	19.27327300	0.59418400
O	5.36642400	17.10364700	0.06867700
C	1.10361200	16.70615400	-0.49641200
C	3.06272000	16.92144900	-1.90855400
H	1.26031400	19.28823800	1.05858900
H	2.89143200	20.10264600	1.31979500
C	2.14101500	20.18097700	-0.66044600
C	6.38439900	19.44326200	0.84267800
C	0.38085500	16.21219500	-1.57035700
H	0.62564900	16.82047200	0.46500000
C	2.35228000	16.40121900	-2.98353600
H	4.10047500	17.19727200	-2.05096900
C	1.06311900	19.82870600	-1.63547500
C	3.25708400	21.02204300	-1.21608600
H	6.46541700	20.08455600	1.72006800
H	6.85431900	18.47707000	1.01551100
C	7.12188900	20.12539200	-0.31671000
C	1.00540900	16.07489700	-2.80983100
H	-0.65922900	15.93854800	-1.46122500
H	2.82539000	16.28062600	-3.95035500
C	0.56160200	21.06428200	-2.41414800
H	1.49759100	19.11499700	-2.35324000
H	0.23419700	19.32520000	-1.13534600
H	3.85782900	20.42525100	-1.91723900
H	3.92057300	21.37701800	-0.43619600
N	2.73704900	22.17546900	-1.96835600
Cl	6.89889200	19.22813100	-1.85703900
Cl	8.86728900	20.13204800	0.13625000
Cl	6.56384900	21.81282000	-0.53949700
Br	-0.02354200	15.49951100	-4.29792600
C	1.73007000	21.86383000	-2.99492400
H	-0.02393600	21.70184900	-1.74555600
H	-0.09883500	20.74143000	-3.22424000
S	2.78940900	23.70596600	-1.31584800
H	2.23698300	21.28541500	-3.77694900
H	1.39349200	22.79784700	-3.44751000
O	3.85556400	23.67127900	-0.29942000
O	2.88671800	24.65115400	-2.43600500
C	1.25057600	24.04147300	-0.47781000
C	0.61411600	25.26464400	-0.68934000
C	0.75505800	23.12385400	0.44965100
C	-0.52347800	25.57048700	0.05677900
H	1.01070100	25.96125000	-1.41809200
C	-0.39285400	23.43488200	1.16637800
H	1.25490300	22.18008500	0.62287700
C	-1.03588700	24.67021700	1.00032200
H	-1.01598100	26.52663900	-0.09310000
H	-0.77895400	22.72215800	1.88305100
C	-2.21866400	25.02636600	1.86100200
H	-1.87494200	25.36203300	2.84699800
H	-2.86702500	24.16120000	2.02247900
H	-2.80925600	25.83383700	1.42111500

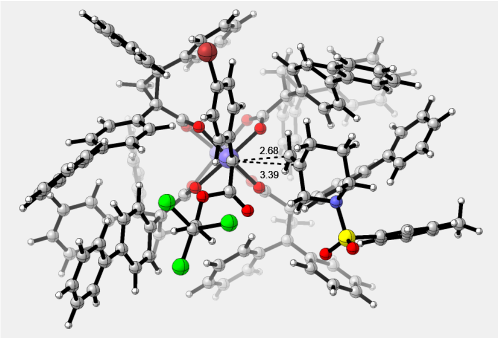

TS2

Rh	2.89210700	15.93871400	4.88297800
Rh	2.88970600	17.03591900	2.68038500
O	2.12305500	15.27028500	1.94638500
O	1.84205800	14.36701400	4.00345100
O	4.73396900	16.17810100	2.32746300
O	1.08640000	16.95209500	5.27352800
O	4.70343200	15.07757500	4.30598800
O	1.02582900	17.73603300	3.15587200
O	3.93315400	17.57024000	5.59414700
O	3.68433800	18.71634500	3.65853100
C	2.13719600	12.97129000	-0.28155400
H	3.07854700	12.61894500	0.12389400
C	4.55494000	19.85138800	5.56541100
C	5.19325700	15.33443300	3.16697200
C	0.28971600	24.23791500	3.37078100
C	-0.24044400	20.05633400	4.33578200
C	6.41407600	14.55388500	2.71489800
C	6.08357500	12.40439600	1.40136100
H	6.22245400	11.83067900	2.31131000
C	0.60416700	17.67621400	4.35869300
C	1.99858000	13.13837600	-1.65355600
H	2.84145300	12.92817500	-2.29842500
C	7.31655000	14.00123700	3.78070200
H	7.75285400	13.02412700	3.61353400
H	7.03157500	14.19973500	4.80695900
C	0.15985400	20.46063000	3.05148800
H	0.33023600	19.71354000	2.29054900
C	-1.98768600	18.07664300	4.34483800
C	5.73366900	13.83991300	-0.95036000
H	5.57177300	14.42640200	-1.84548400
C	6.19074100	13.79952600	1.43382900
C	1.07535800	13.25841900	0.58053900
C	0.78374700	25.15091900	4.31880200
H	1.08618400	24.79348500	5.29756000
C	-0.54500400	18.61220500	4.64774900
C	5.78297900	11.73712600	0.21363700
H	5.70346900	10.65572700	0.22388600
C	5.57152800	12.44537000	-0.98115300
C	2.94038100	9.80412600	1.64207900
H	3.72249700	10.03562200	2.35386600
C	-1.49193700	18.27130200	5.75633400
H	-1.25574800	17.40121100	6.35322400
H	-1.92755200	19.08600400	6.32129900
C	-2.96328800	19.09640300	3.81516100
C	4.77695400	23.71463800	3.60452700
C	6.05020700	14.49876100	0.23018300
H	6.15911300	15.57494300	0.23275600
C	0.79590700	13.60444600	-2.20954800
C	4.66469200	21.11614200	4.76596600

C	5.93655500	23.07042600	4.06256100
H	6.90207200	23.55486300	3.96895900
C	1.77319900	14.32662000	2.74181000
C	0.21685100	12.28546600	2.83783300
H	-0.00400400	12.63975900	3.83523000
H	-0.63393900	11.90034300	2.28949500
C	1.22956700	13.10018600	2.06386700
C	-0.03077400	24.71574200	2.08886900
H	-0.42944200	24.03107100	1.34712400
C	4.01545800	18.62178200	4.89028700
C	-0.40912300	21.04954700	5.30808500
H	-0.70092500	20.77812400	6.31630100
C	1.56981900	11.67746600	2.64316200
C	7.80621900	15.17574600	2.97103500
C	5.55952300	19.58376600	6.67485800
H	5.79886800	18.54156900	6.84284400
H	6.39979600	20.25991200	6.75586400
C	0.33700700	21.80320000	2.74422300
H	0.66172300	22.07387900	1.74521800
C	8.85757600	14.95520900	1.90882800
C	3.51474200	21.72420100	4.24451600
H	2.56129000	21.21395000	4.30469500
C	0.12704400	22.80482100	3.70778800
C	2.86183900	22.18927800	7.30510400
H	2.05156800	21.70957400	6.76981200
C	0.67212900	13.77142300	-3.67595000
C	9.01396600	15.89094100	0.87474500
H	8.38343800	16.77192500	0.84485300
C	1.75191400	10.55070600	1.64979800
C	-0.26007100	13.91367500	-1.33670500
H	-1.20622900	14.26490800	-1.73665900
C	3.56958500	22.99685800	3.68700500
H	2.65423200	23.45239700	3.34141100
C	0.12398100	26.06475500	1.77036200
H	-0.13744600	26.41528000	0.77653700
C	4.05562300	21.49133900	7.54094600
C	5.87495800	21.80543500	4.64232100
H	6.78766600	21.36283600	5.01909600
C	0.93641600	26.49809400	4.00051200
H	1.33255600	27.18371500	4.74311400
C	4.82394100	25.09319000	3.05913800
C	3.93391000	9.96005800	-3.33079900
H	3.35053800	9.04716800	-3.25502300
C	-0.23065300	22.39783800	5.00106600
H	-0.40408900	23.14182500	5.77153400
C	4.20782600	20.05677000	7.08805300
C	9.69061100	13.83113000	1.91729400
H	9.60548900	13.10009300	2.71337700
C	-4.05245300	19.50228800	4.59269900
H	-4.17946800	19.08989000	5.58903500
C	4.92924500	11.71452700	-4.65216100
H	5.14560200	12.16780700	-5.61459200
C	2.70904900	23.50555500	7.73361200
H	1.77756400	24.02899300	7.53841400
C	-2.82079700	19.63325700	2.52710100
H	-1.98150500	19.33253300	1.91191800
C	4.20275700	10.52326100	-4.58104500
H	3.84233000	10.04611800	-5.48722700
C	9.96639000	15.70338900	-0.12443200
H	10.04463200	16.43048400	-0.92528900
C	3.82859600	25.56941500	2.18960600
H	3.00346000	24.92941200	1.90624300
C	0.76165700	10.21010200	0.71444100
H	-0.16609900	10.76698200	0.67245800
C	3.13123500	8.75801300	0.73827100
H	4.05993700	8.19542800	0.76213100
C	3.74648300	24.15020100	8.41329400
H	3.62851900	25.17732400	8.74470700
C	4.93242500	23.46097200	8.66509500
H	5.74347900	23.94695500	9.19916600
C	-0.09513700	14.81055000	-4.22855000
H	-0.61468900	15.50090100	-3.57096600
C	5.38655700	12.33141200	-3.48909400
H	5.95132300	13.25019100	-3.56724400
C	-0.12103800	13.73910000	0.03934700
H	-0.94914500	13.97316800	0.70074100
C	1.35887800	12.91173800	-4.54993300
H	1.94225900	12.09090400	-4.14776100
C	4.39579500	10.57443300	-2.16833200
H	4.13828100	10.14578000	-1.20756600
C	-4.97543500	20.42806100	4.10096400
H	-5.81391500	20.73291600	4.71991500
C	2.13939800	8.43603100	-0.18874400
H	2.28716400	7.62375500	-0.89373500
C	10.64385700	13.63654000	0.91430600
H	11.27800700	12.75536500	0.94175800
C	0.60249300	26.96220600	2.72662100
H	0.72684600	28.01118700	2.47776700
C	5.86839200	25.96946500	3.40600900
H	6.62982800	25.64921100	4.10900500
C	3.88865800	26.85943000	1.66716000
H	3.10508400	27.19669600	0.99647000
C	5.08288500	22.14100100	8.23221200
H	6.00794600	21.61285600	8.43887900
C	10.78434600	14.57005700	-0.11185600
H	11.51955200	14.41738700	-0.89588900
C	5.13580100	11.76768200	-2.22549600
C	4.94131500	27.71097300	2.00535300
H	4.98793200	28.71589400	1.59697500
C	5.92915100	27.25982800	2.88373600
H	6.74501500	27.91645900	3.17104500
C	-3.74078900	20.55474400	2.03083200
H	-3.61057300	20.95337300	1.02953600
C	-4.82139800	20.95812400	2.81904400
H	-5.53706700	21.67952600	2.43667300
C	0.95358600	9.17241100	-0.19598100
H	0.17001600	8.93824800	-0.91020500
C	0.53669700	14.13813600	-6.46667600
H	0.48850200	14.28373600	-7.54149000
C	1.29435900	13.09475600	-5.92962800
H	1.83704900	12.41927600	-6.58442600
C	-0.16087500	14.99299500	-5.60926300
H	-0.74885900	15.81054400	-6.01566600
C	-2.18147800	16.67369000	3.81579700
C	-1.87118400	15.54283000	4.58788600
C	-2.75373800	16.45914000	2.55069700
C	-2.14799600	14.25656000	4.12660800
H	-1.42850900	15.65076900	5.56945700
C	-3.01204100	15.17253300	2.07945600
H	-3.03573000	17.30451800	1.93776800
C	-2.71962700	14.06053800	2.86976200
H	-1.93018800	13.40712400	4.76560200
H	-3.46419800	15.04383000	1.10034500
H	-2.94250800	13.05917700	2.51576100

C	3.29292900	19.12449500	7.83599000
C	3.79571800	18.41539600	8.93311300
C	1.93071500	19.02741800	7.53630700
C	2.95634400	17.62014200	9.71399000
H	4.85160400	18.49131600	9.17510900
C	1.08697100	18.24321000	8.32082600
H	1.53046300	19.54584300	6.67310900
C	1.59483100	17.53552400	9.41207300
H	3.36269500	17.07584300	10.56146700
H	0.03328500	18.17894300	8.07457200
H	0.93592100	16.92527400	10.02248300
C	2.43488900	11.55908300	3.86824800
C	1.94945100	10.86809500	4.98376000
C	3.73755900	12.05936200	3.89650600
C	2.75289700	10.68452700	6.10999100
H	0.93728800	10.47424300	4.96670400
C	4.54674500	11.87634300	5.01654000
H	4.11062300	12.60542900	3.04004400
C	4.05674200	11.18719900	6.12794100
H	2.36361600	10.14747100	6.96989300
H	5.55119000	12.28355400	5.02440400
H	4.68496000	11.04375600	7.00201300
C	7.90493200	16.51797300	3.64660500
C	8.68648400	16.64363500	4.80193500
C	7.32183300	17.66575800	3.09132000
C	8.90385500	17.89330900	5.38183000
H	9.13620600	15.75542900	5.23607600
C	7.54301000	18.91611500	3.66813400
H	6.68799900	17.58642800	2.21631800
C	8.34180800	19.03485400	4.80668000
H	9.51785000	17.97728500	6.27344700
H	7.08571600	19.79576600	3.22853800
H	8.52960100	20.00903000	5.24753600
C	2.76957900	18.08665400	0.95127100
C	3.97012800	18.93764700	0.75284300
C	1.54804300	18.32615500	0.24878800
C	3.37991000	16.15257800	-0.79309100
O	3.69343600	20.23310400	1.06514400
O	5.07557900	18.53765700	0.45430900
C	0.46293200	17.42379000	0.42058100
C	1.34614700	19.46613600	-0.57495600
H	2.41146700	15.70123200	-0.62970500
H	4.08191200	16.09621300	0.02517100
C	3.70996200	16.67120400	-1.98327600
C	4.77797200	21.15629100	1.03172100
C	-0.76763300	17.66658300	-0.16215500
H	0.61387900	16.52283300	0.99705800
C	0.10397700	19.73716100	-1.12437900
H	2.15679600	20.15858000	-0.74940500
C	2.80868500	16.62838200	-3.18074000
C	5.07544200	17.23528100	-2.27185300
H	5.72795400	20.63207900	0.93227000
H	4.74279200	21.73608000	1.95006400
C	4.60338500	22.10484800	-0.15676900
C	-0.94678800	18.83987700	-0.90150500
H	-1.58333600	16.96822300	-0.03028800
H	-0.05391000	20.62941900	-1.71749900
C	3.49705900	15.79438200	-4.27696400
H	2.64063900	17.64863600	-3.55730100
H	1.83834100	16.20112300	-2.92659500
H	4.98876900	18.28193800	-2.60446200
H	5.71250200	17.21256800	-1.39454700
N	5.69152800	16.40076700	-3.33651200
Cl	2.99373200	22.91059600	-0.08815900
Cl	5.90258000	23.33501300	-0.06185700
Cl	4.72603100	21.20433900	-1.71329300
Br	-2.66364900	19.22530900	-1.61040800
C	4.89718600	16.32736700	-4.58235100
H	3.57264300	14.75544600	-3.94385100
H	2.90336300	15.79917200	-5.19584600
S	7.34703200	16.66686300	-3.57988600
H	4.83919400	17.31771600	-5.05500200
H	5.40631900	15.65386400	-5.27533100
O	7.89286500	17.13866100	-2.29716000
O	7.57324600	17.46547900	-4.79873500
C	7.94021200	15.01634900	-3.87971300
C	7.94463700	14.50020600	-5.17792100
C	8.34460100	14.24283800	-2.79124700
C	8.32056600	13.17332600	-5.37180800
H	7.66757500	15.12819800	-6.01675700
C	8.72896300	12.92332300	-3.00791300
H	8.35141800	14.66285300	-1.79520300
C	8.69789500	12.36163500	-4.29159600
H	8.31378300	12.75887300	-6.37535600
H	9.03656000	12.31783200	-2.16088800
C	9.00812200	10.90366300	-4.50189400
H	8.07951500	10.32032500	-4.47379400
H	9.47672600	10.72761500	-5.47395000
H	9.66605300	10.51594300	-3.72006600

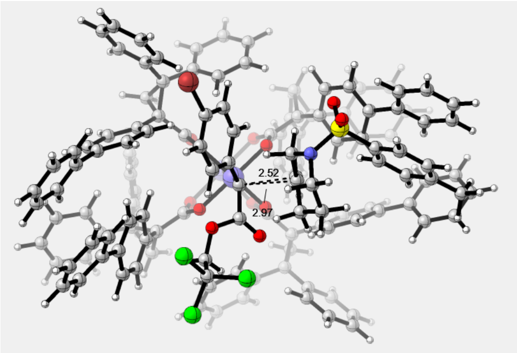

TS3

Rh	2.76700800	15.92466300	4.58221800
Rh	2.83130400	17.11252400	2.42651200
O	1.75352700	15.52640300	1.62270400
O	1.62514800	14.48236600	3.62618400
O	4.49204400	15.99403800	1.96384900
O	1.03880500	16.97093600	5.03566400
O	4.53974300	15.00443400	4.00276200
O	1.15534100	18.15067900	3.10868900
O	3.88521700	17.45734700	5.39475600
O	3.92983600	18.57286000	3.42800800
C	2.12647100	13.16168000	-0.51540000
H	3.06603600	13.15536900	0.02728800
C	4.66715200	19.70041100	5.38124100
C	4.98109400	15.19906600	2.83392700
C	0.40853400	24.46806100	3.78489200
C	-0.23565400	20.26015700	4.48843400
C	6.17747900	14.40423700	2.37182000
C	5.85003300	12.27780100	1.06296900
H	5.91893600	11.72276400	1.99220900
C	0.65960300	17.89266200	4.25656000
C	2.12774500	12.94690700	-1.88798500

H	3.06868300	12.78611700	-2.40053200
C	7.07666000	13.78682800	3.40686300
H	7.47888600	12.80191100	3.20429200
H	6.81513700	13.96480800	4.44313800
C	-0.16908600	20.77217200	3.18429100
H	-0.24753300	20.09584000	2.34620100
C	-1.91702500	18.25845000	4.58973000
C	5.70360700	13.67165300	-1.33663500
H	5.62640000	14.22750100	-2.26611600
C	5.94962800	13.67214300	1.07808000
C	0.92096800	13.32497000	0.18162000
C	1.35432100	25.16032000	4.55852600
H	1.91819700	24.62378500	5.31360600
C	-0.46477400	18.79381600	4.72221900
C	5.68952900	11.58543800	-0.13432300
H	5.64900500	10.50207000	-0.12180500
C	5.62786400	12.26754000	-1.35764400
C	2.62514300	10.12949900	1.20284700
H	3.44534600	10.44040700	1.83612000
C	-1.24428700	18.33473600	5.92854100
H	-0.92411500	17.40083800	6.37193500
H	-1.57233700	19.08843100	6.63371000
C	-2.98464500	19.26897600	4.24835900
C	4.51411600	23.51420000	3.31704900
C	5.86222400	14.36213400	-0.13924500
H	5.92779400	15.44318300	-0.13810300
C	0.92465500	12.89906400	-2.61615300
C	4.67695400	20.97259800	4.58803400
C	5.69616200	23.08848700	3.94263200
H	6.57311100	23.72678600	3.93609600
C	1.48069800	14.52022600	2.36956900
C	-0.14756200	12.54981700	2.43193800
H	-0.40858300	12.95519400	3.40143900
H	-0.99086600	12.18293200	1.86180300
C	0.93420100	13.30340700	1.68198900
C	-0.25953600	25.16037200	2.76234200
H	-1.00033800	24.64060400	2.16197600
C	4.11378600	18.48466900	4.69019100
C	-0.11870100	21.15574300	5.55563700
H	-0.18477800	20.79335700	6.57578400
C	1.18061400	11.87573500	2.32570700
C	7.59208600	14.97595200	2.63745600
C	5.74437800	19.40576000	6.41485000
H	5.99891200	18.35939200	6.52736400
H	6.58754800	20.08127100	6.47109700
C	0.00846600	22.13106800	2.95865300
H	0.07002600	22.49769000	1.93911500
C	8.59546000	14.79101900	1.52636400
C	3.50674900	21.38004100	3.92852700
H	2.63741000	20.73358900	3.91969600
C	0.14992800	23.03089000	4.02882300
C	3.18500400	22.05762500	7.16799300
H	2.38991300	21.65898000	6.55061800
C	0.92778400	12.52865300	-4.04968200
C	8.75406600	15.80968500	0.57411900
H	8.17634400	16.72316700	0.66865800
C	1.37000000	10.74209400	1.33774300
C	-0.26993800	13.15579500	-1.93048100
H	-1.21259900	13.13063200	-2.46813300
C	3.43357900	22.61565200	3.30228400
H	2.50358700	22.90486500	2.83536000
C	-0.00064700	26.51089000	2.53297100
H	-0.53161400	27.03389400	1.74310100
C	4.32037900	21.27981800	7.43151000
C	5.77330600	21.84186600	4.56559700
H	6.70334300	21.55922400	5.04540400
C	1.61487800	26.50875100	4.32628500
H	2.36892100	27.01997700	4.91594300
C	4.38558000	24.86311000	2.71828500
C	4.69090300	9.59191700	-3.87317300
H	4.08474900	8.69161000	-3.90723700
C	0.07803100	22.51829800	5.33062800
H	0.15670000	23.19334800	6.17663100
C	4.42212900	19.85984600	6.92521400
C	9.35565500	13.62549900	1.39061200
H	9.26207300	12.82933300	2.12073800
C	-3.78447600	19.84704100	5.23901600
H	-3.64620800	19.56213600	6.27658100
C	6.15108700	11.18078900	-4.95202700
H	6.70939800	11.50678300	-5.82310500
C	3.08070400	23.35248100	7.67161800
H	2.19413100	23.94270100	7.46102200
C	-3.19992700	19.63948000	2.91313700
H	-2.59550500	19.19626400	2.13066500
C	5.38985200	10.01105000	-5.00732700
H	5.34563400	9.43086300	-5.92371100
C	9.63432700	15.65732100	-0.49443700
H	9.73562300	16.45413700	-1.22530800
C	3.48003100	25.11135800	1.67246400
H	2.87121800	24.30287300	1.28490500
C	0.33175200	10.28640100	0.51176800
H	-0.65728300	10.71946800	0.58630100
C	2.83945000	9.11814500	0.26696400
H	3.81972400	8.65615600	0.19532600
C	4.11015500	23.89310400	8.44750300
H	4.02881600	24.90385800	8.83539800
C	5.24027500	23.12284300	8.72127700
H	6.04455500	23.52934700	9.32715200
C	0.09828500	13.17533400	-4.97794000
H	-0.53788300	13.98931300	-4.64954600
C	6.21252300	11.92686800	-3.77547800
H	6.82953100	12.81901100	-3.73482000
C	-0.26883900	13.35518300	-0.54996400
H	-1.21590600	13.46660900	-0.03354600
C	1.79027200	11.51985200	-4.50507600
H	2.42431300	11.00078500	-3.79666600
C	4.75364100	10.33521200	-2.69555000
H	4.18070300	10.02061200	-1.83041100
C	-4.76694900	20.78363900	4.90791800
H	-5.37854500	21.21997200	5.69198800
C	1.80428100	8.69796500	-0.56931800
H	1.97260300	7.91743500	-1.30490400
C	10.24171600	13.47124100	0.32163300
H	10.82272000	12.55793700	0.23456100
C	0.93595600	27.18978700	3.31524500
H	1.14759100	28.23734200	3.12667000
C	5.14667800	25.94055400	3.20109100
H	5.82379700	25.78805100	4.03521300
C	3.35485800	26.38142100	1.11780500
H	2.64608100	26.54580700	0.31232800
C	5.34111800	21.82382900	8.21731300
H	6.22080000	21.22741100	8.43852200
C	10.38137900	14.48359700	-0.62744300
H	11.06721600	14.36250500	-1.46034800

C	5.51974700	11.51118000	-2.62635600
C	4.12826400	27.43993300	1.59837500
H	4.02881900	28.43133400	1.16712000
C	5.02158300	27.21383000	2.64660400
H	5.61769500	28.03148800	3.04100600
C	-4.17345200	20.57742500	2.57924300
H	-4.30807100	20.85964500	1.54012100
C	-4.96155400	21.15592600	3.57796400
H	-5.72057900	21.88809700	3.31993000
C	0.54648800	9.28738400	-0.43714000
H	-0.27581400	8.96766600	-1.07000600
C	1.02381100	11.84029100	-6.77403000
H	1.07038800	11.58676100	-7.82880800
C	1.83937000	11.17763200	-5.85428800
H	2.51863600	10.39692900	-6.18345300
C	0.15197500	12.83622900	-6.32910500
H	-0.47361000	13.36767800	-7.03998800
C	-2.15450300	16.91131900	3.96595400
C	-2.92450000	15.95314400	4.63512400
C	-1.70593900	16.63529900	2.67058200
C	-3.25648600	14.75063800	4.00921400
H	-3.27296000	16.15957900	5.64284700
C	-2.04122100	15.43960900	2.03911100
H	-1.08609400	17.36105700	2.16452900
C	-2.82686100	14.49658200	2.70414800
H	-3.85805000	14.01605600	4.53595300
H	-1.68825300	15.25212200	1.03122700
H	-3.09897700	13.56722100	2.21411000
C	3.55676500	18.91421900	7.71367000
C	4.13525600	18.13134700	8.71839200
C	2.16983900	18.87425200	7.53901900
C	3.34556800	17.31348600	9.52813800
H	5.21062300	18.16401800	8.86530800
C	1.37714900	18.06622100	8.35144300
H	1.71315600	19.45794800	6.74895600
C	1.96098400	17.28060300	9.34849500
H	3.81017600	16.71061300	10.30292200
H	0.30331500	18.04489700	8.20427400
H	1.34170400	16.65209300	9.98139100
C	1.99629500	11.72409400	3.58012200
C	1.42202800	11.11445200	4.70043300
C	3.33908800	12.10730900	3.63462100
C	2.17535600	10.89616600	5.85538900
H	0.37939400	10.81198500	4.66571600
C	4.09787500	11.88819500	4.78222700
H	3.78301400	12.60092800	2.77946900
C	3.51789800	11.28077200	5.89848100
H	1.71593800	10.42263100	6.71805000
H	5.13375300	12.20726500	4.80830400
H	4.10659800	11.10928300	6.79472900
C	7.78572500	16.26191800	3.39728500
C	8.72657100	16.27328200	4.43644700
C	7.15637600	17.45944900	3.03636300
C	9.05669700	17.46046200	5.08776200
H	9.21149200	15.34341500	4.71934900
C	7.48471200	18.64741500	3.69317400
H	6.41508900	17.46277500	2.24656500
C	8.44255700	18.65539100	4.70684200
H	9.79403000	17.45435700	5.88485100
H	6.98530900	19.56961700	3.42307700
H	8.70211300	19.58452200	5.20463300
C	2.91208800	18.27522700	0.71953700
C	4.29749600	18.78930700	0.52716300
C	1.73892200	18.98809800	0.28423000
C	3.01668000	16.50768500	-1.07624300
O	4.38620200	20.09130500	0.92278700
O	5.26078400	18.13598300	0.18582900
C	0.49567300	18.30960600	0.33970100
C	1.74455800	20.32852500	-0.17441200
H	2.11899400	16.03082800	-0.70329600
H	3.95171600	16.15827500	-0.66167400
C	2.98312300	17.38449400	-2.10809700
C	5.68415400	20.62205600	1.15998000
C	-0.68488500	18.93542200	-0.02202300
H	0.48861000	17.27207900	0.64918400
C	0.55544000	20.98137900	-0.48366600
H	2.67418000	20.87464800	-0.24679000
C	1.66895300	17.88234300	-2.65007800
C	4.20211100	18.04844000	-2.68613900
H	6.41735700	19.82128400	1.24610700
H	5.62006600	21.19608100	2.08194900
C	6.13245500	21.56822100	0.04037200
C	-0.64903700	20.28291900	-0.39974100
H	-1.62508500	18.39807400	0.00060900
H	0.56740800	22.01821200	-0.79703500
H	1.46929800	18.86176300	-2.19032700
H	0.84345500	17.22434200	-2.38940500
H	4.25473800	19.06277500	-2.26447600
H	5.10952200	17.53343800	-2.36713400
Cl	4.89767800	22.83395700	-0.25199600
Cl	7.65899900	22.33842100	0.61143800
Cl	6.44940600	20.69472200	-1.49644700
Br	-2.27505000	21.17905800	-0.79656800
C	2.81710900	18.82889100	-4.64880700
H	2.75873900	19.86160200	-4.28265300
H	2.72118900	18.86554600	-5.73536900
N	1.66126800	18.10374300	-4.09948700
S	0.80046000	17.09670900	-5.10425300
O	0.29093600	17.91506900	-6.21455800
O	-0.15246400	16.37800400	-4.24202200
C	1.96282700	15.94447600	-5.80422300
C	2.68833700	15.09466400	-4.96304900
C	2.12108500	15.90156200	-7.18666600
C	3.57934000	14.19111200	-5.52634000
H	2.55306100	15.13053000	-3.88741100
C	3.01072100	14.97955600	-7.73501900
H	1.54935400	16.57374500	-7.81467500
C	3.74361700	14.11149400	-6.91960000
H	4.13735700	13.51740600	-4.88452200
H	3.13035800	14.92958900	-8.81312200
C	4.68105600	13.09796900	-7.51857700
H	4.42246700	12.09095000	-7.17785300
H	4.64255300	13.11592200	-8.61028000
H	5.71492300	13.28646800	-7.21082300
C	4.13750800	18.18715800	-4.21697100
H	4.97066800	18.80760500	-4.56194500
H	4.24077900	17.20586400	-4.68766000

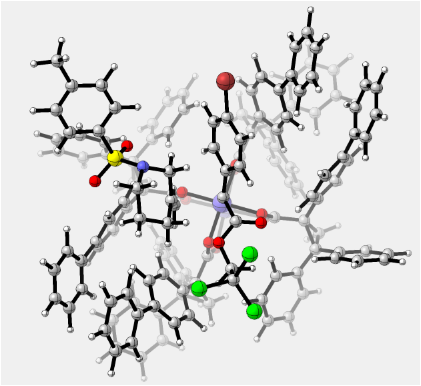

TS4

Rh	2.34017200	16.36086900	4.74656300
Rh	2.64573000	17.02266900	2.39351400
O	1.67309900	15.25596600	1.94352400
O	1.17697400	14.77162400	4.09450400
O	4.35710200	15.88103800	2.43434800
O	0.63873600	17.54765300	4.76840900
O	4.05445300	15.19727500	4.57046900
O	0.86841900	18.09451200	2.58753600
O	3.53083700	17.98553100	5.22707700
O	3.62579600	18.71238300	3.08438900
C	2.01373400	12.95725300	0.33793200
H	2.87542500	13.23290900	0.93346600
C	4.40214200	20.15350600	4.79923000
C	4.64673600	15.17160300	3.45335900
C	0.62540200	24.44564100	1.98139700
C	-0.54080800	20.53506300	3.38458600
C	5.78976500	14.20139600	3.25471400
C	5.46440200	11.93557000	2.16453100
H	5.43913300	11.47543900	3.14688300
C	0.29376300	18.17407800	3.72539600
C	2.19446700	12.63114700	-0.99573800
H	3.19319700	12.64209200	-1.40439600
C	6.49499500	13.68869200	4.48007900
H	6.81979500	12.65539600	4.48163900
H	6.12760200	14.05093000	5.43257000
C	-0.42590400	20.81623900	2.01904300
H	-0.62154100	20.04188300	1.29228900
C	-2.30003400	18.60559400	3.56442700
C	5.56208300	13.07245300	-0.36680300
H	5.58168600	13.53071700	-1.35117200
C	5.63338800	13.31868600	2.04558700
C	0.73901100	12.91925100	0.92297700
C	1.56563100	25.20064100	2.70147300
H	1.96181600	24.81123200	3.63211300
C	-0.87240300	19.13986700	3.83418700
C	5.32586200	11.13095600	1.03156600
H	5.20064800	10.05920300	1.14891300
C	5.36701500	11.68523900	-0.25487300
C	2.02947000	10.17034600	2.19938900
H	2.89175100	10.82414200	2.23973400
C	-1.83890500	18.91567800	4.96485700
H	-1.63416000	18.07386600	5.61503600
H	-2.23392800	19.79165600	5.46517700
C	-3.27081900	19.54528800	2.88605200
C	4.73432200	23.29672800	1.83312200
C	5.68206800	13.87628200	0.75945100
H	5.78880600	14.94531400	0.64408400
C	1.12012400	12.23103900	-1.80620300
C	4.56742500	21.19871900	3.73923200
C	5.85129100	22.91598500	2.58992200
H	6.80288200	23.41432400	2.43714600
C	1.19542900	14.50238600	2.85589900
C	-0.59855000	12.71595900	3.18080000
H	-0.91920500	13.36544100	3.98543800
H	-1.40277200	12.25179100	2.62588400
C	0.60429100	13.20090500	2.39005800
C	0.18280100	24.93614500	0.74038800
H	-0.54204100	24.36699900	0.16676600
C	3.80299100	18.85174000	4.34289600
C	-0.34038700	21.58095300	4.29316100
H	-0.46424400	21.40293700	5.35649600
C	0.67701800	11.96719300	3.35559600
C	7.21755600	14.68314800	3.61054600
C	5.37952700	20.03918100	5.95854700
H	5.54460300	19.03491800	6.32703600
H	6.26515700	20.66005500	5.92841600
C	-0.06662800	22.08062900	1.57453400
H	0.03604900	22.24924200	0.51039900
C	8.33126500	14.21171100	2.70756400
C	3.45343200	21.56837600	2.97042600
H	2.50492000	21.06577500	3.11911100
C	0.17332900	23.12589100	2.48051600
C	2.89515900	22.90249200	6.01434600
H	2.06657000	22.39005900	5.54192100
C	1.36081100	11.81532400	-3.20612600
C	8.64988500	14.94978900	1.55760000
H	8.09646300	15.85488000	1.33226000
C	0.79651900	10.61281900	2.69786600
C	-0.15725000	12.21895300	-1.22841200
H	-1.02146000	11.95763800	-1.83045100
C	3.53960600	22.58400000	2.03026800
H	2.65418700	22.85831600	1.47475700
C	0.66323500	26.14612500	0.24221900
H	0.30971500	26.50900900	-0.71840300
C	4.02399900	22.17361100	6.41214400
C	5.76780000	21.88265000	3.52512100
H	6.65245000	21.62077900	4.09489700
C	2.04689400	26.40856000	2.20037100
H	2.79238500	26.96329500	2.76175100
C	4.79263300	24.41380300	0.86383600
C	3.88603100	9.15175600	-2.64756800
H	3.08056500	8.42380800	-2.65579200
C	0.00902200	22.85785500	3.84830900
H	0.14076900	23.65574200	4.57193100
C	4.06244300	20.67321300	6.24057500
C	9.06503000	13.05406500	2.98330800
H	8.84798500	12.47718100	3.87577400
C	-4.12108500	20.37884700	3.62060600
H	-4.10876900	20.34380800	4.70446300
C	5.66102400	10.32752900	-3.78315300
H	6.25876500	10.50104200	-4.67303900
C	2.83309600	24.28032300	6.21110400
H	1.94832700	24.83034300	5.90505600
C	-3.33332600	19.59691900	1.48576500
H	-2.69128000	18.96346600	0.88732800
C	4.64813200	9.36659400	-3.79694000
H	4.44492000	8.80068500	-4.70050400
C	9.66169100	14.52970000	0.69776200
H	9.88642900	15.11016400	-0.19208500

C	3.97849800	24.41627600	-0.28095200
H	3.30553800	23.58794100	-0.46022800
C	-0.30357700	9.75237400	2.62619300
H	-1.26186400	10.07359000	3.02145500
C	2.15499100	8.89995600	1.64117500
H	3.11893800	8.56834900	1.26757100
C	3.90251500	24.95686700	6.80526300
H	3.85415600	26.03102500	6.95505200
C	5.02775600	24.23919200	7.20913500
H	5.86237500	24.75104500	7.67878800
C	0.53265000	10.87062300	-3.83412600
H	-0.28707100	10.42493900	-3.27974700
C	5.90817200	11.07034700	-2.62950300
H	6.70310200	11.81008100	-2.62140600
C	-0.34128100	12.55720200	0.11390900
H	-1.34319700	12.52787800	0.52755200
C	2.43898700	12.34040500	-3.93797800
H	3.08767800	13.07897500	-3.48126500
C	4.13552100	9.89038000	-1.49266800
H	3.50771700	9.75441000	-0.61951400
C	-4.99593100	21.25668200	2.97545800
H	-5.64773200	21.89386800	3.56587100
C	1.04760300	8.05024600	1.56376900
H	1.14658800	7.06206400	1.12516300
C	10.08354000	12.63205300	2.12490100
H	10.64234100	11.73051200	2.35778100
C	1.59698300	26.88743300	0.96917200
H	1.98421800	27.81980600	0.57106200
C	5.64843100	25.50874000	1.06975200
H	6.26345400	25.54696100	1.96314600
C	4.02132800	25.46916000	-1.19108000
H	3.37839100	25.44682400	-2.06576500
C	5.08418100	22.85647100	7.01642300
H	5.95925000	22.30602800	7.34605600
C	10.38282200	13.36526600	0.97705900
H	11.17162100	13.03637700	0.30741800
C	5.15127700	10.85957200	-1.46587200
C	4.88159800	26.54775900	-0.97781600
H	4.91562900	27.37016300	-1.68574700
C	5.69361800	26.56295100	0.15867900
H	6.35753600	27.40256100	0.34198800
C	-4.19495000	20.47927100	0.83889600
H	-4.20010800	20.51490000	-0.24603900
C	-5.03171400	21.31570400	1.58219100
H	-5.70464200	22.00464500	1.08056100
C	-0.18250400	8.48088300	2.05976500
H	-1.04931500	7.82852300	2.01209000
C	1.83881700	11.01038700	-5.86655100
H	2.02311100	10.70022700	-6.89063200
C	2.67648300	11.94233200	-5.25030300
H	3.51710500	12.36280000	-5.79388300
C	0.76561800	10.47557200	-5.15069500
H	0.11404700	9.74048500	-5.61408400
C	-2.49198700	17.16864200	3.16057500
C	-3.38036300	16.36061000	3.88033000
C	-1.89295800	16.65265000	2.00388500
C	-3.68620300	15.07280000	3.43916100
H	-3.84498500	16.75462500	4.77943600
C	-2.19394400	15.36463800	1.56249900
H	-1.19324600	17.26680500	1.45105300
C	-3.10326400	14.57761900	2.27082000
H	-4.38477000	14.46040900	4.00123200

H	-1.71447900	14.97028800	0.67328300
H	-3.35223900	13.58115700	1.92003100
C	3.06466600	19.97345300	7.12267700
C	3.46141300	19.52948400	8.38849000
C	1.72696200	19.83210000	6.74422900
C	2.53741600	18.94853200	9.25894700
H	4.49926100	19.63938400	8.68951400
C	0.80007000	19.25636600	7.61017100
H	1.41778300	20.14069600	5.75361900
C	1.20147400	18.81287600	8.87273700
H	2.85923900	18.60697800	10.23843500
H	-0.23044800	19.14147700	7.29268300
H	0.48014600	18.36434500	9.54929900
C	1.40824100	12.03093400	4.66914600
C	0.70784400	11.78959600	5.85602400
C	2.78955100	12.23706600	4.72851200
C	1.37432800	11.76173100	7.08208100
H	-0.36488300	11.62462600	5.81569900
C	3.46000600	12.20533100	5.94951400
H	3.33818500	12.45237100	3.81976600
C	2.75540700	11.96714900	7.13191100
H	0.81726100	11.57457100	7.99546600
H	4.53006300	12.37774000	5.97818700
H	3.27773800	11.94184100	8.08361800
C	7.45026700	16.07311600	4.13979900
C	8.20827300	16.23201000	5.30679700
C	7.04626700	17.21261300	3.43168500
C	8.58220500	17.50205000	5.74545400
H	8.51998900	15.35018400	5.85893600
C	7.42377000	18.48278500	3.86796800
H	6.43219000	17.09738700	2.54666800
C	8.20245200	18.63083100	5.01656600
H	9.17664800	17.60967000	6.64769100
H	7.10780900	19.35959300	3.31520100
H	8.50365100	19.61960000	5.34806500
C	3.01806400	17.42425300	0.35884900
C	4.42253300	17.85149000	0.16819300
C	2.34930000	16.71834500	-0.70641600
C	1.82230100	19.56075000	0.23249700
O	4.67527800	19.17473900	0.32081600
O	5.30052800	17.01812500	0.01739400
C	1.00528700	16.31151800	-0.52505800
C	2.95415300	16.47232300	-1.96450300
H	1.02415900	18.92301200	0.57422100
H	2.41873400	20.03960800	0.99657100
C	6.02102200	19.52375700	0.62693000
C	0.28004600	15.73955900	-1.55562900
H	0.53183000	16.48872200	0.42791700
C	2.24295800	15.87292500	-2.99547700
H	3.98698800	16.75585400	-2.12884700
H	5.97784700	20.25097300	1.43838100
H	6.57697100	18.63663900	0.92486500
C	6.77141900	20.16603200	-0.55028300
C	0.90089700	15.53771600	-2.78986200
H	-0.75679900	15.46358600	-1.41624500
H	2.70871500	15.69521500	-3.95693700
Cl	6.67204700	19.15193400	-2.02764600
Cl	8.49330700	20.30624000	-0.02163100
Cl	6.14981900	21.80660100	-0.91699900
Br	-0.12472200	14.85242600	-4.23069200
C	1.93537900	19.89797700	-1.06889400
C	1.05317100	19.28820900	-2.12315500

C	2.86331900	20.94809400	-1.59365100
H	1.66598300	18.79215600	-2.88573500
H	0.35822600	18.56148700	-1.71526200
N	0.28349700	20.33017600	-2.83534500
H	3.58583800	20.48144000	-2.27876700
H	3.43179600	21.39437100	-0.77951600
C	2.06735800	22.02629400	-2.36484000
C	1.12996000	21.40032200	-3.40390300
S	-1.14642100	20.82494300	-2.09896000
H	2.75959400	22.71035000	-2.86673400
H	1.47084000	22.61368900	-1.66054000
H	1.70651300	20.93230800	-4.20959700
H	0.48349500	22.15306400	-3.86057800
O	-1.56394000	19.71946400	-1.21846400
O	-1.03536800	22.18363800	-1.53401900
C	-2.25599300	20.91740000	-3.48745200
C	-3.01125000	22.07087900	-3.68228400
C	-2.39883600	19.80306300	-4.32074900
C	-3.92327300	22.10835800	-4.73782700
H	-2.88074700	22.91987700	-3.02181000
C	-3.31219200	19.85983200	-5.36539100
H	-1.79633500	18.91625400	-4.15746700
C	-4.08762000	21.01099000	-5.59061700
H	-4.51390600	23.00486800	-4.89946200
H	-3.42773800	19.00136400	-6.02050800
C	-5.06651700	21.05383700	-6.73546300
H	-4.54642100	20.97200200	-7.69629900
H	-5.63831300	21.98449700	-6.73763000
H	-5.77141600	20.21771200	-6.67986700

11. References

1. Law, J. A.; Bartfield, N. M.; Frederich, J. H., Site-Specific Alkene Hydromethylation via Protonolysis of Titanacyclobutanes. *Angew. Chem. Int. Ed.* **2021,** *60* (26), 14360-14364.
2. Cong, F.; Sun, G.-Q.; Ye, S.-H.; Hu, R.; Rao, W.; Koh, M. J., A Bimolecular Homolytic Substitution-Enabled Platform for Multicomponent Cross-Coupling of Unactivated Alkenes. *J. Am. Chem. Soc.* **2024,** *146* (15), 10274-10280.
3. Nóvoa, L.; Trulli, L.; Parra, A.; Tortosa, M., Stereoselective Diboration of Spirocyclobutenes: A Platform for the Synthesis of Spirocycles with Orthogonal Exit Vectors. *Angew. Chem. Int. Ed.* **2021,** *60* (21), 11763-11768.
4. Noji, S.; Hara, Y.; Miura, T.; Yamanaka, H.; Maeda, K.; Hori, A.; Yamamoto, H.; Obika, S.; Inoue, M.; Hase, Y.; Orita, T.; Doi, S.; Adachi, T.; Tanimoto, A.; Oki, C.; Kimoto, Y.; Ogawa, Y.; Negoro, T.; Hashimoto, H.; Shiozaki, M., Discovery of a Janus Kinase Inhibitor Bearing a Highly Three-Dimensional Spiro Scaffold: JTE-052 (Delgocitinib) as a New Dermatological Agent to Treat Inflammatory Skin Disorders. *J. Med. Chem.* **2020,** *63* (13), 7163-7185.
5. A. A. Kirichok, H. Tkachuk, K. Levchenko, D. Granat, T. Yegorova, D. Lesyk, A. Anisiforova, Y. Holota, V. Zomchak, I. Bodenchuk, V. Kosach, P. Borysko, R. A. Korzh, G. Al-Maali, V. Kubyshkin, H. S. Rzepa, P. K. Mykhailiuk, *Angew. Chem. Int. Ed.* **2025**, *64*, e202418850.
